# Multi-omics analysis reveals the glycolipid metabolism response mechanism in the liver of genetically improved farmed Tilapia (GIFT, *Oreochromis niloticus*) under hypoxia stress

**DOI:** 10.1186/s12864-021-07410-x

**Published:** 2021-02-06

**Authors:** Jun-Lei Ma, Jun Qiang, Yi-Fan Tao, Jing-Wen Bao, Hao-Jun Zhu, Lian-Ge Li, Pao Xu

**Affiliations:** 1 Wuxi Fisheries College, Nanjing Agricultural University, Wuxi, 214081 China; 2 Key Laboratory of Freshwater Fisheries and Germplasm Resources Utilization, Ministry of Agriculture and Rural Affairs, Freshwater Fisheries Research Center, Chinese Academy of Fishery Sciences, Wuxi, 214081 China

**Keywords:** Genetically improved farmed Tilapia, Hypoxia, Transcriptome, Metabolome, Glucose and lipid metabolism

## Abstract

**Background:**

Dissolved oxygen (DO) in the water is a vital abiotic factor in aquatic animal farming. A hypoxic environment affects the growth, metabolism, and immune system of fish. Glycolipid metabolism is a vital energy pathway under acute hypoxic stress, and it plays a significant role in the adaptation of fish to stressful environments. In this study, we used multi-omics integrative analyses to explore the mechanisms of hypoxia adaptation in Genetically Improved Farmed Tilapia (GIFT, *Oreochromis niloticus*).

**Results:**

The 96 h median lethal hypoxia (96 h-LH50) for GIFT was determined by linear interpolation. We established control (DO: 5.00 mg/L) groups (CG) and hypoxic stress (96 h-LH50: 0.55 mg/L) groups (HG) and extracted liver tissues for high-throughput transcriptome and metabolome sequencing. A total of 581 differentially expressed (DE) genes and 93 DE metabolites were detected between the CG and the HG. Combined analyses of the transcriptome and metabolome revealed that glycolysis/gluconeogenesis and the insulin signaling pathway were down-regulated, the pentose phosphate pathway was activated, and the biosynthesis of unsaturated fatty acids and fatty acid metabolism were up-regulated in GIFT under hypoxia stress.

**Conclusions:**

The results show that lipid metabolism became the primary pathway in GIFT under acute hypoxia stress. Our findings reveal the changes in metabolites and gene expression that occur under hypoxia stress, and shed light on the regulatory pathways that function under such conditions. Ultimately, this information will be useful to devise strategies to decrease the damage caused by hypoxia stress in farmed fish.

**Supplementary Information:**

The online version contains supplementary material available at 10.1186/s12864-021-07410-x.

## Background

Fish growth is affected by environmental elements such as dissolved oxygen (DO), temperature, salinity, the ammonia nitrogen concentration, and pH. In recent years, the effects of hypoxia stress on aquatic organisms have become increasingly severe [1]. This is because the DO in water is prone to decrease as a result of high-density farming, excessive feeding, and blue algae blooms that occur in fish farmed for breeding or for the commercial market [2]. Low DO affects the growth and feed utilization of aquatic organisms and disrupts their morphology, physiology, and behavioral adaptability [3, 4]. Compared with mammals, some aquatic animals have a strong ability to tolerate low oxygen levels [5–7]. However, in general, the normal life processes and body development of aquatic animals can be guaranteed when the DO concentration is higher than 4 mg/L [8].

Fish adapt to hypoxia stress through a series of complex physiological and biochemical processes, including a reduction in the metabolic rate, changes in metabolic pathways, and an improvement in the oxygen transport capacity [9, 10]. Many studies have found that when an organism is in an anoxic environment, glycolipid metabolism undergoes a series of changes [11–14]. Anaerobic metabolism can reduce oxygen consumption and quickly provide energy under hypoxia stress [15]. Previous studies have shown that turbot (*Scophthalmus maximus*) and largemouth bass *(Micropterus salmoides*) switch to anaerobic metabolism under hypoxia stress, causing a decrease in liver glycogen levels and an increase in lactic acid in the blood [16, 17]. Genetically Improved Farmed Tilapia (GIFT, *Oreochromis niloticus*) preferentially use triglyceride (TG) and serum glucose (GLU) to provide energy under acute hypoxia stress [18]. The compensation mechanism of fish under hypoxia stress is connected with increased lipid metabolism [19]. In obese mice, hypoxia stimulates the lipolysis of adipocytes and inhibits the absorption of free fatty acids (FFA) by adipocytes, leading to increased fatty acid concentrations in the plasma [20]. Other studies have found that chronic hypoxia inhibits glycolysis and increases lipolysis, revealing that lipid metabolism is the main energy supply pathway under chronic hypoxia stress [21, 22].

Tilapia, as the main freshwater fish in southern China, has a rapid growth rate and a high economic value. Tilapia is an ideal model to explore the effects of hypoxia stress on fish, because it is mainly cultivated in high-density ponds and shows strong adaptability to hypoxia [23]. There are different types of metabolism under acute hypoxia and long-term hypoxia stress. In Nile tilapia, different energy supply pathways function under short-term and long-term hypoxic conditions; carbohydrates supply energy during short-term hypoxia stress, and lipids become the primary energy source during long-term hypoxia stress [21]. Mahfouz et al. [2] found that glycolysis decreases and gluconeogenesis increases in the liver and muscle of tilapia under acute hypoxia stress. Long-term hypoxia stress has also been shown to affect the fatty acid composition and blood biochemical indicators of GIFT [24].

In the post-genomic era, the widespread use of transcriptome, proteome, and metabolome analyses has led to new insights into biology [25]. Transcriptomics is the study of gene expression at the RNA level, and provides information about differentially expressed (DE) genes and gene function [26]. Metabolomics explains the metabolic processes that occur organisms after stimulation or disruption by providing information about the types and metabolites and changes in their concentrations [27]. In recent years, multi-omics analysis methods have been used to study the stress responses of aquatic animals. For example, combined metabolome and transcriptome analyses of *Litopenaeus vannamei* revealed the relationships between certain secondary metabolites and the transcript levels of DE genes, thereby clarifying the mechanism of nitrite tolerance [28]. Combined metabolome and transcriptome analyses of tilapia under hypoxic stress have provided information about changes in liver metabolism and the cause of death. In the present study, we used a multi-omics method to analyze how glycolipid metabolism changes in GIFT liver tissues under hypoxia stress, and used qRT-PCR to verify the transcriptional patterns of eight DE genes related to glycolipid metabolism. This is the first report of combined transcriptome and metabolome analyses of the GIFT liver. Our results reveal changes in energy supply pathways under hypoxia stress, and provide information about the mechanisms of hypoxia adaptation and hypoxia tolerance in GIFT.

## Results

### Determination of 96 h-LH50 in GIFT

A decrease in DO (from 3.2 to 0.2 mg/L) significantly increased the mortality of GIFT (Table 1). When the DO was 3.2 or 1.6 mg/L, no fish death occurred within 96 h. However, GIFT began to die at 96 h when the DO level was 0.8 mg/L. When the DO level was 0.4 mg/L, the cumulative mortality at 12 h was 20%; and it gradually increased over time to 36.7 and 56.7% at 48 h and 96 h, respectively. When the DO level was 0.2 mg/L, the cumulative mortality at 96 h was 86.7%. The 96 h-LH50 calculated by linear interpolation was 0.536 mg /L. The linear regression equation was y = 18.696 + 58.406x (*R* = 0.868, *P* < 0.0001). On the basis of this result, we chose 0.55 mg/L as the 96 h-LH50 of GIFT for further experiments.

**Table 1:** Effects of different low dissolved oxygen levels on 96 h mortality of GIFT

Time (h)	Dissolved oxygen levels (mg/L)				
	3.2	1.6	0.8	0.4	0.2
0	0	0	0	0	0
6	0	0	0	0	0
12	0	0	0	20	23.3
24	0	0	0	26.7	40
48	0	0	0	36.7	67.7
96	0	0	6.7	56.7	86.7

### Hepatic biochemical indicators

**Fig. 1:**
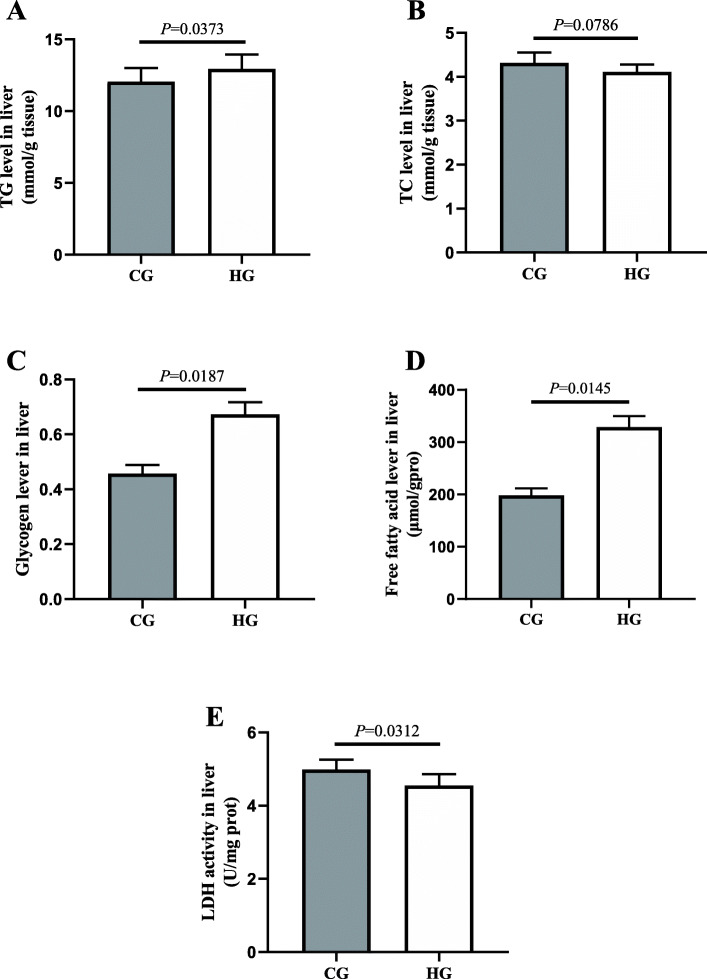
Glucose and lipid metabolism indicators in liver of GIFT under 96 h acute hypoxia stress (*n* = 9 replicates per group). **a** triglyceride, TG; **b** total cholesterol, TC; **c** glycogen; **d** free fatty acids, FFAs; **e** lactate dehydrogenase, LDH. Asterisk (*) indicates significant difference (*P* < 0.05) between hypoxia stress group (HG) and control group (CG)

Figure 1 shows the changes in hepatic cholesterol (TC), triglyceride (TG), free fatty acids (FFA), and glycogen levels and lactate dehydrogenase (LDH) activity in GIFT after 96 h at DO 0.55 mg/L. At 96 h, the TG was significantly higher (*P*=0.0373< 0.05) in the hypoxia-stressed group (HG) than in the control group (CG) (Fig. 1a). Compared with the CG, the HG showed increased TC levels (Fig. 1b), but the difference was not significant (*P*=0.0786> 0.05). Hepatic glycogen (*P*=0.0187< 0.05) (Fig. 1c) and FFA levels (*P*=0.0145< 0.05) (Fig. 1d) were significantly higher in the HG than in the CG at 96 h. Compared with the CG, the HG showed significantly lower (*P*=0.0312< 0.05) hepatic LDH activity at 96 h (Fig. 1e).

### Metabolome profiles in liver of hypoxia stressed GIFT

The liver tissues were collected from the HG and CG for LC-MS analysis. The total ion chromatograms, m/z peak width, and retention time peak width of metabolites detected in liver samples from CG or HG in positive (POS) and negative (NEG) modes are shown in Fig. S1 and Fig. S2, respectively. These figures show the reliability of the metabolomics data in this trial and the stable performance of the UPLC-MS analyses.

**Fig. 2:**
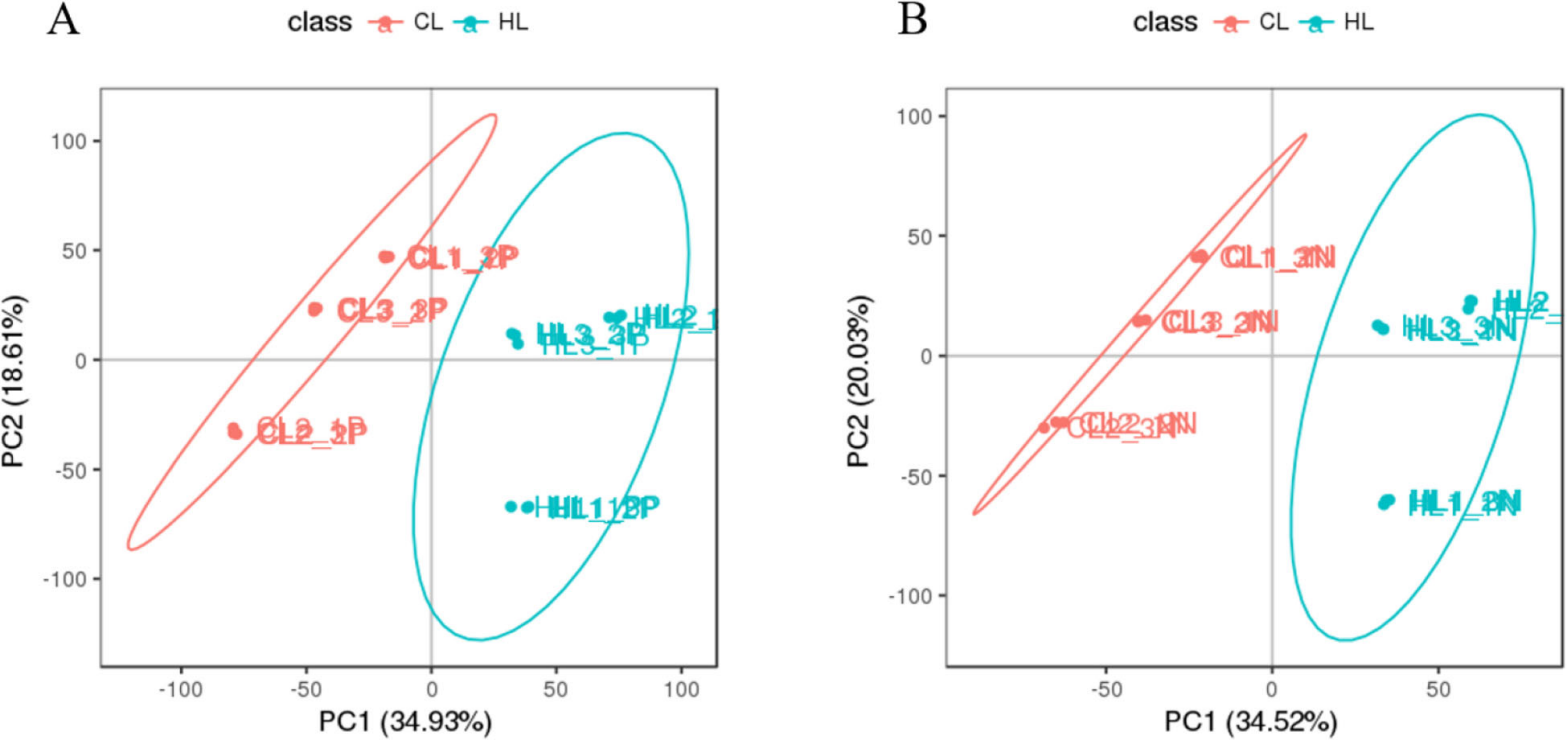
Principal component analysis (PCA) score plot of LC-MS data from profiles of GIFT hepatic metabolites detected in positive ion mode (**a**) and negative ion mode (**b**). Red and green points represent samples in control group (CG) and hypoxia-stressed group (HG), respectively

**Fig. 3:**
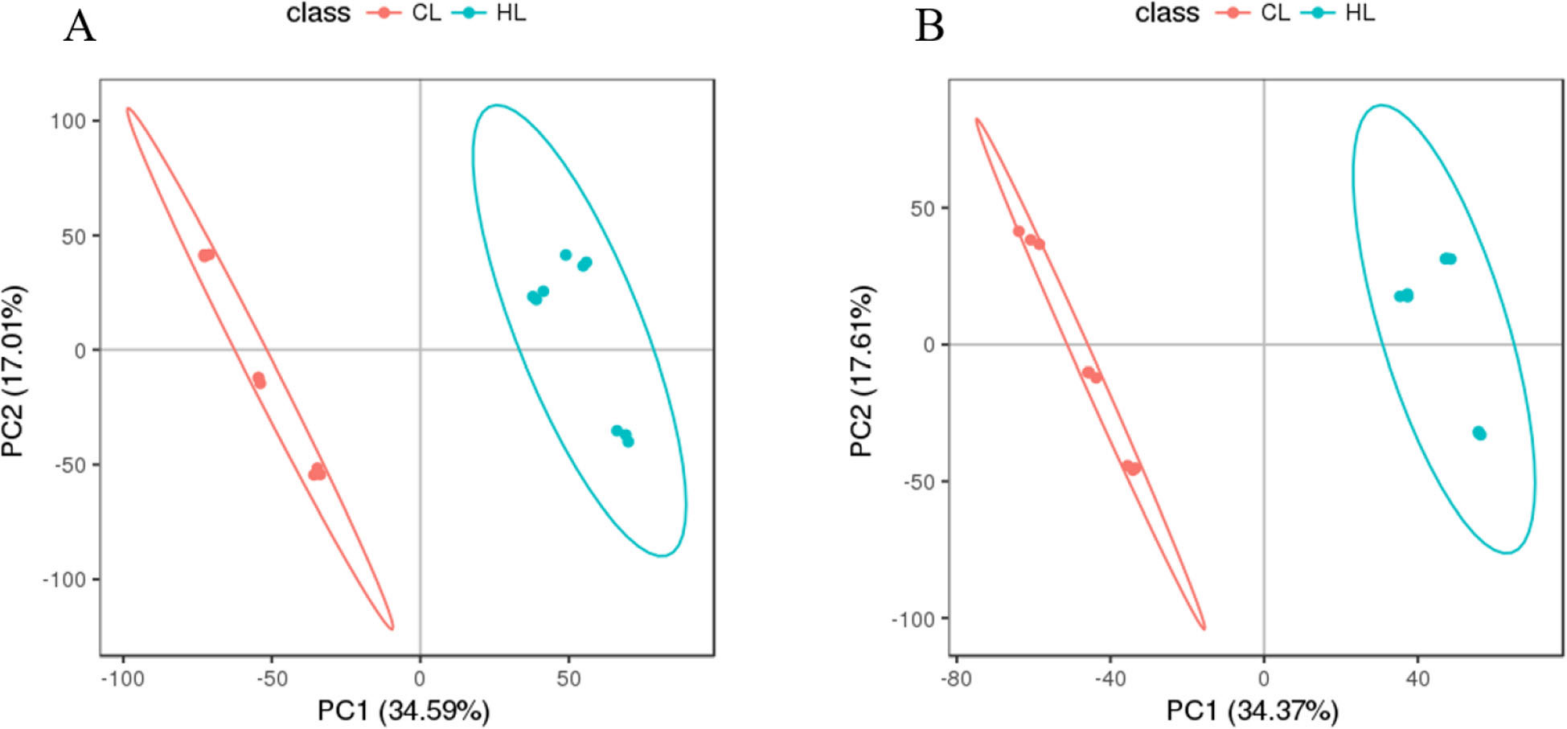
Partial least square discriminant analysis (PLSDA) score plot of LC-MS data from profiles of GIFT hepatic metabolites detected in positive ion mode (**a**) and negative ion mode (**b**). Red and green points represent samples in control group (CG) and hypoxia-stressed group (HG), respectively

A total of 13,765 and 10,308 features were obtained in the POS and NEG mode, respectively. After the data were processed and filtered, 11,303 and 8353 high-quality features were obtained in the POS and NEG mode, respectively (Table 2). The high-quality features were analyzed using multiple statistical methods, including principal component analysis (PCA) and partial least square discriminant analysis (PLSDA). Unsupervised PCA was performed prior to PLSDA. The PCA score plot (Fig. 2) revealed tight clustering for the quality control (QC) sample, indicative of good stability of the metabolic profiles. The first two primary components (PC1 and PC2) explained 53.54 and 54.55% of the PCA model in the POS and NEG modes, respectively, indicating that the high-quality features of the CG and HG were naturally separated and clustered (Fig. 2). The PLSDA models were further used to distinguish differences in metabolites among samples detected in the CG and HG in POS and NEG modes (Fig. 3). After 200 permutation tests, the R^2^ values were 0.9890 or 0.9913 and the corresponding Q2 values were 0.9654 or 0.9481 in the POS and NEG mode, respectively, indicating good credibility of the PLSDA models.

**Table 2:** Statistics for quantitative features. Mode indicates that the mode of MS analysis is mainly divided into a positive ion mode and negative ion mode; features with a VIP value> 1, FC > 2 or < 0.5, and an adjusted *P*-value < 0.05 were considered to be up- and down-regulated, respectively, in response to the HG

Mode	Total feature	High-Quality feature	Up-regulated features	Down-regulated features
POS	13,765	11,303	814	861
NEG	10,308	8353	782	571
Tatal	24,073	19,656	1596	1432

High-quality metabolites were selected for the DE metabolites analysis. The DE metabolites were identified on the basis of multiple statistical analyses. Metabolites with a variable importance in the projection (VIP) value> 1, fold change (FC) > 2 or < 0.5, and an adjusted *P*-value < 0.05 were considered to be significant. A total of 3028 DE features were identified in the comparative analysis between the HG and the CG, of which 1596 were up-regulated and 1432 were down-regulated in the HG compared with the CG (Table 2). We matched the obtained DE features at online databases such as Kyoto Encyclopedia of Genes and Genomes (KEGG) and the human metabolome database (HMDB), and further validated them by comparison with our in-house fragment spectrum library. In total, 93 DE metabolites were identified by MS2, with 76 and 17 in the POS and NEG modes, respectively. We detected 15 DE metabolites (Table 3) in the glucose and lipid metabolism pathways, of which five metabolites were down-regulated and eight were up-regulated.

**Table 3:** Metabolites in GIFT liver showing significant differences in abundance between HG and CG groups

Compound name	RT	Mass	mode	FC	VIP	Corrected *p* value	Trend
Stearoylcarnitine	141.669	428.372	POS	0.114	2.437	0.000	down
Stearic acid	112.689	307.265	POS	0.238	2.080	0.000	down
N-Acetyl-D-glucosamine	441.047	186.075	POS	2.589	1.679	0.000	up
Myricetin	411.039	319.044	POS	2.148	1.456	0.000	up
L-Carnitine	382.561	162.111	POS	0.329	1.028	0.042	down
Lathosterol	120.906	369.354	POS	0.210	1.843	0.000	down
Cholic acid	224.118	391.283	POS	3.804	1.668	0.000	up
1-Octadecanoyl-sn-glycero-3-phosphocholine	155.672	524.369	POS	2.558	1.558	0.000	up
1-Stearoyl-sn-glycerol 3-phosphocholine	195.867	568.339	POS	0.168	2.336	0.000	down
1-Myristoyl-sn-glycero-3-phosphocholine	198.433	468.307	POS	2.044	1.346	0.000	up
N-Acetyl-D-Glucosamine 6-Phosphate	344.593	301.061	NEG	3.750	1.950	0.000	up
D-gluconate	273.289	195.055	NEG	2.083	1.403	0.000	up
Alpha-D-Glucose	346.297	239.076	NEG	2.364	1.486	0.000	up
Phosphatidylinositol	34.376	865.56	NEG	0.321	1.831	0.000	down
Farnesyl pyrophosphate	515.046	763.241	NEG	0.462	1.316	0.000	down

### Gene expression profiles in liver of hypoxia-stressed GIFT

We established and sequenced six mRNA libraries, three from the DO 5.00 mg/l control groups (CG-1, CG-2, and CG-3) and three from the 96 h-LH50 groups (HG-1, HG-2, and HG-3). The biological replicates had good repeatability. After removing low-quality raw sequences, there were 57,905,522, 46,781,934, 42,604,052, 38,581,554, 44,305,838, and 51,230,978 clean reads for the CG-1, CG-2, CG-3, HG-1, HG-2, and HG-3 libraries, respectively (98.05–98.89% valid data; Q20 values of 99.65–99.79%; Q30 values of 95.33–96.54%, and GC contents of 46.5–47%) (Table 4). The number of reads that mapped to the Nile tilapia genome was 44,797,588 (CG-1), 37,629,408 (CG-2), 32,951,044 (CG-3), 31,304,433 (HG-1), 35,303,322 (HG-2), and 41,293,836 (HG-3). More reads mapped to exon regions than to intron and intergenic regions in the genome (Fig. S3).

**Table 4:** Overview of reads for mRNA-seq of GIFT and quality filtering

Sample	Raw Data		Valid Data		Valid%	Q20%	Q30%	GC%
	Read	Base	Read	Base				
HL1	39,014,868	5.85G	38,581,554	5.79G	98.89	99.79	96.62	47
HL2	44,823,970	6.72G	44,305,838	6.65G	98.84	99.78	96.19	46.5
HL3	51,877,956	7.78G	51,230,978	7.68G	98.75	99.65	95.33	47.5
CL1	59,054,504	8.86G	57,905,522	8.69G	98.05	99.70	96.30	46.5
CL2	47,474,440	7.12G	46,781,934	7.02G	98.54	99.66	95.86	47.5
CL3	43,224,404	6.48G	42,604,052	6.39G	98.56	99.76	96.54	46.5

**Fig. 4:**
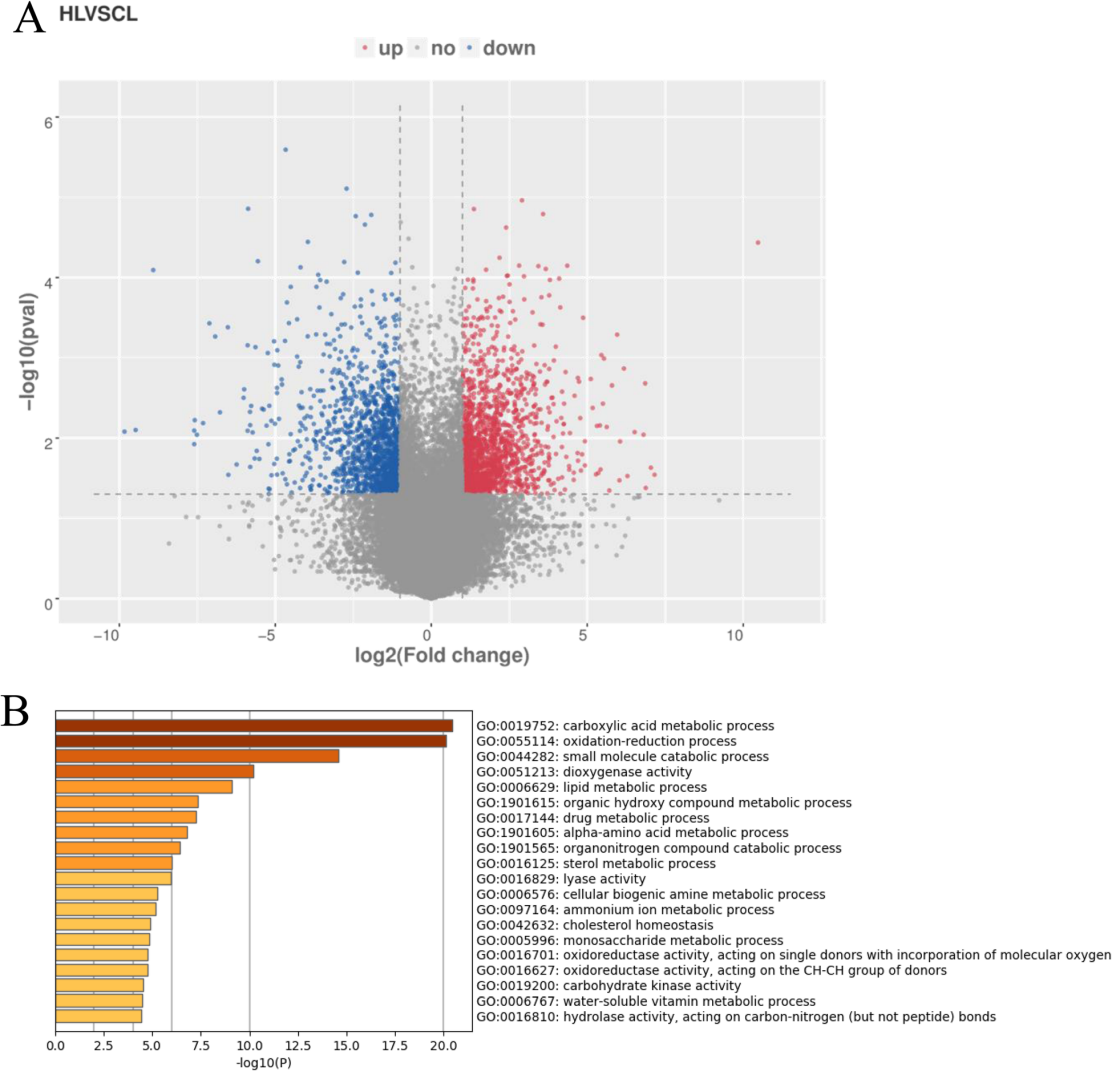
Differentially expressed (DE) genes and related Gene Ontology (GO) terms in liver of GIFT under hypoxia stress. **a** DE gene volcano plot graph. **b** Enriched GO terms based on DE genes. Red and blue dots in volcano plot graph represent up-regulated and down-regulated DE genes, respectively. X and y-axes represent log2(FC) value and -log10(P) value, respectively

Genes with *P*< 0.05, fold-change≥2 or ≤0.5, and fragments per kilobase of exon model per million mapped reads (FPKM) > 10 were considered to be DE genes. We identified 2375 DE genes between the CG and HG libraries, of which 1201 were up-regulated and 1174 were down-regulated (Fig. 4a). A list of DE genes is provided in Table S1. The enrichment analysis of DE genes was conducted using tools at the Metascape database. In the Gene Ontology (GO) enrichment analysis, the GO pathways most enriched with DE genes were carboxylic acid metabolic process, oxidation-reduction process, small molecule catabolic process, dioxygenase activity, lipid metabolic process, and monosaccharide metabolic process. These results show that acute hypoxia stress strongly affects the immune regulation and metabolism of GIFT (Fig. 4b).

**Fig. 5:**
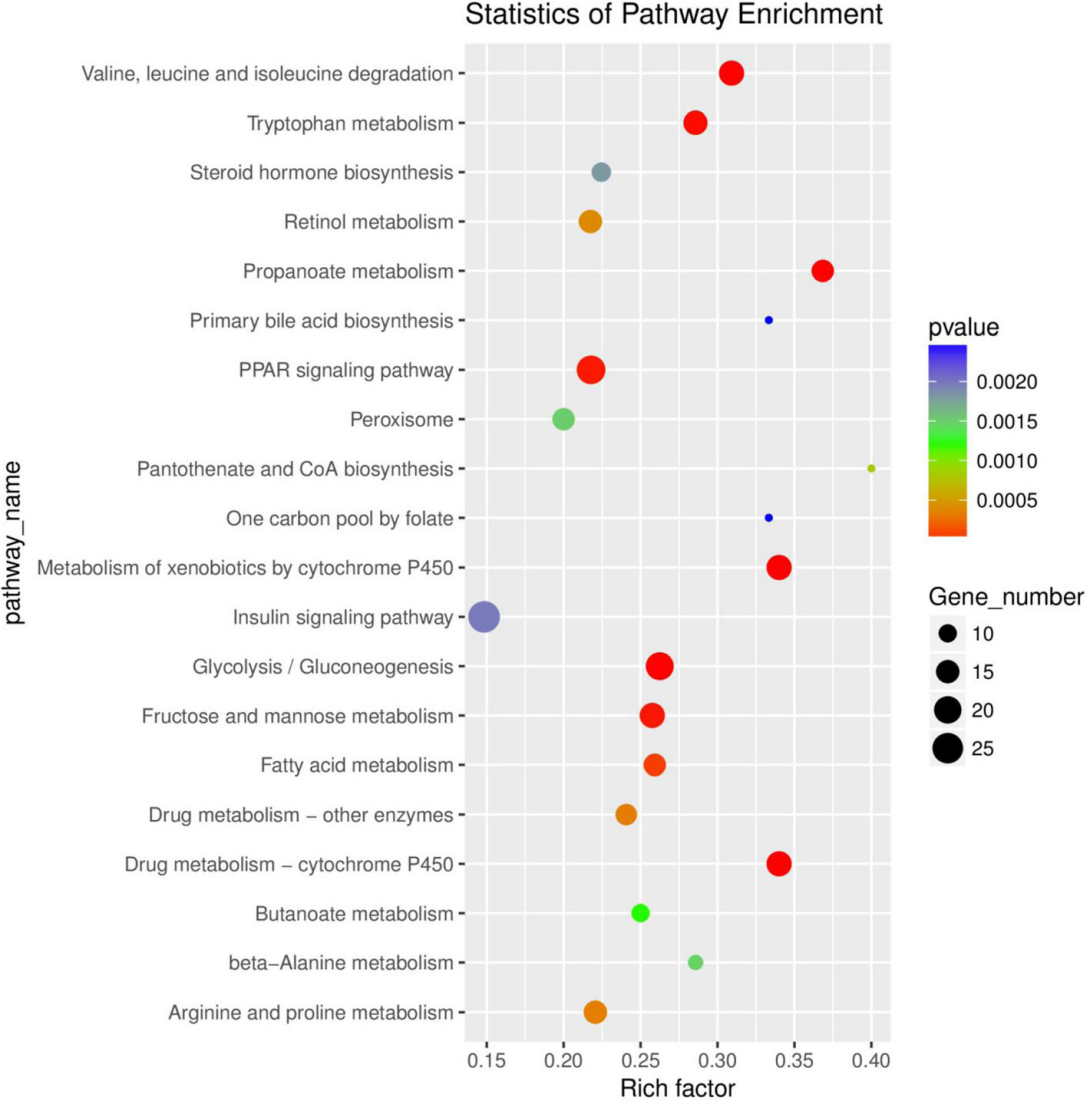
Kyoto Encyclopedia of Genes and Genomes (KEGG) pathway enrichment analysis of differentially expressed (DE) genes in liver of GIFT exposed to acute hypoxia stress

We identified 581 DE genes using tools at the KEGG database. The KEGG pathway enrichment analysis of DE genes identified 20 pathways enriched with DE genes under hypoxia stress (*P*< 0.05, Table S2) (Fig. 5). The DE genes were mainly enriched in the metabolism, organism system, and immune regulation categories, and the main pathways were the glucose, lipid, amino acid, and vitamin metabolic pathways. The specific pathways enriched with DE genes were the insulin signaling pathway, glycolysis/gluconeogenesis, and fatty acid metabolism.

### Integrated transcriptome and metabolome analysis

**Fig. 6:**
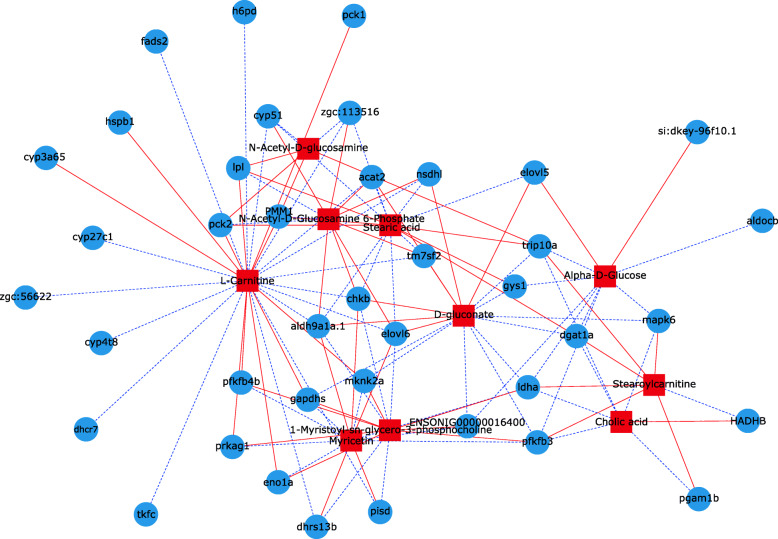
Correlation analysis between differentially expressed (DE) genes and DE metabolites in liver of GIFT under hypoxia stress. Blue squares represent DEGs; red circles represent DE metabolites; solid red line represents positive correlation between metabolites and genes; blue dotted line represents negative correlation between metabolites and genes

We identified the DE metabolites and genes in the same biological pathway. Five representative pathways of glucose and lipid metabolism are shown in Fig. S4, S5, S6, S7, S8. The main DE metabolites and top 40 DE genes in the glucose and lipid metabolism pathways were selected. Pearson’s correlation coefficient analyses were performed using the screened DE metabolites and genes. The selected DE metabolites and genes in the glucose and lipid metabolism pathways were subjected to correlation analyses (Fig. 6).

### Validation of selected DE mRNAs by qRT-PCR

**Fig. 7:**
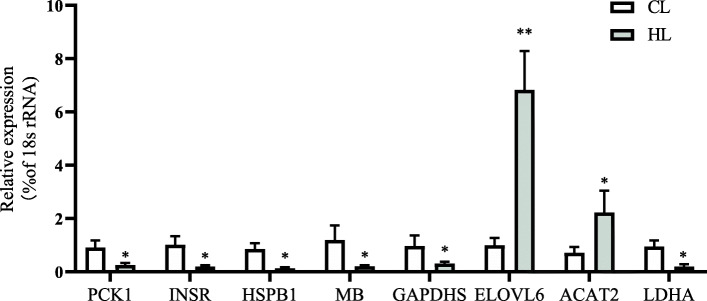
qRT-PCR analysis of differentially expressed genes in control group (CG) and hypoxia-stressed group (HG) (*n* = 9 replicates per group). Asterisk (*) indicates significant difference (*P*< 0.05) between HG and CG

We conducted qRT-PCR analyses to validate the transcriptional patterns of eight DE genes (Table 5) involved in lipid metabolism under hypoxic stress. The changes in gene expression detected by qRT-PCR were consistent with those detected from the sequencing results. The transcript levels of *ELOVL6* (encoding elongation of the very long chain fatty acid protein 6; *ELOVL6*) and *ACAT2* (encoding acyl-coenzyme A: cholesterol acyltransferase 2; *ACAT2*) were significantly higher in the HG than in the CG. The transcript levels of genes encoding phosphoenolpyruvate carboxykinase 1 (*PCK1*), insulin receptor (*INSR*), heat shock protein family B (small) member 1 (*HSPB1*), myoglobin (*MB*), glyceraldehyde-3-phosphate dehydrogenase (*GAPDHS*), and lactate dehydrogenase A (*LDHA*) were significantly lower in HG than in CG (Fig. 7).

**Table 5:** Differentially expressed mRNA verified by mRNA-Seq. Fold change= HG group (mean)/CG group (mean), where “mean” is the mean of three biological replicates

Gene abbreviation	Gene description	Log2 (fold_change)	Regulation (HG vs CG)
PCK1	phosphoenolpyruvate carboxykinase 1	−7.43	down
INSR	insulin receptor	−1.68	down
HSPB1	heat shock protein family B (small) member 1	−4.69	down
MB	myoglobin	−5.57	down
GAPDHS	glyceraldehyde-3-phosphate dehydrogenase, spermatogenic	−3.87	down
ELOVL6	ELOVL fatty acid elongase 6	2.85	up
ACAT2	acetyl-CoA acetyltransferase 2	2,72	up
LDHA	lactate dehydrogenase A	−2.07	down

## Discussion

A hypoxic environment can cause respiratory and metabolic disorders in fish, leading to increased mortality [29, 30]. Fish have evolved a variety of adaptation mechanisms, and adapt to the hypoxic environment by altering their energy supply and metabolic pathways [8, 31]. In this study, we researched responses in the GIFT liver to acute hypoxia stress through transcriptome and metabolome sequencing. We screened eight DE genes involved in the GIFT response to acute hypoxia stress and verified their transcriptional patterns by qRT-PCR. Relevant biochemical indexes and the activity of key enzymes were determined to clarify the effects of hypoxia on the glycolipid metabolic pathways of GIFT.

Carbohydrate metabolism is the main energy pathway for animals in an unstable environment [32, 33]. Previous studies have shown that fish have increased anaerobic metabolism and inhibited aerobic metabolism in the early stage of acute hypoxia stress [18, 21, 34]. For example, *Trichogaster microlepis* exposed to 12 h of hypoxia stress showed a decline in blood glucose and an increase in glucose metabolism to meet the body’s energy requirements [35]. In this study, we found that DE genes under hypoxia stress were enriched in several vital glucose metabolism pathways, including glycolysis/gluconeogenesis, the insulin signaling pathway, and the pentose phosphate pathway. Glycolysis is the main pathway of anaerobic metabolism [36]. Glyceraldehyde-3-phosphate dehydrogenase (GAPDH) is a multifunctional enzyme in glycolysis [37, 38]. It can catalyze the mutual conversion of 1,3-diphosphoglyceric acid and 3-phosphoglycerate and it participates in multiple biological processes, including DNA repair, membrane fusion and transport, RNA binding, autophagy, cell death cytoskeleton dynamics, and cytoskeleton dynamics [39]. Inactivation of GAPDHS can convert the carbohydrate decomposition process in cells from glycolysis to the pentose phosphate pathway, leading to the generation of NADPH and protection of cells against oxidative stress [40]. Lactate dehydrogenase (LDH), a marker enzyme of anaerobic metabolism, catalyzes the conversion of pyruvate and lactic acid in vivo. Its activity represents the level of anaerobic metabolism to a certain extent [41]. LDHA is a subunit of LDH, which is mainly expressed in anaerobic tissues of the liver [42]. A high concentration of pyruvate can increase the transcriptional levels and activity of LDHA. The oxygen partial pressure in tissue cells affects LDH activity. Differences in MB content reflect the oxygen supply to tissue cells, and thus indirectly reflect the LDH activity [43]. In this study, we detected down-regulation of *GAPDHS, LDHA*, and *MB* and decreased hepatic LDH activity in the GIFT liver at 96 h of hypoxia stress. Metabolome analyses showed that the level of alpha-D-glucose in the glycolytic pathway was significantly increased in hypoxia-stressed GIFT. Alpha-D-glucose is an important initial metabolite in the glycolysis pathway. In this study, the significant increase in alpha-D-glucose was consistent with the down-regulation of important regulatory genes in the glycolysis pathway, indicating that the glycolysis pathway was weakened under 96 h hypoxia stress. This would reduce the content of lactic acid, thereby reducing the damage caused by lactic acid accumulation in GIFT under acute hypoxia stress and reducing acidosis. Down-regulation of *GAPDHS* may be related to reducing oxidative stress injury. The results of this study are inconsistent with those of Su et al. [44], who detected a negative correlation between LDH activity and *MB* expression. It may be that the severe oxidative stress weakened the liver regulation capacity of GIFT, leading to decreases in both anaerobic metabolism and oxygen transport capacity after 96 h in a hypoxic environment (DO, 0.55 mg/L). Under acute hypoxic stress, the gluconeogenesis pathway in the fish liver may be inhibited [8, 45]. PCK1 can catalyze the production of phosphoenolpyruvate from oxaloacetic acid, releasing GDP and carbon dioxide. PCK1 is a rate-limiting enzyme that regulates gluconeogenesis, which participates in maintaining blood glucose levels and is considered indispensable for glucose homeostasis [46, 47]. The down-regulation of *PCK1* detected in this study may lead to the inhibition of the gluconeogenic pathway and reduced generation of glucose and glycogen.

The insulin signaling pathway is a key pathway for the maintenance of glucose homeostasis in fish. Insulin is an essential hormone that controls critical energy functions such as glucose and lipid metabolism, and it is perceived by the insulin receptor, INSR. Only when insulin and INSR are combined can they play a role in lowering blood sugar. Any impairment of the relationship between INS and INSR will lead to insulin resistance [48]. *HSPB1* encodes the heat shock protein HSP27, which is a member of the heat shock protein (HSP) family [49, 50]. This protein plays pivotal roles in apoptosis, insulin resistance, and inflammatory responses [51]. We detected significant down-regulation of *INSR* and *HSPB1* in the liver of GIFT under hypoxia stress. We speculate that this may inhibit glucose consumption in GIFT, and may result in hypoxia-induced damage to GIFT by triggering inflammation and insulin resistance. These results confirmed that in GIFT under hypoxia stress, anaerobic glucose metabolism is weakened and glucose homeostasis is imbalanced, which may be related to the increased death rate at 96 h.

The metabolome analyses revealed increased contents of D-gluconate, N-acetyl-D-glucosamine, and N-acetyl-D-glucosamine-6-phosphate in the pentose phosphate pathway, indicating that this pathway was activated under acute hypoxia stress, similar to the results reported elsewhere [52]. Besides providing energy, the pentose phosphate pathway is also the main source of NADPH and ribose in the body [53], and it can provide raw materials for the synthesis of fatty acids, ribonucleic acids, and cholesterol. A previous study showed that NADPH functions as a reducing agent to reduce ROS and alleviate oxidative damage in the tilapia liver caused by acute hypoxia stress [54]. In this study, the pentose phosphate pathway was activated in GIFT under hypoxia stress. This pathway may consume glucose, which would help to maintain glucose homeostasis and, at the same time, produce NADPH to reduce oxidative damage.

Studies have shown that under long-term hypoxia stress in fish, lipid metabolism becomes the primary energy metabolism pathway [19, 34]. In this study, some of the DE genes in the liver of GIFT in response to acute hypoxia stress were related to the biosynthesis of unsaturated fatty acids and fatty acid degradation. Therefore, we verified the transcript levels of *ACAT2* and *ELOVL6* by qRT-PCR, and measured TC, TG, and FFA concentrations as indicators of liver function. The elongase of very long-chain fatty acids gene family comprises *ELOVL1–7*, and different members show catalytic activity towards different fatty acid substrates [55, 56]. In animals, *ELOVL6* is one of the key genes in the biosynthesis of unsaturated fatty acids and its expression levels are related to regulation of this pathway. It catalyzes the extension of saturated fatty acids and monounsaturated fatty acids, and is an essential rate-limiting enzyme for long-chain fatty acids synthesis [57]. *ACAT2* is a key enzyme that regulates cholesterol homeostasis in cells by catalyzing the synthesis of cholesterol esters, and it is specifically expressed in the liver [58]. It can also participate in β-oxidation and lipid metabolism [59]. In this study, key lipid metabolism genes such as *ELOVL6* and *ACAT2* were up-regulated, indicating that lipid metabolism-related pathways were activated in GIFT under hypoxia stress. This is consistent with the results of Sun [60] and others who found that lipid metabolism was enhanced in largemouth bass (*Micropterus salmoides*) under acute hypoxia stress. The transcriptional upregulation of *ELOVL6* may activate the long-chain fatty acid synthesis pathway and increase the synthesis of n-3 and n-6 fatty acids. Interestingly, the content of stearic acid decreased, which may be due to the simultaneous increase in fatty acid synthesis and degradation. A greater metabolic rate than biosynthesis rate would lead to a decrease in the fatty acid content. Recent studies reported that the TG content in the liver and lipid peroxidation were increased in goby (*Gillichthys mirabilis*) and carp (*Cyprinus carpio*) under hypoxia stress [14, 61]. We obtained similar results in this study, in that the TG and FFAs contents were increased in the liver of GIFT at 96 h of acute hypoxic stress, while the TC content was not significantly changed. Similar results have been reported for largemouth bass under hypoxia stress [34]. These results indicate that hypoxia stress activated the unsaturated fatty acid biosynthetic pathway in the GIFT liver, leading to increased lipid production. Our results show that the biosynthesis of unsaturated fatty acids and the fatty acid degradation pathway were up-regulated under 96 h acute hypoxic stress, indicating that GIFT rely on lipid metabolism to provide energy under acute hypoxic stress.

## Conclusions

**Fig. 8:**
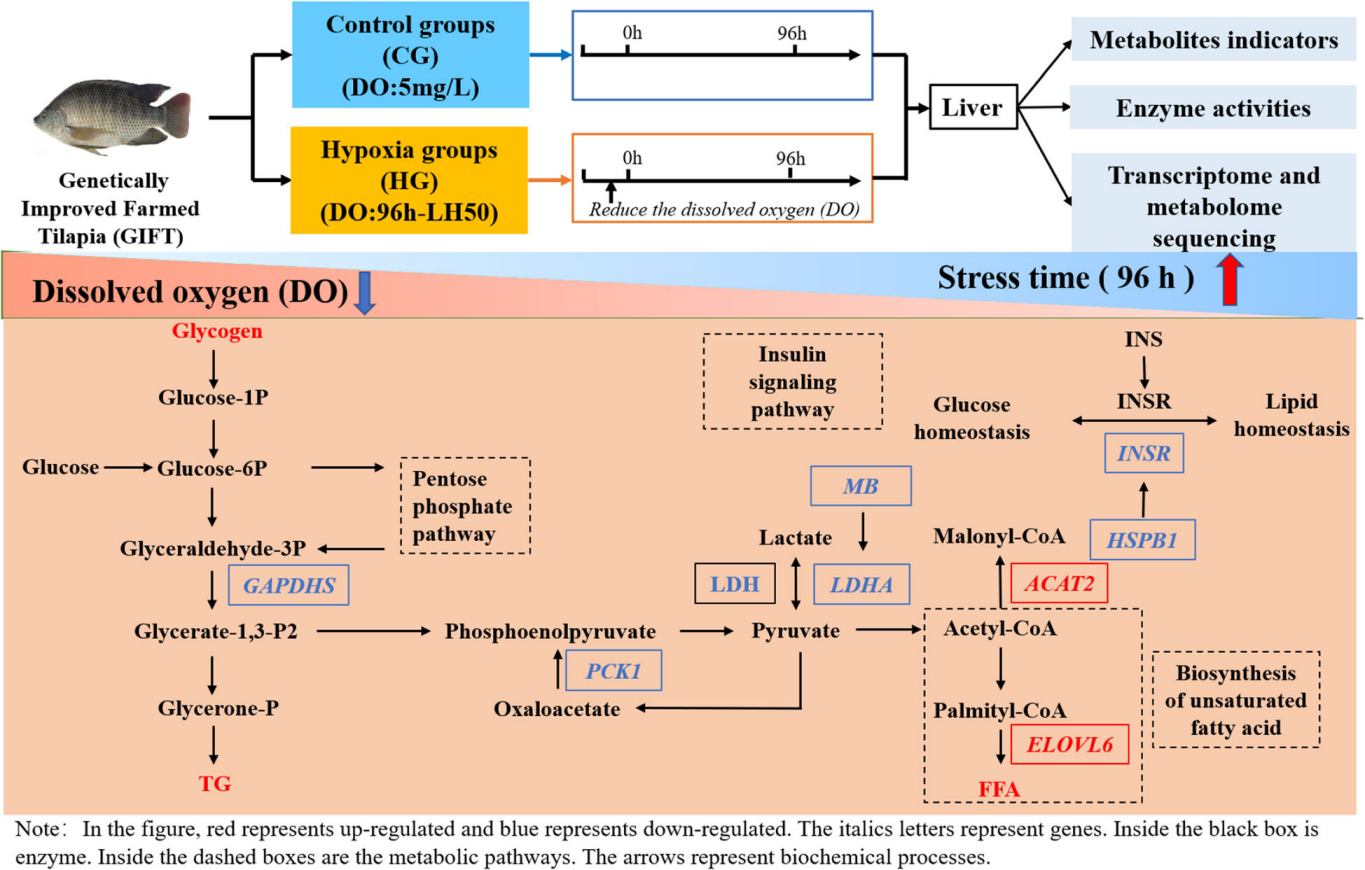
Diagram of regulated pathway in liver of GIFT exposed to acute hypoxia stress after 96 h

We reported for the first time the changes in the liver metabolism of GIFT under hypoxia stress based on combined transcriptome and metabolome analyses. We detected 2375 DE genes between the HG and CG groups, of which 581 DE genes were annotated by KEGG. We verified the transcript levels of eight DE genes by quantitative real-time PCR. We screened 15 DE metabolites related to glucose and lipid metabolism and conducted correlation analysis of DE metabolites and DE genes related to glucose and lipid metabolism. These analyses revealed that glucose metabolism and lipid metabolism change markedly in GIFT under hypoxia stress, and lipid metabolism becomes the main metabolic mode (Fig. 8). These findings provide vital information about the regulatory mechanisms of GIFT as they adapt to hypoxic conditions.

## Methods

### Experimental fish

The experimental fish were obtained from Yixing, a base of the Freshwater Fisheries Research Center, Chinese Academy of Fishery Sciences. Before the start of experiment, the fish were cultured for 15 days in an indoor water-cycling system with DO > 5 mg/L, 28±1 °C, and pH 7.6±0.2. During this period, fish were fed twice a day with feed containing 8% (w/w) crude fat and 30% (w/w) crude protein at 7:00 and 16:00. Before the start of experiment, the fish were fasted for 24 h.

### Determination of 96-h median lethal hypoxia for GIFT

We selected five low DO levels (3.2, 1.6, 0.8, 0.4, and 0.2 mg/L) to determine the 96 h median lethal hypoxia (96 h-LH50) for GIFT. The preliminary experiment was performed in 400-L plastic tanks (10 fish per tank, a total of 150 fish), and each tank was filled with 150 L aerated tap water. According to the method of Jiang et al. [62], real-time readings using a DO meter (LD0101 probe, range 0.1–20.0 mg/L, Hach, Loveland, USA) were used to detect and control the DO in water. At the beginning of the experiment, the DO in the experiment tanks reached oxygen requirement within 2 h by filling with nitrogen [34]. In the experiment, we observed the conditions of the experimental fish every hour, and caught the dead fish in time and recorded it. After the experiment, the surviving experimental fish were temporarily kept in the tank for a week, and then put back into the pond. We calculated the cumulative mortality of each treatment group within 96 h, and obtained the 96 h-LH50 of GIFT through linear interpolation.

### Treatment and sampling

A total of 240 experimental fish were used for the acute hypoxic stress experiment. The GIFT were randomly sorted into six tanks with 40 fish per tank; there were three hypoxic stress groups (HG) and three 5 mg/L control groups (CG). The DO of the three treatment groups was rapidly reduced to 96 h-LH50 using a nitrogen gas cylinder to pump nitrogen into the water [34]. The DO in water was detected by a DO meter and controlled by adjusting the oxygen intake. At the end of the 96-h acute hypoxia stress experiment, all GIFT were rapidly deep anesthetized by the following methods. Three grams Tricaine methanesulfonate (MS-222, Argent Chemical Laboratories, Redmond, WA, USA) was dissolved in 30 L aquaculture water to prepare 100 mg L^− 1^ MS-222 anesthetics in a plastic bucket. Then we soaked 10 GIFT each time with the above anesthetic for 5 min. After 5 min, fish body remained still, the abdomen facing up and the gill movement is not continuous. Then the GIFT were considered under deep anesthesia. After this, the GIFT were euthanized by rapid cooling method. The specific operation method was to soak the experimental fish in 30 L 0 °C ice water mixture (25 L of ice and 5 L of water) for 10 min until the gill cap did not open or close within 60 s. It was identified that the fish had been euthanized and then the subsequent sampling experiment was conducted [63]. Three fish were randomly taken from each tank to sample liver tissues. The liver samples were divided into two parts: one part of the liver was used for high-throughput transcriptome analysis; and the other part was used to analyze gene transcript levels by qRT-PCR. Another 13 fish were caught from each tank and their liver tissues were sampled. Ten of those samples were used for LC-MS metabolome analysis and three were used for biochemical indicator analysis. All the samples were frozen quickly in liquid nitrogen and stored at − 80 °C until subsequent experiments.

### Measurement of hepatic biochemical indicators

Liver samples (about 0.1 g) were homogenized in precooled phosphate buffer (50 mmol L^− 1^, pH 7.4) and then centrifuged for 20 min (4 °C, 3000 *g*). Test kits purchased from the Nanjing Jiancheng Bioengineering Institute (Nanjing, China) were used to measure the hepatic TC, TG, and FFA contents. Enzyme-linked immunosorbent assays were used to measure liver glycogen content and LDH activity, with kits from the Shanghai Longton Biotechnology Co., Ltd. (Shanghai, China).

### Metabolite extraction and parameter settings

The liver tissue samples (about 50 mg) were homogenized in 120 μL pre-cooled 50% methanol buffer using a high-throughput tissue lyser (Ningbo, China). The solutions were placed on ice for 10 min and then centrifuged for 20 min (4 °C, 4000 g). The supernatants were collected and used for metabolomic analysis.

All chromatographic separations were performed using an ultra-performance liquid chromatography (UPLC) system (SCIEX, Warrington, UK) equipped with an ACQUITY UPLC BEH Amide column (100 mm × 2.1 mm, 1.7 μm, Waters, Manchester, UK). The sample injection volume was 4 μl, and the flow rate was 0.4 mL/min. The solvent system consisted of solvent A (25 mM ammonium acetate + 25 mM NH_4_H_2_O) and solvent B (IPA: ACN=9:1 + 0.1% formic acid) and the elution gradient program was as follows: hold at 95% B for 0.5 min, decrease linearly from 95 to 65% B (0.5–9.5 min), decrease linearly from 65 to 40% B (9.5–10.5 min), hold at 40% B (10.5–12 min), linear increase from 40 to 95% B (12–12.2 min) and hold at 95% B until 15 min.

Mass spectrometry data acquisition was performed using a high-resolution tandem mass spectrometer TripleTOF5600plus (SCIEX). The Q-TOF was manipulated in both POS and NEG ion modes using the following parameters: curtain gas and nebulizer pressure set to 30 PSI and 60 PSI, respectively; interface heater temperature, 650 °C. In POS and NEG ion modes, the detector voltage was 5 kV and − 4.5 kV, respectively. The data were collected in IDA mode, and the mass spectra scan range was set to m/z 60 to 1200. The QC samples were prepared by mixing the supernatant mixture of all eight individual liver samples. These QC samples were injected at the beginning, middle, and end of the analytical process to ensure stability of the LC–MS analyses [64].

### Identification and quantification of metabolites

After LC−MS analysis, raw data were converted into mzXML format using ProteoWizard (version 3.0.18199). Then, XCMS software (http://xcmsonline.scripps.edu) was used for peak alignment, extraction, and quantification [65]. The peak data were further processed by the MetaX toolbox implemented with R software and CAMERA. Detectable features were first processed to remove low-quality features (detected in less than 50% of QC samples or 80% of biological samples). The remaining features with missing values were then filled using the K-nearest neighbor algorithm [66]. Before PCA, data normalization was conducted using the probabilistic quotient normalization method [67] and the quality control-base robust loess signal correction method [68]. After data normalization, features with a coefficient of variation > 30% in all QC samples were removed. The remaining features were considered high-quality and used for further analyses. We used PCA to visualize the datasets of the live samples in the HG and CG. PLSDA was then used for cluster analysis and to build a discriminant model. Permutation tests (*n* = 200) were used to evaluate the validity of PLSDA models [69].

### Analysis of DE metabolites

The DE metabolites were preliminarily screened based on variable importance in the projection (VIP) values generated by the PLSDA model in this study. The VIP cut-off value was set at 1.0 and VIP values > 1.0 were chosen as significant discriminatory features between the CG and HG. Then, these features were confirmed by Wilcoxon rank sum test [70]. The *P*-value was adjusted by Benjamini–Hochberg’s approach. The fold change (FC) of each feature between these two groups was calculated as follows: FC = mean value of normalized ion intensity obtained from CG / mean value of normalized ion intensity obtained from HG. Features with a VIP value > 1, FC > 2 or < 0.5, and an adjusted *P*-value < 0.05 were selected as DE features and used for further analyses. These criteria were selected on the basis of previous studies [71, 72].

DE features were annotated in KEGG (http://www.kegg.jp/) [73] and HMDB (http://www.hmdb.ca/) according to Tao et al. [74]. The mass data of each feature were compared with that in the database (mass tolerance, 10 ppm). We also used an in-house fragment spectrum library of metabolites to validate the metabolite identification [72].

### RNA sequencing

Total RNA was extracted from liver samples using Trizol reagent (Invitrogen, CA, USA) according to the manufacturer’s instructions. The total RNA quality and purity were analyzed using a Bioanalyzer 2100 instrument and RNA 6000 Nano Lab Chip Kit (Agilent, Palo Alto, CA, USA) for samples with RNA integrity number (RIN) > 7.0. Poly(A) mRNA was separated from approximately 10 μg total RNA with poly-T oligo-attached magnetic beads (Invitrogen, Carlsbad, CA, USA). Following purification, the mRNA was fragmented into small pieces using divalent cations under elevated temperature. According to the protocol of the mRNA Seq Sample Preparation kit (Illumina, San Diego, CA, USA), mRNA was reverse-transcribed to establish six cDNA libraries. Then, according to the vendor’s recommended protocol, paired-end sequencing (300 ± 50 bp) was performed on the Illumina Hiseq4000 platform (LC Sciences, Santiago, CA, USA) [75].

### Analysis of DE genes

The mapped data set in each sample was assembled using StringTie to reconstruct the complete transcriptome. Then, Ballgown was used to estimate the differential expression levels of genes using the complete transcriptome assembled by StringTie. The transcript levels of mRNAs and genes were estimated based on FPKM values [76]; DE genes were identified to obtain a *P*-value for each gene between any pair of samples, according to Audic et al. [77]. We calculated the false discovery rate (FDR) using the corrected *P*-value. We selected DE mRNAs and genes based on log2 (fold change) > 1 or log2 (fold change) <− 1 and corrected *P* < 0.05 using the R package, Ballgown. GO (http://geneontology.org/) and KEGG enrichment analyses [73] were performed to determine the functions of the DE genes and the metabolic pathways associated with them, respectively, according to the method of Qiang et al. [78]. The GO enrichment analysis of DE genes was carried out using tools at the Metascape database (https://metascape.org/gp/index.html#/main/step1), and a map was drawn. KEGG enrichment analysis of DE genes was performed to make scatter plots in ggplot2 (https://ggplot2.tidyverse.org/).

### Integrated analysis of metabolite and transcription profiles

On the basis of the transcriptome and metabolome results, DE metabolites and DE genes annotated in the same KEGG pathway were observed. Then, we selected the DE metabolites and top 40 DE genes (in order of |log2(fold change) | from large to small) in pathways related to glucose and lipid metabolism for subsequent analysis. Pearson’s correlation coefficient analysis was performed on the selected DE metabolites and genes using the cor program from R (version 3.5.1) [79, 80].. The correlation analysis screening conditions were |r|> 0.9 and *p* value< 0.01. The OmicStudio tools (https://www.omicstudio.cn/tool) were used for network map analysis of the screened pairs.

### Validation of DE gene in GIFT liver after 96 h hypoxic stress by qRT-PCR

We verified the expression patterns of selected DE genes associated with changes in the glycolipid metabolism pathway under hypoxia stress by qRT-PCR, with the primers shown in Table S3. Total RNA was extracted from liver tissues from the CG and HG with Trizol reagent (Invitrogen). We used PrimeScript™ RT Master Mix (Takara, Dalian, China) for the RT reaction of the mRNAs. Each 10 μL RT mixture included RNA sample (≤ 500 ng), 2 μL 5 × PrimeScript RT Master Mix, and RNase Free dH_2_O to complete the volume to 10 μL. The reaction procedure was as follows: 37 °C for 15 min, 85 °C for 5 s, and the reaction was terminated by cooling to 4 °C. We used SYBR® Premix Ex Taq kits (Takara) for analyzing DE genes using a 7900HT Fast Real-Time PCR system (Applied Biosystems, Foster City, CA, USA). Each 25-μL qRT-PCR mixture included 12.5 μL SYBR (2◊) Advantage Premix, 0.5 μL ROX Dye II (5◊), 1 μL forward primer, 1 μL reverse primer, 2 μL cDNA template, and 8 μL RNase-free dH_2_O. The PCR reaction method was that described previously by Qiang et al. [81]. The Ct values measured for each sample were normalized against the value of *β-Actin*, and the relative fold change relative to *β-Actin* expression was calculated by the 2^-ΔΔCt^ method.

### Data analysis

Data were expressed as means±standard error. Independent-sample *t* tests was used to detect differences between the CG and HG. *P* values < 0.05 were considered to indicate significance.

## Supplementary Information

**Additional file 1: Figure S1.** Total ion chromatograms of LC-MS data from profiles of GIFT hepatic metabolites detected in POS ion mode (A) and NEG ion mode (B). CL1-3_1-3 N. mzXML, HL1-3_1-3 N. mzXML, and QC1-8 N. mzXML represent samples in CG group, HG group and quality control group, respectively. X-axis represents retention time and y-axis represents total ion chromatograms in MS.

**Additional file 2: Figure S2.** m/z peak width and retention time peak width of metabolites detected in liver samples from CG or HG groups in POS and NEG modes. (A) Width of m/z peak in POS mode. (B) Width of m/z peak in NEG mode. (C) Width of retention time peak in POS mode. (D) Width of retention time peak in NEG mode.

**Additional file 3: Figure S3.** Regional distribution of reference genome alignment of valid data.

**Additional file 4: Figure S4.** Glycolysis/gluconeogenesis pathway annotated by KEGG. Red: significantly up-regulated transcript annotated to a ko node; blue: significantly down-regulated transcript annotated to a ko node; orange: transcript annotated to a ko node that is both up-regulated and down-regulated. Boxes represent genes or enzymes; open circles represent small molecule compounds; solid arrows represent the direction of biochemical reactions; dotted arrows connect other related metabolic pathways. (Same below for Fig. S5, S6, S7, S8) Reproduced with the permission of ref. 73, copyright@Kyoto Encyclopedia of Genes and Genomes (KEGG).

**Additional file 5: Figure S5.** Insulin signaling pathway annotated by KEGG. Reproduced with the permission of ref. 73, copyright@Kyoto Encyclopedia of Genes and Genomes (KEGG).

**Additional file 6: Figure S6.** Pentose phosphate pathway annotated by KEGG. Reproduced with the permission of ref. 73, copyright@Kyoto Encyclopedia of Genes and Genomes (KEGG).

**Additional file 7: FigureS7.** Biosynthesis of unsaturated fatty acids pathway annotated by KEGG. Reproduced with the permission of ref. 73, copyright@Kyoto Encyclopedia of Genes and Genomes (KEGG).

**Additional file 8: Figure S8.** Fatty acid degradation pathway annotated by KEGG**.** Reproduced with the permission of ref. 73, copyright@Kyoto Encyclopedia of Genes and Genomes (KEGG).

**Additional file 9: Table S1.** Differentially expressed genes in GIFT liver under hypoxia stress.

**Additional file 10: Table S2.** *P* values of top 20 enriched pathways under hypoxia stress.

**Additional file 11: Table S3.** Sequences of primers used to amplify differentially expressed mRNAs.

## Data Availability

The raw sequencing data generated and analyzed in this study have been deposited at the Gene Expression Omnibus (GEO) repository under the accession number (GSE146142) (https://www.ncbi.nlm.nih.gov/geo/query/acc.cgi?acc=GSE146142). The hepatic metabolome data in this study have been deposited at Dryad: 10.5061/dryad.qjq2bvqdw.

## Abbreviations

GIFT: Genetically improved farmed tilapia
DO: Dissolved oxygen
96 h-LH50: 96 h median lethal hypoxia
CG: Control group
HG: Hypoxia stress group
DE: Differentially expressed
GLU: Glucose
TC: Cholesterol
FFA: Free fatty acids
TG: Triglyceride
LDH: Lactate dehydrogenase
UPLC: Ultra-performance liquid chromatography
QC: Quality control
HMDB: Human metabolome database
KEGG: Kyoto Encyclopedia of Genes and Genomes
GO: Gene ontology
FPKM: Fragment per kilobase of exon per million fragments mapped
FC: Fold change
FDR: False discovery rate
ELOVL6: Elongation of the very long chain fatty acid protein 6
ACAT2: Acyl-coenzyme A: cholesterol acyltransferase 2
PCK1: Phosphoenolpyruvate carboxykinase 1
INSR: Insulin receptor
HSPB1: Heat shock protein family B (small) member 1
LDHA: Lactate dehydrogenase A
MB: Myoglobin
GAPDHS: Glyceraldehyde-3-phosphate dehydrogenase
PCA: Principal component analysis
PLSDA: Partial least square discriminant analysis
