# Supplementary figures and images for: Multi-omics analysis reveals the glycolipid metabolism response mechanism in the liver of genetically improved farmed Tilapia (GIFT, Oreochromis niloticus) under hypoxia stress
Source: BMC Genomics. 2021 Feb 6;22:105. doi: 10.1186/s12864-021-07410-x (PMC7866651; doi:10.1186/s12864-021-07410-x)

**A** Total Ion Chromatograms

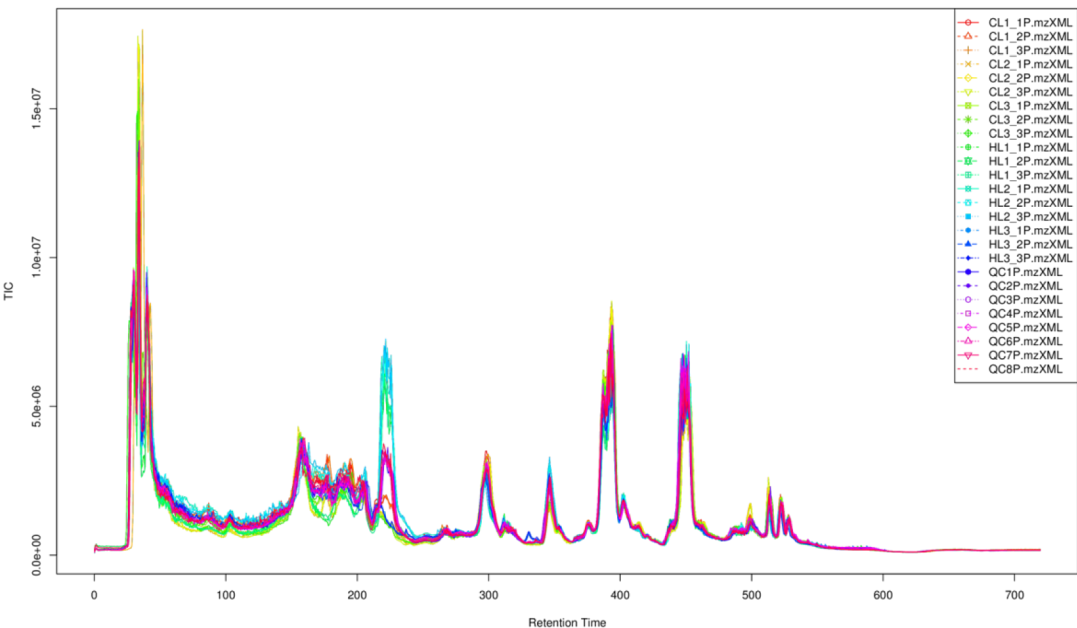

**B** Total Ion Chromatograms

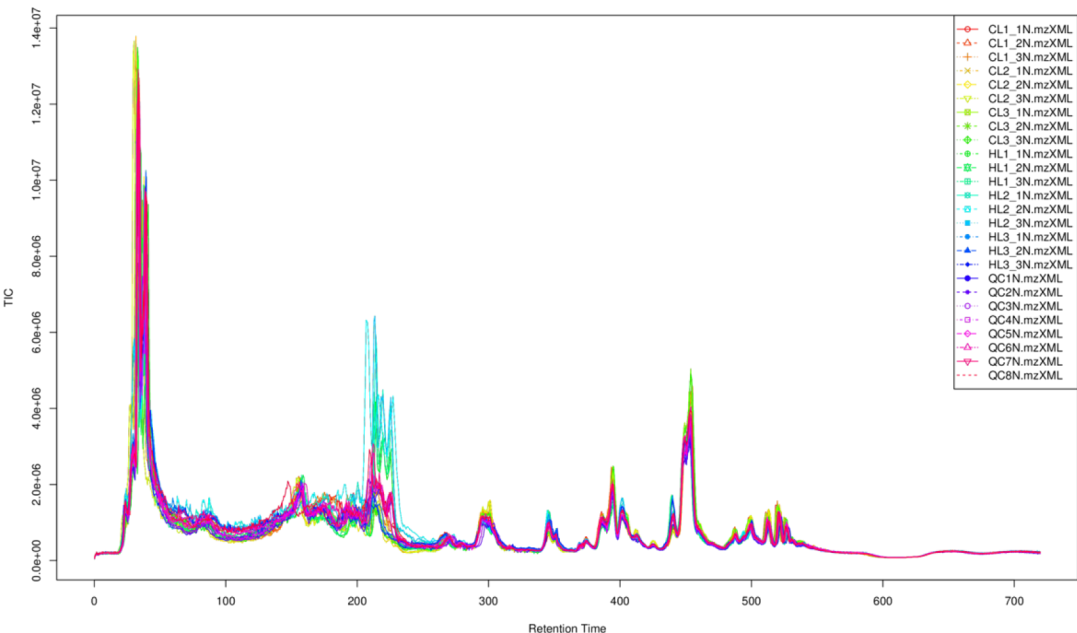

Supplement: Supplementary file 1 — Additional file 1: Figure S1. Total ion chromatograms of LC-MS data from profiles of GIFT hepatic metabolites detected in POS ion mode (A) and NEG ion mode (B). CL1-3_1-3 N. mzXML, HL1-3_1-3 N. mzXML, and QC1-8 N. mzXML represent samples in CG group, HG group and quality control group, respectively. X-axis represents retention time and y-axis represents total ion chromatograms in MS. [file 12864_2021_7410_MOESM1_ESM.pdf]

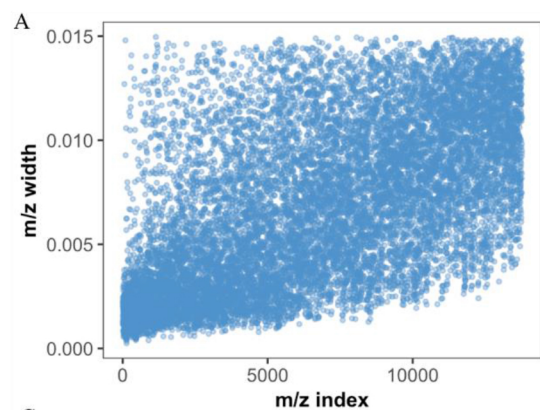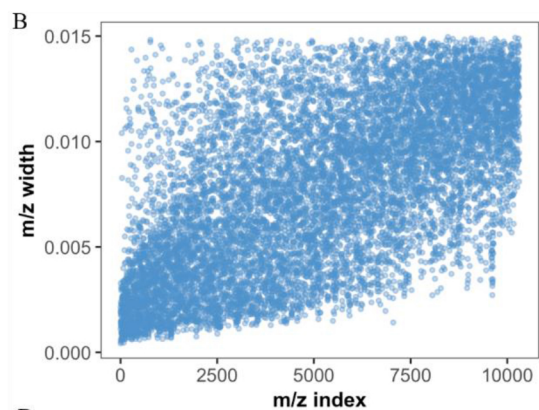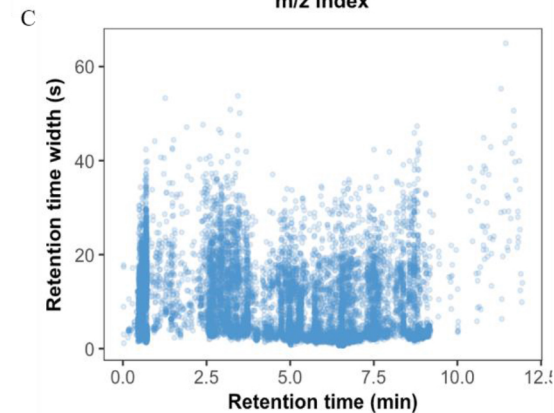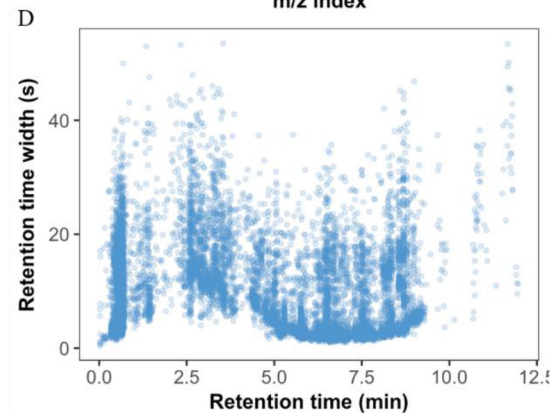

Supplement: Supplementary file 2 — Additional file 2: Figure S2. m/z peak width and retention time peak width of metabolites detected in liver samples from CG or HG groups in POS and NEG modes. (A) Width of m/z peak in POS mode. (B) Width of m/z peak in NEG mode. (C) Width of retention time peak in POS mode. (D) Width of retention time peak in NEG mode. [file 12864_2021_7410_MOESM2_ESM.pdf]

# GLYCOLYSIS / GLUCONEOGENESIS

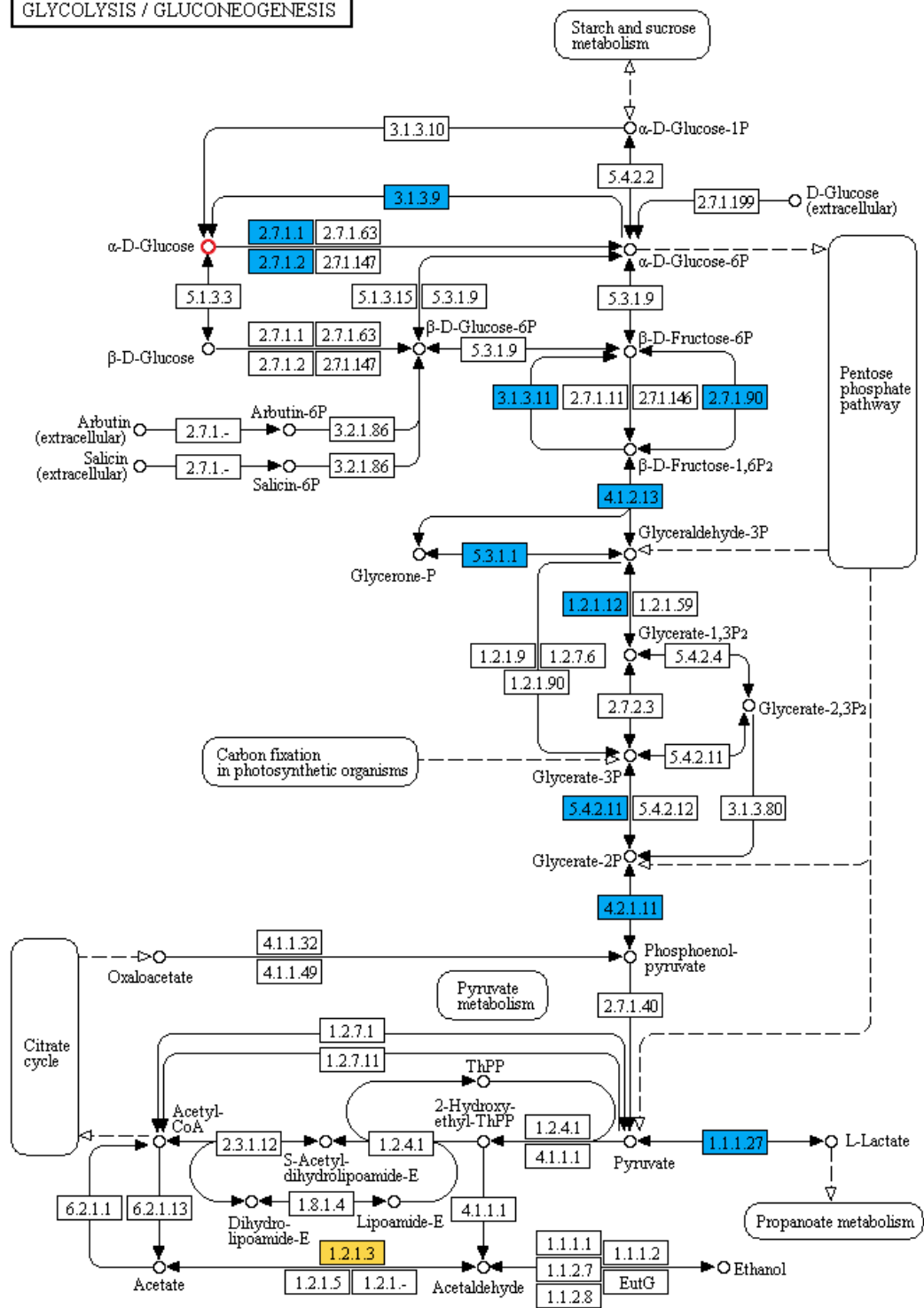

Supplement: Supplementary file 4 — Additional file 4: Figure S4. Glycolysis/gluconeogenesis pathway annotated by KEGG. Red: significantly up-regulated transcript annotated to a ko node; blue: significantly down-regulated transcript annotated to a ko node; orange: transcript annotated to a ko node that is both up-regulated and down-regulated. Boxes represent genes or enzymes; open circles represent small molecule compounds; solid arrows represent the direction of biochemical reactions; dotted arrows connect other related metabolic pathways. (Same below for Fig. S5, S6, S7, S8) Reproduced with the permission of ref. 73, copyright@Kyoto Encyclopedia of Genes and Genomes (KEGG). [file 12864_2021_7410_MOESM4_ESM.pdf]

# INSULIN SIGNALING PATHWAY

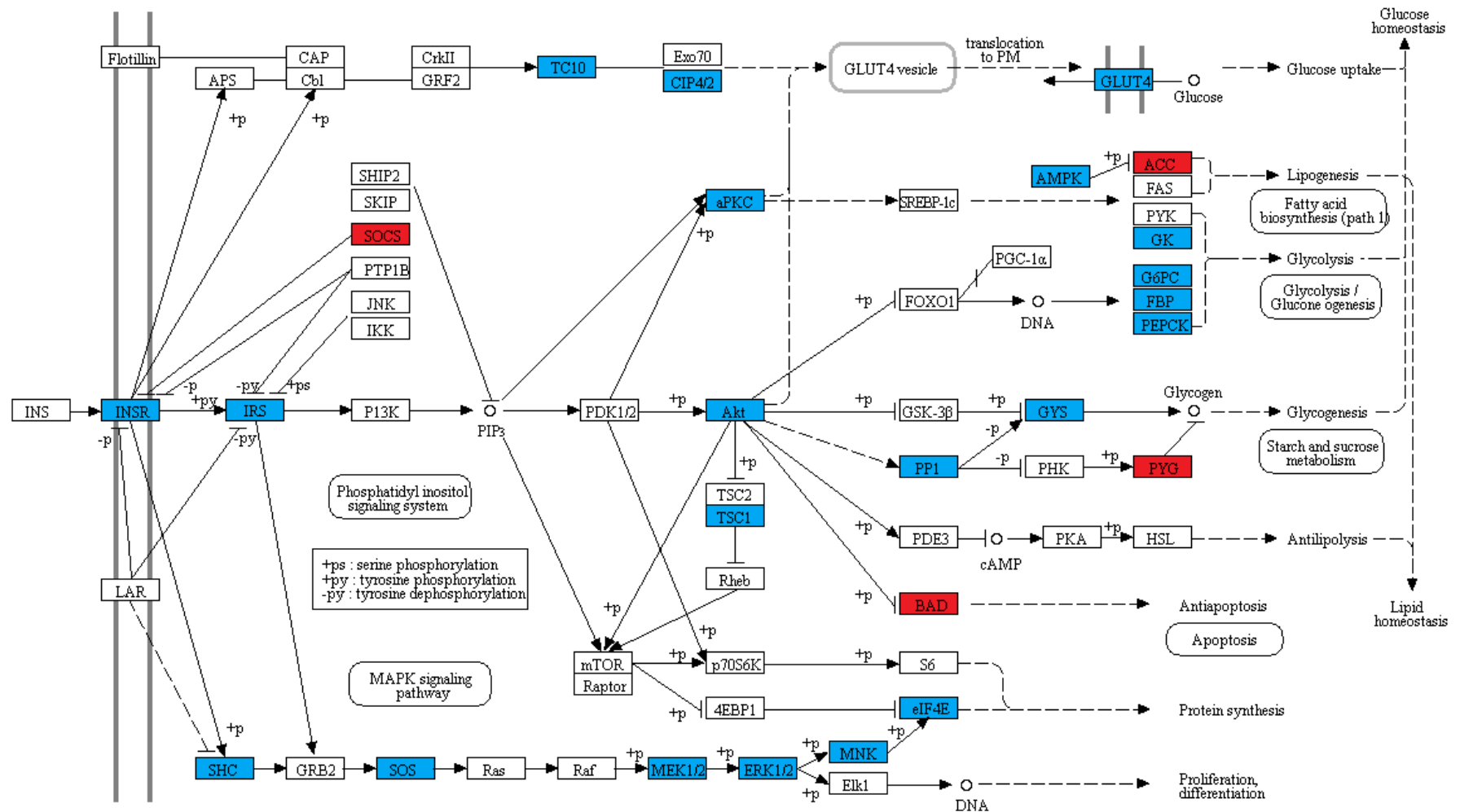

Supplement: Supplementary file 5 — Additional file 5: Figure S5. Insulin signaling pathway annotated by KEGG. Reproduced with the permission of ref. 73, copyright@Kyoto Encyclopedia of Genes and Genomes (KEGG). [file 12864_2021_7410_MOESM5_ESM.pdf]

## PENTOSE PHOSPHATE PATHWAY

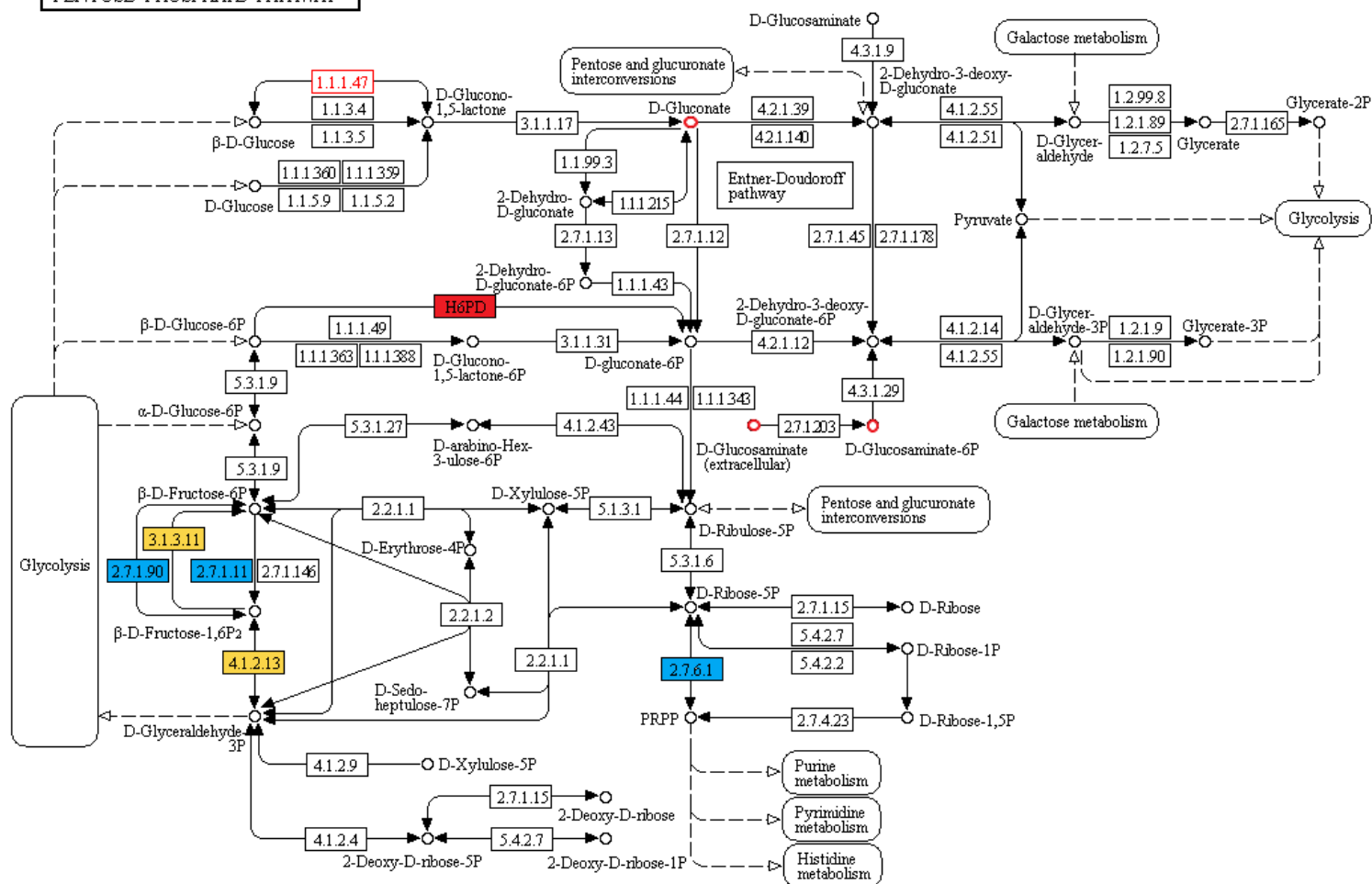

Supplement: Supplementary file 6 — Additional file 6: Figure S6. Pentose phosphate pathway annotated by KEGG. Reproduced with the permission of ref. 73, copyright@Kyoto Encyclopedia of Genes and Genomes (KEGG). [file 12864_2021_7410_MOESM6_ESM.pdf]

# BIOSYNTHESIS OF UNSATURATED FATTY ACIDS

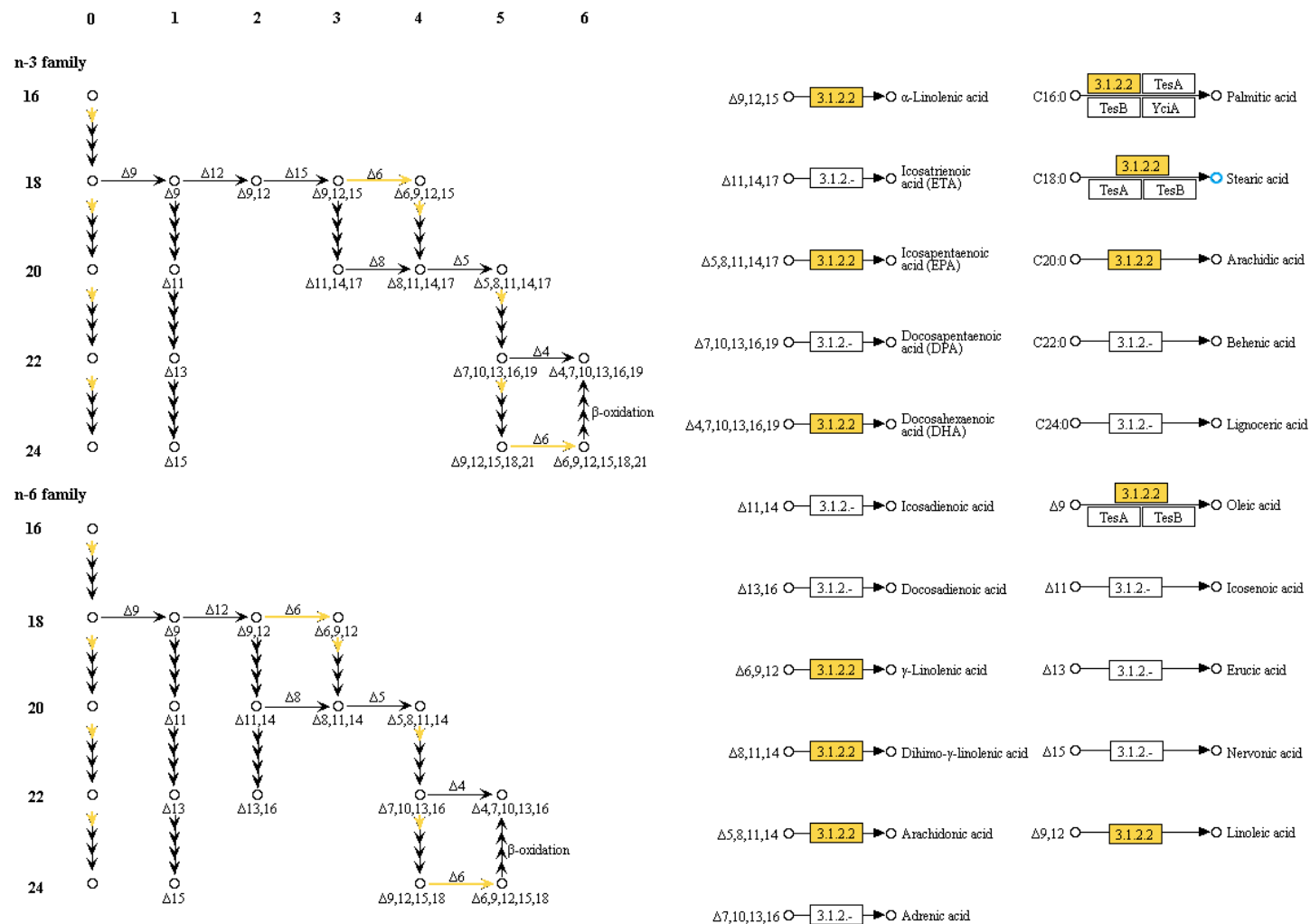

Supplement: Supplementary file 7 — Additional file 7: FigureS7. Biosynthesis of unsaturated fatty acids pathway annotated by KEGG. Reproduced with the permission of ref. 73, copyright@Kyoto Encyclopedia of Genes and Genomes (KEGG). [file 12864_2021_7410_MOESM7_ESM.pdf]

# FATTY ACID DEGRADATION

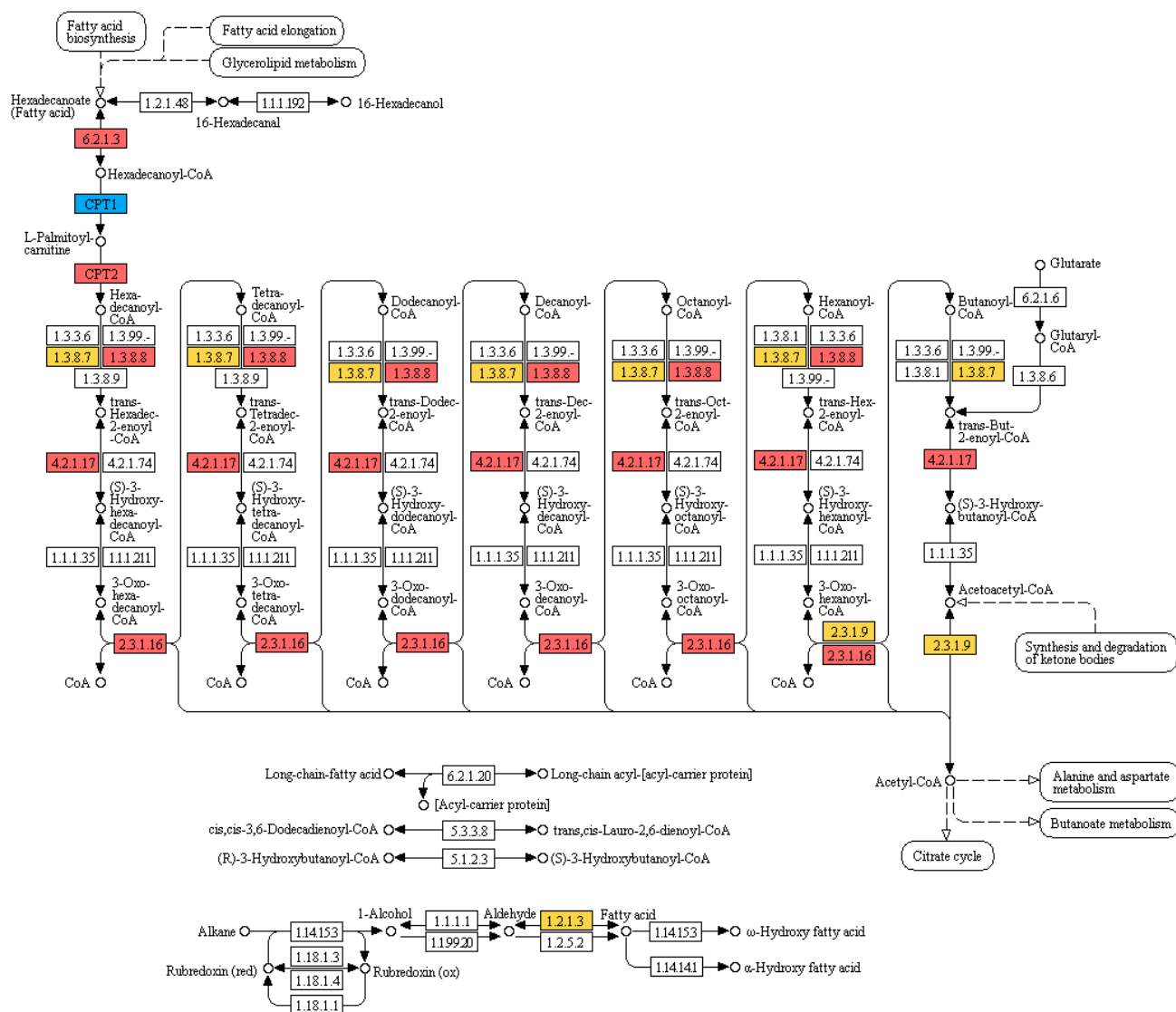

Supplement: Supplementary file 8 — Additional file 8: Figure S8. Fatty acid degradation pathway annotated by KEGG. Reproduced with the permission of ref. 73, copyright@Kyoto Encyclopedia of Genes and Genomes (KEGG). [file 12864_2021_7410_MOESM8_ESM.pdf]
