# Supplementary material for: Multi-omics analysis reveals the glycolipid metabolism response mechanism in the liver of genetically improved farmed Tilapia (GIFT, Oreochromis niloticus) under hypoxia stress
Source: BMC Genomics. 2021 Feb 6;22:105. doi: 10.1186/s12864-021-07410-x (PMC7866651; doi:10.1186/s12864-021-07410-x)
Supplement: Supplementary file 3 — Additional file 3: Figure S3. Regional distribution of reference genome alignment of valid data. [file 12864_2021_7410_MOESM3_ESM.pdf]

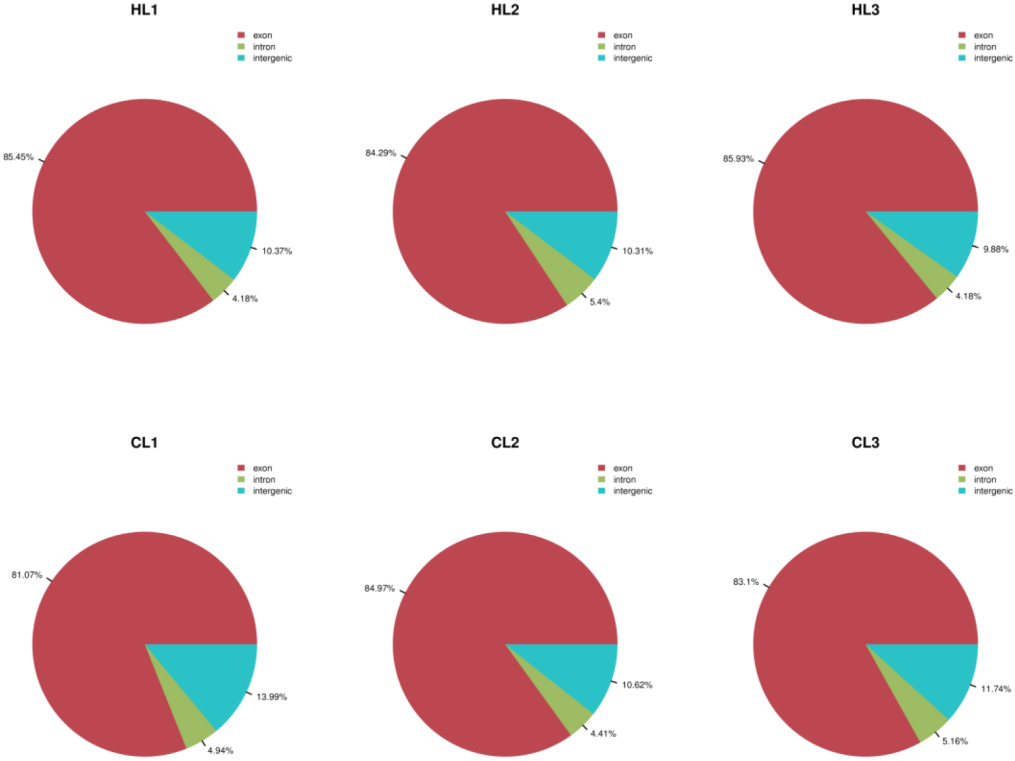

HL1
exon
intron
intergenic
85.45%
10.37%
4.18%
HL2
exon
intron
intergenic
84.29%
10.31%
5.4%
HL3
exon
intron
intergenic
85.93%
9.88%
4.18%
CL1
exon
intron
intergenic
81.07%
13.99%
4.94%
CL2
exon
intron
intergenic
84.97%
10.62%
4.41%
CL3
exon
intron
intergenic
83.1%
11.74%
5.16%
