# Supplementary material for: Multi-omics analysis reveals the glycolipid metabolism response mechanism in the liver of genetically improved farmed Tilapia (GIFT, Oreochromis niloticus) under hypoxia stress
Source: BMC Genomics. 2021 Feb 6;22:105. doi: 10.1186/s12864-021-07410-x (PMC7866651; doi:10.1186/s12864-021-07410-x)
Supplement: Supplementary file 9 — Additional file 9: Table S1. Differentially expressed genes in GIFT liver under hypoxia stress. [file 12864_2021_7410_MOESM9_ESM.docx]

Table S1 Differentially expressed genes in GIFT liver under hypoxia stress

| gene_id | gene_name | FPKM.HL1 | FPKM.HL2 | | FPKM.HL3 | | | FPKM.CL1 | | FPKM.CL2 | | FPKM.CL3 | log2(fc) | | | pval | regulation | | |
| --- | --- | --- | --- | --- | --- | --- | --- | --- | --- | --- | --- | --- | --- | --- | --- | --- | --- | --- | --- |
| MSTRG.9667 | tbxas1 | 11.02 | 16.40 | | 13.86 | | | 7.15 | | 5.38 | | 5.63 | 1.30 | | | 0.00 | up | | |
| MSTRG.1762 | TP53INP2 | 18.01 | | 21.84 | | 19.87 | 3.22 | | 2.60 | | 2.87 | | | 2.55 | 0.00 | | | up |  |
| MSTRG.14501 | ENSONIG00000015762 | 1.00 | | 0.70 | | 0.79 | 9.94 | | 10.11 | | 10.14 | | | -2.68 | 0.00 | | | down |  |
| MSTRG.23097 | ENSONIG00000007202 | 2.27 | 2.56 | | 2.69 | | | 18.39 | | 17.92 | | 17.14 | -2.37 | | | 0.00 | down | | |
| MSTRG.8763 | srsf5b | 10.09 | 8.68 | | 9.57 | | | 25.32 | | 28.09 | | 26.00 | -1.48 | | | 0.00 | down | | |
| MSTRG.6010 | flt1 | 0.95 | 1.05 | | 0.95 | | | 4.31 | | 4.51 | | 4.33 | -1.43 | | | 0.00 | down | | |
| MSTRG.17308 | ero1a | 4.19 | 4.49 | | 4.64 | | | 332.07 | | 264.01 | | 239.15 | -5.55 | | | 0.00 | down | | |
| MSTRG.3838 | tial1 | 2.42 | 2.53 | | 2.60 | | | 14.67 | | 14.80 | | 16.09 | -2.20 | | | 0.00 | down | | |
| MSTRG.17860 | ARHGEF26 | 6.31 | 8.48 | | 7.15 | | | 2.86 | | 2.44 | | 2.52 | 1.35 | | | 0.00 | up | | |
| MSTRG.25242 | dpp4 | 15.41 | 23.28 | | 19.58 | | | 4.03 | | 3.40 | | 3.25 | 2.37 | | | 0.00 | up | | |
| MSTRG.7641 | mcm7 | 15.52 | 15.79 | | 16.99 | | | 2.61 | | 2.20 | | 2.37 | 2.38 | | | 0.00 | up | | |
| MSTRG.8520 | si:ch73-266o15.4 | 0.21 | 0.27 | | 0.37 | | | 6.35 | | 5.62 | | 5.60 | -2.35 | | | 0.00 | down | | |
| MSTRG.4282 | ENSONIG00000005539 | 1.41 | 1.76 | | 1.89 | | | 15.26 | | 12.98 | | 14.14 | -2.38 | | | 0.00 | down | | |
| MSTRG.951 | p4ha1b | 0.39 | 0.34 | | 0.43 | | | 29.73 | | 40.51 | | 31.82 | -4.74 | | | 0.00 | down | | |
| MSTRG.14744 | ddit4 | 0.54 | 0.30 | | 0.02 | | | 589.49 | | 491.93 | | 675.64 | -8.90 | | | 0.00 | down | | |
| MSTRG.14619 | arpc3 (1 of many) | 0 | 0 | | 0 | | | 13.31 | | 10.46 | | 10.57 | -3.55 | | | 0.00 | down | | |
| MSTRG.1130 | adprm | 5.82 | 6.54 | | 7.35 | | | 0 | | 0 | | 0 | 2.97 | | | 0.00 | up | | |
| MSTRG.5904 | tdo2b | 261.27 | 289.49 | | 259.81 | | | 23.94 | | 25.31 | | 28.37 | 3.35 | | | 0.00 | up | | |
| MSTRG.14789 | ptn | 15.05 | 11.03 | | 13.33 | | | 2.12 | | 2.79 | | 2.94 | 1.76 | | | 0.00 | up | | |
| MSTRG.3052 | ENSONIG00000014877 | 218.17 | 334.41 | | 273.66 | | | 48.22 | | 40.36 | | 47.59 | 2.79 | | | 0.00 | up | | |
| MSTRG.7353 | ENSONIG00000002165 | 0.78 | 1.04 | | 0.83 | | | 5.07 | | 4.16 | | 4.68 | -1.48 | | | 0.00 | down | | |
| MSTRG.9160 | filip1l | 0.75 | 0.99 | | 0.91 | | | 3.68 | | 2.93 | | 3.25 | -1.07 | | | 0.00 | down | | |
| MSTRG.20805 | hsd17b10 | 15.44 | 13.73 | | 15.40 | | | 1.87 | | 2.37 | | 1.84 | 2.31 | | | 0.00 | up | | |
| MSTRG.25054 | ptmaa | 491.55 | 675.13 | | 623.87 | | | 210.82 | | 190.92 | | 197.24 | 1.74 | | | 0.00 | up | | |
| MSTRG.20329 | cyp7a1 | 70.21 | 109.69 | | 90.54 | | | 5.30 | | 2.26 | | 2.46 | 4.86 | | | 0.00 | up | | |
| MSTRG.6304 | ST3GAL1 (1 of many) | 6.76 | 10.22 | | 9.03 | | | 1.11 | | 1.01 | | 0.95 | 2.43 | | | 0.00 | up | | |
| MSTRG.10696 | th2 | 1.53 | 3.91 | | 3.08 | | | 0.63 | | 0.08 | | 0.09 | 2.02 | | | 0.00 | up | | |
| MSTRG.6790 | scly | 6.83 | 7.57 | | 8.24 | | | 50.61 | | 53.66 | | 47.67 | -2.56 | | | 0.00 | down | | |
| MSTRG.423 | pkp3b | 4.80 | 5.03 | | 5.36 | | | 44.80 | | 42.14 | | 36.10 | -2.73 | | | 0.00 | down | | |
| MSTRG.1731 | slc17a9b | 2.38 | 3.06 | | 2.65 | | | 0.78 | | 0.75 | | 0.73 | 1.16 | | | 0.00 | up | | |
| MSTRG.5335 | fuca2 | 15.15 | 17.22 | | 16.99 | | | 5.54 | | 5.27 | | 5.87 | 1.47 | | | 0.00 | up | | |
| MSTRG.9735 | TPMT (1 of many) | 24.31 | 21.70 | | 23.23 | | | 0.95 | | 1.48 | | 0.85 | 3.43 | | | 0.00 | up | | |
| MSTRG.11980 | TMEM25 | 3.03 | 3.17 | | 3.47 | | | 0.90 | | 0.83 | | 0.87 | 1.21 | | | 0.00 | up | | |
| MSTRG.6787 | agxta;dtymk | 103.54 | 79.53 | | 78.04 | | | 14.46 | | 16.41 | | 14.93 | 2.27 | | | 0.00 | up | | |
| MSTRG.1446 | camk1gb | 0.05 | 0 | | 0.04 | | | 4.79 | | 4.06 | | 5.09 | -2.45 | | | 0.00 | down | | |
| MSTRG.10053 | myo9ab | 0.17 | 0.14 | | 0.22 | | | 2.47 | | 2.30 | | 2.13 | -1.47 | | | 0.00 | down | | |
| MSTRG.10301 | pank4 | 1.71 | 2.53 | | 2.13 | | | 10.31 | | 7.65 | | 9.21 | -1.51 | | | 0.00 | down | | |
| MSTRG.2272 | il6st | 9.20 | 13.36 | | 10.63 | | | 48.52 | | 34.55 | | 40.29 | -1.55 | | | 0.00 | down | | |
| MSTRG.25809 | polk | 2.01 | 1.58 | | 1.89 | | | 9.62 | | 13.43 | | 11.29 | -2.29 | | | 0.00 | down | | |
| MSTRG.7037 | rusc2 | 2.88 | 3.12 | | 3.25 | | | 8.15 | | 7.23 | | 7.63 | -1.02 | | | 0.00 | down | | |
| MSTRG.10471 | Mb | 73.89 | 40.00 | | 63.90 | | | 2018.34 | | 2834.12 | | 1859.50 | -5.57 | | | 0.00 | down | | |
| MSTRG.14842 | nr0b2 | 1.07 | 2.29 | | 1.84 | | | 78.36 | | 86.31 | | 67.37 | -4.66 | | | 0.00 | down | | |
| MSTRG.15230 | cratb | 1.74 | 2.21 | | 1.71 | | | 25.87 | | 19.94 | | 18.82 | -2.80 | | | 0.00 | down | | |
| MSTRG.23740 | ENSONIG00000020393 | 1204.79 | 978.50 | | 1000.45 | | | 305.93 | | 390.52 | | 374.58 | 1.39 | | | 0.00 | up | | |
| MSTRG.11775 | ENSONIG00000012747 | 49.80 | 63.79 | | 68.21 | | | 12.58 | | 9.07 | | 10.27 | 2.61 | | | 0.00 | up | | |
| MSTRG.7606 | ptgs2b | 0.70 | 0.97 | | 1.05 | | | 10.05 | | 9.06 | | 7.98 | -2.28 | | | 0.00 | down | | |
| MSTRG.9298 | sod1 | 138.15 | 152.88 | | 148.49 | | | 27.77 | | 32.75 | | 30.38 | 2.23 | | | 0.00 | up | | |
| MSTRG.5325 | map4k5 | 6.13 | 8.12 | | 7.46 | | | 3.30 | | 3.03 | | 2.95 | 1.14 | | | 0.00 | up | | |
| MSTRG.11984 | MPZL2 | 1.10 | 1.50 | | 1.34 | | | 10.36 | | 8.12 | | 7.66 | -1.90 | | | 0.00 | down | | |
| MSTRG.6840 | ggcx | 11.51 | 7.97 | | 9.80 | | | 27.89 | | 35.59 | | 29.36 | -1.79 | | | 0.00 | down | | |
| MSTRG.15147 | riok3 | 9.61 | 7.84 | | 8.59 | | | 29.56 | | 30.46 | | 27.42 | -1.72 | | | 0.00 | down | | |
| MSTRG.12857 | gbe1b | 24.01 | 22.04 | | 24.83 | | | 6.40 | | 7.32 | | 6.37 | 1.62 | | | 0.00 | up | | |
| MSTRG.19539 | gusb (1 of many) | 11.74 | 11.26 | | 10.79 | | | 4.50 | | 5.07 | | 4.98 | 1.02 | | | 0.00 | up | | |
| MSTRG.8519 | si:ch73-266o15.4 | 0.11 | 0.04 | | 0 | | | 2.07 | | 2.22 | | 2.56 | -1.70 | | | 0.00 | down | | |
| MSTRG.2432 | si:dkey-42p8.3 | 0.73 | 0.67 | | 0.49 | | | 50.06 | | 38.84 | | 33.08 | -4.58 | | | 0.00 | down | | |
| MSTRG.1930 | tfr1b | 4.20 | 2.94 | | 3.07 | | | 18.05 | | 17.91 | | 17.82 | -2.24 | | | 0.00 | down | | |
| MSTRG.19515 | zgc:63645 | 4.93 | 5.73 | | 5.13 | | | 21.12 | | 23.78 | | 23.08 | -1.90 | | | 0.00 | down | | |
| MSTRG.23580 | ENSONIG00000004827 | 4.74 | 6.98 | | 7.07 | | | 54.03 | | 40.03 | | 49.28 | -2.52 | | | 0.00 | down | | |
| MSTRG.3412 | pla2g7 | 6.04 | 6.62 | | 6.90 | | | 2.37 | | 2.25 | | 2.03 | 1.28 | | | 0.00 | up | | |
| MSTRG.25622 | SOS1 | 6.75 | 4.49 | | 5.17 | | | 11.03 | | 12.97 | | 11.78 | -1.21 | | | 0.00 | down | | |
| MSTRG.4968 | nupr1 | 89.24 | 98.77 | | 103.93 | | | 1893.88 | | 1193.15 | | 1332.05 | -3.68 | | | 0.00 | down | | |
| MSTRG.13500 | egln2 | 8.37 | 5.25 | | 8.30 | | | 55.98 | | 89.04 | | 87.87 | -3.57 | | | 0.00 | down | | |
| MSTRG.22492 | mapk6 | 6.80 | 7.09 | | 7.00 | | | 51.01 | | 51.12 | | 40.89 | -2.57 | | | 0.00 | down | | |
| MSTRG.24936 | ngdn | 14.64 | 10.55 | | 14.26 | | | 48.79 | | 56.96 | | 61.48 | -2.20 | | | 0.00 | down | | |
| MSTRG.6600 | ENSONIG00000002492 | 8.68 | 9.44 | | 7.29 | | | 0.36 | | 0.04 | | 0.11 | 3.13 | | | 0.00 | up | | |
| MSTRG.16838 | ENSONIG00000021121 | 0.52 | 0 | | 0.30 | | | 1.75 | | 2.71 | | 2.88 | -1.73 | | | 0.00 | down | | |
| MSTRG.3141 | si:dkey-37f18.2 | 0.04 | 0.10 | | 0.08 | | | 1.63 | | 1.32 | | 1.27 | -1.09 | | | 0.00 | down | | |
| MSTRG.16884 | gys1 | 1.35 | 1.56 | | 1.31 | | | 56.24 | | 34.41 | | 37.00 | -3.95 | | | 0.00 | down | | |
| MSTRG.7406 | ENSONIG00000022105 | 1.54 | 0.47 | | 1.15 | | | 5.79 | | 7.93 | | 8.65 | -2.41 | | | 0.00 | down | | |
| MSTRG.19388 | cyp4f3 (1 of many) | 5.15 | 4.73 | | 5.19 | | | 0.06 | | 0.09 | | 0.32 | 2.33 | | | 0.00 | up | | |
| MSTRG.5962 | abca1b | 0.70 | 0.93 | | 1.30 | | | 15.94 | | 16.20 | | 14.43 | -3.00 | | | 0.00 | down | | |
| MSTRG.6388 | sqrdl | 11.82 | 10.36 | | 8.86 | | | 79.11 | | 78.63 | | 93.93 | -2.98 | | | 0.00 | down | | |
| MSTRG.5084 | ifi35 | 17.63 | 29.76 | | 28.41 | | | 6.06 | | 4.50 | | 5.22 | 2.35 | | | 0.00 | up | | |
| MSTRG.10418 | ENSONIG00000012347 | 245.21 | 320.68 | | 285.88 | | | 24.10 | | 29.91 | | 23.55 | 3.48 | | | 0.00 | up | | |
| MSTRG.13905 | egln3 | 2.61 | 1.81 | | 1.86 | | | 375.07 | | 212.12 | | 239.07 | -6.37 | | | 0.00 | down | | |
| MSTRG.17165 | DLC1 | 3.19 | 4.75 | | 4.09 | | | 25.12 | | 20.95 | | 18.16 | -1.94 | | | 0.00 | down | | |
| MSTRG.17109 | tesca | 0.92 | 0.97 | | 0.76 | | | 9.78 | | 7.64 | | 7.77 | -2.23 | | | 0.00 | down | | |
| MSTRG.7452 | tspan11 | 12.00 | 18.92 | | 12.52 | | | 3.03 | | 1.97 | | 2.42 | 2.41 | | | 0.00 | up | | |
| MSTRG.26981 | tecpr2 | 4.30 | 4.29 | | 4.69 | | | 35.63 | | 30.36 | | 26.91 | -2.48 | | | 0.00 | down | | |
| MSTRG.21286 | slc22a13 (1 of many) | 2.20 | 1.83 | | 1.65 | | | 0 | | 0.07 | | 0 | 1.42 | | | 0.00 | up | | |
| MSTRG.15922 | cipca | 3.93 | 4.68 | | 4.40 | | | 41.24 | | 26.97 | | 32.71 | -2.49 | | | 0.00 | down | | |
| MSTRG.13196 | stk40 | 2.38 | 4.19 | | 3.55 | | | 12.07 | | 8.69 | | 8.65 | -1.01 | | | 0.00 | down | | |
| MSTRG.18332 | ENSONIG00000012894 | 0.83 | 1.15 | | 0.91 | | | 0 | | 0 | | 0 | 1.04 | | | 0.00 | up | | |
| MSTRG.1867 | si:dkey-10f23.2 | 4.01 | 3.39 | | 4.49 | | | 20.01 | | 21.60 | | 18.97 | -2.16 | | | 0.00 | down | | |
| MSTRG.14136 | fnip1 | 1.35 | 2.01 | | 1.50 | | | 14.32 | | 9.44 | | 10.36 | -2.00 | | | 0.00 | down | | |
| MSTRG.861 | tbl1xr1b | 9.18 | 7.23 | | 8.43 | | | 2.04 | | 2.63 | | 2.07 | 1.38 | | | 0.00 | up | | |
| MSTRG.3044 | mafb | 5.33 | 3.37 | | 4.79 | | | 12.62 | | 20.00 | | 16.78 | -1.96 | | | 0.00 | down | | |
| MSTRG.2365 | tor3a | 7.58 | 5.15 | | 7.82 | | | 67.52 | | 82.67 | | 62.10 | -3.38 | | | 0.00 | down | | |
| MSTRG.5532 | nfil3-2 | 0.27 | 0.45 | | 0.55 | | | 12.91 | | 10.07 | | 14.45 | -3.12 | | | 0.00 | down | | |
| MSTRG.1316 | slc16a6a | 19.13 | 42.70 | | 24.27 | | | 4.16 | | 1.38 | | 2.64 | 3.55 | | | 0.00 | up | | |
| MSTRG.14507 | ctsf | 302.51 | 256.16 | | 251.26 | | | 70.24 | | 66.70 | | 62.13 | 1.95 | | | 0.00 | up | | |
| MSTRG.19586 | tgm1l1 | 3.53 | 3.25 | | 3.52 | | | 9.06 | | 8.30 | | 9.34 | -1.17 | | | 0.00 | down | | |
| MSTRG.6824 | bmp1b | 5.31 | 7.18 | | 4.71 | | | 0.11 | | 0.08 | | 0.09 | 2.78 | | | 0.00 | up | | |
| MSTRG.22615 | rpgrip1l | 0.49 | 0.67 | | 0.77 | | | 4.00 | | 3.75 | | 3.38 | -1.44 | | | 0.00 | down | | |
| MSTRG.11455 | sass6 | 7.59 | 8.85 | | 7.60 | | | 1.82 | | 1.39 | | 1.11 | 2.02 | | | 0.00 | up | | |
| MSTRG.8736 | HADHB | 51.54 | 65.26 | | 51.08 | | | 9.74 | | 9.33 | | 11.24 | 2.45 | | | 0.00 | up | | |
| MSTRG.8943 | cpt1cb | 0.36 | 0.27 | | 0.13 | | | 3.85 | | 3.64 | | 4.34 | -2.01 | | | 0.00 | down | | |
| MSTRG.18641 | tbl1x | 0.71 | 0.13 | | 0.34 | | | 1.24 | | 1.85 | | 1.44 | -1.12 | | | 0.00 | down | | |
| MSTRG.14306 | bcor (1 of many) | 0.62 | 0.51 | | 0.54 | | | 15.34 | | 10.42 | | 13.94 | -3.11 | | | 0.00 | down | | |
| MSTRG.16353 | bckdhb | 28.17 | 30.46 | | 27.89 | | | 8.84 | | 10.35 | | 9.24 | 1.50 | | | 0.00 | up | | |
| MSTRG.18124 | eno4 | 0.94 | 1.50 | | 1.23 | | | 0 | | 0 | | 0 | 1.26 | | | 0.00 | up | | |
| MSTRG.588 | SIRT5 (1 of many) | 6.69 | 6.29 | | 7.97 | | | 139.00 | | 89.96 | | 108.02 | -3.69 | | | 0.00 | down | | |
| MSTRG.17216 | plod1a | 1.69 | 2.21 | | 1.42 | | | 35.17 | | 25.55 | | 23.14 | -3.18 | | | 0.00 | down | | |
| MSTRG.10918 | si:ch211-117c9.5 | 0 | 0.05 | | 0.04 | | | 1.15 | | 1.40 | | 1.42 | -1.19 | | | 0.00 | down | | |
| MSTRG.17991 | eaf2 | 6.37 | 5.73 | | 5.02 | | | 0.60 | | 0.59 | | 0.82 | 1.95 | | | 0.00 | up | | |
| MSTRG.20338 | sdr16c5b | 61.53 | 70.19 | | 58.15 | | | 24.68 | | 24.65 | | 24.53 | 1.38 | | | 0.00 | up | | |
| MSTRG.9522 | f9b | 68.90 | 85.23 | | 72.78 | | | 380.84 | | 259.16 | | 295.94 | -1.80 | | | 0.00 | down | | |
| MSTRG.1337 | cox6b1 | 99.15 | 122.65 | | 115.28 | | | 458.84 | | 486.12 | | 541.08 | -2.07 | | | 0.00 | down | | |
| MSTRG.10387 | ENSONIG00000012251 | 58.49 | 115.96 | | 104.85 | | | 2.84 | | 0.73 | | 2.62 | 5.48 | | | 0.00 | up | | |
| MSTRG.11290 | rnf213b | 9.09 | 13.89 | | 14.60 | | | 0.75 | | 0.58 | | 0.94 | 3.12 | | | 0.00 | up | | |
| MSTRG.4417 | tcf7l2 | 1.48 | 1.92 | | 1.80 | | | 6.22 | | 6.41 | | 5.58 | -1.30 | | | 0.00 | down | | |
| MSTRG.4741 | atmin | 1.06 | 0.86 | | 1.12 | | | 3.46 | | 3.32 | | 3.24 | -1.14 | | | 0.00 | down | | |
| MSTRG.7525 | AKR1D1 (1 of many) | 5.88 | 5.96 | | 5.74 | | | 1.03 | | 1.49 | | 1.13 | 1.59 | | | 0.00 | up | | |
| MSTRG.2142 | si:ch73-190m4.1 | 1.29 | 2.65 | | 2.09 | | | 0.20 | | 0.09 | | 0.10 | 1.63 | | | 0.00 | up | | |
| MSTRG.16006 | col15a1b | 1.83 | 1.99 | | 2.07 | | | 0.19 | | 0.33 | | 0.31 | 1.21 | | | 0.00 | up | | |
| MSTRG.17843 | ENSONIG00000007859 | 1.55 | 1.51 | | 1.60 | | | 7.24 | | 8.65 | | 9.86 | -1.98 | | | 0.00 | down | | |
| MSTRG.6994 | sh2d3cb | 7.85 | 11.43 | | 9.54 | | | 4.41 | | 4.12 | | 3.97 | 1.20 | | | 0.00 | up | | |
| MSTRG.9601 | tgm2l (1 of many) | 4.92 | 4.22 | | 5.85 | | | 34.87 | | 30.02 | | 34.08 | -2.52 | | | 0.00 | down | | |
| MSTRG.10308 | alpl | 4.15 | 3.09 | | 4.29 | | | 10.96 | | 12.37 | | 11.61 | -1.52 | | | 0.00 | down | | |
| MSTRG.25317 | ENSONIG00000000234 | 5.68 | 8.17 | | 9.12 | | | 1.44 | | 1.00 | | 1.14 | 2.18 | | | 0.00 | up | | |
| MSTRG.23990 | ENSONIG00000003847 | 113.02 | 58.45 | | 79.98 | | | 4.39 | | 9.83 | | 5.66 | 3.00 | | | 0.00 | up | | |
| MSTRG.23344 | zgc:92040 | 17.90 | 13.58 | | 15.64 | | | 1.24 | | 2.47 | | 2.47 | 2.20 | | | 0.00 | up | | |
| MSTRG.8640 | nod1 | 0.45 | 0.93 | | 0.88 | | | 5.57 | | 3.60 | | 4.00 | -1.37 | | | 0.00 | down | | |
| MSTRG.278 | cyp1a | 25.86 | 32.97 | | 21.35 | | | 3.29 | | 2.87 | | 2.87 | 2.90 | | | 0.00 | up | | |
| MSTRG.17346 | ENSONIG00000002744 | 3.64 | 4.66 | | 3.95 | | | 1.32 | | 1.40 | | 1.38 | 1.17 | | | 0.00 | up | | |
| MSTRG.3814 | HABP2;ALDH18A1 (1 of many) | 400.56 | 393.59 | | 351.40 | | | 81.53 | | 105.47 | | 89.29 | 1.96 | | | 0.00 | up | | |
| MSTRG.3660 | fuom | 4.39 | 5.06 | | 5.27 | | | 0.52 | | 0.80 | | 0.69 | 1.82 | | | 0.00 | up | | |
| MSTRG.5173 | bhlhe40 | 0.23 | 0.60 | | 0.53 | | | 6.02 | | 6.03 | | 4.71 | -2.04 | | | 0.00 | down | | |
| MSTRG.999 | abcg8 | 7.51 | 12.39 | | 10.59 | | | 0.79 | | 0.70 | | 1.09 | 2.77 | | | 0.00 | up | | |
| MSTRG.5180 | comtb | 94.00 | 107.28 | | 95.88 | | | 31.83 | | 36.36 | | 31.89 | 1.57 | | | 0.00 | up | | |
| MSTRG.7871 | ENSONIG00000019489 | 389.72 | 303.69 | | 332.90 | | | 76.88 | | 111.29 | | 86.66 | 1.68 | | | 0.00 | up | | |
| MSTRG.20656 | pard6b | 2.46 | 4.80 | | 3.20 | | | 15.53 | | 9.47 | | 10.77 | -1.16 | | | 0.00 | down | | |
| MSTRG.3112 | fkbp16 | 0.11 | 0.22 | | 0.28 | | | 1.63 | | 1.77 | | 1.65 | -1.13 | | | 0.00 | down | | |
| MSTRG.16091 | rsad2 | 60.67 | 89.26 | | 138.56 | | | 2.18 | | 1.28 | | 1.12 | 5.53 | | | 0.00 | up | | |
| MSTRG.8655 | ZDHHC14 (1 of many) | 3.44 | 1.93 | | 2.38 | | | 5.37 | | 6.25 | | 6.82 | -1.25 | | | 0.00 | down | | |
| MSTRG.7401 | slc2a5 | 4.39 | 3.40 | | 4.08 | | | 10.34 | | 11.27 | | 13.17 | -1.47 | | | 0.00 | down | | |
| MSTRG.7371 | ppp1r3da | 0 | 0.12 | | 0.10 | | | 5.89 | | 8.98 | | 6.75 | -2.97 | | | 0.00 | down | | |
| MSTRG.9600 | tgm2l (1 of many) | 10.94 | 10.17 | | 13.83 | | | 94.07 | | 80.25 | | 77.48 | -2.71 | | | 0.00 | down | | |
| MSTRG.9900 | RBMS1 (1 of many) | 0.15 | 0.08 | | 0.08 | | | 2.18 | | 2.00 | | 2.65 | -1.59 | | | 0.00 | down | | |
| MSTRG.14533 | ENSONIG00000015804 | 2.48 | 2.98 | | 2.25 | | | 11.33 | | 11.98 | | 13.12 | -1.86 | | | 0.00 | down | | |
| MSTRG.15113 | SYDE2 | 1.81 | 3.43 | | 1.45 | | | 81.20 | | 58.30 | | 85.98 | -4.33 | | | 0.00 | down | | |
| MSTRG.536 | cyp8b2 | 101.31 | 107.43 | | 120.12 | | | 16.86 | | 20.90 | | 15.47 | 2.56 | | | 0.00 | up | | |
| MSTRG.4548 | ENSONIG00000001101 | 14.85 | 22.68 | | 28.01 | | | 2.00 | | 0.73 | | 0.82 | 3.77 | | | 0.00 | up | | |
| MSTRG.23514 | ENSONIG00000018718 | 2.71 | 7.44 | | 4.81 | | | 0.52 | | 0.29 | | 0.30 | 2.48 | | | 0.00 | up | | |
| MSTRG.714 | cirbpa | 258.01 | 211.49 | | 224.01 | | | 429.55 | | 446.37 | | 499.51 | -1.10 | | | 0.00 | down | | |
| MSTRG.14424 | isg15 | 87.90 | 106.95 | | 162.75 | | | 1.84 | | 0.50 | | 1.67 | 5.97 | | | 0.00 | up | | |
| MSTRG.20005 | ENSONIG00000011675 | 2.22 | 3.02 | | 3.19 | | | 0.75 | | 0.64 | | 0.62 | 1.31 | | | 0.00 | up | | |
| MSTRG.17160 | ugt8 | 5.28 | 6.01 | | 4.86 | | | 1.07 | | 1.30 | | 1.13 | 1.57 | | | 0.00 | up | | |
| MSTRG.23607 | ENSONIG00000016482 | 0.90 | 1.25 | | 0.90 | | | 0 | | 0 | | 0 | 1.08 | | | 0.00 | up | | |
| MSTRG.18339 | atp10b | 0.69 | 1.04 | | 1.09 | | | 12.67 | | 8.87 | | 13.12 | -2.52 | | | 0.00 | down | | |
| MSTRG.21643 | si:ch211-236d3.4 | 9.48 | 12.27 | | 10.32 | | | 72.43 | | 97.43 | | 78.27 | -2.81 | | | 0.00 | down | | |
| MSTRG.19371 | gart | 8.23 | 5.23 | | 6.30 | | | 0.96 | | 1.70 | | 1.08 | 1.49 | | | 0.00 | up | | |
| MSTRG.13449 | bbox1 | 4.10 | 3.18 | | 3.50 | | | 0.77 | | 0.69 | | 0.60 | 1.37 | | | 0.00 | up | | |
| MSTRG.3203 | si:ch1073-89b12.1 | 5.89 | 10.70 | | 10.95 | | | 85.72 | | 80.76 | | 80.40 | -2.81 | | | 0.00 | down | | |
| MSTRG.18659 | cog3 | 5.74 | 3.16 | | 3.80 | | | 0.70 | | 1.13 | | 0.86 | 1.16 | | | 0.00 | up | | |
| MSTRG.3471 | abcc4 (1 of many) | 3.30 | 4.93 | | 3.70 | | | 1.70 | | 1.33 | | 1.14 | 1.25 | | | 0.00 | up | | |
| MSTRG.2141 | si:ch73-190m4.1 | 2.22 | 3.30 | | 3.32 | | | 0.53 | | 0.31 | | 0.55 | 1.60 | | | 0.00 | up | | |
| MSTRG.7492 | wnk1b | 1.19 | 2.47 | | 1.46 | | | 11.53 | | 8.50 | | 7.82 | -1.63 | | | 0.00 | down | | |
| MSTRG.14056 | nfia | 0.45 | 0.52 | | 0.63 | | | 3.50 | | 2.73 | | 2.73 | -1.29 | | | 0.00 | down | | |
| MSTRG.14969 | fam13a | 8.05 | 4.08 | | 8.77 | | | 138.43 | | 134.36 | | 120.33 | -4.32 | | | 0.00 | down | | |
| MSTRG.16591 | glut1 | 0.65 | 0.82 | | 0.70 | | | 17.98 | | 13.85 | | 10.53 | -2.95 | | | 0.00 | down | | |
| MSTRG.15340 | nit2 | 15.72 | 17.72 | | 14.48 | | | 7.39 | | 6.64 | | 6.55 | 1.19 | | | 0.00 | up | | |
| MSTRG.18994 | phyh | 201.02 | 249.48 | | 185.19 | | | 76.82 | | 60.65 | | 60.03 | 1.84 | | | 0.00 | up | | |
| MSTRG.3944 | usp4 | 6.57 | 8.44 | | 7.63 | | | 1.57 | | 0.96 | | 1.71 | 1.99 | | | 0.00 | up | | |
| MSTRG.2448 | si:ch1073-396h14.1 | 2.07 | 0.67 | | 0.78 | | | 9.10 | | 10.49 | | 13.49 | -2.86 | | | 0.00 | down | | |
| MSTRG.20162 | hao2;si:rp71-68n21.9 | 9.86 | 10.83 | | 11.91 | | | 1.63 | | 2.33 | | 1.92 | 1.98 | | | 0.00 | up | | |
| MSTRG.7563 | slc5a8 (1 of many) | 61.32 | 66.80 | | 60.75 | | | 13.36 | | 17.03 | | 17.23 | 1.88 | | | 0.00 | up | | |
| MSTRG.9103 | stat1a | 59.28 | 73.00 | | 94.24 | | | 9.15 | | 7.46 | | 9.83 | 3.09 | | | 0.00 | up | | |
| MSTRG.2830 | ENSONIG00000013072 | 2.18 | 2.55 | | 2.61 | | | 12.30 | | 16.49 | | 13.25 | -2.14 | | | 0.00 | down | | |
| MSTRG.3122 | si:dkey-24l11.2 | 11.57 | 9.88 | | 10.87 | | | 60.84 | | 46.23 | | 49.19 | -2.14 | | | 0.00 | down | | |
| MSTRG.2018 | tat | 78.91 | 129.17 | | 109.28 | | | 21.67 | | 8.08 | | 13.06 | 3.37 | | | 0.00 | up | | |
| MSTRG.20960 | sema6d | 0.62 | 0.62 | | 0.88 | | | 5.90 | | 4.39 | | 5.11 | -1.76 | | | 0.00 | down | | |
| MSTRG.7657 | ENSONIG00000002383 | 31.21 | 36.28 | | 26.29 | | | 7.52 | | 5.62 | | 6.71 | 2.21 | | | 0.00 | up | | |
| MSTRG.22818 | PPP2CA (1 of many) | 101.27 | 79.56 | | 73.08 | | | 236.22 | | 263.55 | | 296.79 | -1.81 | | | 0.00 | down | | |
| MSTRG.16901 | mapk3 | 0.96 | 0.65 | | 1.29 | | | 6.68 | | 6.87 | | 6.60 | -2.06 | | | 0.00 | down | | |
| MSTRG.12056 | rbpjb | 0.48 | 0.45 | | 0.37 | | | 9.44 | | 7.41 | | 6.07 | -2.49 | | | 0.00 | down | | |
| MSTRG.2207 | slc27a6 | 23.16 | 16.28 | | 25.16 | | | 109.51 | | 107.73 | | 111.43 | -2.45 | | | 0.00 | down | | |
| MSTRG.21905 | ENSONIG00000017276 | 5.81 | 7.82 | | 5.56 | | | 1.35 | | 0.79 | | 1.28 | 1.97 | | | 0.00 | up | | |
| MSTRG.16146 | scp2a | 153.48 | 218.90 | | 169.87 | | | 60.09 | | 61.72 | | 61.23 | 1.68 | | | 0.00 | up | | |
| MSTRG.11793 | krt8 | 185.72 | 146.64 | | 155.81 | | | 448.45 | | 485.03 | | 398.70 | -1.56 | | | 0.00 | down | | |
| MSTRG.7588 | si:ch73-252i11.1 | 2.75 | 4.16 | | 3.71 | | | 1.21 | | 0.68 | | 0.48 | 1.61 | | | 0.00 | up | | |
| MSTRG.385 | znf395a | 3.26 | 0.89 | | 1.41 | | | 60.32 | | 53.66 | | 97.91 | -5.08 | | | 0.00 | down | | |
| MSTRG.3079 | miox | 33.47 | 27.68 | | 34.76 | | | 1208.56 | | 656.09 | | 678.78 | -4.50 | | | 0.00 | down | | |
| MSTRG.13338 | ENSONIG00000013103 | 11.21 | 18.47 | | 13.79 | | | 9.26 | | 6.72 | | 6.17 | 1.19 | | | 0.00 | up | | |
| MSTRG.996 | dync2li1 | 4.65 | 8.08 | | 4.98 | | | 1.25 | | 1.01 | | 0.97 | 1.95 | | | 0.00 | up | | |
| MSTRG.8095 | rbp4l (1 of many) | 0.50 | 2.93 | | 1.82 | | | 1.13 | | 0 | | 0 | 1.69 | | | 0.00 | up | | |
| MSTRG.15272 | efna1a | 2.39 | 2.45 | | 2.03 | | | 6.05 | | 6.38 | | 6.95 | -1.20 | | | 0.00 | down | | |
| MSTRG.10885 | mov10b.1 | 31.22 | 28.89 | | 40.20 | | | 4.91 | | 6.08 | | 5.56 | 2.31 | | | 0.00 | up | | |
| MSTRG.19459 | znfx1 | 6.84 | 9.54 | | 12.97 | | | 0.71 | | 0.25 | | 0.39 | 3.11 | | | 0.00 | up | | |
| MSTRG.25315 | ENSONIG00000000241 | 12.16 | 9.35 | | 14.11 | | | 1.18 | | 1.74 | | 1.72 | 2.15 | | | 0.00 | up | | |
| MSTRG.15068 | zfp36l2 | 3.99 | 5.55 | | 4.73 | | | 28.73 | | 18.16 | | 25.43 | -1.87 | | | 0.00 | down | | |
| MSTRG.15152 | ASPH | 9.15 | 9.60 | | 8.91 | | | 68.55 | | 43.71 | | 56.64 | -2.32 | | | 0.00 | down | | |
| MSTRG.9755 | map7d1b | 4.48 | 1.76 | | 3.16 | | | 6.15 | | 9.11 | | 7.24 | -1.48 | | | 0.00 | down | | |
| MSTRG.3084 | wash1 | 4.51 | 5.34 | | 5.83 | | | 21.16 | | 15.71 | | 19.00 | -1.50 | | | 0.00 | down | | |
| MSTRG.9669 | parp12a | 8.15 | 8.98 | | 6.94 | | | 1.79 | | 1.35 | | 1.10 | 2.01 | | | 0.00 | up | | |
| MSTRG.5804 | zgc:162780 | 9.54 | 11.84 | | 10.22 | | | 1.49 | | 2.15 | | 1.51 | 2.12 | | | 0.00 | up | | |
| MSTRG.20544 | brd4 (1 of many) | 0.91 | 1.10 | | 1.27 | | | 4.63 | | 3.60 | | 4.29 | -1.20 | | | 0.00 | down | | |
| MSTRG.8950 | ENSONIG00000006516 | 133.54 | 148.81 | | 186.50 | | | 1077.97 | | 775.36 | | 849.39 | -2.35 | | | 0.00 | down | | |
| MSTRG.16693 | nfil3-5 | 0.32 | 0.36 | | 0.85 | | | 10.88 | | 9.24 | | 8.08 | -2.70 | | | 0.00 | down | | |
| MSTRG.2511 | CYP2J2 (1 of many) | 73.26 | 66.76 | | 59.13 | | | 8.54 | | 13.78 | | 10.32 | 2.35 | | | 0.00 | up | | |
| MSTRG.12805 | rnf213a | 4.67 | 7.79 | | 7.84 | | | 1.03 | | 0.75 | | 1.15 | 2.19 | | | 0.00 | up | | |
| MSTRG.6062 | slc51a | 15.13 | 29.81 | | 15.13 | | | 2.88 | | 1.68 | | 1.28 | 3.22 | | | 0.00 | up | | |
| MSTRG.7939 | kcnk5a | 0.23 | 0.25 | | 0.26 | | | 5.80 | | 6.21 | | 9.21 | -2.72 | | | 0.00 | down | | |
| MSTRG.16439 | appbp2 | 1.40 | 1.81 | | 1.89 | | | 6.01 | | 4.82 | | 4.61 | -1.04 | | | 0.00 | down | | |
| MSTRG.20362 | ENSONIG00000006347 | 4.17 | 8.48 | | 5.30 | | | 23.10 | | 16.86 | | 17.77 | -1.21 | | | 0.00 | down | | |
| MSTRG.12187 | ENSONIG00000019132;ENSONIG00000019137 | 7.34 | 7.05 | | 8.57 | | | 1.38 | | 0.67 | | 0.83 | 2.26 | | | 0.00 | up | | |
| MSTRG.9780 | apoea | 0.28 | 0.41 | | 0.13 | | | 13.29 | | 18.29 | | 10.47 | -3.55 | | | 0.00 | down | | |
| MSTRG.6601 | ENSONIG00000002493 | 14.18 | 20.45 | | 21.17 | | | 1.27 | | 0.22 | | 1.26 | 3.71 | | | 0.00 | up | | |
| MSTRG.16653 | si:dkey-28e7.3 | 1.84 | 1.28 | | 0.98 | | | 9.22 | | 12.55 | | 8.88 | -2.42 | | | 0.00 | down | | |
| MSTRG.1406 | si:dkey-94f20.4 | 0.91 | 1.48 | | 1.40 | | | 0.39 | | 0.24 | | 0.14 | 1.01 | | | 0.00 | up | | |
| MSTRG.2481 | SLC25A42 (1 of many) | 2.55 | 2.25 | | 1.70 | | | 57.04 | | 42.41 | | 79.88 | -4.23 | | | 0.00 | down | | |
| MSTRG.7586 | si:ch73-252i11.1 | 0.80 | 1.32 | | 1.19 | | | 0.43 | | 0.09 | | 0.05 | 1.04 | | | 0.00 | up | | |
| MSTRG.22934 | trim71 | 1.15 | 1.59 | | 0.77 | | | 9.41 | | 9.46 | | 9.84 | -2.23 | | | 0.00 | down | | |
| MSTRG.9688 | si:dkey-23p11.2 | 4.16 | 7.39 | | 6.15 | | | 18.69 | | 16.54 | | 16.89 | -1.18 | | | 0.00 | down | | |
| MSTRG.16407 | rgs4 | 1.63 | 1.39 | | 1.81 | | | 24.35 | | 14.82 | | 20.06 | -2.87 | | | 0.00 | down | | |
| MSTRG.4176 | zgc:162730 | 37.07 | 45.51 | | 49.38 | | | 227.87 | | 152.63 | | 164.83 | -1.78 | | | 0.00 | down | | |
| ENSONIG00000010530 | bco2b (1 of many) | 0 | 0.05 | | 0.01 | | | 1.68 | | 1.23 | | 1.14 | -1.11 | | | 0.00 | down | | |
| MSTRG.8292 | dusp1 | 1.78 | 2.57 | | 2.82 | | | 132.95 | | 49.86 | | 72.55 | -4.14 | | | 0.00 | down | | |
| MSTRG.8195 | cyp27c1 | 8.30 | 19.87 | | 11.55 | | | 0.31 | | 0.44 | | 0.48 | 3.57 | | | 0.00 | up | | |
| MSTRG.12555 | usp25 | 3.29 | 3.86 | | 3.97 | | | 11.89 | | 9.23 | | 9.11 | -1.09 | | | 0.00 | down | | |
| MSTRG.9540 | tpst1l | 10.68 | 10.91 | | 10.29 | | | 25.82 | | 21.51 | | 25.40 | -1.06 | | | 0.00 | down | | |
| MSTRG.5065 | ENSONIG00000009728 | 5.04 | 12.77 | | 10.77 | | | 1.64 | | 1.27 | | 1.05 | 2.54 | | | 0.00 | up | | |
| MSTRG.600 | dsc2l | 14.19 | 18.51 | | 13.07 | | | 4.73 | | 4.58 | | 4.39 | 1.65 | | | 0.00 | up | | |
| MSTRG.6380 | slc27a2b | 43.20 | 38.71 | | 38.67 | | | 3.41 | | 5.68 | | 3.01 | 2.92 | | | 0.00 | up | | |
| MSTRG.6689 | clptm1l | 11.51 | 10.39 | | 9.68 | | | 4.34 | | 3.76 | | 3.85 | 1.20 | | | 0.00 | up | | |
| MSTRG.12696 | hmox2a | 12.03 | 4.27 | | 4.75 | | | 13.86 | | 26.49 | | 25.83 | -2.23 | | | 0.00 | down | | |
| MSTRG.2190 | nadk2 | 1.59 | 1.83 | | 1.85 | | | 16.79 | | 10.40 | | 10.96 | -2.09 | | | 0.00 | down | | |
| MSTRG.1493 | ptpn22 | 0.79 | 0.64 | | 1.06 | | | 3.55 | | 4.69 | | 4.63 | -1.65 | | | 0.00 | down | | |
| MSTRG.14939 | ube2kb | 5.13 | 4.19 | | 4.55 | | | 8.20 | | 11.62 | | 11.46 | -1.20 | | | 0.00 | down | | |
| MSTRG.17086 | SUSD2 (1 of many) | 11.63 | 16.63 | | 16.30 | | | 5.70 | | 5.05 | | 4.06 | 1.62 | | | 0.00 | up | | |
| MSTRG.6508 | cdkn1cb | 0.23 | 0.27 | | 1.17 | | | 212.35 | | 80.30 | | 98.06 | -5.96 | | | 0.00 | down | | |
| MSTRG.24167 | dhrs4 | 9.51 | 17.12 | | 11.44 | | | 2.74 | | 2.53 | | 2.97 | 2.08 | | | 0.00 | up | | |
| MSTRG.1667 | ptpdc1b | 0.14 | 0.00 | | 0 | | | 0.57 | | 1.08 | | 0.71 | -1.75 | | | 0.00 | down | | |
| MSTRG.10657 | unc93b1 | 9.81 | 15.49 | | 15.07 | | | 4.34 | | 2.15 | | 3.60 | 2.08 | | | 0.00 | up | | |
| MSTRG.5554 | ddx39ab (1 of many) | 6.39 | 5.15 | | 9.15 | | | 0 | | 0 | | 0 | 2.89 | | | 0.00 | up | | |
| MSTRG.15034 | primpol | 0.03 | 0.01 | | 0.03 | | | 11.03 | | 6.18 | | 6.16 | -2.97 | | | 0.00 | down | | |
| MSTRG.15920 | pgfb | 0.31 | 0.34 | | 0.56 | | | 3.88 | | 2.83 | | 3.37 | -1.54 | | | 0.00 | down | | |
| MSTRG.18400 | nr1d2b | 4.31 | 9.37 | | 7.96 | | | 2.95 | | 1.93 | | 2.54 | 1.59 | | | 0.00 | up | | |
| MSTRG.6004 | ENSONIG00000014168 | 18.79 | 20.27 | | 22.26 | | | 44.37 | | 45.41 | | 41.28 | -1.02 | | | 0.00 | down | | |
| MSTRG.4375 | RGR (1 of many) | 15.04 | 9.71 | | 12.78 | | | 3.24 | | 3.82 | | 4.47 | 1.23 | | | 0.00 | up | | |
| MSTRG.7306 | chdh | 3.85 | 3.43 | | 3.11 | | | 0.60 | | 0.25 | | 0.33 | 1.72 | | | 0.00 | up | | |
| MSTRG.20157 | fam46c | 0.56 | 0.35 | | 0.44 | | | 8.82 | | 5.68 | | 8.21 | -2.51 | | | 0.00 | down | | |
| MSTRG.3555 | adamts1 | 0.12 | 0.15 | | 0.16 | | | 3.42 | | 5.50 | | 5.96 | -2.48 | | | 0.00 | down | | |
| MSTRG.9268 | ENSONIG00000018838 | 0.11 | 0 | | 0.08 | | | 3.73 | | 6.31 | | 3.71 | -2.52 | | | 0.00 | down | | |
| MSTRG.8560 | plk2b | 2.21 | 1.93 | | 2.41 | | | 8.21 | | 11.77 | | 12.91 | -2.05 | | | 0.00 | down | | |
| MSTRG.3533 | spry2 | 0.74 | 0.95 | | 1.29 | | | 10.38 | | 8.57 | | 6.98 | -2.13 | | | 0.00 | down | | |
| MSTRG.5806 | zgc:162396 | 32.65 | 36.50 | | 40.95 | | | 1.75 | | 3.78 | | 2.90 | 3.22 | | | 0.00 | up | | |
| MSTRG.2609 | kdr | 4.80 | 4.40 | | 3.63 | | | 16.76 | | 14.18 | | 16.74 | -1.68 | | | 0.00 | down | | |
| MSTRG.1849 | smchd1 | 7.77 | 13.91 | | 8.23 | | | 0.49 | | 0.66 | | 0.75 | 2.89 | | | 0.00 | up | | |
| MSTRG.4401 | KCNMA1 (1 of many) | 5.92 | 8.03 | | 5.66 | | | 1.19 | | 1.06 | | 0.60 | 2.11 | | | 0.00 | up | | |
| MSTRG.532 | higd1a | 11.08 | 6.13 | | 10.30 | | | 78.96 | | 64.99 | | 90.11 | -3.17 | | | 0.00 | down | | |
| MSTRG.3404 | ENSONIG00000001186 | 289.40 | 261.29 | | 281.02 | | | 105.54 | | 126.72 | | 136.62 | 1.06 | | | 0.00 | up | | |
| MSTRG.18358 | kdm3b | 2.27 | 2.28 | | 2.91 | | | 7.26 | | 7.78 | | 8.42 | -1.36 | | | 0.00 | down | | |
| MSTRG.19747 | sf3b1 | 32.17 | 45.96 | | 33.30 | | | 115.86 | | 99.08 | | 93.20 | -1.25 | | | 0.00 | down | | |
| MSTRG.15232 | OSBPL10 | 9.39 | 8.46 | | 8.30 | | | 19.45 | | 24.85 | | 27.70 | -1.49 | | | 0.00 | down | | |
| MSTRG.1405 | irf3 | 6.82 | 11.10 | | 11.44 | | | 0.94 | | 0.75 | | 1.36 | 2.61 | | | 0.00 | up | | |
| MSTRG.26982 | ENSONIG00000013886 | 3.21 | 2.19 | | 2.91 | | | 9.10 | | 9.74 | | 7.82 | -1.52 | | | 0.00 | down | | |
| MSTRG.15199 | SNORD22;SNORD26 | 20.05 | 15.34 | | 17.31 | | | 52.60 | | 46.69 | | 45.61 | -1.48 | | | 0.00 | down | | |
| MSTRG.12893 | rmdn1 | 43.10 | 45.06 | | 49.18 | | | 3.70 | | 6.91 | | 6.01 | 2.72 | | | 0.00 | up | | |
| MSTRG.15507 | dbpa | 8.17 | 12.70 | | 6.58 | | | 0.99 | | 0.51 | | 0.37 | 2.89 | | | 0.00 | up | | |
| MSTRG.14506 | nrg2b | 2.52 | 6.61 | | 3.87 | | | 0.08 | | 0.11 | | 0 | 2.60 | | | 0.00 | up | | |
| MSTRG.20198 | fam89a | 3.81 | 4.03 | | 4.04 | | | 19.75 | | 15.24 | | 22.29 | -1.93 | | | 0.00 | down | | |
| MSTRG.19648 | oser1 | 4.36 | 4.08 | | 4.74 | | | 25.97 | | 17.64 | | 20.30 | -1.93 | | | 0.00 | down | | |
| MSTRG.24731 | zgc:92594 | 3.71 | 5.49 | | 5.23 | | | 2.18 | | 1.60 | | 2.17 | 1.15 | | | 0.00 | up | | |
| MSTRG.6897 | rcl1 | 5.66 | 4.69 | | 4.09 | | | 13.93 | | 13.06 | | 14.19 | -1.41 | | | 0.00 | down | | |
| MSTRG.7962 | lgmn | 45.63 | 56.86 | | 63.52 | | | 190.62 | | 151.37 | | 151.92 | -1.38 | | | 0.00 | down | | |
| MSTRG.14246 | skiv2l2 | 4.02 | 5.02 | | 6.37 | | | 29.16 | | 23.61 | | 22.39 | -1.92 | | | 0.00 | down | | |
| MSTRG.4370 | lhpp | 2.03 | 2.66 | | 2.51 | | | 0.77 | | 0.89 | | 0.69 | 1.00 | | | 0.00 | up | | |
| MSTRG.20420 | pld2 | 2.08 | 3.06 | | 2.93 | | | 15.51 | | 10.63 | | 9.66 | -1.53 | | | 0.00 | down | | |
| MSTRG.13620 | shc1 | 1.27 | 1.80 | | 2.30 | | | 9.81 | | 8.74 | | 8.17 | -1.70 | | | 0.00 | down | | |
| MSTRG.11758 | ENSONIG00000012697 | 8.37 | 5.95 | | 6.79 | | | 56.99 | | 82.40 | | 132.98 | -3.75 | | | 0.00 | down | | |
| MSTRG.1814 | insig1 | 4.45 | 3.21 | | 5.00 | | | 106.55 | | 66.54 | | 131.23 | -4.27 | | | 0.00 | down | | |
| MSTRG.20024 | ednrab | 0.16 | 0.10 | | 0.23 | | | 5.88 | | 5.50 | | 9.36 | -2.78 | | | 0.00 | down | | |
| MSTRG.11168 | e2f5 | 12.49 | 17.09 | | 13.41 | | | 1.53 | | 2.15 | | 2.44 | 2.37 | | | 0.00 | up | | |
| MSTRG.2694 | crata (1 of many) | 8.44 | 9.48 | | 7.15 | | | 3.17 | | 2.57 | | 2.80 | 1.36 | | | 0.00 | up | | |
| MSTRG.21132 | adhfe1 | 15.85 | 18.84 | | 20.20 | | | 3.07 | | 4.40 | | 3.86 | 2.01 | | | 0.00 | up | | |
| MSTRG.3492 | gpr137c | 0.97 | 1.55 | | 0.68 | | | 15.08 | | 10.75 | | 9.55 | -2.40 | | | 0.00 | down | | |
| MSTRG.8733 | nup43 | 3.31 | 2.17 | | 3.40 | | | 7.12 | | 9.95 | | 7.99 | -1.46 | | | 0.00 | down | | |
| MSTRG.7702 | LAMP2 | 43.81 | 35.34 | | 49.62 | | | 11.62 | | 13.92 | | 13.08 | 1.52 | | | 0.00 | up | | |
| MSTRG.9339 | aldocb | 5.00 | 4.56 | | 10.39 | | | 290.81 | | 241.90 | | 179.62 | -4.92 | | | 0.00 | down | | |
| MSTRG.16090 | cmpk2 | 16.76 | 18.09 | | 30.47 | | | 1.19 | | 0.60 | | 1.15 | 3.62 | | | 0.00 | up | | |
| MSTRG.19139 | sgsh | 18.93 | 19.96 | | 23.88 | | | 2.86 | | 4.48 | | 3.85 | 2.14 | | | 0.00 | up | | |
| MSTRG.215 | igdcc4 | 6.44 | 9.50 | | 8.15 | | | 4.01 | | 3.85 | | 3.25 | 1.11 | | | 0.00 | up | | |
| MSTRG.3559 | APP (1 of many) | 9.71 | 8.28 | | 8.53 | | | 22.12 | | 19.27 | | 19.37 | -1.13 | | | 0.00 | down | | |
| MSTRG.2286 | aldh7a1 | 41.66 | 49.18 | | 52.11 | | | 5.55 | | 7.22 | | 8.96 | 2.55 | | | 0.00 | up | | |
| MSTRG.18441 | scarb2 (1 of many) | 20.25 | 31.28 | | 24.18 | | | 10.12 | | 9.92 | | 8.04 | 1.53 | | | 0.00 | up | | |
| MSTRG.25390 | slc43a1b | 6.19 | 10.86 | | 6.28 | | | 0.18 | | 0.37 | | 0.43 | 2.85 | | | 0.00 | up | | |
| MSTRG.11283 | cep131 | 0.92 | 1.48 | | 0.75 | | | 9.50 | | 6.07 | | 6.94 | -1.82 | | | 0.00 | down | | |
| MSTRG.2300 | lrrc15 | 3.59 | 3.54 | | 3.71 | | | 29.87 | | 20.57 | | 18.55 | -2.22 | | | 0.00 | down | | |
| MSTRG.14805 | usp18 | 37.51 | 48.21 | | 60.97 | | | 3.02 | | 1.73 | | 4.23 | 3.86 | | | 0.00 | up | | |
| MSTRG.21579 | epoa | 0.73 | 0.33 | | 0.46 | | | 4.42 | | 11.20 | | 7.29 | -2.81 | | | 0.00 | down | | |
| MSTRG.11621 | LTBP4 | 0 | 0.06 | | 0 | | | 0.95 | | 1.34 | | 1.00 | -1.06 | | | 0.00 | down | | |
| MSTRG.13266 | rasip1 | 1.73 | 2.06 | | 2.22 | | | 11.13 | | 7.23 | | 7.99 | -1.50 | | | 0.00 | down | | |
| MSTRG.11875 | met | 4.53 | 8.69 | | 4.67 | | | 38.87 | | 25.23 | | 31.61 | -1.91 | | | 0.00 | down | | |
| MSTRG.16519 | slc31a1 | 9.00 | 10.77 | | 10.43 | | | 30.67 | | 23.48 | | 22.20 | -1.08 | | | 0.00 | down | | |
| MSTRG.9524 | afap1l1b | 0.83 | 1.17 | | 1.18 | | | 7.23 | | 4.58 | | 4.71 | -1.43 | | | 0.00 | down | | |
| MSTRG.20401 | ENSONIG00000013347 | 0.64 | 0.09 | | 0.12 | | | 4.04 | | 3.58 | | 5.15 | -2.24 | | | 0.00 | down | | |
| MSTRG.18967 | adob | 3.42 | 2.86 | | 3.25 | | | 23.74 | | 16.94 | | 26.71 | -2.46 | | | 0.00 | down | | |
| MSTRG.11222 | PPP1CC | 25.15 | 16.05 | | 17.86 | | | 219.15 | | 144.40 | | 164.53 | -3.16 | | | 0.00 | down | | |
| MSTRG.3758 | EPHX1 (1 of many) | 26.39 | 25.10 | | 20.24 | | | 2.52 | | 4.39 | | 4.14 | 2.25 | | | 0.00 | up | | |
| MSTRG.14178 | pgrmc1 | 27.36 | 39.84 | | 25.85 | | | 2.76 | | 3.84 | | 4.08 | 2.86 | | | 0.00 | up | | |
| MSTRG.23352 | ATXN2L (1 of many) | 2.61 | 3.15 | | 3.66 | | | 12.09 | | 13.47 | | 10.83 | -1.61 | | | 0.00 | down | | |
| MSTRG.2242 | zmiz2 (1 of many) | 0.34 | 0.70 | | 0.56 | | | 3.68 | | 3.00 | | 4.31 | -1.47 | | | 0.00 | down | | |
| MSTRG.2970 | ENSONIG00000014640 | 3.28 | 5.77 | | 4.59 | | | 0.48 | | 0.65 | | 0.23 | 2.12 | | | 0.00 | up | | |
| MSTRG.3009 | ENSONIG00000014760 | 0.20 | 0.13 | | 0.60 | | | 3.69 | | 4.55 | | 4.71 | -2.11 | | | 0.00 | down | | |
| MSTRG.5939 | sec24b | 2.27 | 2.10 | | 2.99 | | | 8.32 | | 8.03 | | 7.86 | -1.40 | | | 0.00 | down | | |
| MSTRG.8245 | eif4ebp3 | 9.63 | 13.44 | | 12.71 | | | 60.99 | | 39.41 | | 58.93 | -1.80 | | | 0.00 | down | | |
| MSTRG.11521 | nfe2l3 | 2.34 | 4.66 | | 4.43 | | | 1.30 | | 0.96 | | 0.77 | 1.54 | | | 0.00 | up | | |
| MSTRG.5809 | asb1 | 0.64 | 0.16 | | 0.26 | | | 0.97 | | 1.34 | | 1.64 | -1.01 | | | 0.00 | down | | |
| MSTRG.1124 | foxk2 | 1.95 | 1.49 | | 2.35 | | | 11.15 | | 9.51 | | 13.16 | -2.11 | | | 0.00 | down | | |
| MSTRG.589 | igfbp1b | 49.32 | 32.21 | | 21.28 | | | 3400.14 | | 1483.90 | | 1999.71 | -5.96 | | | 0.00 | down | | |
| MSTRG.13579 | fam171a1 | 0.39 | 0.47 | | 0.52 | | | 1.75 | | 1.91 | | 2.29 | -1.04 | | | 0.00 | down | | |
| MSTRG.3993 | stat2 | 76.88 | 86.91 | | 103.16 | | | 13.32 | | 9.76 | | 16.82 | 2.79 | | | 0.00 | up | | |
| MSTRG.2497 | stap2b | 0 | 0 | | 0.04 | | | 1.18 | | 1.23 | | 0.86 | -1.03 | | | 0.00 | down | | |
| MSTRG.18431 | txnipa | 62.98 | 52.51 | | 69.89 | | | 347.49 | | 294.53 | | 454.38 | -2.59 | | | 0.00 | down | | |
| MSTRG.9907 | dap1b | 38.66 | 50.31 | | 47.40 | | | 5.37 | | 8.74 | | 6.51 | 2.57 | | | 0.00 | up | | |
| MSTRG.11993 | ypel2b | 0.90 | 0.53 | | 0.73 | | | 7.92 | | 5.28 | | 6.27 | -2.10 | | | 0.00 | down | | |
| MSTRG.11697 | ENSONIG00000012486 | 56.51 | 42.86 | | 91.45 | | | 1.44 | | 0.13 | | 0.20 | 5.54 | | | 0.00 | up | | |
| MSTRG.18571 | akip1 | 0.12 | 0.27 | | 0.65 | | | 4.87 | | 3.87 | | 4.50 | -1.90 | | | 0.00 | down | | |
| MSTRG.17965 | ENSONIG00000016546 | 2.46 | 2.95 | | 2.92 | | | 7.45 | | 6.80 | | 8.58 | -1.12 | | | 0.00 | down | | |
| MSTRG.5918 | pcgf1 | 1.42 | 1.59 | | 1.85 | | | 5.91 | | 4.67 | | 4.54 | -1.09 | | | 0.00 | down | | |
| MSTRG.12988 | ENSONIG00000006658 | 11.65 | 13.54 | | 17.08 | | | 124.87 | | 75.06 | | 80.99 | -2.38 | | | 0.00 | down | | |
| MSTRG.10490 | si:dkeyp-69e1.8 (1 of many) | 0.54 | 0.47 | | 0.52 | | | 3.48 | | 3.52 | | 5.17 | -1.78 | | | 0.00 | down | | |
| MSTRG.18789 | ZCCHC2 | 6.41 | 10.69 | | 6.67 | | | 3.36 | | 2.28 | | 2.93 | 1.46 | | | 0.00 | up | | |
| MSTRG.4138 | tpra1 | 7.64 | 10.66 | | 11.45 | | | 4.06 | | 2.12 | | 3.12 | 1.71 | | | 0.00 | up | | |
| MSTRG.2903 | ENSONIG00000013184 | 11.30 | 13.69 | | 13.37 | | | 4.06 | | 2.56 | | 4.21 | 1.76 | | | 0.00 | up | | |
| MSTRG.13300 | ENSONIG00000012949;ENSONIG00000012950 | 29.85 | 21.03 | | 27.85 | | | 7.40 | | 6.27 | | 6.67 | 1.70 | | | 0.00 | up | | |
| MSTRG.4307 | trip12 | 4.77 | 6.32 | | 4.63 | | | 25.14 | | 16.12 | | 17.89 | -1.48 | | | 0.00 | down | | |
| MSTRG.19909 | rhbdd1 (1 of many) | 0.80 | 2.76 | | 1.37 | | | 0.22 | | 0 | | 0.09 | 1.58 | | | 0.00 | up | | |
| MSTRG.21007 | si:ch211-191a24.4 | 0.47 | 0.68 | | 0.59 | | | 4.09 | | 2.48 | | 2.93 | -1.21 | | | 0.00 | down | | |
| MSTRG.17585 | ENSONIG00000021418 | 1.29 | 1.88 | | 1.97 | | | 0.56 | | 0 | | 0.19 | 1.38 | | | 0.00 | up | | |
| MSTRG.17681 | ENSONIG00000013713 | 1.59 | 2.53 | | 1.74 | | | 0.31 | | 0.31 | | 0.41 | 1.25 | | | 0.00 | up | | |
| MSTRG.11977 | apoa1a | 24061.93 | 20527.64 | | 18612.68 | | | 7199.10 | | 9468.88 | | 8164.13 | 1.19 | | | 0.00 | up | | |
| MSTRG.1790 | zgc:153639 | 2.47 | 2.15 | | 3.08 | | | 0.39 | | 0.48 | | 0.46 | 1.24 | | | 0.00 | up | | |
| MSTRG.6749 | cpt2 | 2.96 | 2.89 | | 3.22 | | | 0.71 | | 0.86 | | 1.11 | 1.05 | | | 0.00 | up | | |
| MSTRG.2140 | si:ch73-190m4.1 | 11.53 | 12.95 | | 18.68 | | | 2.21 | | 1.19 | | 1.90 | 2.64 | | | 0.00 | up | | |
| MSTRG.12842 | frya | 4.50 | 6.25 | | 4.76 | | | 2.94 | | 1.79 | | 2.45 | 1.09 | | | 0.00 | up | | |
| MSTRG.7651 | hs3st1 | 0 | 0 | | 0.04 | | | 2.04 | | 1.41 | | 2.20 | -1.44 | | | 0.00 | down | | |
| MSTRG.13782 | ENSONIG00000007420 | 1.10 | 0.98 | | 1.43 | | | 0.04 | | 0.05 | | 0.03 | 1.04 | | | 0.00 | up | | |
| MSTRG.3060 | ccdc79 | 0.05 | 0.05 | | 0.02 | | | 2.79 | | 1.86 | | 1.76 | -1.48 | | | 0.00 | down | | |
| MSTRG.9091 | LECT2 (1 of many) | 99.64 | 109.62 | | 54.90 | | | 6.62 | | 5.00 | | 5.57 | 3.76 | | | 0.00 | up | | |
| MSTRG.17305 | gch1 | 18.49 | 36.93 | | 17.37 | | | 4.85 | | 3.55 | | 3.17 | 2.69 | | | 0.00 | up | | |
| MSTRG.6063 | pdk3a | 0.70 | 0.78 | | 0.73 | | | 6.81 | | 4.72 | | 7.67 | -2.00 | | | 0.00 | down | | |
| MSTRG.15255 | ENSONIG00000019706 | 1.65 | 3.60 | | 3.32 | | | 47.31 | | 22.09 | | 20.64 | -2.43 | | | 0.00 | down | | |
| MSTRG.15477 | ENSONIG00000011844 | 1.00 | 1.31 | | 1.15 | | | 3.39 | | 4.08 | | 3.38 | -1.07 | | | 0.00 | down | | |
| MSTRG.14332 | asmtl | 8.81 | 14.18 | | 13.64 | | | 1.64 | | 2.28 | | 1.56 | 2.36 | | | 0.00 | up | | |
| MSTRG.18631 | casq2 | 1.69 | 1.12 | | 1.55 | | | 0.14 | | 0.06 | | 0.14 | 1.04 | | | 0.00 | up | | |
| MSTRG.22496 | zgc:158482 | 3.07 | 3.43 | | 2.13 | | | 0.23 | | 0.11 | | 0.15 | 1.77 | | | 0.00 | up | | |
| MSTRG.917 | ppp2r2d | 13.86 | 12.85 | | 11.33 | | | 41.31 | | 35.79 | | 32.69 | -1.43 | | | 0.00 | down | | |
| MSTRG.13381 | CALD1 (1 of many) | 6.37 | 6.35 | | 5.55 | | | 23.45 | | 16.83 | | 19.65 | -1.46 | | | 0.00 | down | | |
| MSTRG.3050 | chkb | 16.78 | 14.39 | | 22.63 | | | 1.73 | | 2.19 | | 1.20 | 2.74 | | | 0.00 | up | | |
| MSTRG.23319 | EIF5AL1 | 5.47 | 6.41 | | 6.82 | | | 1.50 | | 2.00 | | 1.96 | 1.36 | | | 0.00 | up | | |
| MSTRG.7398 | eno1a | 6.23 | 3.16 | | 13.03 | | | 437.45 | | 348.29 | | 461.87 | -5.94 | | | 0.00 | down | | |
| MSTRG.17139 | mtp | 0 | 0 | | 0 | | | 36.67 | | 17.73 | | 13.41 | -4.16 | | | 0.00 | down | | |
| MSTRG.21801 | ttyh3b | 5.19 | 5.21 | | 4.07 | | | 1.32 | | 1.54 | | 1.54 | 1.19 | | | 0.00 | up | | |
| MSTRG.4971 | sgf29 | 1.86 | 2.86 | | 3.10 | | | 9.86 | | 8.18 | | 7.60 | -1.21 | | | 0.00 | down | | |
| MSTRG.2039 | zgc:91860 | 0.77 | 0.54 | | 1.26 | | | 6.33 | | 6.24 | | 5.45 | -1.97 | | | 0.00 | down | | |
| MSTRG.22338 | tmem129 | 5.88 | 4.85 | | 7.01 | | | 14.98 | | 16.06 | | 16.48 | -1.38 | | | 0.00 | down | | |
| MSTRG.7408 | slc25a33 | 10.88 | 13.19 | | 10.32 | | | 53.82 | | 86.91 | | 83.79 | -2.67 | | | 0.00 | down | | |
| MSTRG.14149 | sar1b | 54.33 | 47.53 | | 59.24 | | | 22.11 | | 24.40 | | 20.66 | 1.15 | | | 0.00 | up | | |
| MSTRG.14490 | gpc4 | 9.15 | 11.98 | | 9.17 | | | 3.64 | | 4.02 | | 3.25 | 1.35 | | | 0.00 | up | | |
| MSTRG.2513 | CYP2J2 (1 of many) | 199.12 | 187.20 | | 237.01 | | | 1.50 | | 4.09 | | 7.19 | 5.14 | | | 0.00 | up | | |
| MSTRG.14081 | zgc:92140 | 7.64 | 6.38 | | 9.11 | | | 18.56 | | 20.86 | | 20.31 | -1.37 | | | 0.00 | down | | |
| MSTRG.12949 | arl4aa | 4.31 | 7.92 | | 6.76 | | | 2.74 | | 2.32 | | 2.71 | 1.26 | | | 0.00 | up | | |
| MSTRG.13233 | USF2 (1 of many) | 2.29 | 1.58 | | 2.50 | | | 5.73 | | 9.72 | | 7.52 | -1.70 | | | 0.00 | down | | |
| MSTRG.12739 | zgc:171452 | 20.24 | 19.79 | | 19.02 | | | 3.60 | | 1.43 | | 2.70 | 2.75 | | | 0.00 | up | | |
| MSTRG.20108 | gstt1b | 284.17 | 331.72 | | 388.05 | | | 21.64 | | 41.80 | | 32.79 | 3.26 | | | 0.00 | up | | |
| MSTRG.1085 | SLC22A7 (1 of many) | 7.61 | 6.04 | | 5.68 | | | 0.73 | | 0.97 | | 0.36 | 2.05 | | | 0.00 | up | | |
| MSTRG.6902 | nrarpa | 0.66 | 0.68 | | 0.82 | | | 2.74 | | 3.08 | | 3.88 | -1.33 | | | 0.00 | down | | |
| MSTRG.11074 | usp33 | 2.50 | 3.66 | | 2.41 | | | 10.31 | | 8.16 | | 10.05 | -1.28 | | | 0.00 | down | | |
| MSTRG.4955 | med15 | 4.15 | 3.34 | | 4.45 | | | 19.36 | | 18.72 | | 14.13 | -1.91 | | | 0.00 | down | | |
| MSTRG.23572 | ENSONIG00000006906 | 2.35 | 2.45 | | 2.90 | | | 1.09 | | 0.70 | | 0.81 | 1.02 | | | 0.00 | up | | |
| MSTRG.2557 | rangrf | 1.51 | 0 | | 0.59 | | | 0.58 | | 2.41 | | 1.31 | -1.19 | | | 0.00 | down | | |
| MSTRG.22678 | nf1a | 2.80 | 3.02 | | 2.60 | | | 0.33 | | 0.18 | | 0.60 | 1.52 | | | 0.00 | up | | |
| MSTRG.6605 | ENSONIG00000002495 | 85.59 | 99.58 | | 116.42 | | | 17.19 | | 6.54 | | 11.81 | 3.39 | | | 0.00 | up | | |
| MSTRG.18403 | thrb | 17.16 | 25.83 | | 20.47 | | | 7.19 | | 7.22 | | 8.47 | 1.50 | | | 0.00 | up | | |
| MSTRG.110 | ENSONIG00000000060 | 8.06 | 14.21 | | 18.60 | | | 2.68 | | 1.53 | | 1.50 | 2.67 | | | 0.00 | up | | |
| MSTRG.7555 | etnk1 | 16.00 | 11.82 | | 16.07 | | | 37.35 | | 42.12 | | 54.38 | -1.73 | | | 0.00 | down | | |
| MSTRG.3111 | si:ch211-13k12.2 | 3.85 | 5.90 | | 4.96 | | | 30.04 | | 25.66 | | 40.48 | -2.31 | | | 0.00 | down | | |
| MSTRG.2941 | rassf7a | 1.29 | 3.70 | | 2.27 | | | 12.05 | | 7.67 | | 6.85 | -1.08 | | | 0.00 | down | | |
| MSTRG.4348 | gch2 | 3.32 | 4.89 | | 4.15 | | | 1.24 | | 1.25 | | 1.56 | 1.24 | | | 0.00 | up | | |
| MSTRG.7628 | nr2f6b | 0.45 | 1.40 | | 0.68 | | | 4.71 | | 3.51 | | 4.57 | -1.26 | | | 0.00 | down | | |
| MSTRG.14453 | morc2 | 14.73 | 21.28 | | 21.39 | | | 8.63 | | 7.99 | | 8.89 | 1.24 | | | 0.00 | up | | |
| MSTRG.8135 | dhodh | 2.63 | 1.53 | | 3.09 | | | 9.48 | | 12.20 | | 9.62 | -1.97 | | | 0.00 | down | | |
| MSTRG.19612 | mmadhc | 4.86 | 3.26 | | 5.00 | | | 13.85 | | 14.23 | | 18.66 | -1.80 | | | 0.00 | down | | |
| MSTRG.9182 | atf3 | 0.61 | 0.58 | | 2.81 | | | 160.18 | | 84.46 | | 86.94 | -5.39 | | | 0.00 | down | | |
| MSTRG.4445 | dnmbp | 1.36 | 1.93 | | 2.13 | | | 8.53 | | 8.34 | | 6.43 | -1.51 | | | 0.00 | down | | |
| MSTRG.10275 | hgh1 | 0.30 | 0.10 | | 0.08 | | | 2.14 | | 1.84 | | 1.55 | -1.33 | | | 0.00 | down | | |
| MSTRG.4849 | scube2 | 31.51 | 37.46 | | 20.19 | | | 1.14 | | 1.87 | | 2.16 | 3.41 | | | 0.00 | up | | |
| MSTRG.15869 | pcmtl | 7.98 | 9.72 | | 8.16 | | | 1.70 | | 2.43 | | 1.51 | 1.77 | | | 0.00 | up | | |
| MSTRG.17377 | macc1 | 1.39 | 1.60 | | 1.09 | | | 6.68 | | 5.05 | | 6.80 | -1.51 | | | 0.00 | down | | |
| MSTRG.5738 | PLCL2 | 2.08 | 3.09 | | 2.19 | | | 0.50 | | 0.27 | | 0.65 | 1.38 | | | 0.00 | up | | |
| MSTRG.22962 | sacm1la | 17.91 | 16.00 | | 20.65 | | | 51.30 | | 52.35 | | 43.82 | -1.42 | | | 0.00 | down | | |
| MSTRG.9876 | tlk1a | 3.50 | 3.71 | | 3.49 | | | 10.42 | | 7.71 | | 9.66 | -1.07 | | | 0.00 | down | | |
| MSTRG.2933 | ldha | 2.15 | 2.37 | | 3.57 | | | 18.96 | | 14.97 | | 13.76 | -2.07 | | | 0.00 | down | | |
| MSTRG.6697 | dgat1a | 2.66 | 2.93 | | 2.50 | | | 34.24 | | 28.57 | | 18.13 | -2.74 | | | 0.00 | down | | |
| MSTRG.5087 | akt1s1 | 23.56 | 26.03 | | 19.15 | | | 80.81 | | 65.51 | | 79.20 | -1.59 | | | 0.00 | down | | |
| MSTRG.2983 | plekhg7 | 2.00 | 3.68 | | 2.30 | | | 11.78 | | 6.94 | | 8.55 | -1.12 | | | 0.00 | down | | |
| MSTRG.10752 | ECHDC3 | 18.74 | 15.50 | | 15.85 | | | 43.67 | | 51.29 | | 37.33 | -1.45 | | | 0.00 | down | | |
| MSTRG.2217 | arrdc3b | 0.75 | 0.36 | | 0.41 | | | 13.39 | | 9.69 | | 7.31 | -2.84 | | | 0.00 | down | | |
| MSTRG.590 | igfbp1b | 12.57 | 6.09 | | 4.81 | | | 639.51 | | 270.83 | | 409.72 | -5.70 | | | 0.00 | down | | |
| MSTRG.21216 | ENSONIG00000009118 | 3.47 | 2.08 | | 3.28 | | | 0.33 | | 0.40 | | 0.57 | 1.26 | | | 0.00 | up | | |
| MSTRG.7301 | selk | 12.72 | 9.84 | | 13.19 | | | 27.08 | | 27.72 | | 24.94 | -1.20 | | | 0.00 | down | | |
| MSTRG.9446 | ENSONIG00000005342 | 1.21 | 0.54 | | 0.98 | | | 11.30 | | 8.12 | | 7.65 | -2.44 | | | 0.00 | down | | |
| MSTRG.22736 | usp37 | 1.16 | 1.54 | | 1.57 | | | 5.26 | | 4.91 | | 3.90 | -1.11 | | | 0.00 | down | | |
| MSTRG.20393 | ENSONIG00000021870 | 5.25 | 2.72 | | 2.15 | | | 99.96 | | 61.85 | | 59.09 | -4.19 | | | 0.00 | down | | |
| MSTRG.4611 | rhbdf1a | 0.90 | 0.66 | | 0.81 | | | 5.92 | | 4.38 | | 4.02 | -1.64 | | | 0.00 | down | | |
| MSTRG.262 | si:ch211-79l17.1 | 11.26 | 11.70 | | 10.29 | | | 39.47 | | 31.45 | | 28.28 | -1.38 | | | 0.00 | down | | |
| MSTRG.3511 | zgc:92335 | 3.00 | 3.44 | | 3.52 | | | 29.01 | | 16.97 | | 16.59 | -2.06 | | | 0.00 | down | | |
| MSTRG.27420 | abcc5 (1 of many) | 1.09 | 1.76 | | 1.06 | | | 0.23 | | 0.13 | | 0.23 | 1.07 | | | 0.00 | up | | |
| MSTRG.17108 | fbxw8 | 0.49 | 1.04 | | 0.42 | | | 8.42 | | 6.99 | | 5.05 | -2.02 | | | 0.00 | down | | |
| MSTRG.10433 | ENSONIG00000012393 | 5.63 | 7.69 | | 6.69 | | | 25.32 | | 15.44 | | 20.45 | -1.20 | | | 0.00 | down | | |
| MSTRG.12070 | mettl3 | 1.08 | 1.06 | | 2.96 | | | 112.38 | | 158.60 | | 64.19 | -5.37 | | | 0.00 | down | | |
| MSTRG.8805 | aida | 6.15 | 7.24 | | 7.99 | | | 2.65 | | 2.93 | | 2.30 | 1.22 | | | 0.00 | up | | |
| MSTRG.26808 | ENSONIG00000003790 | 2.01 | 4.00 | | 1.97 | | | 0 | | 0 | | 0 | 2.04 | | | 0.00 | up | | |
| MSTRG.9823 | SLC9A1 (1 of many) | 2.83 | 4.19 | | 3.18 | | | 1.98 | | 0.92 | | 0.91 | 1.26 | | | 0.00 | up | | |
| MSTRG.998 | abcg5 | 9.20 | 16.11 | | 8.97 | | | 0.36 | | 0.88 | | 0.68 | 3.00 | | | 0.00 | up | | |
| MSTRG.12253 | mao | 1.04 | 1.83 | | 1.77 | | | 11.27 | | 6.98 | | 6.32 | -1.52 | | | 0.00 | down | | |
| MSTRG.5146 | ENSONIG00000020255 | 0 | 0.16 | | 0.55 | | | 6.49 | | 7.92 | | 10.95 | -2.94 | | | 0.00 | down | | |
| MSTRG.6536 | ndrg1a | 18.27 | 14.66 | | 21.23 | | | 384.00 | | 310.70 | | 180.79 | -3.84 | | | 0.00 | down | | |
| MSTRG.25214 | ENSONIG00000012412 | 1.81 | 1.15 | | 1.05 | | | 0 | | 0.04 | | 0 | 1.05 | | | 0.00 | up | | |
| MSTRG.15002 | arhgef38 | 7.23 | 7.26 | | 5.94 | | | 27.23 | | 19.49 | | 23.43 | -1.55 | | | 0.00 | down | | |
| MSTRG.9443 | sptbn2 | 11.75 | 15.64 | | 16.38 | | | 2.86 | | 3.87 | | 3.66 | 1.85 | | | 0.00 | up | | |
| MSTRG.24841 | ENSONIG00000005902 | 9.46 | 16.38 | | 15.20 | | | 4.48 | | 4.63 | | 3.86 | 1.67 | | | 0.00 | up | | |
| MSTRG.12386 | fbxw7 | 1.52 | 1.63 | | 1.73 | | | 5.67 | | 6.80 | | 4.85 | -1.36 | | | 0.00 | down | | |
| MSTRG.7404 | gpr157 | 1.24 | 1.18 | | 1.38 | | | 3.69 | | 3.44 | | 4.54 | -1.12 | | | 0.00 | down | | |
| MSTRG.22878 | ENSONIG00000018192 | 13.48 | 14.06 | | 11.46 | | | 3.57 | | 2.12 | | 3.43 | 1.92 | | | 0.00 | up | | |
| MSTRG.20194 | fam89a | 0.60 | 0.86 | | 0.65 | | | 3.50 | | 2.57 | | 3.74 | -1.20 | | | 0.00 | down | | |
| MSTRG.1241 | zgc:163107 | 3.25 | 4.25 | | 4.88 | | | 11.55 | | 10.99 | | 10.87 | -1.14 | | | 0.00 | down | | |
| MSTRG.14420 | alkbh2 | 2.15 | 2.75 | | 2.57 | | | 0.09 | | 0.17 | | 0.45 | 1.53 | | | 0.00 | up | | |
| MSTRG.15860 | ivd | 16.50 | 18.52 | | 15.27 | | | 7.56 | | 5.72 | | 7.64 | 1.26 | | | 0.00 | up | | |
| MSTRG.15184 | zgc:110591 | 3.77 | 2.54 | | 2.32 | | | 11.17 | | 9.46 | | 10.93 | -1.69 | | | 0.00 | down | | |
| MSTRG.16776 | si:ch73-209e20.3 | 1.27 | 0.37 | | 0.24 | | | 401.32 | | 134.89 | | 109.92 | -6.69 | | | 0.00 | down | | |
| MSTRG.17903 | gfm1 | 16.04 | 15.48 | | 19.81 | | | 2.72 | | 4.51 | | 3.86 | 1.83 | | | 0.00 | up | | |
| MSTRG.14925 | klf3 | 0.14 | 0.18 | | 0.47 | | | 3.19 | | 2.29 | | 3.03 | -1.54 | | | 0.00 | down | | |
| MSTRG.1145 | SPAG9 (1 of many) | 3.25 | 4.96 | | 3.90 | | | 2.70 | | 1.66 | | 1.43 | 1.07 | | | 0.00 | up | | |
| MSTRG.2166 | si:ch211-122c9.5 | 0.87 | 1.68 | | 1.82 | | | 0.27 | | 0.16 | | 0.15 | 1.22 | | | 0.00 | up | | |
| MSTRG.16430 | SERPINB1 (1 of many) | 8.77 | 7.99 | | 8.92 | | | 31.55 | | 50.89 | | 31.28 | -2.14 | | | 0.00 | down | | |
| MSTRG.4017 | nr1d4b | 1.47 | 1.98 | | 1.06 | | | 7.89 | | 6.18 | | 7.24 | -1.57 | | | 0.00 | down | | |
| MSTRG.21457 | ABHD15 | 6.37 | 11.73 | | 8.27 | | | 0.24 | | 0.81 | | 0.21 | 2.92 | | | 0.00 | up | | |
| MSTRG.15104 | usp1 | 1.55 | 1.53 | | 2.07 | | | 5.21 | | 4.85 | | 5.89 | -1.21 | | | 0.00 | down | | |
| MSTRG.16128 | rgs5b | 0.04 | 0.55 | | 0.80 | | | 21.03 | | 16.07 | | 9.62 | -3.14 | | | 0.00 | down | | |
| MSTRG.10449 | exoc3l4 | 6.04 | 14.57 | | 9.81 | | | 1.20 | | 1.53 | | 1.25 | 2.50 | | | 0.00 | up | | |
| MSTRG.22587 | ubl3a | 10.26 | 7.78 | | 7.98 | | | 47.48 | | 35.55 | | 33.30 | -2.04 | | | 0.00 | down | | |
| MSTRG.15054 | slc1a4 | 1.50 | 0.36 | | 1.91 | | | 10.02 | | 17.46 | | 24.11 | -3.47 | | | 0.00 | down | | |
| MSTRG.12419 | cyp4v8 | 67.45 | 41.11 | | 47.18 | | | 4.26 | | 10.50 | | 10.19 | 2.07 | | | 0.00 | up | | |
| MSTRG.1492 | RSBN1 | 0.54 | 0.47 | | 0.36 | | | 6.12 | | 3.54 | | 4.63 | -1.85 | | | 0.00 | down | | |
| MSTRG.23706 | kng1 | 418.31 | 334.86 | | 404.43 | | | 48.44 | | 90.03 | | 88.89 | 2.07 | | | 0.00 | up | | |
| MSTRG.3979 | prkag1 | 5.99 | 5.33 | | 6.85 | | | 29.45 | | 22.87 | | 35.25 | -2.08 | | | 0.00 | down | | |
| MSTRG.18772 | rnf175 | 2.61 | 2.08 | | 2.91 | | | 0.44 | | 0.31 | | 0.55 | 1.25 | | | 0.00 | up | | |
| MSTRG.6999 | gstt1a | 15.80 | 18.07 | | 17.61 | | | 5.42 | | 3.98 | | 2.67 | 2.07 | | | 0.00 | up | | |
| MSTRG.12983 | ptpn23a | 2.37 | 2.05 | | 2.44 | | | 13.15 | | 9.92 | | 8.62 | -1.73 | | | 0.00 | down | | |
| MSTRG.17029 | ptp4a1 | 3.35 | 4.10 | | 5.14 | | | 21.12 | | 15.53 | | 14.63 | -1.61 | | | 0.00 | down | | |
| MSTRG.23138 | psph | 3.58 | 2.28 | | 2.42 | | | 0.55 | | 0.75 | | 0.46 | 1.08 | | | 0.00 | up | | |
| MSTRG.3407 | glrx5 | 11.77 | 8.16 | | 9.61 | | | 3.70 | | 3.32 | | 3.53 | 1.13 | | | 0.00 | up | | |
| MSTRG.14326 | clybl | 3.19 | 3.85 | | 3.57 | | | 0.31 | | 0.71 | | 0.72 | 1.50 | | | 0.00 | up | | |
| MSTRG.13170 | apoa2 | 3921.29 | 3792.68 | | 2821.05 | | | 853.77 | | 1094.12 | | 1008.01 | 1.72 | | | 0.00 | up | | |
| MSTRG.8058 | fgfr1a | 0.38 | 0.35 | | 0.40 | | | 4.38 | | 3.36 | | 2.62 | -1.59 | | | 0.00 | down | | |
| MSTRG.11452 | ENSONIG00000007712 | 6134.58 | 7064.55 | | 6751.40 | | | 898.20 | | 1344.73 | | 1515.58 | 2.35 | | | 0.00 | up | | |
| MSTRG.14532 | ENSONIG00000015804 | 4.37 | 3.67 | | 4.92 | | | 29.66 | | 25.08 | | 18.57 | -2.21 | | | 0.00 | down | | |
| MSTRG.12470 | ENSONIG00000012022 | 0.24 | 0.36 | | 0.29 | | | 2.83 | | 2.28 | | 1.73 | -1.21 | | | 0.00 | down | | |
| MSTRG.12092 | SAT2 (1 of many) | 5.52 | 19.22 | | 9.80 | | | 7.75 | | 1.14 | | 1.82 | 2.55 | | | 0.00 | up | | |
| MSTRG.1058 | cdh5 | 1.41 | 2.36 | | 1.77 | | | 9.13 | | 5.26 | | 5.58 | -1.10 | | | 0.00 | down | | |
| MSTRG.19467 | gls2b | 2.63 | 5.80 | | 3.89 | | | 0.66 | | 0.73 | | 0.27 | 1.98 | | | 0.00 | up | | |
| MSTRG.2160 | myhz1.2 | 0.86 | 3.17 | | 2.03 | | | 0.22 | | 0.14 | | 0.06 | 1.73 | | | 0.00 | up | | |
| MSTRG.5306 | numb | 4.22 | 4.44 | | 3.67 | | | 11.56 | | 9.00 | | 9.50 | -1.02 | | | 0.00 | down | | |
| MSTRG.18862 | flvcr2b | 13.79 | 17.24 | | 11.95 | | | 2.45 | | 2.68 | | 3.67 | 2.00 | | | 0.00 | up | | |
| MSTRG.22369 | ubtfl | 4.80 | 4.44 | | 3.88 | | | 16.48 | | 22.81 | | 14.44 | -1.89 | | | 0.01 | down | | |
| MSTRG.10965 | h6pd | 24.90 | 35.94 | | 28.40 | | | 1.45 | | 3.68 | | 2.21 | 3.16 | | | 0.01 | up | | |
| MSTRG.13661 | sphk2 | 13.33 | 18.25 | | 12.21 | | | 3.32 | | 3.46 | | 4.33 | 1.81 | | | 0.01 | up | | |
| MSTRG.11417 | parp12b | 4.96 | 10.51 | | 16.44 | | | 0.58 | | 0.10 | | 0.26 | 3.47 | | | 0.01 | up | | |
| MSTRG.4547 | dhx58 | 9.38 | 9.87 | | 16.10 | | | 1.88 | | 1.24 | | 1.13 | 2.51 | | | 0.01 | up | | |
| MSTRG.20064 | aftpha | 4.46 | 4.20 | | 5.27 | | | 10.32 | | 11.00 | | 9.39 | -1.02 | | | 0.01 | down | | |
| MSTRG.12839 | b3glcta | 3.39 | 3.98 | | 2.44 | | | 0.57 | | 0.45 | | 0.52 | 1.55 | | | 0.01 | up | | |
| MSTRG.13635 | adar | 6.14 | 14.79 | | 15.02 | | | 1.84 | | 1.69 | | 1.72 | 2.53 | | | 0.01 | up | | |
| MSTRG.1140 | TOB1 (1 of many) | 10.20 | 12.85 | | 9.10 | | | 59.03 | | 35.05 | | 42.48 | -1.72 | | | 0.01 | down | | |
| MSTRG.18466 | si:ch211-214j8.1 | 28.61 | 38.72 | | 35.75 | | | 1.49 | | 4.01 | | 2.83 | 3.19 | | | 0.01 | up | | |
| MSTRG.3129 | ETFA | 57.98 | 67.67 | | 55.94 | | | 26.37 | | 30.72 | | 25.69 | 1.13 | | | 0.01 | up | | |
| MSTRG.2643 | tmem45a | 0.41 | 0.29 | | 0.14 | | | 11.45 | | 5.45 | | 8.03 | -2.66 | | | 0.01 | down | | |
| MSTRG.11563 | ENSONIG00000004721 | 47.50 | 67.81 | | 48.47 | | | 10.35 | | 14.24 | | 13.26 | 2.07 | | | 0.01 | up | | |
| MSTRG.12394 | uchl1 | 2.75 | 3.25 | | 2.58 | | | 61.13 | | 92.53 | | 36.17 | -3.96 | | | 0.01 | down | | |
| MSTRG.17763 | zgc:174680 | 21.69 | 19.17 | | 16.81 | | | 1.95 | | 3.24 | | 4.27 | 2.11 | | | 0.01 | up | | |
| MSTRG.15921 | cipca | 0.50 | 0.43 | | 0.44 | | | 2.55 | | 1.94 | | 2.91 | -1.23 | | | 0.01 | down | | |
| MSTRG.15712 | srebf1 | 5.06 | 7.81 | | 8.57 | | | 3.70 | | 2.13 | | 1.99 | 1.51 | | | 0.01 | up | | |
| MSTRG.18513 | ENSONIG00000006626 | 0 | 0 | | 0.08 | | | 1.47 | | 1.50 | | 2.33 | -1.45 | | | 0.01 | down | | |
| MSTRG.7438 | si:ch211-244b2.3 | 2.82 | 5.42 | | 5.56 | | | 0.62 | | 0.23 | | 0.85 | 2.11 | | | 0.01 | up | | |
| MSTRG.13760 | hsd17b4 | 26.63 | 33.29 | | 30.10 | | | 6.44 | | 9.24 | | 9.02 | 1.74 | | | 0.01 | up | | |
| MSTRG.10986 | SRGAP3 (1 of many) | 0.20 | 0.14 | | 0.18 | | | 6.23 | | 3.90 | | 3.29 | -2.06 | | | 0.01 | down | | |
| MSTRG.11950 | si:dkey-114g7.4 | 2.30 | 1.93 | | 3.12 | | | 8.62 | | 8.39 | | 7.92 | -1.47 | | | 0.01 | down | | |
| MSTRG.14900 | kdm4c | 0.57 | 1.37 | | 1.48 | | | 5.26 | | 4.38 | | 4.94 | -1.24 | | | 0.01 | down | | |
| MSTRG.23945 | ranbp9 | 2.59 | 3.61 | | 2.78 | | | 10.90 | | 9.71 | | 7.61 | -1.21 | | | 0.01 | down | | |
| MSTRG.22489 | dmxl2 | 1.01 | 1.71 | | 1.37 | | | 6.25 | | 6.16 | | 8.55 | -1.65 | | | 0.01 | down | | |
| MSTRG.10453 | apopt1 | 4.40 | 6.64 | | 6.37 | | | 37.14 | | 24.52 | | 19.97 | -1.70 | | | 0.01 | down | | |
| MSTRG.6499 | AKR1D1 (1 of many) | 40.09 | 56.29 | | 46.94 | | | 12.05 | | 15.97 | | 11.21 | 1.88 | | | 0.01 | up | | |
| MSTRG.1869 | smarcd3b | 1.46 | 1.55 | | 1.16 | | | 18.48 | | 10.34 | | 9.98 | -2.29 | | | 0.01 | down | | |
| MSTRG.11295 | kcnj2a | 0 | 0.02 | | 0.50 | | | 6.77 | | 4.80 | | 4.30 | -2.29 | | | 0.01 | down | | |
| MSTRG.20156 | fam46c | 0.24 | 0 | | 0.11 | | | 21.68 | | 22.08 | | 9.14 | -3.99 | | | 0.01 | down | | |
| MSTRG.22666 | wsb1 | 6.90 | 9.03 | | 4.61 | | | 49.89 | | 36.86 | | 43.62 | -2.36 | | | 0.01 | down | | |
| MSTRG.839 | atp13a3 (1 of many);ENSONIG00000010631 | 3.70 | 5.25 | | 3.56 | | | 1.53 | | 1.48 | | 1.10 | 1.26 | | | 0.01 | up | | |
| MSTRG.10360 | PLD4 | 12.66 | 14.54 | | 31.02 | | | 0.68 | | 0.54 | | 0.56 | 3.69 | | | 0.01 | up | | |
| MSTRG.8725 | dnmt3aa | 3.78 | 5.97 | | 6.92 | | | 1.20 | | 1.24 | | 1.19 | 1.71 | | | 0.01 | up | | |
| MSTRG.11451 | ENSONIG00000007712 | 41.45 | 49.13 | | 70.68 | | | 7.49 | | 5.49 | | 3.00 | 3.38 | | | 0.01 | up | | |
| MSTRG.9388 | gabrr3a | 1.67 | 4.90 | | 2.92 | | | 0.12 | | 0.20 | | 0.11 | 2.12 | | | 0.01 | up | | |
| MSTRG.17154 | bdh2 | 122.54 | 147.93 | | 154.30 | | | 26.83 | | 41.70 | | 33.55 | 2.00 | | | 0.01 | up | | |
| MSTRG.25616 | ENSONIG00000017774 | 4.80 | 2.76 | | 3.18 | | | 0.24 | | 0.31 | | 0.71 | 1.44 | | | 0.01 | up | | |
| MSTRG.554 | agap3 | 0.26 | 0.33 | | 0 | | | 2.22 | | 2.17 | | 1.86 | -1.34 | | | 0.01 | down | | |
| MSTRG.916 | bnip3 | 58.63 | 66.52 | | 48.62 | | | 142.17 | | 152.96 | | 142.29 | -1.30 | | | 0.01 | down | | |
| MSTRG.11356 | si:ch211-94l19.4 | 3.37 | 4.95 | | 3.57 | | | 2.68 | | 1.53 | | 1.51 | 1.05 | | | 0.01 | up | | |
| MSTRG.19800 | ENSONIG00000010888 | 14.82 | 23.86 | | 29.61 | | | 2.33 | | 2.78 | | 3.39 | 2.75 | | | 0.01 | up | | |
| MSTRG.16862 | si:dkeyp-23e4.3 | 0.68 | 0.47 | | 0.71 | | | 2.00 | | 2.40 | | 3.18 | -1.24 | | | 0.01 | down | | |
| MSTRG.23409 | cd79a | 1.92 | 2.30 | | 3.28 | | | 0.22 | | 0.04 | | 0.28 | 1.66 | | | 0.01 | up | | |
| MSTRG.18966 | adob | 1.25 | 0.98 | | 1.36 | | | 6.76 | | 5.44 | | 8.84 | -1.88 | | | 0.01 | down | | |
| MSTRG.19335 | si:dkeyp-9d4.5 | 3.27 | 2.98 | | 4.22 | | | 0.29 | | 0.49 | | 0.71 | 1.50 | | | 0.01 | up | | |
| MSTRG.15112 | SYDE2 | 0.38 | 1.11 | | 0.14 | | | 10.96 | | 7.09 | | 11.56 | -2.58 | | | 0.01 | down | | |
| MSTRG.4818 | ENSONIG00000005911 | 27.71 | 30.76 | | 16.39 | | | 2.15 | | 2.72 | | 2.90 | 2.77 | | | 0.01 | up | | |
| MSTRG.3951 | ip6k2b | 22.53 | 39.30 | | 32.50 | | | 20.14 | | 8.26 | | 8.74 | 1.88 | | | 0.01 | up | | |
| MSTRG.6613 | rbm24a | 1.43 | 2.46 | | 2.68 | | | 10.09 | | 6.76 | | 6.72 | -1.18 | | | 0.01 | down | | |
| MSTRG.14951 | ENSONIG00000009058 | 2.44 | 1.10 | | 1.54 | | | 9.21 | | 7.11 | | 8.01 | -1.93 | | | 0.01 | down | | |
| MSTRG.2807 | phpt1 | 5.07 | 5.38 | | 6.29 | | | 0.90 | | 1.63 | | 1.37 | 1.45 | | | 0.01 | up | | |
| MSTRG.14840 | gpatch3 | 5.33 | 3.27 | | 5.57 | | | 21.42 | | 21.22 | | 16.48 | -2.01 | | | 0.01 | down | | |
| MSTRG.9469 | p4ha2 | 0.76 | 0.29 | | 0.55 | | | 7.98 | | 5.05 | | 5.20 | -2.19 | | | 0.01 | down | | |
| MSTRG.11023 | ENSONIG00000004343 | 3.19 | 2.17 | | 4.01 | | | 0.13 | | 0 | | 0.06 | 1.86 | | | 0.01 | up | | |
| MSTRG.3234 | sgk1 | 3.86 | 5.69 | | 6.82 | | | 51.15 | | 46.89 | | 85.66 | -3.12 | | | 0.01 | down | | |
| MSTRG.2703 | wdr91 | 2.73 | 3.19 | | 3.76 | | | 9.39 | | 7.89 | | 7.32 | -1.00 | | | 0.01 | down | | |
| MSTRG.15015 | smox | 3.87 | 2.71 | | 2.97 | | | 48.82 | | 50.00 | | 24.60 | -3.33 | | | 0.01 | down | | |
| MSTRG.16775 | gal3st1b | 0.15 | 0.11 | | 0.12 | | | 173.04 | | 75.18 | | 35.24 | -5.77 | | | 0.01 | down | | |
| MSTRG.3073 | gtf3c6 | 8.55 | 8.77 | | 12.47 | | | 2.65 | | 2.59 | | 2.06 | 1.70 | | | 0.01 | up | | |
| MSTRG.11348 | pnpo | 1.82 | 4.81 | | 2.87 | | | 0.73 | | 0.60 | | 0.75 | 1.57 | | | 0.01 | up | | |
| MSTRG.11213 | upb1 | 34.85 | 43.35 | | 36.41 | | | 2.57 | | 5.85 | | 5.00 | 2.76 | | | 0.01 | up | | |
| MSTRG.18363 | mapk9 | 0.12 | 0.03 | | 0.07 | | | 5.75 | | 2.85 | | 3.68 | -2.07 | | | 0.01 | down | | |
| MSTRG.22778 | alox12 | 1.28 | 3.90 | | 1.98 | | | 15.94 | | 16.92 | | 17.80 | -2.17 | | | 0.01 | down | | |
| MSTRG.6500 | SLC6A13 (1 of many) | 28.88 | 48.26 | | 55.29 | | | 0.75 | | 2.82 | | 1.16 | 4.18 | | | 0.01 | up | | |
| MSTRG.12422 | tlr3 | 13.92 | 17.36 | | 18.98 | | | 2.10 | | 1.61 | | 3.60 | 2.50 | | | 0.01 | up | | |
| MSTRG.7829 | agpat3 | 12.77 | 12.04 | | 13.65 | | | 2.56 | | 3.95 | | 4.39 | 1.45 | | | 0.01 | up | | |
| MSTRG.3644 | wdfy4 | 1.02 | 2.66 | | 1.86 | | | 0.72 | | 0.54 | | 0.65 | 1.06 | | | 0.01 | up | | |
| MSTRG.23584 | si:ch73-335l21.4 | 0.09 | 0.31 | | 0.22 | | | 319.15 | | 64.04 | | 59.29 | -5.83 | | | 0.01 | down | | |
| MSTRG.18023 | llgl1 | 11.46 | 10.48 | | 7.16 | | | 31.15 | | 37.88 | | 31.99 | -1.82 | | | 0.01 | down | | |
| MSTRG.17031 | hk1 (1 of many) | 0.92 | 0.64 | | 0.53 | | | 4.29 | | 3.02 | | 4.16 | -1.51 | | | 0.01 | down | | |
| MSTRG.23281 | ncf1 | 1.62 | 0.93 | | 1.76 | | | 46.19 | | 23.01 | | 59.01 | -4.07 | | | 0.01 | down | | |
| MSTRG.5826 | esyt3 (1 of many) | 4.68 | 12.62 | | 6.02 | | | 0 | | 0 | | 0.47 | 3.18 | | | 0.01 | up | | |
| MSTRG.4760 | mvda;cyba;pdcd5;urah | 28.21 | 50.05 | | 44.25 | | | 9.48 | | 11.91 | | 8.86 | 2.09 | | | 0.01 | up | | |
| MSTRG.15206 | mark2b | 4.85 | 4.21 | | 4.71 | | | 14.91 | | 12.05 | | 10.93 | -1.25 | | | 0.01 | down | | |
| MSTRG.17877 | kpna4 | 18.71 | 12.67 | | 12.52 | | | 31.58 | | 33.50 | | 29.05 | -1.25 | | | 0.01 | down | | |
| MSTRG.7000 | gstt1a | 438.43 | 382.66 | | 444.07 | | | 106.56 | | 125.15 | | 77.19 | 1.98 | | | 0.01 | up | | |
| MSTRG.9615 | elmo3 | 3.22 | 4.23 | | 4.25 | | | 11.17 | | 14.24 | | 15.25 | -1.55 | | | 0.01 | down | | |
| MSTRG.9402 | zgc:153704 (1 of many) | 1377.70 | 1548.32 | | 931.83 | | | 281.10 | | 304.69 | | 276.10 | 2.14 | | | 0.01 | up | | |
| MSTRG.11294 | kcnj2a | 0.91 | 0.31 | | 0.99 | | | 18.85 | | 12.74 | | 9.68 | -3.06 | | | 0.01 | down | | |
| MSTRG.4959 | jmjd6 | 2.36 | 1.89 | | 2.21 | | | 30.12 | | 17.88 | | 15.13 | -2.62 | | | 0.01 | down | | |
| MSTRG.7841 | HNMT (1 of many) | 19.62 | 19.67 | | 20.91 | | | 2.04 | | 4.73 | | 3.46 | 2.11 | | | 0.01 | up | | |
| MSTRG.11840 | RNF223 | 0.55 | 0.24 | | 0.23 | | | 6.95 | | 4.80 | | 3.93 | -2.17 | | | 0.01 | down | | |
| MSTRG.10553 | PRPSAP1 (1 of many) | 3.19 | 2.95 | | 3.73 | | | 10.99 | | 8.55 | | 11.73 | -1.38 | | | 0.01 | down | | |
| MSTRG.11559 | si:ch211-276i12.9 | 5.97 | 6.74 | | 7.65 | | | 2.54 | | 2.79 | | 1.97 | 1.24 | | | 0.01 | up | | |
| MSTRG.10634 | CBX4 (1 of many) | 0.70 | 0.59 | | 0.40 | | | 12.30 | | 11.33 | | 6.20 | -2.72 | | | 0.01 | down | | |
| MSTRG.1830 | chmp4c | 0.16 | 0.41 | | 0.26 | | | 3.13 | | 2.40 | | 4.13 | -1.60 | | | 0.01 | down | | |
| MSTRG.17682 | ENSONIG00000013713 | 0.53 | 1.71 | | 1.15 | | | 0 | | 0 | | 0 | 1.31 | | | 0.01 | up | | |
| MSTRG.10211 | pcdh12 | 2.34 | 2.85 | | 2.70 | | | 8.46 | | 5.82 | | 8.10 | -1.06 | | | 0.01 | down | | |
| MSTRG.18607 | mfge8b | 9.14 | 8.56 | | 7.05 | | | 2.19 | | 3.06 | | 3.11 | 1.17 | | | 0.01 | up | | |
| MSTRG.16616 | cadm1a | 0.53 | 2.48 | | 1.48 | | | 0.57 | | 0.02 | | 0.46 | 1.31 | | | 0.01 | up | | |
| MSTRG.24185 | dhrs13b | 6.34 | 11.02 | | 5.95 | | | 1.58 | | 1.72 | | 1.36 | 1.94 | | | 0.01 | up | | |
| MSTRG.13214 | pnrc2 | 24.40 | 35.79 | | 26.62 | | | 78.12 | | 91.15 | | 89.28 | -1.45 | | | 0.01 | down | | |
| MSTRG.11068 | acadm | 101.98 | 122.10 | | 83.98 | | | 36.05 | | 28.66 | | 38.55 | 1.66 | | | 0.01 | up | | |
| MSTRG.2244 | pargl | 12.56 | 14.89 | | 23.26 | | | 2.21 | | 1.18 | | 2.49 | 2.76 | | | 0.01 | up | | |
| MSTRG.17598 | ENSONIG00000009001 | 0.01 | 0 | | 0.19 | | | 1.04 | | 1.71 | | 1.28 | -1.21 | | | 0.01 | down | | |
| MSTRG.19874 | ENSONIG00000007672 | 1.42 | 2.16 | | 1.55 | | | 7.52 | | 5.96 | | 4.75 | -1.17 | | | 0.01 | down | | |
| MSTRG.4742 | ENSONIG00000021392 | 0.15 | 0.03 | | 0.04 | | | 3.41 | | 1.88 | | 2.42 | -1.63 | | | 0.01 | down | | |
| MSTRG.8043 | slc15a4 | 0.77 | 1.31 | | 1.59 | | | 4.69 | | 3.62 | | 4.08 | -1.03 | | | 0.01 | down | | |
| MSTRG.12838 | hs3st3b1a | 19.06 | 24.43 | | 16.96 | | | 123.04 | | 72.59 | | 118.55 | -2.07 | | | 0.01 | down | | |
| MSTRG.7561 | slc5a8 (1 of many) | 37.26 | 42.08 | | 41.82 | | | 5.22 | | 9.52 | | 9.06 | 2.12 | | | 0.01 | up | | |
| MSTRG.15937 | ENSONIG00000020177 | 201.15 | 191.70 | | 166.35 | | | 78.32 | | 75.55 | | 93.73 | 1.13 | | | 0.01 | up | | |
| MSTRG.9779 | apoa4a | 0.03 | 0.08 | | 0.10 | | | 0.79 | | 1.37 | | 1.17 | -1.04 | | | 0.01 | down | | |
| MSTRG.6786 | atg4b | 6.48 | 7.01 | | 8.39 | | | 18.65 | | 17.01 | | 15.11 | -1.03 | | | 0.01 | down | | |
| MSTRG.3243 | si:ch211-286f9.2 | 18.93 | 12.66 | | 19.17 | | | 2.94 | | 4.53 | | 2.57 | 1.82 | | | 0.01 | up | | |
| MSTRG.17367 | cyp21a2 | 147.81 | 110.66 | | 136.33 | | | 19.31 | | 40.51 | | 33.48 | 1.74 | | | 0.01 | up | | |
| MSTRG.9817 | granzyme | 1.41 | 1.56 | | 3.42 | | | 46.98 | | 34.23 | | 23.78 | -3.34 | | | 0.01 | down | | |
| MSTRG.9237 | adgrg6 | 0.49 | 0.55 | | 0.90 | | | 7.76 | | 4.19 | | 5.35 | -1.82 | | | 0.01 | down | | |
| MSTRG.13090 | ENSONIG00000018405 | 4.64 | 6.43 | | 3.60 | | | 0 | | 0 | | 0.45 | 2.42 | | | 0.01 | up | | |
| MSTRG.4077 | srgap2 | 2.67 | 3.64 | | 2.69 | | | 7.05 | | 8.14 | | 7.86 | -1.07 | | | 0.01 | down | | |
| MSTRG.8157 | dync1li2 | 4.28 | 6.56 | | 4.51 | | | 2.71 | | 0.95 | | 1.03 | 1.68 | | | 0.01 | up | | |
| MSTRG.6596 | azin1b | 6.93 | 7.44 | | 7.69 | | | 153.02 | | 55.54 | | 111.23 | -3.26 | | | 0.01 | down | | |
| MSTRG.2512 | CYP2J2 (1 of many) | 24.52 | 10.14 | | 29.38 | | | 0.61 | | 0.64 | | 0.49 | 3.36 | | | 0.01 | up | | |
| MSTRG.9046 | acad9 | 13.59 | 17.99 | | 22.48 | | | 4.46 | | 5.26 | | 4.15 | 1.84 | | | 0.01 | up | | |
| MSTRG.7236 | ENSONIG00000005412 | 1.58 | 3.29 | | 1.72 | | | 0.39 | | 0.28 | | 0.42 | 1.43 | | | 0.01 | up | | |
| MSTRG.19839 | cbx8a | 2.20 | 2.57 | | 2.85 | | | 6.57 | | 7.91 | | 6.12 | -1.13 | | | 0.01 | down | | |
| MSTRG.16851 | wwc1 | 3.32 | 5.77 | | 4.23 | | | 1.80 | | 1.91 | | 1.48 | 1.18 | | | 0.01 | up | | |
| MSTRG.26323 | ENSONIG00000007395 | 71.14 | 106.12 | | 105.35 | | | 1.36 | | 3.78 | | 5.73 | 4.37 | | | 0.01 | up | | |
| MSTRG.15437 | BIN3 | 5.74 | 4.44 | | 5.26 | | | 15.10 | | 11.80 | | 12.59 | -1.22 | | | 0.01 | down | | |
| MSTRG.5118 | hsd3b7 | 31.18 | 31.99 | | 31.93 | | | 86.41 | | 110.79 | | 74.38 | -1.50 | | | 0.01 | down | | |
| MSTRG.19892 | zgc:152863 | 0.25 | 0.24 | | 0.24 | | | 3.33 | | 2.01 | | 3.46 | -1.55 | | | 0.01 | down | | |
| MSTRG.12055 | rbpjb | 2.66 | 1.30 | | 3.03 | | | 41.05 | | 24.26 | | 23.91 | -3.21 | | | 0.01 | down | | |
| MSTRG.1917 | anxa13 | 10.28 | 11.86 | | 14.00 | | | 37.31 | | 28.16 | | 28.18 | -1.14 | | | 0.01 | down | | |
| MSTRG.23141 | si:dkey-95o3.4 | 0.93 | 1.71 | | 0.78 | | | 13.15 | | 7.58 | | 6.75 | -1.89 | | | 0.01 | down | | |
| MSTRG.23606 | ENSONIG00000016482 | 4.95 | 4.34 | | 2.40 | | | 0 | | 0 | | 0 | 2.18 | | | 0.01 | up | | |
| MSTRG.10809 | pfkma | 4.14 | 4.65 | | 4.12 | | | 10.08 | | 9.64 | | 12.82 | -1.13 | | | 0.01 | down | | |
| MSTRG.24519 | mpv17 | 6.78 | 11.16 | | 13.33 | | | 1.40 | | 1.93 | | 1.42 | 2.27 | | | 0.01 | up | | |
| MSTRG.23104 | SCGN (1 of many) | 0 | 0.01 | | 0 | | | 3.24 | | 1.83 | | 3.58 | -1.82 | | | 0.01 | down | | |
| MSTRG.5658 | zgc:162198 | 7.52 | 8.81 | | 10.43 | | | 1.29 | | 1.79 | | 2.36 | 1.81 | | | 0.01 | up | | |
| MSTRG.18984 | pcgf5a | 2.02 | 1.78 | | 2.36 | | | 10.60 | | 7.08 | | 7.45 | -1.51 | | | 0.01 | down | | |
| MSTRG.26855 | mesdc1 | 0 | 0 | | 0 | | | 4.16 | | 1.93 | | 2.69 | -1.76 | | | 0.01 | down | | |
| MSTRG.14118 | hsd11b1la (1 of many) | 92.55 | 106.82 | | 95.22 | | | 7.23 | | 12.15 | | 17.04 | 2.85 | | | 0.01 | up | | |
| MSTRG.3302 | zgc:172246 | 2545.53 | 2824.28 | | 2284.23 | | | 216.94 | | 300.13 | | 114.64 | 3.72 | | | 0.01 | up | | |
| MSTRG.18365 | gfpt2 | 0.20 | 0.12 | | 0.22 | | | 0.83 | | 1.43 | | 1.59 | -1.07 | | | 0.01 | down | | |
| MSTRG.9901 | RBMS1 (1 of many) | 4.37 | 3.52 | | 7.51 | | | 135.50 | | 65.86 | | 67.13 | -3.65 | | | 0.01 | down | | |
| MSTRG.7439 | si:ch211-244b2.4 | 2.30 | 3.32 | | 5.15 | | | 0.23 | | 0.12 | | 0.22 | 2.08 | | | 0.01 | up | | |
| MSTRG.8999 | si:dkey-97a13.6 | 106.19 | 115.45 | | 148.41 | | | 26.49 | | 27.19 | | 38.45 | 1.98 | | | 0.01 | up | | |
| MSTRG.19830 | ENSONIG00000017173 | 2.64 | 2.03 | | 3.69 | | | 0.18 | | 0.11 | | 0.21 | 1.62 | | | 0.01 | up | | |
| MSTRG.4327 | ENSONIG00000020661 | 1.33 | 2.59 | | 2.48 | | | 0.65 | | 0.42 | | 0.75 | 1.17 | | | 0.01 | up | | |
| MSTRG.9942 | pfkla | 0.92 | 1.42 | | 1.66 | | | 7.73 | | 5.22 | | 4.67 | -1.31 | | | 0.01 | down | | |
| MSTRG.16902 | mapk3 | 0.58 | 0.52 | | 0.64 | | | 2.47 | | 3.09 | | 4.37 | -1.52 | | | 0.01 | down | | |
| MSTRG.4385 | DDIT4 (1 of many) | 1.51 | 0.87 | | 1.17 | | | 109.31 | | 32.27 | | 95.01 | -4.84 | | | 0.01 | down | | |
| MSTRG.3792 | egln1a | 0.78 | 1.01 | | 1.14 | | | 5.17 | | 5.32 | | 8.31 | -1.85 | | | 0.01 | down | | |
| MSTRG.11058 | pck1 | 0.05 | 0 | | 0.02 | | | 1042.30 | | 171.08 | | 116.28 | -7.43 | | | 0.01 | down | | |
| MSTRG.18906 | nek7 | 3.71 | 4.48 | | 4.32 | | | 20.00 | | 13.91 | | 11.71 | -1.41 | | | 0.01 | down | | |
| MSTRG.21511 | SLC39A1 | 1.96 | 0.45 | | 2.00 | | | 8.45 | | 37.53 | | 40.73 | -4.21 | | | 0.01 | down | | |
| MSTRG.6604 | ENSONIG00000002494 | 27.73 | 30.46 | | 20.29 | | | 3.24 | | 0.25 | | 1.25 | 3.95 | | | 0.01 | up | | |
| MSTRG.23946 | ENSONIG00000001492 | 2.52 | 4.47 | | 4.36 | | | 1.61 | | 1.09 | | 1.69 | 1.19 | | | 0.01 | up | | |
| MSTRG.5651 | st13 | 21.39 | 17.02 | | 24.48 | | | 61.35 | | 58.42 | | 50.32 | -1.46 | | | 0.01 | down | | |
| MSTRG.26658 | ENSONIG00000003999 | 28.05 | 32.48 | | 27.29 | | | 2.33 | | 5.72 | | 3.03 | 2.61 | | | 0.01 | up | | |
| MSTRG.17095 | wsb2 | 9.07 | 10.83 | | 7.31 | | | 71.78 | | 51.40 | | 38.48 | -2.21 | | | 0.01 | down | | |
| MSTRG.22925 | ENSONIG00000021006 | 28.52 | 24.00 | | 48.91 | | | 603.71 | | 1777.08 | | 3299.75 | -6.01 | | | 0.01 | down | | |
| MSTRG.6811 | atp1b1a | 6.59 | 5.72 | | 6.69 | | | 26.33 | | 17.60 | | 19.35 | -1.50 | | | 0.01 | down | | |
| MSTRG.18715 | gstt2 | 44.32 | 42.65 | | 52.35 | | | 17.61 | | 22.87 | | 18.87 | 1.12 | | | 0.01 | up | | |
| MSTRG.5316 | tmem206 | 9.64 | 23.04 | | 18.97 | | | 7.48 | | 6.62 | | 5.51 | 1.62 | | | 0.01 | up | | |
| MSTRG.26833 | nlrx1 | 2.28 | 2.58 | | 3.10 | | | 6.46 | | 7.58 | | 6.19 | -1.07 | | | 0.01 | down | | |
| MSTRG.11167 | ackr4a | 65.03 | 77.76 | | 71.71 | | | 15.82 | | 24.41 | | 15.64 | 1.89 | | | 0.01 | up | | |
| MSTRG.13862 | ENSONIG00000007488 | 0.32 | 0 | | 0.11 | | | 0.67 | | 2.18 | | 1.54 | -1.39 | | | 0.01 | down | | |
| MSTRG.22013 | tm9sf1 | 11.40 | 5.18 | | 5.45 | | | 0.71 | | 1.59 | | 1.79 | 1.30 | | | 0.01 | up | | |
| MSTRG.6595 | ATP6V1C1 (1 of many) | 4.32 | 7.06 | | 5.82 | | | 72.17 | | 28.63 | | 31.29 | -2.14 | | | 0.01 | down | | |
| MSTRG.4301 | agfg1a | 1.31 | 0.92 | | 1.29 | | | 4.20 | | 3.66 | | 3.12 | -1.13 | | | 0.01 | down | | |
| MSTRG.4292 | BCHE | 55.02 | 93.12 | | 86.25 | | | 12.64 | | 11.65 | | 20.22 | 2.53 | | | 0.01 | up | | |
| MSTRG.10815 | si:dkey-157g16.6 | 2.83 | 3.42 | | 3.85 | | | 10.76 | | 14.63 | | 17.29 | -1.83 | | | 0.01 | down | | |
| MSTRG.5145 | ENSONIG00000020254 | 1.18 | 0.94 | | 1.80 | | | 4.04 | | 5.86 | | 5.61 | -1.57 | | | 0.01 | down | | |
| MSTRG.7248 | si:zfos-80g12.1 | 4.95 | 2.28 | | 4.11 | | | 38.37 | | 35.21 | | 21.34 | -2.95 | | | 0.01 | down | | |
| MSTRG.391 | nqo1 (1 of many) | 26.90 | 25.03 | | 25.56 | | | 6.97 | | 11.57 | | 8.77 | 1.27 | | | 0.01 | up | | |
| MSTRG.7562 | slc5a8 (1 of many) | 16.66 | 19.65 | | 16.59 | | | 1.73 | | 2.89 | | 3.98 | 2.22 | | | 0.01 | up | | |
| MSTRG.5908 | qdpra | 123.44 | 206.87 | | 148.05 | | | 38.59 | | 51.39 | | 43.54 | 1.94 | | | 0.01 | up | | |
| MSTRG.11184 | tet3 | 1.39 | 2.38 | | 0.93 | | | 12.65 | | 11.48 | | 8.70 | -2.05 | | | 0.01 | down | | |
| MSTRG.24646 | RIDA (1 of many) | 60.11 | 71.98 | | 57.17 | | | 22.91 | | 28.66 | | 21.39 | 1.38 | | | 0.01 | up | | |
| MSTRG.18045 | ENSONIG00000015704 | 5.45 | 6.65 | | 4.52 | | | 0.72 | | 1.36 | | 1.06 | 1.64 | | | 0.01 | up | | |
| MSTRG.23353 | sult1st6 | 12.25 | 15.78 | | 12.02 | | | 2.29 | | 3.06 | | 3.97 | 1.82 | | | 0.01 | up | | |
| MSTRG.17347 | ENSONIG00000002744 | 5.92 | 5.17 | | 5.17 | | | 2.01 | | 1.71 | | 1.17 | 1.30 | | | 0.01 | up | | |
| MSTRG.2243 | ogdhb | 1.47 | 1.45 | | 2.28 | | | 0.22 | | 0.15 | | 0.19 | 1.21 | | | 0.01 | up | | |
| MSTRG.1688 | ENSONIG00000017244 | 8.06 | 5.58 | | 6.37 | | | 0.29 | | 1.39 | | 1.47 | 1.58 | | | 0.01 | up | | |
| MSTRG.17140 | TSPAN5 (1 of many) | 11.76 | 20.54 | | 10.55 | | | 6.35 | | 4.09 | | 4.29 | 1.68 | | | 0.01 | up | | |
| MSTRG.2601 | tmem165 | 4.35 | 4.77 | | 5.31 | | | 1.38 | | 1.91 | | 1.99 | 1.04 | | | 0.01 | up | | |
| MSTRG.16759 | setd1ba | 1.92 | 4.96 | | 2.40 | | | 0.94 | | 0.73 | | 0.61 | 1.51 | | | 0.01 | up | | |
| MSTRG.20481 | SLC16A6 (1 of many) | 0.16 | 0.07 | | 0.06 | | | 8.27 | | 5.89 | | 3.56 | -2.49 | | | 0.01 | down | | |
| MSTRG.3171 | hacd3 | 42.38 | 70.49 | | 69.89 | | | 38.48 | | 17.80 | | 27.30 | 1.58 | | | 0.01 | up | | |
| MSTRG.7622 | mknk2a | 5.82 | 3.44 | | 8.69 | | | 266.50 | | 98.61 | | 151.98 | -4.45 | | | 0.01 | down | | |
| MSTRG.23022 | TCEB3 | 4.76 | 5.68 | | 4.81 | | | 21.46 | | 15.66 | | 13.02 | -1.33 | | | 0.01 | down | | |
| MSTRG.14403 | tctn2 | 0.47 | 0.40 | | 0.63 | | | 1.93 | | 1.97 | | 2.80 | -1.15 | | | 0.01 | down | | |
| MSTRG.10705 | tspan9b | 94.59 | 87.38 | | 115.20 | | | 36.54 | | 44.21 | | 44.54 | 1.13 | | | 0.01 | up | | |
| MSTRG.19334 | grapb | 3.14 | 2.77 | | 3.73 | | | 0.59 | | 0.63 | | 1.04 | 1.20 | | | 0.01 | up | | |
| MSTRG.13960 | dcst2 | 1.80 | 1.51 | | 2.73 | | | 0.11 | | 0 | | 0 | 1.52 | | | 0.01 | up | | |
| MSTRG.23081 | JMY (1 of many) | 0.96 | 1.43 | | 0.75 | | | 7.78 | | 5.92 | | 4.54 | -1.58 | | | 0.01 | down | | |
| MSTRG.10406 | atrn | 12.26 | 11.59 | | 9.47 | | | 0.89 | | 2.18 | | 2.32 | 1.93 | | | 0.01 | up | | |
| MSTRG.25265 | ENSONIG00000019976 | 39.01 | 24.19 | | 34.77 | | | 12.30 | | 12.58 | | 11.98 | 1.11 | | | 0.01 | up | | |
| MSTRG.1482 | si:dkey-205h13.1 | 65.83 | 61.00 | | 47.37 | | | 13.86 | | 13.56 | | 19.48 | 1.76 | | | 0.01 | up | | |
| MSTRG.16802 | cenpv | 2.01 | 1.63 | | 2.41 | | | 4.00 | | 4.86 | | 5.43 | -1.05 | | | 0.01 | down | | |
| MSTRG.11147 | INSR (1 of many) | 2.10 | 3.68 | | 4.55 | | | 15.86 | | 13.45 | | 16.60 | -1.68 | | | 0.01 | down | | |
| MSTRG.14739 | rgra | 7.60 | 9.10 | | 7.12 | | | 0.64 | | 1.39 | | 1.68 | 1.94 | | | 0.01 | up | | |
| MSTRG.3762 | flrt3 | 2.20 | 0.08 | | 1.41 | | | 7.23 | | 7.13 | | 8.59 | -2.57 | | | 0.01 | down | | |
| MSTRG.7973 | fbln5 | 0.66 | 1.50 | | 1.21 | | | 4.68 | | 4.80 | | 3.62 | -1.15 | | | 0.01 | down | | |
| MSTRG.20377 | rxfp3.3a1 | 0.12 | 0 | | 0.07 | | | 2.08 | | 1.19 | | 1.56 | -1.23 | | | 0.01 | down | | |
| ENSONIG00000008285 | abcc5 (1 of many) | 1.12 | 1.68 | | 0.95 | | | 0.17 | | 0.11 | | 0.24 | 1.03 | | | 0.01 | up | | |
| MSTRG.2046 | lsp1 | 12.10 | 18.55 | | 13.04 | | | 52.28 | | 30.35 | | 35.73 | -1.02 | | | 0.01 | down | | |
| MSTRG.26053 | tac1 | 1.92 | 11.55 | | 5.66 | | | 1.29 | | 0.78 | | 1.05 | 2.34 | | | 0.01 | up | | |
| MSTRG.16914 | fam195b | 7.25 | 8.59 | | 9.59 | | | 29.33 | | 20.01 | | 20.07 | -1.13 | | | 0.01 | down | | |
| MSTRG.20376 | MORF4L1 (1 of many) | 2.03 | 2.93 | | 1.32 | | | 10.09 | | 9.63 | | 10.33 | -1.76 | | | 0.01 | down | | |
| MSTRG.5997 | pdx1 | 3.22 | 3.68 | | 3.58 | | | 0.23 | | 0.54 | | 0.87 | 1.50 | | | 0.01 | up | | |
| MSTRG.5829 | si:ch211-219a4.6 | 5.76 | 10.59 | | 12.41 | | | 3.39 | | 0.96 | | 2.32 | 2.22 | | | 0.01 | up | | |
| MSTRG.15064 | xpo1a | 3.54 | 5.49 | | 2.73 | | | 28.66 | | 28.18 | | 18.86 | -2.24 | | | 0.01 | down | | |
| MSTRG.3891 | slc25a20 | 9.28 | 12.05 | | 12.71 | | | 2.81 | | 4.14 | | 3.20 | 1.52 | | | 0.01 | up | | |
| MSTRG.20996 | ENSONIG00000010847 | 0.16 | 0.68 | | 0.56 | | | 8.94 | | 7.52 | | 4.18 | -2.12 | | | 0.01 | down | | |
| MSTRG.21598 | serpinf2a | 52.16 | 77.84 | | 46.31 | | | 21.25 | | 17.96 | | 12.89 | 1.94 | | | 0.01 | up | | |
| MSTRG.1125 | foxk2 | 0.81 | 0.80 | | 0.74 | | | 3.30 | | 7.18 | | 7.08 | -2.13 | | | 0.01 | down | | |
| MSTRG.14720 | stox1 | 0.69 | 0.59 | | 0.82 | | | 1.79 | | 3.25 | | 2.86 | -1.24 | | | 0.01 | down | | |
| MSTRG.22438 | im:6907928 | 7.43 | 4.81 | | 10.12 | | | 0.70 | | 0.78 | | 0.55 | 2.14 | | | 0.01 | up | | |
| MSTRG.8185 | cul4a | 12.45 | 11.81 | | 11.71 | | | 4.38 | | 4.19 | | 5.98 | 1.12 | | | 0.01 | up | | |
| MSTRG.10790 | zgc:154045 | 11.91 | 13.27 | | 11.90 | | | 35.82 | | 28.31 | | 24.47 | -1.04 | | | 0.01 | down | | |
| MSTRG.26740 | osbpl5 | 1.54 | 3.33 | | 1.60 | | | 12.87 | | 6.83 | | 8.29 | -1.30 | | | 0.01 | down | | |
| MSTRG.7599 | ivns1abpa | 13.77 | 12.64 | | 11.58 | | | 58.83 | | 38.05 | | 39.02 | -1.63 | | | 0.01 | down | | |
| MSTRG.4534 | ppp1r1b | 26.44 | 25.11 | | 20.50 | | | 3.07 | | 6.66 | | 4.19 | 1.98 | | | 0.01 | up | | |
| MSTRG.26004 | usp2a | 4.11 | 7.73 | | 3.94 | | | 1.26 | | 1.25 | | 0.89 | 1.75 | | | 0.01 | up | | |
| MSTRG.22893 | c6ast1 | 0.40 | 0.33 | | 0.68 | | | 9.82 | | 4.70 | | 8.95 | -2.39 | | | 0.01 | down | | |
| MSTRG.8643 | aldh2.2 | 162.45 | 169.26 | | 121.77 | | | 56.02 | | 63.09 | | 57.62 | 1.31 | | | 0.01 | up | | |
| MSTRG.25454 | ENSONIG00000004801 | 5.17 | 4.40 | | 6.52 | | | 0.07 | | 0.33 | | 0.86 | 2.03 | | | 0.01 | up | | |
| MSTRG.12714 | si:ch211-114l13.7 | 1.48 | 1.26 | | 1.20 | | | 3.23 | | 3.06 | | 4.29 | -1.02 | | | 0.01 | down | | |
| MSTRG.14938 | ube2kb | 96.46 | 119.86 | | 78.65 | | | 29.88 | | 25.75 | | 36.23 | 1.74 | | | 0.01 | up | | |
| MSTRG.23094 | enpp2 (1 of many) | 19.66 | 26.82 | | 17.13 | | | 10.73 | | 8.91 | | 9.53 | 1.20 | | | 0.01 | up | | |
| MSTRG.3761 | flrt3 | 5.26 | 5.94 | | 5.70 | | | 1.01 | | 1.09 | | 1.95 | 1.51 | | | 0.01 | up | | |
| MSTRG.18533 | her6 | 0.93 | 0.39 | | 1.30 | | | 4.34 | | 4.00 | | 4.89 | -1.68 | | | 0.01 | down | | |
| MSTRG.7069 | ctsla | 244.66 | 224.85 | | 223.04 | | | 2730.56 | | 1274.77 | | 1328.56 | -2.62 | | | 0.01 | down | | |
| MSTRG.2518 | CYP2J2 (1 of many) | 18.60 | 21.75 | | 17.85 | | | 8.20 | | 6.64 | | 9.77 | 1.25 | | | 0.01 | up | | |
| MSTRG.6785 | farp2 | 3.02 | 6.08 | | 4.65 | | | 1.78 | | 0.87 | | 1.99 | 1.47 | | | 0.01 | up | | |
| MSTRG.24943 | pck2 | 151.83 | 101.44 | | 82.17 | | | 595.49 | | 442.35 | | 695.19 | -2.52 | | | 0.01 | down | | |
| MSTRG.1479 | agmat | 10.31 | 15.45 | | 11.10 | | | 1.43 | | 2.90 | | 1.74 | 2.18 | | | 0.01 | up | | |
| MSTRG.4706 | hgs | 5.18 | 5.17 | | 5.75 | | | 17.90 | | 14.50 | | 12.02 | -1.20 | | | 0.01 | down | | |
| MSTRG.21318 | nfkb2 | 3.72 | 2.19 | | 7.44 | | | 50.35 | | 61.57 | | 40.23 | -3.51 | | | 0.01 | down | | |
| MSTRG.422 | pkp3b | 6.78 | 6.39 | | 7.65 | | | 120.52 | | 73.92 | | 46.16 | -3.05 | | | 0.01 | down | | |
| MSTRG.22585 | b3glctb | 0.97 | 1.45 | | 1.43 | | | 0.02 | | 0.23 | | 0.17 | 1.04 | | | 0.01 | up | | |
| MSTRG.22514 | WHAMM | 3.33 | 3.25 | | 4.19 | | | 8.51 | | 10.76 | | 8.04 | -1.18 | | | 0.01 | down | | |
| MSTRG.24078 | CSNK1G2 (1 of many) | 4.66 | 6.62 | | 6.27 | | | 17.04 | | 17.54 | | 13.09 | -1.15 | | | 0.01 | down | | |
| MSTRG.22239 | pdzd3b | 14.83 | 30.60 | | 11.37 | | | 3.43 | | 1.76 | | 3.06 | 2.73 | | | 0.01 | up | | |
| MSTRG.16421 | fetub (1 of many) | 274.45 | 229.21 | | 169.30 | | | 49.73 | | 64.65 | | 43.98 | 1.91 | | | 0.01 | up | | |
| MSTRG.2461 | faf1 | 4.86 | 5.24 | | 6.40 | | | 15.79 | | 29.22 | | 22.97 | -1.98 | | | 0.01 | down | | |
| MSTRG.17999 | hpx | 8624.97 | 7889.96 | | 5599.63 | | | 20253.86 | | 19835.88 | | 22730.85 | -1.59 | | | 0.01 | down | | |
| MSTRG.20411 | si:ch211-137i24.10 (1 of many) | 31.37 | 43.06 | | 36.79 | | | 14.24 | | 18.03 | | 13.98 | 1.29 | | | 0.01 | up | | |
| MSTRG.12687 | mxi1 | 0.41 | 1.20 | | 0.50 | | | 4.06 | | 4.04 | | 5.31 | -1.53 | | | 0.01 | down | | |
| MSTRG.7431 | anks1b | 0.21 | 0.17 | | 0.17 | | | 3.21 | | 2.13 | | 1.72 | -1.38 | | | 0.01 | down | | |
| MSTRG.10147 | ENSONIG00000012951 | 1.48 | 1.63 | | 2.00 | | | 0.58 | | 0 | | 0.14 | 1.33 | | | 0.01 | up | | |
| MSTRG.6739 | NAALADL2 | 0.95 | 0.13 | | 0.81 | | | 1.17 | | 2.19 | | 2.61 | -1.27 | | | 0.01 | down | | |
| MSTRG.3984 | arhgef25b | 1.77 | 1.23 | | 2.22 | | | 9.90 | | 6.67 | | 8.14 | -1.73 | | | 0.01 | down | | |
| MSTRG.14421 | unga | 5.11 | 3.09 | | 5.43 | | | 0.52 | | 1.23 | | 0.67 | 1.34 | | | 0.01 | up | | |
| MSTRG.3796 | ntpcr | 2.41 | 1.77 | | 1.31 | | | 7.08 | | 7.10 | | 5.47 | -1.51 | | | 0.01 | down | | |
| MSTRG.21459 | taok1a | 2.68 | 4.10 | | 1.70 | | | 0.01 | | 0 | | 0 | 2.01 | | | 0.01 | up | | |
| MSTRG.1234 | xrcc6 | 1.12 | 1.58 | | 2.19 | | | 5.97 | | 5.03 | | 5.55 | -1.19 | | | 0.01 | down | | |
| MSTRG.10148 | ENSONIG00000012951 | 1.91 | 4.85 | | 5.16 | | | 0.73 | | 0.66 | | 0.36 | 1.94 | | | 0.01 | up | | |
| MSTRG.10978 | uba7 | 0.67 | 1.76 | | 1.77 | | | 0.32 | | 0.21 | | 0.23 | 1.16 | | | 0.01 | up | | |
| MSTRG.19113 | pgam1b;exosc1 | 5.05 | 4.10 | | 6.86 | | | 72.39 | | 94.99 | | 37.91 | -3.44 | | | 0.01 | down | | |
| MSTRG.6179 | ENSONIG00000007222 | 1.91 | 2.47 | | 4.95 | | | 53.02 | | 112.08 | | 205.76 | -5.01 | | | 0.01 | down | | |
| MSTRG.14862 | mrs2 | 6.37 | 4.36 | | 7.81 | | | 0.75 | | 1.53 | | 1.18 | 1.48 | | | 0.01 | up | | |
| MSTRG.19848 | MSRB1 (1 of many) | 15.14 | 13.48 | | 22.60 | | | 1.41 | | 2.62 | | 3.04 | 2.25 | | | 0.01 | up | | |
| MSTRG.12683 | pnpla6 | 6.71 | 5.21 | | 5.55 | | | 10.74 | | 18.50 | | 21.71 | -1.64 | | | 0.01 | down | | |
| MSTRG.8810 | hlx1 | 3.79 | 2.71 | | 4.66 | | | 9.47 | | 9.53 | | 11.24 | -1.37 | | | 0.01 | down | | |
| MSTRG.6839 | snx24 | 6.65 | 4.14 | | 5.49 | | | 35.57 | | 28.91 | | 20.29 | -2.24 | | | 0.01 | down | | |
| MSTRG.18262 | ENSONIG00000011213 | 0.76 | 4.08 | | 2.71 | | | 0 | | 0 | | 0 | 2.16 | | | 0.01 | up | | |
| MSTRG.4890 | itga3a | 3.12 | 8.73 | | 6.46 | | | 2.92 | | 0.46 | | 2.07 | 2.02 | | | 0.01 | up | | |
| MSTRG.4208 | ENSONIG00000005185 | 10.00 | 16.34 | | 8.12 | | | 1.88 | | 1.85 | | 2.54 | 2.13 | | | 0.01 | up | | |
| MSTRG.5605 | wbp2nl | 8.22 | 5.65 | | 7.47 | | | 21.49 | | 16.30 | | 20.22 | -1.40 | | | 0.01 | down | | |
| MSTRG.24966 | ENSONIG00000014008 | 0 | 0.57 | | 0 | | | 2.44 | | 2.84 | | 2.73 | -1.51 | | | 0.01 | down | | |
| MSTRG.5091 | si:ch211-51c14.1 | 1.43 | 1.71 | | 2.29 | | | 13.74 | | 7.03 | | 10.18 | -1.74 | | | 0.01 | down | | |
| MSTRG.14863 | mrs2 | 16.71 | 19.81 | | 16.75 | | | 1.01 | | 3.22 | | 2.51 | 2.42 | | | 0.01 | up | | |
| MSTRG.4978 | ENSONIG00000021447 | 1.45 | 0.74 | | 0.36 | | | 1958.05 | | 210.02 | | 278.53 | -7.49 | | | 0.01 | down | | |
| MSTRG.4537 | p3h4 | 0.09 | 0.09 | | 0.04 | | | 1.11 | | 1.67 | | 0.92 | -1.10 | | | 0.01 | down | | |
| MSTRG.3612 | cox15 | 3.47 | 3.79 | | 5.32 | | | 14.82 | | 13.33 | | 11.32 | -1.36 | | | 0.01 | down | | |
| MSTRG.4434 | PHOSPHO1 (1 of many) | 1.11 | 2.72 | | 3.11 | | | 0 | | 0.06 | | 0.06 | 1.88 | | | 0.01 | up | | |
| MSTRG.3630 | mgea5 | 5.95 | 10.48 | | 5.65 | | | 26.62 | | 25.62 | | 28.17 | -1.57 | | | 0.01 | down | | |
| MSTRG.2019 | cetp | 51.39 | 73.79 | | 33.43 | | | 4.79 | | 7.53 | | 6.14 | 2.87 | | | 0.01 | up | | |
| MSTRG.11103 | vapal | 10.09 | 7.48 | | 9.18 | | | 12.82 | | 16.84 | | 20.57 | -1.05 | | | 0.01 | down | | |
| MSTRG.9315 | stoml3b | 0.58 | 0.44 | | 1.77 | | | 38.12 | | 15.23 | | 20.57 | -3.43 | | | 0.01 | down | | |
| MSTRG.20796 | pip4k2ca | 9.44 | 8.85 | | 10.35 | | | 0.91 | | 2.39 | | 0.89 | 2.03 | | | 0.01 | up | | |
| MSTRG.6122 | ENSONIG00000007019 | 12.01 | 20.27 | | 15.97 | | | 7.52 | | 2.11 | | 4.96 | 2.14 | | | 0.01 | up | | |
| MSTRG.13895 | ENSONIG00000001722 | 0.94 | 2.62 | | 1.85 | | | 0.85 | | 0.37 | | 0 | 1.41 | | | 0.01 | up | | |
| MSTRG.20345 | jph1b | 0.40 | 0 | | 0.10 | | | 0.35 | | 1.22 | | 1.28 | -1.04 | | | 0.01 | down | | |
| MSTRG.4604 | polr3k | 6.88 | 6.73 | | 5.29 | | | 21.57 | | 17.78 | | 15.15 | -1.32 | | | 0.01 | down | | |
| MSTRG.14422 | si:rp71-1c23.3 | 10.45 | 16.12 | | 13.74 | | | 6.43 | | 6.83 | | 7.27 | 1.02 | | | 0.01 | up | | |
| MSTRG.10556 | PGLYRP2 (1 of many) | 26.23 | 16.57 | | 14.81 | | | 45.86 | | 59.03 | | 88.57 | -2.02 | | | 0.01 | down | | |
| MSTRG.1119 | mettl9 | 3.49 | 6.89 | | 6.55 | | | 2.42 | | 2.04 | | 2.52 | 1.24 | | | 0.01 | up | | |
| MSTRG.15316 | cdh2 | 39.19 | 32.91 | | 31.49 | | | 17.71 | | 13.88 | | 16.13 | 1.06 | | | 0.01 | up | | |
| MSTRG.1035 | map4k3b | 1.46 | 2.34 | | 1.17 | | | 10.59 | | 6.16 | | 6.65 | -1.43 | | | 0.01 | down | | |
| MSTRG.14679 | ENSONIG00000007908 | 3.07 | 3.41 | | 3.57 | | | 8.07 | | 12.93 | | 13.60 | -1.62 | | | 0.01 | down | | |
| MSTRG.2820 | bnip3lb | 5.67 | 8.47 | | 5.19 | | | 21.63 | | 14.88 | | 17.13 | -1.10 | | | 0.01 | down | | |
| MSTRG.9985 | ENSONIG00000015071 | 5.60 | 8.01 | | 6.00 | | | 0.85 | | 1.37 | | 1.78 | 1.73 | | | 0.01 | up | | |
| MSTRG.11774 | arrdc2 | 16.86 | 15.78 | | 18.80 | | | 40.43 | | 41.63 | | 60.21 | -1.47 | | | 0.01 | down | | |
| MSTRG.2727 | ENSONIG00000012673 | 0.69 | 0.95 | | 0.18 | | | 10.14 | | 10.81 | | 5.84 | -2.53 | | | 0.01 | down | | |
| MSTRG.5936 | arl9 | 1.53 | 0.78 | | 0.73 | | | 6.80 | | 5.78 | | 4.29 | -1.81 | | | 0.01 | down | | |
| MSTRG.16063 | SLC25A29 (1 of many) | 1.60 | 2.14 | | 1.21 | | | 14.11 | | 7.65 | | 7.83 | -1.73 | | | 0.01 | down | | |
| MSTRG.14441 | kctd10 | 1.40 | 1.42 | | 0.86 | | | 6.23 | | 5.29 | | 4.18 | -1.42 | | | 0.01 | down | | |
| MSTRG.6043 | ube2w | 0.79 | 0.39 | | 0.57 | | | 2.19 | | 1.74 | | 2.57 | -1.08 | | | 0.01 | down | | |
| MSTRG.4442 | cpn1 | 25.82 | 35.95 | | 24.40 | | | 3.31 | | 6.24 | | 5.80 | 2.25 | | | 0.01 | up | | |
| MSTRG.9186 | CYP2W1 (1 of many) | 74.88 | 102.31 | | 96.03 | | | 20.38 | | 20.95 | | 33.04 | 1.94 | | | 0.01 | up | | |
| MSTRG.18491 | MIB2 | 9.08 | 12.74 | | 7.86 | | | 64.74 | | 33.44 | | 38.31 | -1.72 | | | 0.01 | down | | |
| MSTRG.15481 | zgc:174904 | 50.60 | 49.72 | | 33.95 | | | 3.56 | | 5.49 | | 8.62 | 2.59 | | | 0.01 | up | | |
| MSTRG.17391 | tax1bp1b | 16.06 | 14.19 | | 15.86 | | | 71.30 | | 52.02 | | 42.23 | -1.67 | | | 0.01 | down | | |
| MSTRG.17008 | XPO1 (1 of many) | 3.00 | 1.53 | | 2.89 | | | 18.38 | | 14.47 | | 10.88 | -2.25 | | | 0.01 | down | | |
| MSTRG.8324 | si:ch211-153b23.5 | 0.24 | 0.60 | | 0.37 | | | 2.51 | | 3.84 | | 2.52 | -1.46 | | | 0.01 | down | | |
| MSTRG.11818 | znf341 | 1.31 | 0.75 | | 1.37 | | | 3.04 | | 3.74 | | 2.63 | -1.10 | | | 0.01 | down | | |
| MSTRG.22265 | gtpbp4 | 23.61 | 38.23 | | 25.65 | | | 1.44 | | 1.61 | | 4.46 | 3.27 | | | 0.01 | up | | |
| MSTRG.15330 | CARTPT (1 of many) | 6.96 | 10.05 | | 20.58 | | | 0.25 | | 0.13 | | 0.66 | 3.40 | | | 0.01 | up | | |
| MSTRG.22735 | rqcd1 | 3.54 | 3.48 | | 4.43 | | | 11.04 | | 11.31 | | 8.39 | -1.20 | | | 0.01 | down | | |
| MSTRG.3196 | chid1 | 8.85 | 7.11 | | 6.26 | | | 1.78 | | 3.20 | | 2.69 | 1.02 | | | 0.01 | up | | |
| MSTRG.4183 | ENSONIG00000005018 | 10.82 | 14.83 | | 27.94 | | | 2.66 | | 1.92 | | 1.77 | 2.75 | | | 0.01 | up | | |
| MSTRG.7220 | macrod1 | 4.26 | 4.16 | | 5.73 | | | 1.32 | | 1.53 | | 1.81 | 1.13 | | | 0.01 | up | | |
| MSTRG.6965 | ddr2l | 1.47 | 1.36 | | 1.20 | | | 5.69 | | 3.74 | | 5.67 | -1.28 | | | 0.01 | down | | |
| MSTRG.11221 | pptc7a | 0.57 | 0.71 | | 0.46 | | | 14.15 | | 6.19 | | 6.08 | -2.24 | | | 0.01 | down | | |
| MSTRG.12640 | prox1a | 0.56 | 0.33 | | 0.78 | | | 2.12 | | 4.30 | | 4.91 | -1.83 | | | 0.01 | down | | |
| MSTRG.8086 | atad1a | 8.34 | 13.51 | | 5.72 | | | 0.30 | | 0.86 | | 0.46 | 2.74 | | | 0.01 | up | | |
| MSTRG.15635 | ENSONIG00000002020 | 17.15 | 15.50 | | 36.30 | | | 2.34 | | 0.85 | | 1.54 | 3.32 | | | 0.01 | up | | |
| MSTRG.20924 | jmjd7 | 4.60 | 4.31 | | 6.21 | | | 1.53 | | 1.88 | | 1.54 | 1.13 | | | 0.01 | up | | |
| MSTRG.15189 | badb | 3.99 | 5.77 | | 3.69 | | | 2.86 | | 1.57 | | 2.02 | 1.07 | | | 0.01 | up | | |
| MSTRG.16410 | si:ch211-284e20.8 | 234.90 | 318.24 | | 273.08 | | | 16.31 | | 40.47 | | 35.24 | 3.05 | | | 0.01 | up | | |
| MSTRG.16951 | mtr | 10.13 | 16.92 | | 17.25 | | | 0.94 | | 2.31 | | 0.64 | 2.92 | | | 0.01 | up | | |
| MSTRG.3251 | si:ch73-21k16.5 (1 of many) | 10.53 | 10.42 | | 11.63 | | | 3.58 | | 4.62 | | 2.76 | 1.34 | | | 0.01 | up | | |
| MSTRG.20916 | ccdc88c | 1.96 | 2.58 | | 1.06 | | | 9.27 | | 8.75 | | 9.93 | -1.81 | | | 0.01 | down | | |
| MSTRG.16037 | acadl | 31.95 | 26.04 | | 31.45 | | | 12.90 | | 8.23 | | 10.27 | 1.48 | | | 0.01 | up | | |
| MSTRG.18815 | MED13L | 1.32 | 1.21 | | 2.07 | | | 5.79 | | 4.65 | | 5.13 | -1.25 | | | 0.01 | down | | |
| MSTRG.2626 | nek1 | 0.57 | 1.08 | | 0.32 | | | 3.49 | | 3.96 | | 3.60 | -1.44 | | | 0.01 | down | | |
| MSTRG.791 | si:dkey-208k22.3 | 2.64 | 1.42 | | 3.49 | | | 15.47 | | 11.53 | | 13.49 | -2.16 | | | 0.01 | down | | |
| MSTRG.15797 | si:dkey-186o21.1 | 164.65 | 251.43 | | 149.75 | | | 39.07 | | 35.10 | | 57.82 | 2.23 | | | 0.01 | up | | |
| MSTRG.3972 | pa2g4a | 10.66 | 9.73 | | 13.59 | | | 4.77 | | 5.08 | | 4.58 | 1.04 | | | 0.01 | up | | |
| MSTRG.10073 | hmg20a | 1.99 | 1.60 | | 1.61 | | | 6.19 | | 4.37 | | 4.65 | -1.11 | | | 0.01 | down | | |
| MSTRG.13607 | aqp10a | 31.04 | 93.59 | | 74.97 | | | 5.72 | | 7.81 | | 9.33 | 3.23 | | | 0.01 | up | | |
| MSTRG.229 | ENSONIG00000020600 | 9.63 | 7.17 | | 12.20 | | | 2.00 | | 2.72 | | 2.82 | 1.40 | | | 0.01 | up | | |
| MSTRG.5799 | acmsd | 103.44 | 112.45 | | 102.37 | | | 9.93 | | 19.00 | | 23.26 | 2.42 | | | 0.01 | up | | |
| MSTRG.16652 | jupa | 6.70 | 9.47 | | 7.28 | | | 57.63 | | 31.61 | | 26.62 | -1.74 | | | 0.01 | down | | |
| MSTRG.1309 | fam20a | 5.67 | 15.44 | | 8.70 | | | 44.78 | | 36.58 | | 29.33 | -1.39 | | | 0.01 | down | | |
| MSTRG.26852 | ENSONIG00000013907 | 5.11 | 8.69 | | 5.80 | | | 61.84 | | 60.29 | | 140.87 | -3.35 | | | 0.01 | down | | |
| MSTRG.6973 | GRAMD3 (1 of many) | 20.49 | 27.28 | | 19.88 | | | 58.09 | | 77.07 | | 85.48 | -1.65 | | | 0.01 | down | | |
| MSTRG.2864 | hic1 | 0 | 0 | | 0 | | | 1.61 | | 0.85 | | 1.57 | -1.12 | | | 0.01 | down | | |
| MSTRG.24727 | supt16h | 3.43 | 3.34 | | 4.63 | | | 8.73 | | 9.91 | | 8.16 | -1.08 | | | 0.01 | down | | |
| MSTRG.9212 | crnkl1 | 3.52 | 2.28 | | 3.59 | | | 9.28 | | 7.70 | | 7.88 | -1.26 | | | 0.01 | down | | |
| MSTRG.6436 | taf3 | 3.86 | 3.54 | | 3.96 | | | 11.24 | | 7.93 | | 8.49 | -1.00 | | | 0.01 | down | | |
| MSTRG.1383 | ENSONIG00000020022 | 2.16 | 5.06 | | 3.13 | | | 0.96 | | 0.65 | | 1.30 | 1.45 | | | 0.01 | up | | |
| MSTRG.3140 | klf13 | 4.73 | 11.97 | | 3.92 | | | 2.10 | | 0.51 | | 0.99 | 2.33 | | | 0.01 | up | | |
| MSTRG.19986 | si:ch211-149b19.3 | 1.73 | 0.73 | | 0.30 | | | 48.06 | | 119.97 | | 23.07 | -5.21 | | | 0.01 | down | | |
| MSTRG.18852 | fosab | 0.53 | 0.89 | | 0.43 | | | 10.03 | | 4.00 | | 6.28 | -1.87 | | | 0.01 | down | | |
| MSTRG.11795 | tns2a | 6.42 | 9.60 | | 6.40 | | | 35.32 | | 18.38 | | 25.97 | -1.34 | | | 0.01 | down | | |
| MSTRG.5376 | nceh1a | 50.21 | 76.67 | | 47.49 | | | 5.26 | | 11.12 | | 8.46 | 2.66 | | | 0.01 | up | | |
| MSTRG.10899 | zgc:194242 | 56.77 | 40.00 | | 63.24 | | | 16.59 | | 22.50 | | 21.12 | 1.12 | | | 0.01 | up | | |
| MSTRG.10326 | DLL4 (1 of many) | 1.50 | 2.42 | | 2.54 | | | 8.07 | | 5.16 | | 7.67 | -1.09 | | | 0.01 | down | | |
| MSTRG.11706 | nppc | 0 | 0 | | 0 | | | 18.57 | | 5.63 | | 7.00 | -2.98 | | | 0.01 | down | | |
| MSTRG.2638 | ENSONIG00000003159 | 2.23 | 2.12 | | 2.70 | | | 8.27 | | 6.24 | | 5.67 | -1.11 | | | 0.01 | down | | |
| MSTRG.21125 | tkfc | 63.47 | 72.98 | | 69.14 | | | 10.55 | | 20.68 | | 17.34 | 1.92 | | | 0.01 | up | | |
| MSTRG.9910 | CCDC148 | 9.61 | 9.10 | | 7.50 | | | 3.70 | | 2.82 | | 3.98 | 1.14 | | | 0.01 | up | | |
| MSTRG.6149 | grb10b | 1.32 | 2.53 | | 0.95 | | | 34.24 | | 16.86 | | 12.65 | -2.59 | | | 0.01 | down | | |
| MSTRG.19547 | pigl | 1.04 | 1.84 | | 1.32 | | | 4.14 | | 5.45 | | 4.56 | -1.18 | | | 0.01 | down | | |
| MSTRG.6758 | aldh9a1a.1 | 153.52 | 100.71 | | 164.88 | | | 21.26 | | 38.76 | | 20.30 | 2.06 | | | 0.01 | up | | |
| MSTRG.27076 | ENSONIG00000017781 | 0.89 | 2.35 | | 1.01 | | | 0.01 | | 0.01 | | 0.06 | 1.42 | | | 0.01 | up | | |
| MSTRG.1966 | tnfsf10 (1 of many) | 2.71 | 5.82 | | 6.40 | | | 0.85 | | 0.63 | | 1.23 | 1.90 | | | 0.01 | up | | |
| MSTRG.5334 | zgc:175176 | 2.87 | 9.57 | | 2.92 | | | 0.04 | | 0.07 | | 0.12 | 2.74 | | | 0.01 | up | | |
| MSTRG.3957 | pfkfb4b | 0.68 | 0.42 | | 1.26 | | | 8.54 | | 5.49 | | 10.47 | -2.33 | | | 0.01 | down | | |
| MSTRG.7918 | slc4a1ap | 1.80 | 2.15 | | 2.74 | | | 5.11 | | 6.35 | | 6.07 | -1.09 | | | 0.01 | down | | |
| MSTRG.1443 | ENSONIG00000021134 | 142.43 | 48.65 | | 182.94 | | | 918.01 | | 876.45 | | 1258.41 | -3.66 | | | 0.01 | down | | |
| MSTRG.13316 | si:ch73-86n18.1 (1 of many) | 105.01 | 37.49 | | 162.62 | | | 479.19 | | 963.85 | | 745.59 | -3.63 | | | 0.01 | down | | |
| MSTRG.17990 | si:dkey-91i10.3 | 8.84 | 18.64 | | 11.70 | | | 1.93 | | 2.09 | | 3.51 | 2.21 | | | 0.01 | up | | |
| MSTRG.19781 | xaf1 | 4.82 | 5.94 | | 11.03 | | | 0.89 | | 0.58 | | 0.89 | 2.29 | | | 0.01 | up | | |
| MSTRG.969 | ENSONIG00000000833 | 1.97 | 3.21 | | 2.33 | | | 15.26 | | 9.72 | | 7.53 | -1.40 | | | 0.01 | down | | |
| MSTRG.3975 | erbb3a | 0.33 | 0.38 | | 0.24 | | | 2.84 | | 1.76 | | 1.64 | -1.07 | | | 0.01 | down | | |
| MSTRG.10664 | vwa11 | 0.14 | 0.11 | | 0.52 | | | 5.63 | | 2.82 | | 3.51 | -1.79 | | | 0.01 | down | | |
| MSTRG.22713 | zgc:153521 | 8.05 | 4.69 | | 6.35 | | | 2.03 | | 1.81 | | 2.32 | 1.05 | | | 0.01 | up | | |
| MSTRG.15419 | sorbs3 | 5.44 | 8.30 | | 7.38 | | | 53.17 | | 38.41 | | 24.10 | -1.92 | | | 0.01 | down | | |
| MSTRG.13145 | vezf1a | 1.58 | 0.79 | | 1.21 | | | 5.57 | | 3.77 | | 5.22 | -1.49 | | | 0.01 | down | | |
| MSTRG.9861 | scrn3 | 20.14 | 25.11 | | 40.36 | | | 7.04 | | 6.88 | | 6.01 | 2.03 | | | 0.01 | up | | |
| MSTRG.24583 | TMPRSS6 | 36.75 | 37.54 | | 42.51 | | | 5.80 | | 11.84 | | 10.22 | 1.81 | | | 0.01 | up | | |
| MSTRG.2778 | gadd45gb.1 | 0.10 | 0 | | 0.04 | | | 1.27 | | 3.02 | | 1.43 | -1.62 | | | 0.01 | down | | |
| MSTRG.18433 | gatad2b | 0.20 | 0.09 | | 0.04 | | | 1.30 | | 1.53 | | 0.86 | -1.04 | | | 0.01 | down | | |
| MSTRG.20768 | si:dkey-78k11.9 (1 of many) | 30.96 | 27.30 | | 18.37 | | | 0.95 | | 3.87 | | 2.83 | 2.60 | | | 0.01 | up | | |
| MSTRG.18623 | acp6 | 17.11 | 20.35 | | 18.01 | | | 9.15 | | 6.57 | | 4.47 | 1.59 | | | 0.01 | up | | |
| MSTRG.3776 | ENSONIG00000010094 | 8.16 | 88.76 | | 13.56 | | | 3.39 | | 1.68 | | 1.89 | 3.97 | | | 0.01 | up | | |
| MSTRG.3659 | echs1 | 30.84 | 45.91 | | 41.47 | | | 13.36 | | 17.06 | | 16.67 | 1.36 | | | 0.01 | up | | |
| MSTRG.6178 | ENSONIG00000007222 | 0.41 | 1.01 | | 2.48 | | | 20.77 | | 23.47 | | 43.85 | -3.66 | | | 0.01 | down | | |
| MSTRG.2496 | rab11bb (1 of many) | 2.21 | 2.30 | | 3.07 | | | 15.95 | | 13.05 | | 8.69 | -1.79 | | | 0.01 | down | | |
| MSTRG.8994 | elf3 | 1.89 | 2.80 | | 1.70 | | | 6.96 | | 9.11 | | 10.80 | -1.65 | | | 0.01 | down | | |
| MSTRG.2342 | mibp | 3.53 | 1.05 | | 1.73 | | | 36.29 | | 17.30 | | 41.95 | -3.58 | | | 0.01 | down | | |
| MSTRG.8813 | enpp1 | 25.71 | 27.14 | | 21.79 | | | 80.06 | | 52.36 | | 66.46 | -1.22 | | | 0.01 | down | | |
| MSTRG.21630 | ENSONIG00000000250 | 1.13 | 3.54 | | 2.87 | | | 0.05 | | 0.06 | | 0.33 | 1.87 | | | 0.01 | up | | |
| MSTRG.23710 | mccc1 | 5.40 | 7.46 | | 5.82 | | | 1.43 | | 2.39 | | 1.80 | 1.36 | | | 0.01 | up | | |
| MSTRG.1156 | fdxr | 8.51 | 14.32 | | 9.22 | | | 4.25 | | 4.76 | | 4.35 | 1.25 | | | 0.01 | up | | |
| MSTRG.16918 | sec14l1 | 6.25 | 9.16 | | 9.97 | | | 1.83 | | 2.63 | | 2.61 | 1.55 | | | 0.01 | up | | |
| MSTRG.7515 | nudt4b | 0.32 | 0.24 | | 0.39 | | | 15.77 | | 8.88 | | 5.36 | -2.75 | | | 0.01 | down | | |
| MSTRG.1663 | zgc:77375 | 3.29 | 4.92 | | 3.33 | | | 2.46 | | 1.71 | | 1.29 | 1.02 | | | 0.01 | up | | |
| MSTRG.18621 | acp6 | 5.70 | 8.06 | | 6.07 | | | 2.84 | | 3.45 | | 2.65 | 1.02 | | | 0.01 | up | | |
| MSTRG.4780 | cog4 | 2.47 | 2.29 | | 3.50 | | | 7.06 | | 6.90 | | 6.40 | -1.06 | | | 0.01 | down | | |
| MSTRG.13274 | si:ch1073-90m23.1 | 2.06 | 1.79 | | 1.06 | | | 0 | | 0.06 | | 0.10 | 1.22 | | | 0.01 | up | | |
| MSTRG.4164 | aqp7 | 21.63 | 30.54 | | 22.97 | | | 2.86 | | 5.08 | | 6.07 | 2.19 | | | 0.01 | up | | |
| MSTRG.8749 | clu | 0.34 | 0.38 | | 0.85 | | | 3.90 | | 2.80 | | 2.60 | -1.31 | | | 0.01 | down | | |
| MSTRG.22661 | lgals9l5 | 33.66 | 46.68 | | 55.74 | | | 15.39 | | 16.71 | | 18.47 | 1.47 | | | 0.01 | up | | |
| MSTRG.15055 | slc1a4 | 0.63 | 0.43 | | 0.79 | | | 3.14 | | 9.29 | | 10.13 | -2.66 | | | 0.01 | down | | |
| MSTRG.21078 | NEBL | 17.32 | 20.70 | | 25.96 | | | 7.31 | | 8.46 | | 8.99 | 1.29 | | | 0.01 | up | | |
| MSTRG.23381 | nop10 | 18.40 | 12.93 | | 13.79 | | | 37.71 | | 30.73 | | 44.57 | -1.39 | | | 0.01 | down | | |
| MSTRG.5531 | nfil3-6 | 17.43 | 19.54 | | 28.14 | | | 8.15 | | 3.08 | | 4.07 | 2.28 | | | 0.01 | up | | |
| MSTRG.12538 | arhgap32b | 2.10 | 3.55 | | 2.50 | | | 13.08 | | 10.99 | | 7.44 | -1.36 | | | 0.01 | down | | |
| MSTRG.3955 | uroc1;MAPKAPK3 | 46.39 | 30.17 | | 30.45 | | | 7.29 | | 15.61 | | 11.53 | 1.17 | | | 0.01 | up | | |
| MSTRG.14109 | hmg20b | 2.01 | 2.96 | | 2.54 | | | 13.66 | | 14.86 | | 8.30 | -1.74 | | | 0.01 | down | | |
| MSTRG.10105 | gba2 | 4.99 | 9.84 | | 6.58 | | | 1.59 | | 1.64 | | 2.52 | 1.67 | | | 0.01 | up | | |
| MSTRG.22252 | ENSONIG00000017719 | 0.41 | 1.40 | | 1.19 | | | 0.15 | | 0 | | 0.23 | 1.06 | | | 0.01 | up | | |
| MSTRG.6303 | ST3GAL1 (1 of many) | 4.98 | 10.58 | | 5.75 | | | 3.27 | | 2.95 | | 2.25 | 1.36 | | | 0.01 | up | | |
| MSTRG.7100 | ENSONIG00000004864 | 1.27 | 1.11 | | 1.41 | | | 4.06 | | 9.23 | | 5.25 | -1.83 | | | 0.01 | down | | |
| MSTRG.13766 | mapkapk5 | 4.57 | 7.37 | | 5.42 | | | 22.16 | | 17.40 | | 29.50 | -1.61 | | | 0.01 | down | | |
| MSTRG.17035 | lcor | 5.46 | 2.03 | | 4.06 | | | 0.29 | | 0.25 | | 0.19 | 1.58 | | | 0.01 | up | | |
| MSTRG.14454 | ENSONIG00000013594 | 25.94 | 26.59 | | 27.57 | | | 6.84 | | 5.86 | | 10.73 | 1.70 | | | 0.01 | up | | |
| MSTRG.21023 | BPHL | 31.86 | 38.60 | | 35.87 | | | 7.23 | | 12.42 | | 11.48 | 1.63 | | | 0.01 | up | | |
| MSTRG.19929 | snrnp48 | 4.70 | 5.16 | | 4.94 | | | 15.47 | | 13.01 | | 9.94 | -1.08 | | | 0.01 | down | | |
| MSTRG.9256 | allc | 34.04 | 48.34 | | 35.71 | | | 5.55 | | 9.24 | | 10.47 | 2.10 | | | 0.01 | up | | |
| MSTRG.997 | dync2li1 | 0.98 | 4.39 | | 2.53 | | | 0 | | 0.12 | | 0.10 | 2.05 | | | 0.01 | up | | |
| MSTRG.24879 | ENSONIG00000013606 | 0.38 | 0.06 | | 0.06 | | | 0.97 | | 1.48 | | 0.78 | -1.00 | | | 0.01 | down | | |
| MSTRG.3831 | COX5B (1 of many) | 121.90 | 102.72 | | 143.45 | | | 298.33 | | 278.96 | | 241.10 | -1.18 | | | 0.01 | down | | |
| MSTRG.12093 | SAT2 (1 of many) | 8.43 | 15.37 | | 18.58 | | | 6.46 | | 0.97 | | 2.01 | 2.72 | | | 0.01 | up | | |
| MSTRG.6077 | bmi1a | 1.79 | 2.09 | | 1.94 | | | 9.91 | | 7.31 | | 5.40 | -1.34 | | | 0.01 | down | | |
| MSTRG.19514 | hpda | 141.14 | 87.92 | | 95.50 | | | 200.20 | | 230.65 | | 350.42 | -1.56 | | | 0.01 | down | | |
| MSTRG.10636 | nocta | 2.66 | 2.19 | | 1.66 | | | 11.21 | | 7.15 | | 11.80 | -1.76 | | | 0.01 | down | | |
| MSTRG.10937 | si:dkey-183j2.10 | 2.39 | 4.62 | | 2.55 | | | 0.60 | | 0.46 | | 0.97 | 1.51 | | | 0.01 | up | | |
| MSTRG.12022 | shbg | 556.57 | 579.99 | | 598.15 | | | 56.18 | | 143.45 | | 76.03 | 2.51 | | | 0.01 | up | | |
| ENSONIG00000002864 | ENSONIG00000002864 | 5.51 | 1.90 | | 3.70 | | | 0.24 | | 0.26 | | 0.43 | 1.40 | | | 0.01 | up | | |
| MSTRG.4163 | tpgs2 | 14.70 | 24.33 | | 12.19 | | | 4.67 | | 4.16 | | 5.43 | 1.81 | | | 0.01 | up | | |
| MSTRG.1793 | csrnp1b | 2.02 | 2.60 | | 3.44 | | | 20.78 | | 11.09 | | 10.81 | -1.70 | | | 0.01 | down | | |
| MSTRG.5428 | ENSONIG00000001091 | 2.99 | 1.07 | | 2.52 | | | 0 | | 0.13 | | 0 | 1.25 | | | 0.01 | up | | |
| MSTRG.10653 | PRF1 (1 of many) | 0.44 | 0.44 | | 0.01 | | | 3.53 | | 2.98 | | 5.58 | -1.96 | | | 0.01 | down | | |
| MSTRG.11354 | pdk2a | 22.26 | 24.64 | | 18.77 | | | 63.65 | | 77.12 | | 117.50 | -1.96 | | | 0.01 | down | | |
| MSTRG.15406 | ENSONIG00000015659 | 4.61 | 3.58 | | 1.99 | | | 0.08 | | 0.17 | | 0.18 | 1.78 | | | 0.01 | up | | |
| MSTRG.7578 | TIMP3 | 0.41 | 0.28 | | 0.24 | | | 2.75 | | 1.61 | | 2.80 | -1.31 | | | 0.01 | down | | |
| MSTRG.7694 | pttg1 | 0.53 | 0.38 | | 0.34 | | | 13.03 | | 5.35 | | 5.76 | -2.35 | | | 0.01 | down | | |
| MSTRG.9868 | pdk1 | 7.83 | 10.16 | | 10.09 | | | 2.96 | | 4.33 | | 3.03 | 1.26 | | | 0.01 | up | | |
| MSTRG.13281 | kel;zyx | 4.61 | 2.99 | | 2.74 | | | 42.59 | | 25.57 | | 19.05 | -2.66 | | | 0.01 | down | | |
| MSTRG.9663 | hipk2 | 34.80 | 31.18 | | 25.86 | | | 7.54 | | 5.65 | | 10.48 | 1.83 | | | 0.01 | up | | |
| MSTRG.2445 | lpl | 21.69 | 12.30 | | 12.16 | | | 148.98 | | 78.37 | | 186.30 | -3.16 | | | 0.01 | down | | |
| MSTRG.15329 | CARTPT (1 of many) | 6.85 | 12.33 | | 24.28 | | | 0.36 | | 0.19 | | 0.97 | 3.50 | | | 0.01 | up | | |
| MSTRG.6160 | ext1a | 2.60 | 3.83 | | 2.88 | | | 0.94 | | 1.35 | | 1.13 | 1.00 | | | 0.01 | up | | |
| MSTRG.10172 | pnp5a | 22.74 | 43.56 | | 66.30 | | | 12.68 | | 4.51 | | 9.07 | 2.75 | | | 0.01 | up | | |
| MSTRG.9867 | si:ch1073-219n12.1 | 7.62 | 18.82 | | 9.27 | | | 2.82 | | 3.26 | | 2.95 | 1.92 | | | 0.01 | up | | |
| MSTRG.8525 | slc1a3a | 56.30 | 45.18 | | 73.58 | | | 9.09 | | 16.41 | | 8.87 | 2.06 | | | 0.01 | up | | |
| MSTRG.22310 | PRPS2 | 43.08 | 29.99 | | 42.80 | | | 66.42 | | 67.87 | | 87.49 | -1.11 | | | 0.01 | down | | |
| MSTRG.16692 | ENSONIG00000018890 | 1.51 | 1.43 | | 2.42 | | | 0.37 | | 0.22 | | 0.24 | 1.15 | | | 0.01 | up | | |
| MSTRG.18261 | ENSONIG00000011213 | 1.86 | 5.75 | | 8.66 | | | 0 | | 0 | | 0 | 2.93 | | | 0.01 | up | | |
| MSTRG.3121 | kti12 | 1.69 | 0.89 | | 2.09 | | | 31.59 | | 18.63 | | 12.57 | -2.96 | | | 0.01 | down | | |
| MSTRG.2396 | si:zfos-1404b8.2;ENSONIG00000002113 | 50.74 | 64.01 | | 56.46 | | | 4.84 | | 10.65 | | 11.66 | 2.45 | | | 0.01 | up | | |
| MSTRG.17138 | mtp | 17.57 | 28.48 | | 27.16 | | | 1.40 | | 4.31 | | 2.59 | 2.77 | | | 0.01 | up | | |
| MSTRG.16335 | mpzl1l | 2.17 | 3.45 | | 2.67 | | | 14.25 | | 8.71 | | 7.23 | -1.19 | | | 0.01 | down | | |
| MSTRG.18108 | COL13A1 | 0.97 | 1.48 | | 0.65 | | | 0 | | 0 | | 0 | 1.09 | | | 0.01 | up | | |
| MSTRG.7550 | ABCD2 | 0.80 | 1.85 | | 0.67 | | | 0.38 | | 0 | | 0.05 | 1.15 | | | 0.01 | up | | |
| MSTRG.18243 | cadm1b | 8.12 | 11.66 | | 9.30 | | | 2.34 | | 3.20 | | 3.80 | 1.43 | | | 0.01 | up | | |
| MSTRG.11979 | SIK3 | 0.83 | 1.88 | | 1.27 | | | 0.09 | | 0.18 | | 0.29 | 1.12 | | | 0.01 | up | | |
| MSTRG.18002 | si:dkey-145p14.5 | 0.39 | 1.09 | | 1.25 | | | 5.49 | | 6.95 | | 4.51 | -1.65 | | | 0.01 | down | | |
| MSTRG.6191 | gapdhs | 3.49 | 1.85 | | 7.43 | | | 112.31 | | 54.28 | | 61.45 | -3.87 | | | 0.01 | down | | |
| MSTRG.15411 | ccnb2 | 0.07 | 0.03 | | 0.06 | | | 2.15 | | 2.32 | | 1.13 | -1.41 | | | 0.01 | down | | |
| MSTRG.5961 | abca1b | 2.51 | 1.94 | | 6.67 | | | 60.31 | | 46.03 | | 34.20 | -3.36 | | | 0.01 | down | | |
| MSTRG.13306 | tpi1b | 42.71 | 36.54 | | 68.77 | | | 216.13 | | 199.34 | | 170.09 | -2.01 | | | 0.01 | down | | |
| MSTRG.19897 | MAP4K1 | 0.80 | 3.14 | | 1.34 | | | 0.69 | | 0.40 | | 0.53 | 1.23 | | | 0.01 | up | | |
| MSTRG.9336 | slc47a1 | 18.13 | 25.86 | | 30.25 | | | 0.52 | | 2.99 | | 0.54 | 3.50 | | | 0.01 | up | | |
| MSTRG.3322 | hsp90aa1.1 | 0.01 | 0.10 | | 0.10 | | | 153.29 | | 32.24 | | 21.56 | -4.89 | | | 0.01 | down | | |
| MSTRG.12366 | ednraa | 1.40 | 1.36 | | 1.35 | | | 11.06 | | 5.80 | | 12.28 | -1.99 | | | 0.01 | down | | |
| MSTRG.2305 | CCDC50 | 1.58 | 1.49 | | 2.36 | | | 5.81 | | 4.55 | | 5.91 | -1.15 | | | 0.01 | down | | |
| MSTRG.10882 | RHOC (1 of many) | 18.05 | 17.81 | | 14.38 | | | 41.82 | | 110.74 | | 90.37 | -2.44 | | | 0.02 | down | | |
| MSTRG.2498 | fsd1 | 0.07 | 0.19 | | 0.14 | | | 1.32 | | 1.38 | | 2.35 | -1.22 | | | 0.02 | down | | |
| MSTRG.4973 | spns1 | 15.14 | 11.38 | | 15.65 | | | 57.20 | | 37.03 | | 41.55 | -1.58 | | | 0.02 | down | | |
| MSTRG.13271 | si:dkey-26c10.5 | 6.62 | 6.75 | | 10.05 | | | 1.76 | | 2.43 | | 2.44 | 1.39 | | | 0.02 | up | | |
| MSTRG.16062 | soul3 | 12.06 | 7.03 | | 7.44 | | | 74.72 | | 37.57 | | 51.23 | -2.49 | | | 0.02 | down | | |
| MSTRG.3462 | abcb11b | 24.16 | 49.93 | | 22.56 | | | 1.54 | | 3.39 | | 3.80 | 3.14 | | | 0.02 | up | | |
| MSTRG.12573 | snx3 | 17.45 | 15.59 | | 17.97 | | | 51.91 | | 37.18 | | 35.91 | -1.15 | | | 0.02 | down | | |
| MSTRG.3868 | ENSONIG00000010389 | 10.74 | 21.67 | | 15.43 | | | 9.27 | | 8.82 | | 6.56 | 1.18 | | | 0.02 | up | | |
| MSTRG.13615 | chrnb2b | 0.77 | 0.70 | | 0.32 | | | 5.66 | | 3.49 | | 3.29 | -1.55 | | | 0.02 | down | | |
| MSTRG.15001 | ints12 | 4.80 | 1.70 | | 2.38 | | | 18.01 | | 12.87 | | 12.14 | -2.24 | | | 0.02 | down | | |
| MSTRG.5086 | tbc1d17 | 2.45 | 2.61 | | 3.01 | | | 29.45 | | 12.89 | | 13.65 | -2.02 | | | 0.02 | down | | |
| MSTRG.7291 | amt | 22.98 | 35.09 | | 21.26 | | | 2.81 | | 6.18 | | 3.98 | 2.36 | | | 0.02 | up | | |
| MSTRG.16137 | prdx1 | 2.61 | 0.55 | | 3.46 | | | 58.50 | | 43.85 | | 23.95 | -3.98 | | | 0.02 | down | | |
| MSTRG.12713 | adgre5b.3 | 3.16 | 2.59 | | 2.94 | | | 21.94 | | 17.05 | | 10.45 | -2.04 | | | 0.02 | down | | |
| MSTRG.282 | ENSONIG00000002944 | 1.08 | 0.89 | | 1.07 | | | 2.40 | | 2.52 | | 3.77 | -1.02 | | | 0.02 | down | | |
| MSTRG.11698 | ENSONIG00000012487 | 20.73 | 17.60 | | 70.48 | | | 1.08 | | 0.34 | | 0.54 | 4.36 | | | 0.02 | up | | |
| MSTRG.9093 | dnpep | 4.35 | 5.53 | | 3.51 | | | 0.70 | | 1.35 | | 0.70 | 1.51 | | | 0.02 | up | | |
| MSTRG.1100 | cbr1 (1 of many) | 9.25 | 9.78 | | 7.56 | | | 4.79 | | 2.89 | | 3.95 | 1.16 | | | 0.02 | up | | |
| MSTRG.5602 | ENSONIG00000008347 | 20.57 | 31.22 | | 52.45 | | | 6.42 | | 1.27 | | 4.64 | 3.40 | | | 0.02 | up | | |
| MSTRG.13180 | cyp4t8 | 47.29 | 104.21 | | 75.82 | | | 11.86 | | 16.10 | | 19.90 | 2.36 | | | 0.02 | up | | |
| MSTRG.9459 | bco2a | 56.92 | 68.05 | | 59.40 | | | 13.17 | | 19.66 | | 23.28 | 1.62 | | | 0.02 | up | | |
| MSTRG.20197 | fam89a | 1.05 | 1.25 | | 1.08 | | | 10.97 | | 4.60 | | 7.59 | -1.71 | | | 0.02 | down | | |
| MSTRG.7982 | SMOC1 | 10.53 | 18.09 | | 10.66 | | | 3.09 | | 3.87 | | 4.37 | 1.67 | | | 0.02 | up | | |
| MSTRG.7254 | cfl1 | 1.32 | 1.99 | | 2.42 | | | 9.97 | | 5.24 | | 7.53 | -1.25 | | | 0.02 | down | | |
| MSTRG.16602 | tmem120a | 0.67 | 0.23 | | 0.59 | | | 2.14 | | 1.59 | | 1.90 | -1.02 | | | 0.02 | down | | |
| MSTRG.8998 | si:dkey-97a13.6 | 32.22 | 57.05 | | 45.28 | | | 5.30 | | 7.02 | | 11.58 | 2.49 | | | 0.02 | up | | |
| MSTRG.10706 | si:ch73-71d17.1 | 49.43 | 49.88 | | 68.99 | | | 10.01 | | 16.17 | | 7.79 | 2.18 | | | 0.02 | up | | |
| MSTRG.10421 | letm1 | 5.31 | 6.16 | | 6.78 | | | 3.93 | | 1.79 | | 2.46 | 1.20 | | | 0.02 | up | | |
| MSTRG.21278 | selj | 10.97 | 8.16 | | 10.47 | | | 3.95 | | 3.62 | | 2.54 | 1.26 | | | 0.02 | up | | |
| MSTRG.16531 | pisd | 1.12 | 1.81 | | 2.85 | | | 0.11 | | 0.13 | | 0 | 1.55 | | | 0.02 | up | | |
| MSTRG.9116 | aox6 | 10.42 | 20.13 | | 12.61 | | | 5.53 | | 6.31 | | 4.39 | 1.48 | | | 0.02 | up | | |
| MSTRG.14585 | shmt2 (1 of many) | 10.13 | 9.34 | | 9.49 | | | 1.58 | | 3.69 | | 1.89 | 1.51 | | | 0.02 | up | | |
| MSTRG.5393 | snap23.1 | 8.98 | 9.92 | | 10.24 | | | 46.69 | | 26.40 | | 26.19 | -1.38 | | | 0.02 | down | | |
| MSTRG.2920 | diabloa (1 of many) | 67.70 | 81.62 | | 56.13 | | | 1050.32 | | 337.33 | | 776.97 | -2.86 | | | 0.02 | down | | |
| MSTRG.17532 | acy1 | 32.34 | 25.91 | | 33.01 | | | 16.46 | | 12.70 | | 13.11 | 1.05 | | | 0.02 | up | | |
| MSTRG.2436 | cdc34a | 1.03 | 1.71 | | 0.71 | | | 27.01 | | 11.49 | | 9.32 | -2.44 | | | 0.02 | down | | |
| MSTRG.7941 | slc39a8 | 5.75 | 2.30 | | 3.90 | | | 7.23 | | 13.47 | | 22.95 | -2.14 | | | 0.02 | down | | |
| MSTRG.15693 | sept9a | 93.67 | 87.63 | | 105.61 | | | 7.99 | | 5.58 | | 18.30 | 3.18 | | | 0.02 | up | | |
| MSTRG.15647 | ankrd40 | 1.50 | 1.39 | | 2.34 | | | 9.24 | | 5.58 | | 7.41 | -1.49 | | | 0.02 | down | | |
| MSTRG.13641 | ca14 | 0.94 | 0.03 | | 0.08 | | | 53.67 | | 13.99 | | 20.93 | -4.22 | | | 0.02 | down | | |
| MSTRG.3299 | ctgfa | 3.74 | 2.20 | | 2.91 | | | 0 | | 0 | | 0.61 | 1.51 | | | 0.02 | up | | |
| MSTRG.2008 | ENSONIG00000021301 | 72.83 | 60.82 | | 68.45 | | | 13.86 | | 29.09 | | 19.81 | 1.41 | | | 0.02 | up | | |
| MSTRG.3677 | ENSONIG00000009676 | 5.68 | 6.14 | | 3.41 | | | 0.54 | | 0.68 | | 1.10 | 1.72 | | | 0.02 | up | | |
| MSTRG.20985 | zgc:85777 | 28.40 | 38.37 | | 21.88 | | | 9.34 | | 10.12 | | 10.70 | 1.51 | | | 0.02 | up | | |
| MSTRG.20392 | mibp2 | 151.75 | 127.07 | | 81.85 | | | 34.48 | | 25.34 | | 26.61 | 1.99 | | | 0.02 | up | | |
| MSTRG.12625 | hebp2 | 21.98 | 24.18 | | 16.71 | | | 10.47 | | 8.09 | | 9.94 | 1.15 | | | 0.02 | up | | |
| MSTRG.15649 | tob1a | 35.60 | 43.78 | | 31.80 | | | 89.83 | | 77.86 | | 108.93 | -1.20 | | | 0.02 | down | | |
| MSTRG.14212 | dolk | 30.89 | 41.40 | | 39.38 | | | 5.67 | | 9.60 | | 3.54 | 2.52 | | | 0.02 | up | | |
| MSTRG.290 | arid3b | 0.40 | 2.84 | | 1.37 | | | 0.30 | | 0.22 | | 0 | 1.49 | | | 0.02 | up | | |
| MSTRG.15282 | hax1 | 3.85 | 5.19 | | 5.50 | | | 25.60 | | 15.01 | | 13.12 | -1.34 | | | 0.02 | down | | |
| MSTRG.7442 | btg1 | 28.50 | 33.37 | | 24.53 | | | 155.65 | | 80.40 | | 143.86 | -1.82 | | | 0.02 | down | | |
| MSTRG.15470 | fam172a | 0.55 | 0.31 | | 0.46 | | | 2.67 | | 1.63 | | 1.90 | -1.04 | | | 0.02 | down | | |
| MSTRG.2028 | zgc:56622 | 23.59 | 24.85 | | 24.64 | | | 2.68 | | 7.30 | | 3.94 | 2.04 | | | 0.02 | up | | |
| MSTRG.23812 | ENSONIG00000010955;ENSONIG00000010953 | 5.37 | 4.47 | | 10.14 | | | 0.78 | | 0.61 | | 0.59 | 2.13 | | | 0.02 | up | | |
| MSTRG.2534 | echdc2 | 29.79 | 37.95 | | 45.29 | | | 3.44 | | 7.93 | | 7.23 | 2.35 | | | 0.02 | up | | |
| MSTRG.13914 | ehd4 | 0.16 | 2.25 | | 1.17 | | | 0.66 | | 0.26 | | 0 | 1.23 | | | 0.02 | up | | |
| MSTRG.11725 | kdm4b | 0.92 | 1.23 | | 1.39 | | | 2.91 | | 4.15 | | 3.20 | -1.03 | | | 0.02 | down | | |
| MSTRG.7589 | si:ch73-252i11.1 | 7.35 | 6.75 | | 10.45 | | | 2.74 | | 1.19 | | 1.00 | 2.02 | | | 0.02 | up | | |
| MSTRG.6416 | gpr22b | 0.02 | 0.01 | | 0 | | | 1.88 | | 1.48 | | 3.40 | -1.66 | | | 0.02 | down | | |
| MSTRG.2977 | arntl2 (1 of many) | 1.29 | 2.49 | | 2.78 | | | 10.97 | | 6.35 | | 6.19 | -1.09 | | | 0.02 | down | | |
| MSTRG.14282 | ENSONIG00000006637 | 3.02 | 3.26 | | 2.84 | | | 0.34 | | 0.65 | | 1.08 | 1.20 | | | 0.02 | up | | |
| MSTRG.3580 | spp2 | 727.46 | 508.76 | | 825.92 | | | 236.13 | | 309.44 | | 263.61 | 1.10 | | | 0.02 | up | | |
| MSTRG.6567 | pdcd6ip | 8.39 | 5.76 | | 8.56 | | | 22.19 | | 20.09 | | 16.01 | -1.34 | | | 0.02 | down | | |
| MSTRG.12450 | net1 | 1.35 | 1.89 | | 0.99 | | | 20.73 | | 14.46 | | 8.00 | -2.34 | | | 0.02 | down | | |
| MSTRG.14240 | si:dkey-27b3.2 | 4.13 | 4.73 | | 3.45 | | | 11.97 | | 10.77 | | 8.37 | -1.05 | | | 0.02 | down | | |
| MSTRG.1848 | METTL4 | 2.80 | 6.69 | | 2.14 | | | 0.12 | | 0.16 | | 0.20 | 2.23 | | | 0.02 | up | | |
| MSTRG.2147 | map1lc3b | 13.92 | 9.22 | | 15.21 | | | 80.94 | | 43.90 | | 62.22 | -2.16 | | | 0.02 | down | | |
| MSTRG.2307 | si:ch211-232d19.4 | 1.69 | 1.94 | | 1.06 | | | 4.44 | | 4.74 | | 4.11 | -1.08 | | | 0.02 | down | | |
| MSTRG.10395 | loxl3b | 1.47 | 1.43 | | 0.97 | | | 3.44 | | 3.19 | | 4.53 | -1.06 | | | 0.02 | down | | |
| MSTRG.17713 | ENSONIG00000020643 | 3.12 | 1.16 | | 1.64 | | | 3.70 | | 3.69 | | 4.59 | -1.10 | | | 0.02 | down | | |
| MSTRG.4621 | kank2 | 1.43 | 1.68 | | 1.42 | | | 13.07 | | 5.74 | | 11.31 | -1.83 | | | 0.02 | down | | |
| MSTRG.3133 | lactb | 11.13 | 8.58 | | 7.89 | | | 2.56 | | 3.34 | | 4.36 | 1.01 | | | 0.02 | up | | |
| MSTRG.15473 | ENSONIG00000011824 | 2.91 | 6.31 | | 8.14 | | | 0.31 | | 0.38 | | 0.86 | 2.34 | | | 0.02 | up | | |
| MSTRG.6453 | dnm1l | 2.59 | 2.51 | | 3.66 | | | 6.38 | | 7.97 | | 6.30 | -1.06 | | | 0.02 | down | | |
| MSTRG.13772 | ostf1 | 10.68 | 17.88 | | 8.54 | | | 45.76 | | 48.89 | | 47.21 | -1.74 | | | 0.02 | down | | |
| MSTRG.10679 | RNF38 (1 of many) | 0.31 | 1.42 | | 1.06 | | | 8.68 | | 3.26 | | 3.75 | -1.08 | | | 0.02 | down | | |
| MSTRG.4225 | gdpd5b | 6.24 | 4.19 | | 3.54 | | | 6.84 | | 10.42 | | 7.99 | -1.01 | | | 0.02 | down | | |
| MSTRG.7868 | SPTLC2 (1 of many) | 0.85 | 1.75 | | 1.18 | | | 7.63 | | 5.72 | | 3.81 | -1.25 | | | 0.02 | down | | |
| MSTRG.25141 | ENSONIG00000008142 | 0.89 | 1.62 | | 0.61 | | | 0.21 | | 0 | | 0 | 1.11 | | | 0.02 | up | | |
| MSTRG.8001 | zfand5a | 3.16 | 2.80 | | 9.65 | | | 162.60 | | 74.77 | | 69.33 | -3.83 | | | 0.02 | down | | |
| MSTRG.20778 | r3hdml | 6.34 | 11.46 | | 7.53 | | | 1.18 | | 2.08 | | 2.19 | 1.83 | | | 0.02 | up | | |
| MSTRG.12814 | cyth1a | 9.02 | 9.50 | | 8.91 | | | 41.73 | | 25.64 | | 23.09 | -1.38 | | | 0.02 | down | | |
| MSTRG.24815 | LRRC30 (1 of many) | 0.99 | 1.33 | | 0.98 | | | 4.74 | | 2.99 | | 5.17 | -1.17 | | | 0.02 | down | | |
| MSTRG.14343 | znf385b | 0.25 | 0.66 | | 1.05 | | | 8.28 | | 3.76 | | 7.60 | -1.85 | | | 0.02 | down | | |
| MSTRG.10510 | PLPPR2 (1 of many) | 0.18 | 0 | | 0.40 | | | 0.98 | | 2.87 | | 2.19 | -1.58 | | | 0.02 | down | | |
| MSTRG.20574 | spsb4a | 3.08 | 8.23 | | 3.51 | | | 42.54 | | 21.70 | | 17.77 | -1.66 | | | 0.02 | down | | |
| MSTRG.15684 | sept9a | 3.53 | 5.74 | | 6.61 | | | 25.19 | | 18.51 | | 34.01 | -1.86 | | | 0.02 | down | | |
| MSTRG.9507 | fhl1b | 0.78 | 1.07 | | 1.49 | | | 4.85 | | 5.16 | | 3.40 | -1.27 | | | 0.02 | down | | |
| MSTRG.22896 | ENSONIG00000015044 | 0.95 | 3.97 | | 1.09 | | | 0.54 | | 0.14 | | 0.14 | 1.62 | | | 0.02 | up | | |
| MSTRG.8213 | atic | 19.34 | 17.07 | | 30.02 | | | 4.04 | | 5.93 | | 3.34 | 1.98 | | | 0.02 | up | | |
| MSTRG.21542 | phldb1b | 0.04 | 0.08 | | 0.05 | | | 4.82 | | 3.11 | | 1.83 | -1.77 | | | 0.02 | down | | |
| MSTRG.19009 | rbm17 | 7.23 | 9.38 | | 11.17 | | | 20.09 | | 23.06 | | 21.32 | -1.07 | | | 0.02 | down | | |
| MSTRG.239 | slc12a4 | 10.15 | 22.00 | | 10.30 | | | 6.97 | | 5.61 | | 5.29 | 1.42 | | | 0.02 | up | | |
| MSTRG.9847 | cycsb (1 of many) | 17.87 | 20.49 | | 18.74 | | | 276.79 | | 160.71 | | 89.09 | -2.69 | | | 0.02 | down | | |
| MSTRG.19675 | ENSONIG00000008188 | 0.39 | 0.86 | | 0.32 | | | 21.58 | | 9.51 | | 6.17 | -2.56 | | | 0.02 | down | | |
| MSTRG.22536 | ENSONIG00000021455 | 0.94 | 2.23 | | 2.14 | | | 0.60 | | 0.41 | | 0.72 | 1.04 | | | 0.02 | up | | |
| MSTRG.14408 | limk2 | 3.73 | 6.67 | | 5.71 | | | 3.46 | | 0.76 | | 2.17 | 1.58 | | | 0.02 | up | | |
| MSTRG.10732 | itih2 | 650.17 | 730.87 | | 574.71 | | | 165.80 | | 277.17 | | 229.56 | 1.44 | | | 0.02 | up | | |
| MSTRG.17157 | suclg1 | 68.02 | 54.42 | | 54.54 | | | 10.12 | | 23.62 | | 16.55 | 1.47 | | | 0.02 | up | | |
| MSTRG.10845 | acvr1bb | 0.95 | 1.06 | | 0.57 | | | 3.99 | | 2.60 | | 3.89 | -1.19 | | | 0.02 | down | | |
| MSTRG.13599 | rorc | 0.83 | 2.34 | | 1.26 | | | 0.15 | | 0.21 | | 0.30 | 1.22 | | | 0.02 | up | | |
| MSTRG.2940 | zgc:123278 | 17.53 | 14.19 | | 23.69 | | | 1.47 | | 4.15 | | 1.46 | 2.34 | | | 0.02 | up | | |
| MSTRG.6161 | CSMD3 (1 of many) | 4.62 | 9.28 | | 3.94 | | | 1.33 | | 0.62 | | 1.64 | 1.94 | | | 0.02 | up | | |
| MSTRG.27348 | ENSONIG00000017776 | 0.63 | 1.81 | | 1.09 | | | 0.07 | | 0.20 | | 0.14 | 1.11 | | | 0.02 | up | | |
| MSTRG.10761 | mdm2 | 3.09 | 3.24 | | 3.04 | | | 23.99 | | 13.38 | | 10.93 | -1.75 | | | 0.02 | down | | |
| MSTRG.18103 | RGS3 | 5.78 | 5.61 | | 11.27 | | | 34.71 | | 32.77 | | 51.01 | -2.29 | | | 0.02 | down | | |
| MSTRG.8729 | rab10 | 7.36 | 7.23 | | 6.88 | | | 12.65 | | 25.48 | | 23.19 | -1.57 | | | 0.02 | down | | |
| MSTRG.9957 | NCOA2 (1 of many) | 0.42 | 1.10 | | 0.23 | | | 20.24 | | 5.66 | | 13.10 | -2.54 | | | 0.02 | down | | |
| MSTRG.19590 | SNORD53_SNORD92 | 1.12 | 1.93 | | 1.11 | | | 4.41 | | 5.84 | | 4.28 | -1.22 | | | 0.02 | down | | |
| MSTRG.26782 | rgn | 59.40 | 55.29 | | 77.27 | | | 15.40 | | 26.24 | | 20.51 | 1.42 | | | 0.02 | up | | |
| MSTRG.20384 | stom (1 of many) | 3.37 | 4.02 | | 3.46 | | | 14.29 | | 10.89 | | 8.07 | -1.18 | | | 0.02 | down | | |
| MSTRG.7970 | golga5 | 3.43 | 3.16 | | 3.83 | | | 12.62 | | 9.31 | | 7.82 | -1.17 | | | 0.02 | down | | |
| MSTRG.4562 | ezh1 | 1.03 | 2.49 | | 1.11 | | | 10.36 | | 8.25 | | 5.43 | -1.49 | | | 0.02 | down | | |
| MSTRG.14429 | mvk | 1.24 | 2.70 | | 2.80 | | | 0.43 | | 0.60 | | 0.45 | 1.29 | | | 0.02 | up | | |
| MSTRG.23252 | sqstm1 | 21.98 | 15.78 | | 27.71 | | | 225.28 | | 150.89 | | 96.28 | -2.68 | | | 0.02 | down | | |
| MSTRG.6653 | gmpr | 6.68 | 3.17 | | 5.23 | | | 6.72 | | 13.15 | | 7.54 | -1.17 | | | 0.02 | down | | |
| MSTRG.13448 | nr1h3 | 16.22 | 18.89 | | 13.70 | | | 7.91 | | 5.87 | | 3.97 | 1.53 | | | 0.02 | up | | |
| MSTRG.17850 | ENSONIG00000007916 | 1.08 | 5.07 | | 3.71 | | | 1.03 | | 0.86 | | 0.37 | 1.72 | | | 0.02 | up | | |
| MSTRG.5270 | ENSONIG00000020363 | 2.99 | 4.51 | | 9.88 | | | 0.03 | | 0.03 | | 0.20 | 2.68 | | | 0.02 | up | | |
| MSTRG.11779 | ENSONIG00000012749 | 25.97 | 107.07 | | 82.19 | | | 4.23 | | 0.33 | | 8.18 | 4.88 | | | 0.02 | up | | |
| MSTRG.20310 | slc22a2 | 12.25 | 15.14 | | 11.52 | | | 1.66 | | 2.67 | | 4.08 | 1.86 | | | 0.02 | up | | |
| MSTRG.2492 | gpx4a | 1337.67 | 1416.04 | | 1150.48 | | | 420.94 | | 494.84 | | 649.84 | 1.27 | | | 0.02 | up | | |
| MSTRG.5307 | galnt16 | 34.00 | 36.98 | | 27.89 | | | 3.77 | | 8.65 | | 8.10 | 1.96 | | | 0.02 | up | | |
| MSTRG.1851 | METTL4 | 8.23 | 4.70 | | 11.37 | | | 0.24 | | 0.69 | | 1.21 | 2.05 | | | 0.02 | up | | |
| MSTRG.14555 | acsl1b | 26.88 | 46.37 | | 28.31 | | | 5.24 | | 9.39 | | 7.89 | 2.08 | | | 0.02 | up | | |
| MSTRG.19076 | mospd1 | 6.40 | 4.15 | | 5.17 | | | 44.46 | | 20.49 | | 27.55 | -2.22 | | | 0.02 | down | | |
| MSTRG.11048 | ergic3 | 140.70 | 68.98 | | 68.17 | | | 127.20 | | 162.95 | | 150.28 | -1.14 | | | 0.02 | down | | |
| MSTRG.4800 | lipca | 234.46 | 222.61 | | 261.95 | | | 80.34 | | 128.04 | | 104.01 | 1.06 | | | 0.02 | up | | |
| MSTRG.1655 | irf10 | 7.60 | 9.37 | | 23.45 | | | 0.82 | | 0.84 | | 0.86 | 2.93 | | | 0.02 | up | | |
| MSTRG.22932 | zgc:110239 | 11.37 | 12.52 | | 16.81 | | | 6.54 | | 6.04 | | 6.86 | 1.01 | | | 0.02 | up | | |
| MSTRG.13695 | serpind1 | 1456.60 | 1293.29 | | 838.22 | | | 292.70 | | 409.59 | | 321.15 | 1.62 | | | 0.02 | up | | |
| MSTRG.15059 | RNF122 (1 of many) | 4.41 | 9.44 | | 5.12 | | | 5.03 | | 2.24 | | 3.80 | 1.09 | | | 0.02 | up | | |
| MSTRG.18256 | c18h3orf33 | 3.64 | 4.57 | | 5.24 | | | 1.79 | | 2.11 | | 1.28 | 1.09 | | | 0.02 | up | | |
| MSTRG.24105 | ENSONIG00000011076 | 21.91 | 26.79 | | 10.69 | | | 0.31 | | 1.90 | | 1.09 | 3.09 | | | 0.02 | up | | |
| MSTRG.4992 | ubald2 | 2.17 | 1.74 | | 2.25 | | | 11.66 | | 7.73 | | 6.29 | -1.52 | | | 0.02 | down | | |
| MSTRG.14445 | acacb | 3.11 | 5.52 | | 3.00 | | | 0.82 | | 1.17 | | 1.03 | 1.39 | | | 0.02 | up | | |
| MSTRG.8129 | fth1a | 3882.12 | 4194.89 | | 2573.87 | | | 13111.86 | | 12782.72 | | 9417.54 | -1.69 | | | 0.02 | down | | |
| MSTRG.19523 | ggnbp2 | 3.95 | 4.06 | | 4.15 | | | 15.04 | | 8.53 | | 12.09 | -1.16 | | | 0.02 | down | | |
| MSTRG.13161 | ENSONIG00000016148 | 2.17 | 5.40 | | 2.45 | | | 0.18 | | 0 | | 0.70 | 2.01 | | | 0.02 | up | | |
| MSTRG.16931 | ZCCHC11 | 5.33 | 14.99 | | 21.91 | | | 2.77 | | 1.95 | | 2.20 | 2.53 | | | 0.02 | up | | |
| MSTRG.3001 | ENSONIG00000014729 | 40.19 | 37.81 | | 49.79 | | | 8.22 | | 15.19 | | 7.64 | 1.84 | | | 0.02 | up | | |
| MSTRG.17383 | hacl1 | 24.38 | 36.09 | | 25.94 | | | 8.92 | | 12.53 | | 11.67 | 1.36 | | | 0.02 | up | | |
| MSTRG.6124 | si:ch211-93i7.4 | 25.36 | 21.92 | | 26.17 | | | 4.83 | | 8.50 | | 9.83 | 1.35 | | | 0.02 | up | | |
| MSTRG.5606 | rrp7a | 7.77 | 7.04 | | 7.81 | | | 28.29 | | 19.77 | | 16.90 | -1.28 | | | 0.02 | down | | |
| MSTRG.23989 | ENSONIG00000003847;ENSONIG00000003852 | 40.89 | 70.98 | | 31.56 | | | 6.02 | | 7.81 | | 10.59 | 2.47 | | | 0.02 | up | | |
| MSTRG.7329 | SHMT2 (1 of many) | 29.03 | 61.39 | | 38.06 | | | 1.73 | | 6.30 | | 3.33 | 3.27 | | | 0.02 | up | | |
| MSTRG.5760 | ENSONIG00000004273 | 14.36 | 39.96 | | 91.00 | | | 3.52 | | 1.00 | | 3.51 | 4.18 | | | 0.02 | up | | |
| MSTRG.5803 | ube2f | 1.18 | 1.36 | | 1.06 | | | 11.81 | | 7.53 | | 4.99 | -1.77 | | | 0.02 | down | | |
| MSTRG.18425 | chtopb | 18.96 | 14.14 | | 19.73 | | | 49.25 | | 42.38 | | 34.87 | -1.26 | | | 0.02 | down | | |
| MSTRG.866 | FYTTD1 | 4.33 | 5.47 | | 5.85 | | | 23.65 | | 20.47 | | 12.91 | -1.47 | | | 0.02 | down | | |
| MSTRG.7488 | napepld | 3.65 | 5.27 | | 4.68 | | | 1.23 | | 2.03 | | 1.59 | 1.12 | | | 0.02 | up | | |
| MSTRG.23105 | ndufb9 | 41.12 | 41.29 | | 57.76 | | | 15.21 | | 20.49 | | 19.23 | 1.22 | | | 0.02 | up | | |
| MSTRG.3312 | dll4 | 2.28 | 2.16 | | 3.63 | | | 8.60 | | 7.63 | | 6.87 | -1.21 | | | 0.02 | down | | |
| MSTRG.5044 | im:6904045 | 68.11 | 81.70 | | 74.44 | | | 20.97 | | 29.98 | | 34.07 | 1.34 | | | 0.02 | up | | |
| MSTRG.2003 | tmed6 | 0.35 | 0.27 | | 0.48 | | | 1.39 | | 2.01 | | 3.07 | -1.31 | | | 0.02 | down | | |
| MSTRG.3305 | rhov | 0.88 | 1.09 | | 2.18 | | | 8.44 | | 6.92 | | 5.50 | -1.61 | | | 0.02 | down | | |
| MSTRG.16904 | SLC9A3R1 (1 of many) | 3.04 | 3.09 | | 4.29 | | | 35.33 | | 14.31 | | 18.48 | -2.02 | | | 0.02 | down | | |
| MSTRG.1640 | SNORD59 | 1.27 | 0.44 | | 0.66 | | | 4.00 | | 5.24 | | 2.47 | -1.66 | | | 0.02 | down | | |
| MSTRG.654 | zgc:153615 | 0.08 | 0.03 | | 0.17 | | | 4.25 | | 1.76 | | 2.24 | -1.54 | | | 0.02 | down | | |
| MSTRG.14211 | phyhd1 | 8.14 | 14.15 | | 8.97 | | | 1.09 | | 2.73 | | 1.22 | 2.17 | | | 0.02 | up | | |
| MSTRG.21536 | ENSONIG00000014771 | 11.61 | 11.97 | | 18.06 | | | 80.14 | | 52.79 | | 43.42 | -1.79 | | | 0.02 | down | | |
| MSTRG.1821 | mnx1 | 0 | 0 | | 0 | | | 1.36 | | 1.93 | | 0.82 | -1.25 | | | 0.02 | down | | |
| MSTRG.3199 | cracr2b | 3.95 | 8.27 | | 7.54 | | | 2.26 | | 2.43 | | 2.74 | 1.33 | | | 0.02 | up | | |
| MSTRG.10470 | serhl (1 of many) | 16.42 | 16.41 | | 18.77 | | | 1.22 | | 3.98 | | 3.72 | 2.01 | | | 0.02 | up | | |
| MSTRG.7051 | rufy3 | 5.51 | 6.94 | | 6.49 | | | 28.65 | | 15.44 | | 15.64 | -1.17 | | | 0.02 | down | | |
| MSTRG.7454 | ENSONIG00000020520 | 9.97 | 14.53 | | 8.71 | | | 2.34 | | 1.57 | | 3.84 | 1.94 | | | 0.02 | up | | |
| MSTRG.1965 | tnfsf10 (1 of many) | 0.18 | 1.74 | | 1.51 | | | 0.38 | | 0.14 | | 0.10 | 1.20 | | | 0.02 | up | | |
| MSTRG.13370 | cdkn1d | 1.39 | 1.86 | | 1.11 | | | 4.48 | | 3.93 | | 5.92 | -1.16 | | | 0.02 | down | | |
| MSTRG.20961 | ENSONIG00000003942 | 1798.28 | 1188.53 | | 1446.07 | | | 363.29 | | 519.64 | | 696.01 | 1.18 | | | 0.02 | up | | |
| MSTRG.26411 | sde2 | 8.15 | 8.83 | | 12.36 | | | 30.69 | | 21.74 | | 24.55 | -1.16 | | | 0.02 | down | | |
| MSTRG.5144 | cdk5rap1 | 0.81 | 0.73 | | 1.47 | | | 3.04 | | 3.15 | | 2.86 | -1.03 | | | 0.02 | down | | |
| MSTRG.18465 | erap1b | 4.25 | 3.93 | | 7.49 | | | 0.80 | | 0.47 | | 1.08 | 1.80 | | | 0.02 | up | | |
| MSTRG.6326 | ldlrap1a | 17.79 | 22.92 | | 12.69 | | | 3.90 | | 4.65 | | 5.81 | 1.69 | | | 0.02 | up | | |
| MSTRG.25111 | arhgef10lb | 3.22 | 2.09 | | 3.40 | | | 4.32 | | 6.23 | | 7.82 | -1.11 | | | 0.02 | down | | |
| MSTRG.8817 | sytl3 | 0.71 | 0.72 | | 0.69 | | | 1.68 | | 2.16 | | 3.06 | -1.01 | | | 0.02 | down | | |
| MSTRG.13273 | si:dkey-40m6.14 | 79.36 | 130.79 | | 112.48 | | | 8.41 | | 21.30 | | 17.61 | 2.69 | | | 0.02 | up | | |
| MSTRG.11212 | adora2aa | 0.08 | 0.17 | | 0.41 | | | 2.40 | | 1.23 | | 1.76 | -1.01 | | | 0.02 | down | | |
| MSTRG.6930 | antxr2b | 9.25 | 12.56 | | 11.92 | | | 4.46 | | 4.11 | | 6.36 | 1.16 | | | 0.02 | up | | |
| MSTRG.22923 | mfsd2ab | 0.78 | 0.39 | | 1.11 | | | 7.01 | | 5.98 | | 3.55 | -1.87 | | | 0.02 | down | | |
| MSTRG.12907 | pfkpa | 0.57 | 0.82 | | 1.00 | | | 15.46 | | 5.93 | | 5.85 | -1.98 | | | 0.02 | down | | |
| MSTRG.2508 | CYP2J2 (1 of many) | 134.91 | 168.41 | | 137.43 | | | 48.61 | | 52.02 | | 75.56 | 1.36 | | | 0.02 | up | | |
| MSTRG.8328 | rlim | 3.58 | 2.04 | | 4.11 | | | 10.14 | | 10.00 | | 7.73 | -1.44 | | | 0.02 | down | | |
| MSTRG.16204 | ENSONIG00000007879 | 2.41 | 1.70 | | 3.98 | | | 0.15 | | 0.16 | | 0.14 | 1.55 | | | 0.02 | up | | |
| MSTRG.1457 | pfdn4 | 75.50 | 82.97 | | 42.64 | | | 237.14 | | 226.17 | | 255.75 | -1.86 | | | 0.02 | down | | |
| MSTRG.21553 | abcd1 | 3.90 | 6.48 | | 3.21 | | | 1.57 | | 1.54 | | 1.36 | 1.29 | | | 0.02 | up | | |
| MSTRG.15083 | rhoq | 0.38 | 0.62 | | 0.29 | | | 3.59 | | 1.68 | | 2.72 | -1.11 | | | 0.02 | down | | |
| MSTRG.14271 | smad7 | 2.68 | 2.63 | | 4.45 | | | 7.64 | | 10.66 | | 11.22 | -1.46 | | | 0.02 | down | | |
| MSTRG.11128 | tmem59 | 17.47 | 21.41 | | 19.50 | | | 6.89 | | 9.14 | | 10.26 | 1.06 | | | 0.02 | up | | |
| MSTRG.22590 | ENSONIG00000012703 | 17.74 | 14.47 | | 24.50 | | | 6.36 | | 7.13 | | 5.99 | 1.29 | | | 0.02 | up | | |
| MSTRG.12367 | tmem184c | 6.28 | 8.57 | | 9.59 | | | 3.06 | | 3.38 | | 4.12 | 1.09 | | | 0.02 | up | | |
| MSTRG.14286 | rcbtb1 (1 of many) | 8.63 | 6.83 | | 5.27 | | | 16.74 | | 15.64 | | 23.69 | -1.44 | | | 0.02 | down | | |
| MSTRG.823 | gtf2b | 6.10 | 5.66 | | 7.29 | | | 34.27 | | 17.65 | | 21.32 | -1.56 | | | 0.02 | down | | |
| MSTRG.9117 | kynu | 30.19 | 41.22 | | 44.06 | | | 7.71 | | 13.08 | | 12.49 | 1.70 | | | 0.02 | up | | |
| MSTRG.891 | OTOR | 0.73 | 0.58 | | 0.24 | | | 3.32 | | 3.33 | | 2.05 | -1.37 | | | 0.02 | down | | |
| MSTRG.10357 | AKT1 | 0.36 | 0.34 | | 0.72 | | | 2.87 | | 1.92 | | 3.41 | -1.28 | | | 0.02 | down | | |
| MSTRG.26856 | pomk | 0.14 | 0.78 | | 0 | | | 3.76 | | 3.59 | | 6.43 | -1.96 | | | 0.02 | down | | |
| MSTRG.18883 | sh3bgrl2 | 0.72 | 1.11 | | 0.91 | | | 21.46 | | 6.88 | | 21.80 | -2.71 | | | 0.02 | down | | |
| MSTRG.1721 | CERS5 (1 of many) | 12.41 | 12.47 | | 13.07 | | | 46.25 | | 25.98 | | 32.93 | -1.18 | | | 0.02 | down | | |
| MSTRG.3934 | PTGIS (1 of many) | 0 | 0 | | 0.19 | | | 12.27 | | 4.14 | | 4.06 | -2.38 | | | 0.02 | down | | |
| MSTRG.8800 | tmem151ba | 0.71 | 1.46 | | 0.49 | | | 3.60 | | 3.33 | | 4.04 | -1.18 | | | 0.02 | down | | |
| MSTRG.810 | fam69aa | 2.53 | 3.96 | | 2.70 | | | 1.03 | | 1.45 | | 1.00 | 1.00 | | | 0.02 | up | | |
| MSTRG.5830 | si:ch211-219a4.3 | 4.41 | 4.66 | | 5.05 | | | 0.86 | | 0.65 | | 1.83 | 1.48 | | | 0.02 | up | | |
| MSTRG.9659 | ttc38 | 18.36 | 25.70 | | 20.47 | | | 4.21 | | 6.71 | | 7.88 | 1.64 | | | 0.02 | up | | |
| MSTRG.7697 | nkap | 4.79 | 4.84 | | 5.32 | | | 21.61 | | 12.40 | | 12.74 | -1.24 | | | 0.02 | down | | |
| MSTRG.6554 | yrk | 3.17 | 3.03 | | 4.76 | | | 22.55 | | 12.44 | | 13.07 | -1.65 | | | 0.02 | down | | |
| MSTRG.23961 | ENSONIG00000017456 | 2.24 | 5.26 | | 5.73 | | | 1.22 | | 1.06 | | 1.49 | 1.51 | | | 0.02 | up | | |
| MSTRG.1692 | ENSONIG00000017246 | 7.82 | 3.13 | | 7.16 | | | 0.33 | | 0.92 | | 1.43 | 1.38 | | | 0.02 | up | | |
| MSTRG.10012 | pnp5b | 0.28 | 0.16 | | 0.51 | | | 21.10 | | 5.34 | | 10.50 | -2.82 | | | 0.02 | down | | |
| MSTRG.2944 | irf7 | 5.18 | 7.91 | | 9.61 | | | 4.68 | | 1.92 | | 3.37 | 1.37 | | | 0.02 | up | | |
| MSTRG.3311 | chac1 | 38.78 | 21.74 | | 36.91 | | | 102.70 | | 82.15 | | 77.76 | -1.59 | | | 0.02 | down | | |
| MSTRG.7497 | adipor2 | 48.81 | 79.67 | | 44.41 | | | 34.40 | | 23.00 | | 31.25 | 1.22 | | | 0.02 | up | | |
| MSTRG.12658 | ENSONIG00000013592 | 1.36 | 1.03 | | 2.44 | | | 5.99 | | 26.41 | | 18.12 | -3.09 | | | 0.02 | down | | |
| MSTRG.17572 | slkb | 1.44 | 1.33 | | 1.51 | | | 6.04 | | 3.49 | | 4.09 | -1.04 | | | 0.02 | down | | |
| MSTRG.21014 | igfbp1a | 0.56 | 1.02 | | 1.20 | | | 48.33 | | 9.56 | | 18.31 | -2.90 | | | 0.02 | down | | |
| MSTRG.13665 | ndufa3 | 1.27 | 0.81 | | 1.63 | | | 4.88 | | 3.73 | | 3.52 | -1.19 | | | 0.02 | down | | |
| MSTRG.16386 | ENSONIG00000000164 | 0.06 | 0.25 | | 0.74 | | | 2.86 | | 3.30 | | 2.22 | -1.43 | | | 0.02 | down | | |
| MSTRG.2865 | hic1 | 1.23 | 0 | | 1.33 | | | 8.75 | | 5.48 | | 7.19 | -2.43 | | | 0.02 | down | | |
| MSTRG.10142 | ccs | 6.19 | 12.01 | | 4.88 | | | 36.55 | | 26.83 | | 27.74 | -1.58 | | | 0.02 | down | | |
| MSTRG.1868 | smarcd3b | 0.94 | 0.94 | | 0.13 | | | 6.72 | | 4.03 | | 5.74 | -1.88 | | | 0.02 | down | | |
| MSTRG.16518 | slc31a1 | 28.76 | 34.06 | | 50.29 | | | 181.91 | | 154.86 | | 107.01 | -1.76 | | | 0.02 | down | | |
| MSTRG.11371 | g6pca.2 | 2.62 | 2.02 | | 2.80 | | | 4.27 | | 6.06 | | 8.27 | -1.22 | | | 0.02 | down | | |
| MSTRG.14587 | myg1 | 5.41 | 8.14 | | 6.83 | | | 1.83 | | 2.37 | | 3.11 | 1.26 | | | 0.02 | up | | |
| MSTRG.9905 | BAZ2B (1 of many) | 1.49 | 0.85 | | 1.65 | | | 4.06 | | 4.98 | | 3.01 | -1.25 | | | 0.02 | down | | |
| MSTRG.20908 | ENSONIG00000011682 | 2.46 | 4.64 | | 3.22 | | | 0.78 | | 0.58 | | 1.47 | 1.40 | | | 0.02 | up | | |
| MSTRG.640 | agxtb;thap4 | 76.59 | 51.03 | | 29.38 | | | 466.61 | | 304.20 | | 263.45 | -2.80 | | | 0.02 | down | | |
| MSTRG.13320 | si:ch73-86n18.1 (1 of many) | 5.60 | 4.19 | | 16.68 | | | 64.40 | | 77.69 | | 108.13 | -3.45 | | | 0.02 | down | | |
| MSTRG.22315 | gemin8 | 2.10 | 1.75 | | 2.94 | | | 5.40 | | 5.77 | | 4.70 | -1.00 | | | 0.02 | down | | |
| MSTRG.16662 | nt5c2b | 18.17 | 8.79 | | 13.18 | | | 67.51 | | 66.56 | | 35.81 | -2.23 | | | 0.02 | down | | |
| MSTRG.25006 | HIST2H2AB (1 of many) | 2.01 | 1.31 | | 1.16 | | | 7.75 | | 5.19 | | 4.70 | -1.45 | | | 0.02 | down | | |
| MSTRG.1474 | pink1 | 14.56 | 19.28 | | 13.94 | | | 4.00 | | 6.01 | | 6.49 | 1.37 | | | 0.02 | up | | |
| MSTRG.11546 | ptp4a2b | 4.69 | 4.09 | | 6.82 | | | 13.02 | | 15.20 | | 11.48 | -1.27 | | | 0.02 | down | | |
| MSTRG.7421 | kcnc4 | 0.21 | 0.31 | | 0.40 | | | 3.83 | | 2.01 | | 1.79 | -1.18 | | | 0.02 | down | | |
| MSTRG.13319 | si:ch73-86n18.1 (1 of many) | 1260.96 | 744.98 | | 2467.40 | | | 4958.96 | | 9835.35 | | 9660.50 | -2.99 | | | 0.02 | down | | |
| MSTRG.3560 | APP (1 of many) | 2.21 | 3.45 | | 2.28 | | | 5.91 | | 7.15 | | 7.83 | -1.07 | | | 0.02 | down | | |
| MSTRG.23467 | ABHD5 (1 of many) | 2.76 | 2.25 | | 3.34 | | | 20.01 | | 11.05 | | 10.11 | -1.76 | | | 0.02 | down | | |
| MSTRG.16158 | fggy | 13.02 | 12.62 | | 17.14 | | | 4.58 | | 6.30 | | 3.72 | 1.34 | | | 0.02 | up | | |
| MSTRG.1585 | rap1gap | 1.89 | 3.54 | | 2.47 | | | 0.32 | | 0.68 | | 0.75 | 1.29 | | | 0.02 | up | | |
| MSTRG.8692 | syf2 | 17.88 | 17.13 | | 20.58 | | | 51.78 | | 38.60 | | 34.74 | -1.01 | | | 0.02 | down | | |
| MSTRG.9017 | CPNE9 (1 of many) | 1.46 | 2.93 | | 1.35 | | | 0.45 | | 0.40 | | 0.57 | 1.14 | | | 0.02 | up | | |
| MSTRG.16758 | ENSONIG00000013897 | 4.38 | 5.16 | | 2.67 | | | 0.82 | | 1.05 | | 0.65 | 1.46 | | | 0.02 | up | | |
| MSTRG.22067 | hao1 | 46.13 | 62.26 | | 43.87 | | | 12.02 | | 14.84 | | 21.20 | 1.64 | | | 0.02 | up | | |
| MSTRG.2460 | faf1 | 5.58 | 6.07 | | 10.49 | | | 30.86 | | 40.82 | | 24.20 | -1.98 | | | 0.02 | down | | |
| MSTRG.7359 | zymnd12;emp3b | 0.03 | 0.16 | | 0.23 | | | 4.37 | | 1.79 | | 4.55 | -1.72 | | | 0.02 | down | | |
| MSTRG.13734 | fahd2a | 14.42 | 19.41 | | 12.69 | | | 2.62 | | 0.31 | | 3.22 | 2.93 | | | 0.02 | up | | |
| MSTRG.8034 | RASAL1 | 2.37 | 3.08 | | 3.40 | | | 0.64 | | 0.66 | | 1.29 | 1.15 | | | 0.02 | up | | |
| MSTRG.7014 | slc25a25a | 2.53 | 3.01 | | 4.69 | | | 200.09 | | 43.36 | | 45.56 | -3.51 | | | 0.02 | down | | |
| MSTRG.26003 | ENSONIG00000016101 | 1.58 | 3.69 | | 5.71 | | | 0.60 | | 0.20 | | 0.62 | 1.98 | | | 0.02 | up | | |
| MSTRG.2493 | pip5k1ca | 17.44 | 10.77 | | 20.30 | | | 3.33 | | 1.03 | | 2.33 | 2.48 | | | 0.02 | up | | |
| MSTRG.5005 | rnf25;kat8 | 6.49 | 5.70 | | 5.30 | | | 20.41 | | 15.24 | | 12.37 | -1.23 | | | 0.02 | down | | |
| MSTRG.3378 | nrxn3b | 1.31 | 0.07 | | 0.10 | | | 0.85 | | 6.23 | | 7.72 | -2.67 | | | 0.02 | down | | |
| MSTRG.17970 | ENSONIG00000017002 | 0.44 | 0.32 | | 0.18 | | | 110.98 | | 17.11 | | 21.98 | -4.21 | | | 0.02 | down | | |
| MSTRG.5659 | tefb | 1.62 | 2.19 | | 1.02 | | | 10.82 | | 5.65 | | 10.36 | -1.69 | | | 0.02 | down | | |
| MSTRG.8027 | NPC1L1 | 3.61 | 7.98 | | 5.28 | | | 0.71 | | 1.41 | | 1.39 | 1.75 | | | 0.02 | up | | |
| MSTRG.12915 | rab18a | 1.72 | 1.84 | | 2.68 | | | 15.27 | | 8.21 | | 7.56 | -1.59 | | | 0.02 | down | | |
| MSTRG.20178 | cryl1 | 8.23 | 11.83 | | 17.20 | | | 2.76 | | 3.23 | | 3.77 | 1.72 | | | 0.02 | up | | |
| MSTRG.17292 | FCHSD1 | 3.14 | 1.43 | | 1.81 | | | 0.35 | | 0.25 | | 0.13 | 1.09 | | | 0.02 | up | | |
| MSTRG.8236 | zc4h2 | 4.83 | 8.46 | | 5.02 | | | 1.61 | | 2.30 | | 2.03 | 1.35 | | | 0.02 | up | | |
| MSTRG.14766 | comtd1 | 503.74 | 504.17 | | 788.92 | | | 28.16 | | 105.58 | | 43.26 | 3.16 | | | 0.02 | up | | |
| MSTRG.1708 | slc12a5b | 12.26 | 13.90 | | 21.95 | | | 1.40 | | 2.71 | | 0.38 | 2.86 | | | 0.02 | up | | |
| MSTRG.375 | nob1 | 10.88 | 7.36 | | 14.39 | | | 20.56 | | 26.35 | | 30.54 | -1.44 | | | 0.02 | down | | |
| MSTRG.17721 | ENSONIG00000003583 | 0.40 | 1.09 | | 0.34 | | | 2.83 | | 3.74 | | 2.81 | -1.26 | | | 0.02 | down | | |
| MSTRG.2969 | ENSONIG00000014640 | 29.23 | 21.44 | | 24.81 | | | 4.12 | | 8.94 | | 3.56 | 1.79 | | | 0.02 | up | | |
| MSTRG.1850 | METTL4 | 0.65 | 2.18 | | 1.15 | | | 0 | | 0 | | 0.27 | 1.31 | | | 0.02 | up | | |
| MSTRG.10130 | nit1 | 5.72 | 8.07 | | 4.72 | | | 2.33 | | 2.21 | | 2.85 | 1.14 | | | 0.02 | up | | |
| MSTRG.4324 | eef2k | 19.26 | 27.68 | | 13.33 | | | 64.59 | | 78.19 | | 70.94 | -1.76 | | | 0.02 | down | | |
| MSTRG.20572 | si:ch211-276c2.2 | 0.19 | 1.30 | | 1.15 | | | 0 | | 0 | | 0 | 1.15 | | | 0.02 | up | | |
| MSTRG.2733 | ccng2 | 4.70 | 5.00 | | 3.02 | | | 19.90 | | 12.63 | | 23.30 | -1.79 | | | 0.02 | down | | |
| MSTRG.11738 | mast3b | 2.04 | 2.95 | | 3.17 | | | 0.75 | | 0.83 | | 1.27 | 1.01 | | | 0.02 | up | | |
| MSTRG.10094 | ENSONIG00000012900;ENSONIG00000012901 | 119.75 | 151.04 | | 190.66 | | | 93.87 | | 75.19 | | 61.08 | 1.21 | | | 0.02 | up | | |
| MSTRG.9406 | ATP6V1A (1 of many) | 3.70 | 5.09 | | 7.86 | | | 19.46 | | 16.54 | | 17.72 | -1.39 | | | 0.02 | down | | |
| MSTRG.20693 | ik;ndufa2 | 19.03 | 15.45 | | 22.43 | | | 45.46 | | 40.43 | | 35.03 | -1.08 | | | 0.02 | down | | |
| MSTRG.26857 | pam | 7.20 | 8.03 | | 5.06 | | | 28.57 | | 18.75 | | 18.11 | -1.37 | | | 0.02 | down | | |
| MSTRG.10386 | si:dkey-188i13.9 | 564.42 | 1039.67 | | 623.19 | | | 316.18 | | 77.56 | | 225.73 | 2.65 | | | 0.02 | up | | |
| MSTRG.8844 | ENSONIG00000006133 | 365.78 | 263.44 | | 228.57 | | | 51.93 | | 115.69 | | 86.57 | 1.38 | | | 0.02 | up | | |
| MSTRG.7064 | GZMA (1 of many) | 0.10 | 0 | | 0.15 | | | 2.23 | | 1.12 | | 1.23 | -1.09 | | | 0.02 | down | | |
| MSTRG.18570 | znf143b | 2.71 | 2.97 | | 2.88 | | | 10.99 | | 8.18 | | 6.11 | -1.11 | | | 0.02 | down | | |
| MSTRG.341 | dhcr7 | 12.05 | 40.62 | | 36.52 | | | 1.05 | | 3.62 | | 1.64 | 3.53 | | | 0.02 | up | | |
| MSTRG.21015 | igfbp1a | 0.52 | 0.19 | | 0.51 | | | 18.21 | | 5.06 | | 12.46 | -2.83 | | | 0.02 | down | | |
| MSTRG.3204 | fads2 (1 of many) | 49.46 | 170.74 | | 152.27 | | | 21.75 | | 26.21 | | 27.92 | 2.56 | | | 0.02 | up | | |
| MSTRG.5794 | mras | 0.14 | 0.55 | | 0.20 | | | 4.18 | | 1.70 | | 1.94 | -1.08 | | | 0.02 | down | | |
| MSTRG.3061 | pdp2 | 0.73 | 0.83 | | 1.01 | | | 6.72 | | 3.25 | | 7.04 | -1.62 | | | 0.02 | down | | |
| MSTRG.20004 | ENSONIG00000011683 | 2.09 | 4.15 | | 3.16 | | | 1.10 | | 1.19 | | 1.55 | 1.03 | | | 0.02 | up | | |
| MSTRG.8812 | enpp1 | 8.37 | 12.89 | | 7.31 | | | 50.54 | | 24.27 | | 30.96 | -1.37 | | | 0.02 | down | | |
| MSTRG.17373 | si:ch211-254p10.2 | 2.55 | 2.16 | | 1.88 | | | 0.93 | | 0.25 | | 0.47 | 1.14 | | | 0.02 | up | | |
| MSTRG.19693 | sat1b | 22.73 | 16.69 | | 21.26 | | | 197.38 | | 107.31 | | 77.62 | -2.38 | | | 0.02 | down | | |
| MSTRG.14218 | si:ch73-29c22.2 | 1.35 | 1.49 | | 1.33 | | | 0 | | 0 | | 0.45 | 1.06 | | | 0.02 | up | | |
| MSTRG.6812 | nme7 | 1.96 | 1.10 | | 1.73 | | | 5.75 | | 3.81 | | 4.77 | -1.20 | | | 0.02 | down | | |
| MSTRG.6346 | grb10a | 0.24 | 2.17 | | 0.15 | | | 8.75 | | 10.49 | | 10.13 | -2.37 | | | 0.02 | down | | |
| MSTRG.16837 | ENSONIG00000021121 | 3.72 | 0 | | 0.81 | | | 1.94 | | 3.00 | | 5.18 | -1.88 | | | 0.02 | down | | |
| MSTRG.18625 | morc3a | 3.04 | 4.40 | | 5.85 | | | 2.23 | | 1.66 | | 1.98 | 1.05 | | | 0.02 | up | | |
| MSTRG.314 | slc7a10a | 0.18 | 0.46 | | 0.06 | | | 6.99 | | 3.32 | | 2.53 | -1.70 | | | 0.02 | down | | |
| MSTRG.380 | ENSONIG00000022032 | 0 | 0 | | 0 | | | 1.37 | | 0.90 | | 2.17 | -1.25 | | | 0.02 | down | | |
| MSTRG.13146 | vezf1a | 3.08 | 3.23 | | 3.90 | | | 16.11 | | 8.16 | | 11.29 | -1.29 | | | 0.02 | down | | |
| MSTRG.20538 | ENSONIG00000003834 | 0.26 | 0.52 | | 0.25 | | | 3.33 | | 1.98 | | 1.53 | -1.03 | | | 0.02 | down | | |
| MSTRG.11109 | ENSONIG00000018485 | 6.92 | 4.16 | | 4.84 | | | 12.50 | | 9.60 | | 11.92 | -1.11 | | | 0.02 | down | | |
| MSTRG.23679 | rasal2 | 4.04 | 2.54 | | 3.87 | | | 9.46 | | 7.85 | | 6.97 | -1.11 | | | 0.02 | down | | |
| MSTRG.1703 | magi1a | 4.90 | 10.21 | | 5.02 | | | 3.62 | | 2.18 | | 3.51 | 1.23 | | | 0.02 | up | | |
| MSTRG.16456 | ENSONIG00000022001 | 1.27 | 1.35 | | 1.92 | | | 4.25 | | 9.95 | | 5.92 | -1.74 | | | 0.02 | down | | |
| MSTRG.4984 | TAOK2 (1 of many) | 0.18 | 0.16 | | 0.09 | | | 1135.19 | | 76.32 | | 71.74 | -6.40 | | | 0.02 | down | | |
| MSTRG.16052 | asap3 | 0.62 | 0.73 | | 1.41 | | | 3.03 | | 4.91 | | 3.50 | -1.39 | | | 0.02 | down | | |
| MSTRG.22571 | smad9 | 0.81 | 0.60 | | 0.42 | | | 1.05 | | 2.20 | | 2.29 | -1.02 | | | 0.02 | down | | |
| MSTRG.14379 | ENSONIG00000013320 | 0.81 | 0.38 | | 0.58 | | | 4.68 | | 2.37 | | 3.43 | -1.44 | | | 0.02 | down | | |
| MSTRG.13792 | ENSONIG00000007424 | 0.43 | 1.94 | | 2.18 | | | 0.58 | | 0.14 | | 0.40 | 1.23 | | | 0.02 | up | | |
| MSTRG.18341 | atp7a | 0.73 | 0.82 | | 0.66 | | | 8.10 | | 3.13 | | 5.17 | -1.57 | | | 0.02 | down | | |
| MSTRG.7761 | ASS1 | 7.54 | 6.02 | | 11.26 | | | 0.48 | | 1.49 | | 1.78 | 1.79 | | | 0.02 | up | | |
| MSTRG.21013 | igfbp1a | 0.46 | 0.35 | | 0.57 | | | 4.45 | | 2.20 | | 2.47 | -1.28 | | | 0.02 | down | | |
| MSTRG.3742 | ENSONIG00000009945 | 1.35 | 1.68 | | 2.55 | | | 0.33 | | 0.49 | | 0.38 | 1.06 | | | 0.02 | up | | |
| MSTRG.17363 | ENSONIG00000002804;ENSONIG00000002805 | 19.43 | 23.49 | | 15.62 | | | 5.21 | | 6.40 | | 8.47 | 1.40 | | | 0.02 | up | | |
| MSTRG.24785 | atad2 (1 of many) | 3.06 | 3.26 | | 3.52 | | | 1.57 | | 0.60 | | 0.24 | 1.53 | | | 0.02 | up | | |
| MSTRG.977 | tnfaip3 | 0.51 | 0.87 | | 4.33 | | | 25.40 | | 24.52 | | 40.52 | -3.52 | | | 0.02 | down | | |
| MSTRG.14757 | zcchc24 | 0.69 | 0.53 | | 0.49 | | | 6.70 | | 3.01 | | 7.22 | -1.90 | | | 0.02 | down | | |
| MSTRG.25570 | ENSONIG00000008284 | 0 | 0 | | 0.02 | | | 1.39 | | 1.63 | | 0.70 | -1.12 | | | 0.02 | down | | |
| MSTRG.11254 | loxl2b | 0.70 | 0.51 | | 0.32 | | | 3.85 | | 2.28 | | 2.23 | -1.23 | | | 0.02 | down | | |
| MSTRG.22588 | ubl3a | 17.37 | 10.93 | | 16.30 | | | 129.55 | | 74.86 | | 55.27 | -2.36 | | | 0.02 | down | | |
| MSTRG.6912 | sardh | 19.82 | 41.43 | | 13.79 | | | 4.26 | | 5.03 | | 3.04 | 2.50 | | | 0.02 | up | | |
| MSTRG.16092 | rsad2 | 1.96 | 4.28 | | 9.80 | | | 0.29 | | 0 | | 0 | 2.73 | | | 0.02 | up | | |
| MSTRG.23936 | ENSONIG00000001462 | 2.37 | 5.23 | | 4.86 | | | 0.54 | | 0.73 | | 1.27 | 1.66 | | | 0.02 | up | | |
| MSTRG.18102 | znf618 | 1.48 | 2.49 | | 1.22 | | | 14.81 | | 14.74 | | 6.98 | -2.02 | | | 0.02 | down | | |
| MSTRG.5856 | igfbp2b | 97.45 | 106.55 | | 86.11 | | | 17.46 | | 17.01 | | 36.03 | 2.04 | | | 0.02 | up | | |
| MSTRG.7852 | ENSONIG00000004471 | 2.07 | 2.92 | | 3.94 | | | 1.49 | | 0.15 | | 0.75 | 1.54 | | | 0.02 | up | | |
| MSTRG.16105 | LRR1 | 0.87 | 0.79 | | 1.01 | | | 7.99 | | 4.29 | | 3.68 | -1.50 | | | 0.02 | down | | |
| MSTRG.10907 | bgnb | 11.77 | 10.44 | | 12.07 | | | 5.15 | | 2.94 | | 5.25 | 1.28 | | | 0.02 | up | | |
| MSTRG.19420 | ldhba | 13.86 | 13.65 | | 17.23 | | | 111.28 | | 48.46 | | 55.69 | -1.82 | | | 0.02 | down | | |
| MSTRG.3649 | rassf4 | 5.99 | 2.19 | | 5.50 | | | 9.63 | | 119.76 | | 95.25 | -4.41 | | | 0.02 | down | | |
| MSTRG.16786 | pdlim5b | 4.44 | 4.68 | | 3.50 | | | 10.09 | | 13.23 | | 8.35 | -1.17 | | | 0.02 | down | | |
| MSTRG.1829 | ENSONIG00000006023 | 0.72 | 1.11 | | 0.28 | | | 3.99 | | 3.42 | | 2.80 | -1.26 | | | 0.02 | down | | |
| MSTRG.8020 | rnf34a | 3.84 | 5.17 | | 3.93 | | | 3.18 | | 1.31 | | 2.32 | 1.01 | | | 0.02 | up | | |
| MSTRG.2040 | ces3 | 158.35 | 248.49 | | 219.40 | | | 12.58 | | 31.55 | | 37.68 | 2.88 | | | 0.02 | up | | |
| MSTRG.896 | minpp1a | 40.37 | 54.13 | | 58.03 | | | 13.56 | | 22.62 | | 16.69 | 1.48 | | | 0.02 | up | | |
| MSTRG.18104 | RGS3 | 0.88 | 1.06 | | 2.40 | | | 5.86 | | 6.10 | | 7.52 | -1.63 | | | 0.02 | down | | |
| MSTRG.4552 | stat3 | 20.17 | 17.79 | | 14.82 | | | 98.32 | | 51.81 | | 54.76 | -1.69 | | | 0.02 | down | | |
| MSTRG.4411 | coasy | 5.54 | 8.29 | | 6.58 | | | 21.13 | | 21.81 | | 13.90 | -1.16 | | | 0.02 | down | | |
| MSTRG.5959 | nipsnap3a | 37.42 | 32.55 | | 22.78 | | | 60.40 | | 109.04 | | 70.03 | -1.56 | | | 0.02 | down | | |
| MSTRG.12686 | mxi1 | 2.08 | 2.62 | | 3.15 | | | 5.10 | | 6.81 | | 7.08 | -1.03 | | | 0.02 | down | | |
| MSTRG.10416 | cfd | 8.12 | 9.10 | | 6.41 | | | 2.19 | | 2.88 | | 3.61 | 1.14 | | | 0.02 | up | | |
| MSTRG.26052 | asns | 1.90 | 12.48 | | 4.78 | | | 1.45 | | 1.32 | | 1.33 | 2.06 | | | 0.03 | up | | |
| MSTRG.7674 | ENSONIG00000002436 | 0.78 | 4.56 | | 1.09 | | | 0.20 | | 0.08 | | 0.03 | 1.85 | | | 0.03 | up | | |
| MSTRG.8609 | CSGALNACT1 (1 of many) | 0.94 | 2.90 | | 2.25 | | | 0.50 | | 0.58 | | 0 | 1.47 | | | 0.03 | up | | |
| MSTRG.946 | slc16a9a | 0.10 | 0.02 | | 0.09 | | | 13.90 | | 5.99 | | 3.57 | -2.59 | | | 0.03 | down | | |
| MSTRG.2816 | tsc1b | 7.24 | 9.74 | | 11.67 | | | 34.83 | | 23.66 | | 20.59 | -1.09 | | | 0.03 | down | | |
| MSTRG.5615 | srebf2 | 2.94 | 7.75 | | 4.88 | | | 1.98 | | 1.93 | | 2.46 | 1.26 | | | 0.03 | up | | |
| MSTRG.21287 | csrnp1a | 2.93 | 2.27 | | 3.22 | | | 10.16 | | 6.40 | | 7.30 | -1.16 | | | 0.03 | down | | |
| MSTRG.7260 | efemp2a | 0.32 | 0.22 | | 0.38 | | | 10.06 | | 3.14 | | 5.09 | -2.05 | | | 0.03 | down | | |
| MSTRG.9385 | nfrkb | 0.96 | 1.69 | | 1.53 | | | 4.19 | | 5.44 | | 3.54 | -1.07 | | | 0.03 | down | | |
| MSTRG.16540 | pisd | 48.31 | 59.00 | | 43.73 | | | 17.43 | | 17.81 | | 8.32 | 1.93 | | | 0.03 | up | | |
| MSTRG.1139 | sult2st2 | 2.29 | 3.07 | | 3.81 | | | 0.43 | | 0.53 | | 1.10 | 1.36 | | | 0.03 | up | | |
| MSTRG.6683 | ctss1 | 0.87 | 0.24 | | 0.60 | | | 4.02 | | 2.23 | | 2.99 | -1.42 | | | 0.03 | down | | |
| MSTRG.17851 | ENSONIG00000007916 | 0.35 | 2.26 | | 1.49 | | | 0.06 | | 0.02 | | 0.24 | 1.40 | | | 0.03 | up | | |
| MSTRG.14119 | hsd11b1la (1 of many) | 52.05 | 62.09 | | 53.43 | | | 16.44 | | 27.03 | | 24.28 | 1.21 | | | 0.03 | up | | |
| MSTRG.3858 | prdx3 | 45.18 | 50.75 | | 62.72 | | | 17.41 | | 26.19 | | 22.12 | 1.18 | | | 0.03 | up | | |
| MSTRG.12808 | zgc:112492 | 19.63 | 47.06 | | 182.28 | | | 4.78 | | 1.95 | | 1.60 | 4.70 | | | 0.03 | up | | |
| MSTRG.5591 | myh10 (1 of many) | 6.42 | 14.44 | | 6.69 | | | 63.26 | | 28.63 | | 27.72 | -1.40 | | | 0.03 | down | | |
| MSTRG.9700 | cecr5 | 17.23 | 19.35 | | 23.46 | | | 4.50 | | 7.03 | | 8.26 | 1.40 | | | 0.03 | up | | |
| MSTRG.19454 | SNTA1 | 4.56 | 5.42 | | 3.69 | | | 1.44 | | 2.09 | | 1.19 | 1.12 | | | 0.03 | up | | |
| MSTRG.16622 | usp2b | 1.80 | 0.78 | | 0.85 | | | 8.70 | | 5.79 | | 4.40 | -1.82 | | | 0.03 | down | | |
| MSTRG.4038 | cdk2 | 1.96 | 2.36 | | 3.90 | | | 0.87 | | 0.47 | | 0.79 | 1.24 | | | 0.03 | up | | |
| MSTRG.3077 | KCP | 4.07 | 0.78 | | 0.95 | | | 1.25 | | 3.08 | | 2.96 | -1.04 | | | 0.03 | down | | |
| MSTRG.12797 | si:dkey-29p9.3 | 72.56 | 115.53 | | 53.65 | | | 10.35 | | 9.56 | | 21.48 | 2.61 | | | 0.03 | up | | |
| MSTRG.12711 | sid4 | 2.27 | 1.79 | | 3.67 | | | 0.24 | | 0.29 | | 0.52 | 1.30 | | | 0.03 | up | | |
| MSTRG.17306 | wdhd1 | 0.47 | 1.47 | | 0.74 | | | 0 | | 0 | | 0.20 | 1.00 | | | 0.03 | up | | |
| MSTRG.10873 | copz1 | 10.50 | 6.17 | | 11.10 | | | 20.50 | | 19.42 | | 17.81 | -1.19 | | | 0.03 | down | | |
| MSTRG.4969 | nupr1 | 5.83 | 4.76 | | 1.41 | | | 31.47 | | 26.23 | | 56.07 | -3.15 | | | 0.03 | down | | |
| MSTRG.3476 | VANGL1 (1 of many) | 0.35 | 0.90 | | 0.59 | | | 4.74 | | 2.74 | | 2.03 | -1.01 | | | 0.03 | down | | |
| MSTRG.9491 | spry4 | 0.86 | 0.83 | | 0.69 | | | 2.79 | | 4.42 | | 2.18 | -1.25 | | | 0.03 | down | | |
| MSTRG.20722 | ENSONIG00000017881 | 3.92 | 2.39 | | 2.86 | | | 23.05 | | 11.08 | | 12.70 | -1.92 | | | 0.03 | down | | |
| MSTRG.13767 | mapkapk5 | 5.76 | 5.56 | | 5.97 | | | 28.04 | | 13.47 | | 22.39 | -1.48 | | | 0.03 | down | | |
| MSTRG.6488 | DENND2A (1 of many) | 1.36 | 1.59 | | 3.30 | | | 14.71 | | 11.93 | | 8.05 | -1.87 | | | 0.03 | down | | |
| MSTRG.8728 | ENSONIG00000005558 | 0.58 | 0.15 | | 1.05 | | | 2.96 | | 17.16 | | 6.44 | -2.95 | | | 0.03 | down | | |
| MSTRG.7457 | ENSONIG00000011552 | 1.08 | 1.14 | | 2.39 | | | 0.03 | | 0 | | 0.08 | 1.29 | | | 0.03 | up | | |
| MSTRG.7256 | yif1a | 8.16 | 6.40 | | 5.45 | | | 27.56 | | 19.65 | | 16.00 | -1.48 | | | 0.03 | down | | |
| MSTRG.8772 | LAMA2 | 7.00 | 5.10 | | 3.66 | | | 1.08 | | 1.34 | | 1.77 | 1.16 | | | 0.03 | up | | |
| MSTRG.4659 | nr5a5 | 12.58 | 14.53 | | 10.24 | | | 1.58 | | 4.30 | | 2.21 | 1.77 | | | 0.03 | up | | |
| MSTRG.2548 | ENSONIG00000002748 | 309.95 | 203.67 | | 254.05 | | | 31.13 | | 67.77 | | 21.52 | 2.45 | | | 0.03 | up | | |
| MSTRG.19785 | slc43a2a | 0.32 | 5.62 | | 3.87 | | | 56.18 | | 38.18 | | 19.31 | -2.42 | | | 0.03 | down | | |
| MSTRG.3406 | ENSONIG00000001190 | 6927.10 | 8938.75 | | 5150.25 | | | 891.37 | | 1990.15 | | 1389.12 | 2.19 | | | 0.03 | up | | |
| MSTRG.15508 | dbpa | 7.70 | 15.17 | | 3.54 | | | 1.52 | | 0.37 | | 0.49 | 2.76 | | | 0.03 | up | | |
| MSTRG.18027 | znf598 | 4.76 | 3.57 | | 5.52 | | | 15.55 | | 12.96 | | 9.88 | -1.30 | | | 0.03 | down | | |
| MSTRG.10896 | tnnt2a | 0.25 | 0.20 | | 0.11 | | | 1.49 | | 1.81 | | 3.78 | -1.56 | | | 0.03 | down | | |
| MSTRG.17940 | rwdd1 | 0.98 | 1.23 | | 0.71 | | | 5.83 | | 6.54 | | 3.22 | -1.55 | | | 0.03 | down | | |
| MSTRG.3172 | vwa9 | 6.36 | 13.08 | | 11.47 | | | 7.65 | | 1.42 | | 4.25 | 1.85 | | | 0.03 | up | | |
| MSTRG.10793 | mmp9 | 0.29 | 0.32 | | 0.73 | | | 29.34 | | 7.57 | | 8.18 | -2.76 | | | 0.03 | down | | |
| MSTRG.9575 | cldnc | 0 | 0.29 | | 0 | | | 2.76 | | 1.15 | | 1.19 | -1.00 | | | 0.03 | down | | |
| MSTRG.6513 | ENSONIG00000008890 | 214.58 | 176.13 | | 146.07 | | | 31.12 | | 71.42 | | 53.58 | 1.47 | | | 0.03 | up | | |
| MSTRG.2482 | tmem161a | 5.55 | 4.86 | | 6.91 | | | 1.69 | | 0.84 | | 2.16 | 1.46 | | | 0.03 | up | | |
| MSTRG.11234 | tacr1a | 2.98 | 6.16 | | 2.82 | | | 1.68 | | 1.03 | | 1.81 | 1.26 | | | 0.03 | up | | |
| MSTRG.19119 | SLC9A3R2 (1 of many) | 0.23 | 1.69 | | 0.72 | | | 0.20 | | 0 | | 0.28 | 1.01 | | | 0.03 | up | | |
| MSTRG.13318 | ENSONIG00000013024 | 0.16 | 0.36 | | 1.16 | | | 3.04 | | 4.07 | | 4.63 | -1.69 | | | 0.03 | down | | |
| MSTRG.2901 | ENSONIG00000013183 | 28.54 | 19.04 | | 35.60 | | | 6.49 | | 10.91 | | 8.71 | 1.25 | | | 0.03 | up | | |
| MSTRG.1371 | decr2 | 25.23 | 40.80 | | 29.95 | | | 6.85 | | 9.86 | | 12.74 | 1.68 | | | 0.03 | up | | |
| MSTRG.6428 | cdk17 (1 of many) | 0.60 | 0.94 | | 1.58 | | | 3.56 | | 3.31 | | 4.16 | -1.12 | | | 0.03 | down | | |
| MSTRG.18997 | mcm10 | 5.02 | 1.82 | | 2.39 | | | 28.33 | | 24.96 | | 11.37 | -2.68 | | | 0.03 | down | | |
| MSTRG.6133 | MTSS1 (1 of many) | 12.05 | 11.57 | | 15.08 | | | 35.50 | | 32.11 | | 23.59 | -1.11 | | | 0.03 | down | | |
| MSTRG.14289 | rnaseh2b | 5.08 | 3.95 | | 2.97 | | | 15.33 | | 9.51 | | 13.59 | -1.45 | | | 0.03 | down | | |
| MSTRG.4939 | ENSONIG00000009301 | 41.46 | 57.90 | | 31.99 | | | 6.43 | | 12.14 | | 11.45 | 1.96 | | | 0.03 | up | | |
| MSTRG.15071 | haao | 113.83 | 144.32 | | 133.55 | | | 16.95 | | 37.96 | | 36.53 | 1.97 | | | 0.03 | up | | |
| MSTRG.13425 | senp8 | 0.35 | 0.52 | | 0.04 | | | 2.59 | | 2.72 | | 1.56 | -1.27 | | | 0.03 | down | | |
| MSTRG.19323 | cyp3a65 (1 of many) | 22.03 | 10.80 | | 18.24 | | | 203.95 | | 118.38 | | 72.43 | -2.86 | | | 0.03 | down | | |
| MSTRG.16690 | klf1 | 2.72 | 2.75 | | 1.78 | | | 1.03 | | 0.14 | | 0.25 | 1.50 | | | 0.03 | up | | |
| MSTRG.3341 | entpd5b;aldh6a1 (1 of many) | 14.85 | 14.73 | | 10.54 | | | 3.82 | | 6.21 | | 3.94 | 1.24 | | | 0.03 | up | | |
| MSTRG.1330 | raraa | 0.70 | 0.70 | | 0.63 | | | 2.18 | | 1.80 | | 3.26 | -1.00 | | | 0.03 | down | | |
| MSTRG.14251 | ENSONIG00000007616 | 23.06 | 38.00 | | 23.41 | | | 3.29 | | 4.39 | | 8.64 | 2.29 | | | 0.03 | up | | |
| MSTRG.14423 | pxmp2 | 66.65 | 47.17 | | 102.30 | | | 13.83 | | 13.49 | | 20.26 | 1.91 | | | 0.03 | up | | |
| MSTRG.22854 | ENSONIG00000021280 | 0.84 | 1.61 | | 2.96 | | | 0 | | 0 | | 0 | 1.60 | | | 0.03 | up | | |
| MSTRG.1327 | itga3b | 0.34 | 0.36 | | 0.36 | | | 7.27 | | 5.69 | | 2.52 | -1.95 | | | 0.03 | down | | |
| MSTRG.21585 | tmem88b | 6.94 | 6.07 | | 6.68 | | | 35.21 | | 16.47 | | 23.03 | -1.54 | | | 0.03 | down | | |
| MSTRG.16369 | FZD6 | 3.65 | 2.08 | | 4.49 | | | 10.91 | | 8.29 | | 11.79 | -1.50 | | | 0.03 | down | | |
| MSTRG.10498 | NFIX (1 of many) | 5.43 | 8.21 | | 5.11 | | | 2.61 | | 2.40 | | 3.44 | 1.05 | | | 0.03 | up | | |
| MSTRG.6021 | MCMDC2 | 8.29 | 8.11 | | 9.43 | | | 23.84 | | 20.11 | | 15.11 | -1.00 | | | 0.03 | down | | |
| MSTRG.9644 | cecr1a | 1.27 | 3.21 | | 2.00 | | | 0.45 | | 0.77 | | 0.48 | 1.19 | | | 0.03 | up | | |
| MSTRG.1533 | ENSONIG00000016742 | 4.06 | 8.21 | | 4.28 | | | 0.31 | | 1.01 | | 1.13 | 1.92 | | | 0.03 | up | | |
| MSTRG.16840 | ENSONIG00000016400 | 0.51 | 0.72 | | 1.25 | | | 18.21 | | 5.93 | | 6.81 | -2.07 | | | 0.03 | down | | |
| MSTRG.19218 | ENSONIG00000009619 | 2.04 | 3.61 | | 3.12 | | | 0.36 | | 0.69 | | 1.04 | 1.30 | | | 0.03 | up | | |
| MSTRG.4688 | tmed1a | 4.18 | 11.39 | | 7.18 | | | 1.71 | | 1.76 | | 2.99 | 1.72 | | | 0.03 | up | | |
| MSTRG.15185 | zgc:110591 | 4.51 | 3.53 | | 5.88 | | | 18.75 | | 14.71 | | 11.23 | -1.46 | | | 0.03 | down | | |
| MSTRG.10270 | stab1 | 0 | 0 | | 0.35 | | | 3.80 | | 5.90 | | 1.97 | -2.09 | | | 0.03 | down | | |
| MSTRG.17701 | ENSONIG00000003615 | 1.75 | 3.67 | | 1.00 | | | 0.43 | | 0.06 | | 0.08 | 1.64 | | | 0.03 | up | | |
| MSTRG.22042 | ENSONIG00000020404 | 0.04 | 0 | | 0 | | | 0.59 | | 2.36 | | 1.19 | -1.40 | | | 0.03 | down | | |
| MSTRG.8064 | lrrc75ba | 0 | 0 | | 0 | | | 1.53 | | 1.69 | | 0.69 | -1.15 | | | 0.03 | down | | |
| MSTRG.1908 | ralaa | 3.20 | 3.39 | | 2.87 | | | 13.47 | | 7.33 | | 8.04 | -1.11 | | | 0.03 | down | | |
| MSTRG.20391 | abhd2a | 2.06 | 10.31 | | 6.54 | | | 11.71 | | 1.72 | | 2.20 | 1.53 | | | 0.03 | up | | |
| MSTRG.5967 | ENSONIG00000014066 | 26.28 | 29.22 | | 39.99 | | | 99.91 | | 64.36 | | 89.79 | -1.20 | | | 0.03 | down | | |
| MSTRG.8273 | TSPAN17 | 1.15 | 2.38 | | 1.79 | | | 13.97 | | 5.25 | | 6.07 | -1.18 | | | 0.03 | down | | |
| MSTRG.12662 | gne | 5.58 | 11.49 | | 7.21 | | | 1.03 | | 1.22 | | 2.75 | 1.97 | | | 0.03 | up | | |
| MSTRG.1582 | slc38a5a | 12.86 | 19.62 | | 20.89 | | | 5.19 | | 7.54 | | 3.70 | 1.69 | | | 0.03 | up | | |
| MSTRG.24993 | pik3cb | 2.48 | 1.44 | | 3.14 | | | 9.62 | | 6.51 | | 7.59 | -1.46 | | | 0.03 | down | | |
| MSTRG.9997 | pih1d3 | 2.73 | 2.99 | | 2.17 | | | 1.46 | | 0.26 | | 0.38 | 1.40 | | | 0.03 | up | | |
| MSTRG.15838 | amd1 | 5.37 | 4.65 | | 3.96 | | | 2.46 | | 1.37 | | 1.24 | 1.17 | | | 0.03 | up | | |
| MSTRG.12009 | lrrc51 (1 of many) | 0.92 | 0.96 | | 0.88 | | | 3.87 | | 3.82 | | 2.19 | -1.08 | | | 0.03 | down | | |
| MSTRG.4387 | ascc1 | 2.60 | 1.59 | | 1.84 | | | 15.66 | | 7.01 | | 9.48 | -1.83 | | | 0.03 | down | | |
| MSTRG.862 | tbl1xr1b | 5.54 | 7.19 | | 3.15 | | | 0.58 | | 1.00 | | 1.02 | 1.70 | | | 0.03 | up | | |
| MSTRG.15221 | ddah2 | 0.72 | 2.20 | | 1.90 | | | 2.03 | | 0.21 | | 0.71 | 1.02 | | | 0.03 | up | | |
| MSTRG.10756 | ENSONIG00000017979 | 16.53 | 23.58 | | 16.72 | | | 7.23 | | 8.83 | | 10.22 | 1.09 | | | 0.03 | up | | |
| MSTRG.10196 | glra1 | 2.41 | 8.14 | | 2.49 | | | 0 | | 0 | | 0.60 | 2.39 | | | 0.03 | up | | |
| MSTRG.15109 | cyr61 | 0.26 | 0.08 | | 0.09 | | | 9.07 | | 2.58 | | 6.74 | -2.30 | | | 0.03 | down | | |
| MSTRG.18265 | ENSONIG00000011218 | 0.14 | 2.65 | | 1.90 | | | 0 | | 0 | | 0 | 1.73 | | | 0.03 | up | | |
| MSTRG.17635 | CFI | 344.29 | 314.26 | | 422.29 | | | 66.84 | | 144.90 | | 111.96 | 1.51 | | | 0.03 | up | | |
| MSTRG.13704 | vdac3 | 13.66 | 9.46 | | 6.94 | | | 21.44 | | 24.24 | | 19.23 | -1.26 | | | 0.03 | down | | |
| MSTRG.23622 | ENSONIG00000006649 | 5.22 | 5.97 | | 5.48 | | | 0.22 | | 0.13 | | 1.59 | 2.09 | | | 0.03 | up | | |
| MSTRG.21840 | creg1 | 36.83 | 46.85 | | 30.44 | | | 17.74 | | 17.20 | | 21.60 | 1.04 | | | 0.03 | up | | |
| MSTRG.3721 | itga9 | 2.25 | 5.46 | | 4.60 | | | 1.22 | | 1.27 | | 1.82 | 1.30 | | | 0.03 | up | | |
| MSTRG.7849 | tfcp2l1 | 3.90 | 10.61 | | 2.37 | | | 0.49 | | 0.41 | | 0.28 | 2.43 | | | 0.03 | up | | |
| MSTRG.6896 | anxa3b | 14.66 | 10.00 | | 10.70 | | | 35.32 | | 31.17 | | 21.96 | -1.33 | | | 0.03 | down | | |
| MSTRG.6392 | cox5aa | 23.35 | 13.61 | | 24.21 | | | 47.84 | | 50.14 | | 36.75 | -1.33 | | | 0.03 | down | | |
| MSTRG.2521 | CYP2J2 (1 of many) | 0 | 1.61 | | 1.15 | | | 0.08 | | 0 | | 0 | 1.25 | | | 0.03 | up | | |
| MSTRG.17090 | xbp1 | 81.19 | 68.28 | | 146.44 | | | 272.22 | | 310.71 | | 372.98 | -1.86 | | | 0.03 | down | | |
| MSTRG.1791 | zgc:153639 | 3.95 | 4.39 | | 7.41 | | | 0.54 | | 1.40 | | 0.88 | 1.60 | | | 0.03 | up | | |
| MSTRG.9114 | ENSONIG00000012262 | 0.52 | 0.12 | | 0.20 | | | 2.32 | | 1.62 | | 1.26 | -1.13 | | | 0.03 | down | | |
| MSTRG.17855 | rap2b | 2.40 | 2.84 | | 3.69 | | | 50.71 | | 18.27 | | 15.55 | -2.26 | | | 0.03 | down | | |
| MSTRG.2453 | tpm2 | 25.36 | 22.16 | | 27.55 | | | 70.82 | | 54.33 | | 45.24 | -1.07 | | | 0.03 | down | | |
| MSTRG.20254 | pcca | 14.91 | 18.31 | | 21.61 | | | 2.78 | | 5.76 | | 5.87 | 1.66 | | | 0.03 | up | | |
| MSTRG.15445 | gda | 37.37 | 44.57 | | 34.38 | | | 24.85 | | 18.82 | | 13.13 | 1.23 | | | 0.03 | up | | |
| MSTRG.9246 | tmem242 | 2.29 | 0.74 | | 0.90 | | | 4.96 | | 3.62 | | 3.94 | -1.43 | | | 0.03 | down | | |
| MSTRG.16154 | fzr1b | 13.70 | 9.14 | | 10.02 | | | 22.46 | | 17.68 | | 25.49 | -1.06 | | | 0.03 | down | | |
| MSTRG.10698 | pah | 358.44 | 491.79 | | 368.64 | | | 367.70 | | 167.87 | | 187.30 | 1.23 | | | 0.03 | up | | |
| MSTRG.19034 | slc38a4 | 110.57 | 115.45 | | 77.23 | | | 178.15 | | 225.83 | | 253.86 | -1.21 | | | 0.03 | down | | |
| MSTRG.6905 | ambp | 1756.19 | 1955.69 | | 1522.45 | | | 503.19 | | 744.96 | | 838.62 | 1.24 | | | 0.03 | up | | |
| MSTRG.13962 | adam15 | 0.46 | 0.46 | | 0.64 | | | 4.23 | | 2.31 | | 2.03 | -1.12 | | | 0.03 | down | | |
| MSTRG.18326 | ENSONIG00000012894 | 0.84 | 3.16 | | 0.92 | | | 0.00 | | 0.03 | | 0.10 | 1.56 | | | 0.03 | up | | |
| MSTRG.13102 | tmem256 | 2.79 | 3.06 | | 3.64 | | | 7.51 | | 12.07 | | 6.91 | -1.27 | | | 0.03 | down | | |
| MSTRG.5570 | ldlra | 27.96 | 69.47 | | 43.60 | | | 19.77 | | 20.70 | | 23.78 | 1.37 | | | 0.03 | up | | |
| MSTRG.3682 | elovl5 | 53.62 | 39.84 | | 101.75 | | | 4.85 | | 12.57 | | 5.70 | 2.60 | | | 0.03 | up | | |
| MSTRG.8260 | clint1a | 5.67 | 2.99 | | 5.26 | | | 14.23 | | 11.14 | | 10.14 | -1.34 | | | 0.03 | down | | |
| MSTRG.13124 | tm7sf2 | 84.80 | 144.92 | | 157.60 | | | 7.84 | | 26.28 | | 15.57 | 2.89 | | | 0.03 | up | | |
| MSTRG.15464 | arrdc3a | 13.95 | 7.08 | | 8.76 | | | 15.64 | | 87.76 | | 121.68 | -3.32 | | | 0.03 | down | | |
| MSTRG.15665 | KHSRP (1 of many) | 6.03 | 3.65 | | 2.75 | | | 0.91 | | 1.07 | | 0.84 | 1.14 | | | 0.03 | up | | |
| MSTRG.13571 | epb41l4b | 0.77 | 0.53 | | 0.85 | | | 4.99 | | 3.71 | | 2.34 | -1.35 | | | 0.03 | down | | |
| MSTRG.20207 | cx32.3 | 14.36 | 19.50 | | 3.09 | | | 143.45 | | 157.28 | | 169.07 | -3.83 | | | 0.03 | down | | |
| MSTRG.19007 | pfkfb3 | 0.33 | 0.44 | | 0.65 | | | 24.91 | | 9.25 | | 5.62 | -2.63 | | | 0.03 | down | | |
| MSTRG.13833 | ENSONIG00000007433 | 85.41 | 119.96 | | 57.85 | | | 10.76 | | 19.36 | | 21.52 | 2.20 | | | 0.03 | up | | |
| MSTRG.9076 | zgc:112255 | 5.68 | 28.75 | | 20.24 | | | 4.90 | | 5.28 | | 4.19 | 2.16 | | | 0.03 | up | | |
| MSTRG.7934 | ENSONIG00000019558 | 7.95 | 8.15 | | 11.51 | | | 1.93 | | 3.89 | | 2.45 | 1.34 | | | 0.03 | up | | |
| MSTRG.8469 | adrm1 | 3.39 | 2.69 | | 4.56 | | | 8.86 | | 9.89 | | 6.87 | -1.14 | | | 0.03 | down | | |
| MSTRG.13232 | etfb | 33.65 | 29.60 | | 34.29 | | | 9.02 | | 17.64 | | 13.55 | 1.02 | | | 0.03 | up | | |
| MSTRG.18495 | 5S_rRNA | 0.36 | 0.27 | | 0.09 | | | 0.81 | | 1.56 | | 2.29 | -1.19 | | | 0.03 | down | | |
| MSTRG.2517 | cyp2n13 | 6.38 | 6.62 | | 8.09 | | | 1.65 | | 3.35 | | 1.81 | 1.22 | | | 0.03 | up | | |
| MSTRG.19493 | hspb1 | 1.11 | 0.27 | | 0.57 | | | 126.38 | | 42.92 | | 15.23 | -4.59 | | | 0.03 | down | | |
| MSTRG.18337 | fbxl3l | 0.06 | 0.45 | | 0.81 | | | 2.70 | | 2.65 | | 4.35 | -1.45 | | | 0.03 | down | | |
| MSTRG.12798 | si:dkey-29p9.3 | 26.51 | 33.65 | | 23.15 | | | 4.98 | | 7.83 | | 10.70 | 1.66 | | | 0.03 | up | | |
| MSTRG.14239 | kcmf1 (1 of many) | 2.04 | 2.08 | | 2.65 | | | 9.87 | | 7.60 | | 5.16 | -1.23 | | | 0.03 | down | | |
| MSTRG.11114 | crygn1 | 1.01 | 7.65 | | 1.32 | | | 1.60 | | 0.04 | | 0.77 | 1.91 | | | 0.03 | up | | |
| MSTRG.8675 | gale | 5.40 | 2.34 | | 8.77 | | | 30.48 | | 25.00 | | 22.60 | -2.35 | | | 0.03 | down | | |
| MSTRG.1513 | cs | 32.08 | 34.50 | | 42.53 | | | 17.49 | | 18.55 | | 11.31 | 1.24 | | | 0.03 | up | | |
| MSTRG.14773 | pawr | 2.43 | 3.37 | | 1.63 | | | 21.24 | | 11.80 | | 8.80 | -1.73 | | | 0.03 | down | | |
| MSTRG.16416 | fetub (1 of many) | 3826.94 | 3020.82 | | 3749.54 | | | 306.98 | | 1067.54 | | 395.42 | 2.36 | | | 0.03 | up | | |
| MSTRG.13008 | sh3bp5b | 0.58 | 0.42 | | 0.63 | | | 3.52 | | 2.35 | | 1.76 | -1.08 | | | 0.03 | down | | |
| MSTRG.9062 | si:dkey-166c18.1 | 1.22 | 7.33 | | 1.66 | | | 0.38 | | 0.31 | | 0.25 | 2.07 | | | 0.03 | up | | |
| MSTRG.3320 | ENSONIG00000000805 | 4.79 | 5.25 | | 12.94 | | | 1156.11 | | 186.30 | | 139.23 | -4.55 | | | 0.03 | down | | |
| MSTRG.15719 | txndc9 | 3.46 | 2.74 | | 3.04 | | | 12.09 | | 7.93 | | 6.88 | -1.19 | | | 0.03 | down | | |
| MSTRG.1726 | ENSONIG00000017360 | 12.55 | 13.52 | | 18.49 | | | 78.07 | | 52.85 | | 37.09 | -1.58 | | | 0.03 | down | | |
| MSTRG.21853 | xdh | 33.71 | 47.45 | | 31.78 | | | 10.35 | | 13.17 | | 17.72 | 1.43 | | | 0.03 | up | | |
| MSTRG.26716 | ENSONIG00000006704 | 0.51 | 0.60 | | 0.38 | | | 9.15 | | 2.89 | | 7.33 | -1.93 | | | 0.03 | down | | |
| MSTRG.21591 | ENSONIG00000019925 | 2.12 | 2.42 | | 1.89 | | | 8.96 | | 6.18 | | 4.70 | -1.07 | | | 0.03 | down | | |
| MSTRG.16107 | KLF11 (1 of many) | 18.90 | 13.00 | | 20.87 | | | 29.00 | | 50.64 | | 29.42 | -1.26 | | | 0.03 | down | | |
| MSTRG.1689 | ENSONIG00000017255 | 0.21 | 0.26 | | 0.24 | | | 7.22 | | 4.08 | | 2.21 | -1.82 | | | 0.03 | down | | |
| MSTRG.6099 | bco1 | 3.79 | 7.64 | | 4.60 | | | 0.75 | | 1.82 | | 0.67 | 1.75 | | | 0.03 | up | | |
| MSTRG.5329 | pygl | 33.64 | 56.01 | | 53.21 | | | 22.88 | | 28.15 | | 22.30 | 1.09 | | | 0.03 | up | | |
| MSTRG.2668 | thnsl2 | 12.47 | 17.54 | | 21.99 | | | 3.20 | | 4.56 | | 6.46 | 1.71 | | | 0.03 | up | | |
| MSTRG.17129 | ENSONIG00000010731;ENSONIG00000010735 | 8.97 | 10.29 | | 12.22 | | | 1.86 | | 4.10 | | 3.53 | 1.37 | | | 0.03 | up | | |
| MSTRG.10259 | abhd6a | 7.75 | 6.64 | | 10.68 | | | 1.70 | | 3.03 | | 1.20 | 1.55 | | | 0.03 | up | | |
| MSTRG.6390 | ube2q2 | 2.32 | 1.83 | | 3.31 | | | 9.95 | | 8.23 | | 5.98 | -1.34 | | | 0.03 | down | | |
| MSTRG.12498 | ntsr1 | 0.14 | 1.47 | | 0.51 | | | 0 | | 0 | | 0 | 1.03 | | | 0.03 | up | | |
| MSTRG.6977 | slc2a8 | 4.95 | 8.78 | | 11.98 | | | 5.51 | | 3.01 | | 2.41 | 1.46 | | | 0.03 | up | | |
| MSTRG.4733 | prrg4 | 2.40 | 1.10 | | 2.46 | | | 29.27 | | 17.02 | | 9.58 | -2.58 | | | 0.03 | down | | |
| MSTRG.20419 | gltpd2 | 17.30 | 22.38 | | 21.82 | | | 4.64 | | 9.07 | | 7.38 | 1.38 | | | 0.03 | up | | |
| MSTRG.2697 | ENSONIG00000012618 | 0.23 | 0.24 | | 0.28 | | | 3.57 | | 1.36 | | 2.23 | -1.18 | | | 0.03 | down | | |
| MSTRG.9598 | ENSONIG00000010218 | 101.17 | 103.80 | | 97.41 | | | 22.23 | | 46.83 | | 36.56 | 1.33 | | | 0.03 | up | | |
| MSTRG.23964 | ENSONIG00000017461 | 3.27 | 3.25 | | 1.92 | | | 0.42 | | 0.95 | | 0.49 | 1.13 | | | 0.03 | up | | |
| MSTRG.18551 | adgrl4 | 3.47 | 3.25 | | 4.62 | | | 17.83 | | 10.35 | | 9.81 | -1.31 | | | 0.03 | down | | |
| MSTRG.26983 | cinp | 3.66 | 3.26 | | 3.94 | | | 20.47 | | 9.03 | | 14.09 | -1.49 | | | 0.03 | down | | |
| MSTRG.2463 | bsg | 184.52 | 147.91 | | 164.95 | | | 182.35 | | 396.14 | | 318.75 | -1.13 | | | 0.03 | down | | |
| MSTRG.8848 | ENSONIG00000006129 | 75.08 | 237.20 | | 235.39 | | | 80.19 | | 68.40 | | 72.33 | 1.70 | | | 0.03 | up | | |
| MSTRG.14756 | ppifa | 2.76 | 2.50 | | 3.09 | | | 48.82 | | 15.61 | | 16.06 | -2.32 | | | 0.03 | down | | |
| MSTRG.22092 | si:dkey-190l8.2 | 13.13 | 23.45 | | 8.01 | | | 2.48 | | 3.10 | | 2.91 | 2.09 | | | 0.03 | up | | |
| MSTRG.7977 | arg2 | 0.65 | 0.84 | | 1.28 | | | 6.97 | | 3.27 | | 3.58 | -1.22 | | | 0.03 | down | | |
| MSTRG.6625 | tmem55a | 5.06 | 4.33 | | 5.26 | | | 1.52 | | 1.26 | | 0.25 | 1.66 | | | 0.03 | up | | |
| MSTRG.14296 | tfg | 11.02 | 8.48 | | 14.43 | | | 29.38 | | 26.40 | | 22.25 | -1.19 | | | 0.03 | down | | |
| MSTRG.8241 | xiap | 5.72 | 6.28 | | 5.27 | | | 33.54 | | 16.58 | | 15.64 | -1.41 | | | 0.03 | down | | |
| MSTRG.22561 | tram2 | 10.76 | 13.56 | | 14.17 | | | 2.25 | | 3.95 | | 5.19 | 1.50 | | | 0.03 | up | | |
| MSTRG.3635 | sufu | 9.50 | 9.30 | | 6.99 | | | 5.14 | | 3.70 | | 3.08 | 1.05 | | | 0.03 | up | | |
| MSTRG.20583 | kcne4 | 0.11 | 0.05 | | 0.23 | | | 2.05 | | 0.90 | | 1.83 | -1.07 | | | 0.03 | down | | |
| MSTRG.6724 | ENSONIG00000019189 | 8.97 | 0.10 | | 5.69 | | | 3.66 | | 12.27 | | 8.36 | -2.27 | | | 0.03 | down | | |
| MSTRG.23771 | araf | 1.16 | 2.53 | | 1.32 | | | 0.21 | | 0.42 | | 0.48 | 1.08 | | | 0.03 | up | | |
| MSTRG.11791 | krt18 | 89.67 | 57.07 | | 71.52 | | | 218.28 | | 137.44 | | 182.83 | -1.35 | | | 0.03 | down | | |
| MSTRG.15308 | AKAP9 | 17.27 | 35.68 | | 40.02 | | | 5.69 | | 10.02 | | 4.60 | 2.24 | | | 0.03 | up | | |
| MSTRG.1794 | cry-dash;myd88 | 4.13 | 3.37 | | 5.03 | | | 20.02 | | 18.41 | | 9.99 | -1.66 | | | 0.03 | down | | |
| MSTRG.16030 | si:ch211-93g23.2 | 0.50 | 1.63 | | 2.13 | | | 0.21 | | 0.22 | | 0.08 | 1.26 | | | 0.03 | up | | |
| MSTRG.13244 | flcn | 6.76 | 7.36 | | 7.87 | | | 29.22 | | 16.09 | | 16.21 | -1.08 | | | 0.03 | down | | |
| MSTRG.15688 | sept9a | 0.88 | 1.56 | | 1.18 | | | 3.78 | | 3.85 | | 6.65 | -1.28 | | | 0.03 | down | | |
| MSTRG.833 | zgc:153913 | 23.56 | 23.20 | | 22.98 | | | 12.34 | | 5.39 | | 10.47 | 1.48 | | | 0.03 | up | | |
| MSTRG.9270 | zbtb1 | 1.60 | 0.16 | | 0.04 | | | 3.30 | | 2.95 | | 2.48 | -1.74 | | | 0.03 | down | | |
| MSTRG.21899 | F7 | 0 | 28.93 | | 29.86 | | | 22.55 | | 0 | | 0 | 4.46 | | | 0.03 | up | | |
| MSTRG.6167 | ECHDC1 | 5.06 | 5.76 | | 8.56 | | | 1.49 | | 2.60 | | 1.63 | 1.32 | | | 0.03 | up | | |
| MSTRG.3330 | ENSONIG00000000865 | 0.80 | 1.16 | | 0.95 | | | 2.58 | | 7.48 | | 6.48 | -1.85 | | | 0.03 | down | | |
| MSTRG.4422 | gpam | 4.08 | 4.84 | | 5.52 | | | 1.54 | | 2.26 | | 0.92 | 1.24 | | | 0.03 | up | | |
| MSTRG.8426 | si:dkey-96f10.1 | 17.59 | 33.91 | | 15.46 | | | 4.39 | | 6.13 | | 7.11 | 1.85 | | | 0.03 | up | | |
| MSTRG.16001 | astn1 | 5.61 | 7.41 | | 4.20 | | | 1.20 | | 1.97 | | 2.03 | 1.27 | | | 0.03 | up | | |
| MSTRG.7866 | ENSONIG00000019487 | 67.29 | 66.10 | | 52.17 | | | 15.75 | | 30.21 | | 17.43 | 1.37 | | | 0.03 | up | | |
| MSTRG.11566 | ENSONIG00000004728 | 183.92 | 278.22 | | 185.42 | | | 73.42 | | 64.76 | | 24.18 | 2.41 | | | 0.03 | up | | |
| MSTRG.22123 | ftcd | 100.80 | 106.14 | | 69.85 | | | 25.57 | | 15.39 | | 35.98 | 1.95 | | | 0.03 | up | | |
| MSTRG.7919 | slc4a1ap | 1.09 | 0.28 | | 0.62 | | | 7.34 | | 4.37 | | 3.11 | -1.84 | | | 0.03 | down | | |
| MSTRG.21017 | sirt5 | 6.03 | 4.62 | | 5.28 | | | 22.39 | | 16.95 | | 11.37 | -1.43 | | | 0.03 | down | | |
| MSTRG.13705 | si:ch211-220d9.3 | 1.00 | 1.50 | | 1.51 | | | 10.26 | | 4.33 | | 11.11 | -1.67 | | | 0.03 | down | | |
| MSTRG.13022 | ENSONIG00000007551 | 3.86 | 5.30 | | 4.16 | | | 1.09 | | 1.55 | | 2.23 | 1.08 | | | 0.03 | up | | |
| MSTRG.16506 | dbnla | 3.95 | 2.75 | | 2.06 | | | 8.01 | | 6.30 | | 6.48 | -1.10 | | | 0.03 | down | | |
| MSTRG.22565 | ugt5d1 (1 of many) | 11.51 | 5.54 | | 9.07 | | | 1.65 | | 1.80 | | 3.36 | 1.21 | | | 0.03 | up | | |
| MSTRG.16082 | AKIRIN2 (1 of many) | 12.93 | 10.18 | | 12.30 | | | 45.57 | | 25.79 | | 28.53 | -1.29 | | | 0.03 | down | | |
| MSTRG.5365 | gyg1a | 1.23 | 1.84 | | 4.30 | | | 20.15 | | 11.60 | | 11.62 | -1.89 | | | 0.03 | down | | |
| MSTRG.3765 | sptlc3 | 3.59 | 3.22 | | 4.45 | | | 2.20 | | 0.90 | | 1.05 | 1.18 | | | 0.03 | up | | |
| MSTRG.18503 | SLC25A45 | 2.12 | 8.42 | | 10.78 | | | 5.02 | | 1.59 | | 2.36 | 1.70 | | | 0.03 | up | | |
| MSTRG.3832 | dnajc12 | 2.17 | 1.51 | | 1.65 | | | 20.87 | | 8.24 | | 8.58 | -1.98 | | | 0.03 | down | | |
| MSTRG.19342 | socs1b | 2.30 | 3.83 | | 8.44 | | | 0.61 | | 0.42 | | 0.51 | 2.06 | | | 0.04 | up | | |
| MSTRG.17910 | SH2B2 | 2.71 | 5.61 | | 2.73 | | | 3.81 | | 1.26 | | 1.56 | 1.07 | | | 0.04 | up | | |
| MSTRG.24015 | ENSONIG00000009197 | 7.42 | 7.67 | | 6.01 | | | 2.63 | | 3.26 | | 1.50 | 1.25 | | | 0.04 | up | | |
| MSTRG.19064 | stk17a | 0.47 | 1.83 | | 0.41 | | | 0.35 | | 0 | | 0.10 | 1.03 | | | 0.04 | up | | |
| MSTRG.17660 | ENSONIG00000013714 | 5.92 | 4.42 | | 5.62 | | | 1.55 | | 1.72 | | 0.49 | 1.46 | | | 0.04 | up | | |
| MSTRG.20311 | plg (1 of many) | 102.54 | 125.59 | | 98.92 | | | 10.37 | | 18.43 | | 33.55 | 2.31 | | | 0.04 | up | | |
| MSTRG.783 | angptl4 | 2.15 | 1.30 | | 1.16 | | | 11.17 | | 4.88 | | 8.61 | -1.76 | | | 0.04 | down | | |
| MSTRG.16683 | KIAA0430 | 0.59 | 4.18 | | 1.61 | | | 4.13 | | 0.57 | | 0.53 | 1.21 | | | 0.04 | up | | |
| MSTRG.15772 | irs2b | 1.49 | 2.71 | | 1.41 | | | 12.10 | | 5.68 | | 5.48 | -1.15 | | | 0.04 | down | | |
| MSTRG.12170 | a1cf | 349.50 | 415.31 | | 293.39 | | | 61.22 | | 117.75 | | 125.32 | 1.67 | | | 0.04 | up | | |
| MSTRG.11619 | LTBP4 | 18.05 | 19.51 | | 15.26 | | | 41.83 | | 45.81 | | 85.84 | -1.66 | | | 0.04 | down | | |
| MSTRG.19324 | cyp3a65 (1 of many) | 7.59 | 3.47 | | 9.17 | | | 70.10 | | 45.33 | | 26.51 | -2.68 | | | 0.04 | down | | |
| MSTRG.24846 | slc24a3 (1 of many) | 0.23 | 0.43 | | 0.17 | | | 0.95 | | 2.29 | | 2.29 | -1.25 | | | 0.04 | down | | |
| MSTRG.9903 | CD302 | 47.60 | 51.86 | | 39.56 | | | 101.86 | | 109.02 | | 189.86 | -1.51 | | | 0.04 | down | | |
| MSTRG.5314 | hhipl2 | 2.19 | 3.85 | | 2.02 | | | 0.43 | | 0.00 | | 1.00 | 1.57 | | | 0.04 | up | | |
| MSTRG.16972 | atp6v0a1b | 6.43 | 11.27 | | 5.63 | | | 4.31 | | 2.65 | | 4.28 | 1.16 | | | 0.04 | up | | |
| MSTRG.16442 | tmem97 | 66.60 | 70.93 | | 97.06 | | | 10.65 | | 26.96 | | 10.89 | 2.14 | | | 0.04 | up | | |
| MSTRG.15118 | si:ch211-262h13.5 | 265.00 | 198.91 | | 250.46 | | | 42.50 | | 87.76 | | 99.39 | 1.32 | | | 0.04 | up | | |
| MSTRG.5355 | rmnd1 | 1.94 | 3.16 | | 2.09 | | | 0.42 | | 0.95 | | 0.79 | 1.02 | | | 0.04 | up | | |
| MSTRG.14134 | irf1b | 15.95 | 13.55 | | 30.77 | | | 6.49 | | 3.22 | | 3.75 | 2.06 | | | 0.04 | up | | |
| MSTRG.22784 | acox3 | 21.28 | 30.46 | | 24.41 | | | 7.40 | | 10.27 | | 12.92 | 1.28 | | | 0.04 | up | | |
| MSTRG.17289 | mgst2 | 14.11 | 17.11 | | 15.29 | | | 10.99 | | 4.45 | | 8.26 | 1.26 | | | 0.04 | up | | |
| MSTRG.24264 | ENSONIG00000003564 | 6.41 | 13.83 | | 4.57 | | | 1.19 | | 0.33 | | 2.16 | 2.38 | | | 0.04 | up | | |
| MSTRG.16552 | ADORA3 (1 of many) | 1177.18 | 1178.02 | | 868.42 | | | 86.02 | | 55.40 | | 252.56 | 3.29 | | | 0.04 | up | | |
| MSTRG.14247 | GNG10 | 4.63 | 4.82 | | 5.24 | | | 19.85 | | 9.74 | | 16.59 | -1.22 | | | 0.04 | down | | |
| MSTRG.18490 | MIB2 | 4.14 | 8.61 | | 3.02 | | | 24.53 | | 16.33 | | 18.49 | -1.44 | | | 0.04 | down | | |
| MSTRG.9384 | tmem45b | 8.97 | 5.21 | | 10.40 | | | 26.25 | | 24.27 | | 17.14 | -1.52 | | | 0.04 | down | | |
| MSTRG.875 | ENSONIG00000010732 | 952.20 | 303.85 | | 685.60 | | | 600.69 | | 914.21 | | 1251.36 | -1.27 | | | 0.04 | down | | |
| MSTRG.13958 | s100v1 | 3.87 | 4.47 | | 3.36 | | | 26.26 | | 10.48 | | 13.65 | -1.44 | | | 0.04 | down | | |
| MSTRG.15318 | hyi | 35.40 | 37.89 | | 31.58 | | | 8.46 | | 16.03 | | 15.02 | 1.21 | | | 0.04 | up | | |
| MSTRG.2169 | il34 | 0.96 | 1.36 | | 1.30 | | | 11.95 | | 5.64 | | 4.30 | -1.46 | | | 0.04 | down | | |
| MSTRG.5212 | lsm14b | 0.79 | 0.11 | | 0.17 | | | 2.21 | | 1.61 | | 4.26 | -1.64 | | | 0.04 | down | | |
| MSTRG.2149 | map1lc3b | 12.50 | 10.17 | | 8.01 | | | 24.40 | | 18.01 | | 24.77 | -1.09 | | | 0.04 | down | | |
| MSTRG.7828 | agpat3 | 17.37 | 16.22 | | 27.43 | | | 5.73 | | 8.62 | | 6.70 | 1.28 | | | 0.04 | up | | |
| MSTRG.20710 | ENSONIG00000017857 | 0.81 | 1.35 | | 0.62 | | | 20.38 | | 7.98 | | 5.48 | -2.05 | | | 0.04 | down | | |
| MSTRG.3481 | zgc:56576 | 8.18 | 7.82 | | 13.01 | | | 3.22 | | 4.20 | | 3.33 | 1.14 | | | 0.04 | up | | |
| MSTRG.13005 | bmper | 1.24 | 0.41 | | 1.11 | | | 2.93 | | 2.18 | | 2.37 | -1.02 | | | 0.04 | down | | |
| MSTRG.11250 | zmat4a | 1.92 | 3.62 | | 1.24 | | | 9.78 | | 17.24 | | 9.39 | -1.97 | | | 0.04 | down | | |
| MSTRG.10050 | oaz2a | 7.65 | 9.32 | | 11.76 | | | 34.31 | | 18.63 | | 30.02 | -1.16 | | | 0.04 | down | | |
| MSTRG.6585 | ubqln4 | 10.79 | 7.88 | | 14.51 | | | 26.02 | | 24.36 | | 21.81 | -1.16 | | | 0.04 | down | | |
| MSTRG.4366 | CTBP2 | 0.60 | 0.58 | | 0.78 | | | 1.41 | | 3.06 | | 1.74 | -1.00 | | | 0.04 | down | | |
| MSTRG.16553 | ADORA3 (1 of many) | 79.85 | 84.90 | | 75.46 | | | 10.81 | | 7.21 | | 26.74 | 2.57 | | | 0.04 | up | | |
| MSTRG.22540 | ENSONIG00000009379 | 2.88 | 4.03 | | 4.79 | | | 1.17 | | 0.11 | | 1.53 | 1.68 | | | 0.04 | up | | |
| MSTRG.19409 | hsf2 | 3.49 | 9.00 | | 5.27 | | | 5.70 | | 1.27 | | 3.75 | 1.31 | | | 0.04 | up | | |
| MSTRG.21264 | dhrs1 | 30.83 | 35.46 | | 35.01 | | | 11.86 | | 19.53 | | 11.39 | 1.17 | | | 0.04 | up | | |
| MSTRG.3754 | ENSONIG00000010020 | 239.66 | 195.81 | | 131.77 | | | 55.40 | | 81.79 | | 69.85 | 1.19 | | | 0.04 | up | | |
| MSTRG.14410 | si:ch211-225b11.1 | 4.87 | 8.55 | | 8.66 | | | 2.75 | | 3.52 | | 1.57 | 1.43 | | | 0.04 | up | | |
| MSTRG.17093 | pebp1 | 41.57 | 23.52 | | 54.63 | | | 10.11 | | 10.90 | | 11.89 | 1.44 | | | 0.04 | up | | |
| MSTRG.23061 | gyg2 | 5.68 | 5.26 | | 3.32 | | | 7.44 | | 12.82 | | 10.69 | -1.17 | | | 0.04 | down | | |
| MSTRG.12568 | acat2 | 12.91 | 25.53 | | 38.04 | | | 2.03 | | 5.58 | | 2.22 | 2.72 | | | 0.04 | up | | |
| MSTRG.14265 | nedd4l | 2.65 | 2.92 | | 3.32 | | | 18.70 | | 20.92 | | 8.25 | -1.92 | | | 0.04 | down | | |
| MSTRG.13524 | ttc12 | 1.79 | 3.17 | | 1.28 | | | 1.22 | | 0.29 | | 0.68 | 1.15 | | | 0.04 | up | | |
| MSTRG.8308 | nsdhl | 11.49 | 28.45 | | 37.55 | | | 0.72 | | 4.00 | | 1.53 | 3.22 | | | 0.04 | up | | |
| MSTRG.2349 | sin3b | 5.39 | 3.67 | | 4.83 | | | 15.08 | | 9.06 | | 16.46 | -1.37 | | | 0.04 | down | | |
| MSTRG.7997 | bnip3la | 17.95 | 18.54 | | 11.89 | | | 44.42 | | 31.26 | | 41.47 | -1.15 | | | 0.04 | down | | |
| MSTRG.19766 | PSMD2 | 11.08 | 8.74 | | 17.44 | | | 27.62 | | 33.33 | | 26.63 | -1.33 | | | 0.04 | down | | |
| MSTRG.4654 | trip10a | 0.83 | 0.83 | | 0.31 | | | 34.98 | | 10.42 | | 7.41 | -2.78 | | | 0.04 | down | | |
| MSTRG.3793 | tsnax | 2.39 | 3.98 | | 3.70 | | | 30.93 | | 9.66 | | 25.19 | -1.80 | | | 0.04 | down | | |
| MSTRG.25061 | mycb | 0.17 | 0.42 | | 0.68 | | | 73.99 | | 11.11 | | 11.73 | -3.27 | | | 0.04 | down | | |
| MSTRG.2593 | rabggta | 1.15 | 0.78 | | 1.76 | | | 2.93 | | 4.89 | | 2.77 | -1.18 | | | 0.04 | down | | |
| MSTRG.9924 | glb1l | 9.68 | 14.67 | | 19.50 | | | 3.41 | | 5.08 | | 5.29 | 1.52 | | | 0.04 | up | | |
| MSTRG.2455 | ENSONIG00000002356 | 2.42 | 2.01 | | 2.74 | | | 8.33 | | 5.73 | | 4.89 | -1.01 | | | 0.04 | down | | |
| MSTRG.26007 | gemin4 | 0 | 3.81 | | 2.97 | | | 0.05 | | 0 | | 0 | 2.13 | | | 0.04 | up | | |
| MSTRG.19952 | trappc1 | 3.67 | 2.48 | | 4.48 | | | 8.35 | | 6.56 | | 8.12 | -1.01 | | | 0.04 | down | | |
| MSTRG.23486 | tns1b | 4.97 | 3.86 | | 3.89 | | | 13.66 | | 7.87 | | 11.41 | -1.12 | | | 0.04 | down | | |
| MSTRG.18578 | zgc:113516 | 15.29 | 19.29 | | 37.60 | | | 3.31 | | 4.89 | | 1.09 | 2.76 | | | 0.04 | up | | |
| MSTRG.25144 | ENSONIG00000008142 | 1.99 | 1.08 | | 2.93 | | | 0.06 | | 0.10 | | 0.26 | 1.18 | | | 0.04 | up | | |
| MSTRG.7611 | ENSONIG00000010033 | 46.04 | 63.06 | | 33.01 | | | 3.72 | | 4.85 | | 12.97 | 2.63 | | | 0.04 | up | | |
| MSTRG.1263 | hbbe2 | 1.22 | 0 | | 0.44 | | | 37.78 | | 14.29 | | 7.38 | -3.49 | | | 0.04 | down | | |
| MSTRG.4613 | ENSONIG00000001457 | 3676.19 | 2913.09 | | 2181.80 | | | 685.26 | | 39.43 | | 58.85 | 5.44 | | | 0.04 | up | | |
| MSTRG.12937 | slc12a7b | 2.81 | 5.83 | | 2.46 | | | 3.35 | | 0.97 | | 1.22 | 1.26 | | | 0.04 | up | | |
| MSTRG.20009 | arfip1 (1 of many) | 1.99 | 2.25 | | 1.12 | | | 0.72 | | 0.23 | | 0.45 | 1.03 | | | 0.04 | up | | |
| MSTRG.8277 | anxa6 | 21.12 | 25.20 | | 27.74 | | | 8.39 | | 13.85 | | 11.10 | 1.04 | | | 0.04 | up | | |
| MSTRG.4386 | anapc16 | 18.60 | 9.30 | | 10.18 | | | 47.81 | | 28.84 | | 34.65 | -1.65 | | | 0.04 | down | | |
| MSTRG.7207 | ENSONIG00000005270 | 3.25 | 6.49 | | 3.56 | | | 1.20 | | 1.26 | | 2.12 | 1.27 | | | 0.04 | up | | |
| MSTRG.20664 | BCL2L1 (1 of many) | 5.11 | 5.41 | | 1.31 | | | 35.66 | | 22.27 | | 23.02 | -2.47 | | | 0.04 | down | | |
| MSTRG.13249 | ntd5 | 26.69 | 15.88 | | 3.86 | | | 200.28 | | 111.06 | | 218.58 | -3.84 | | | 0.04 | down | | |
| MSTRG.6641 | si:dkeyp-120h9.1 | 1.30 | 3.98 | | 1.25 | | | 1.18 | | 0.67 | | 0.48 | 1.18 | | | 0.04 | up | | |
| MSTRG.9712 | RNF5 | 7.29 | 6.05 | | 6.40 | | | 29.03 | | 20.28 | | 13.92 | -1.40 | | | 0.04 | down | | |
| MSTRG.13267 | slc2a3b | 0.45 | 0.22 | | 0.73 | | | 32.11 | | 7.67 | | 7.55 | -2.81 | | | 0.04 | down | | |
| MSTRG.1352 | h3f3d | 96.76 | 55.62 | | 94.64 | | | 280.92 | | 230.56 | | 163.91 | -1.57 | | | 0.04 | down | | |
| MSTRG.13977 | zgc:171704 | 0.10 | 0 | | 0.08 | | | 2.05 | | 2.08 | | 6.93 | -2.13 | | | 0.04 | down | | |
| MSTRG.20545 | akap8l | 3.87 | 1.94 | | 4.35 | | | 7.23 | | 7.43 | | 6.21 | -1.10 | | | 0.04 | down | | |
| MSTRG.6614 | rbm24a | 4.32 | 4.90 | | 4.37 | | | 25.08 | | 10.59 | | 13.41 | -1.25 | | | 0.04 | down | | |
| MSTRG.15307 | cyp51 | 208.58 | 385.38 | | 435.01 | | | 23.65 | | 78.86 | | 21.53 | 3.22 | | | 0.04 | up | | |
| MSTRG.9281 | ugt5d1 (1 of many) | 47.02 | 23.00 | | 40.64 | | | 6.84 | | 9.05 | | 15.14 | 1.31 | | | 0.04 | up | | |
| MSTRG.10862 | FAIM2 (1 of many) | 0 | 0.03 | | 0.02 | | | 162.61 | | 20.63 | | 12.88 | -4.40 | | | 0.04 | down | | |
| MSTRG.10425 | asmt2 | 2.19 | 3.64 | | 1.53 | | | 6.49 | | 11.90 | | 10.22 | -1.65 | | | 0.04 | down | | |
| MSTRG.20110 | chchd10 | 16.61 | 13.13 | | 23.96 | | | 0.97 | | 2.37 | | 4.80 | 2.12 | | | 0.04 | up | | |
| MSTRG.4864 | CAPRIN1 (1 of many) | 4.68 | 3.01 | | 6.25 | | | 7.19 | | 9.24 | | 9.58 | -1.03 | | | 0.04 | down | | |
| MSTRG.13677 | snx18a | 3.88 | 4.11 | | 3.17 | | | 34.59 | | 11.84 | | 15.82 | -1.72 | | | 0.04 | down | | |
| MSTRG.235 | lpcat2 | 0.46 | 0.38 | | 1.00 | | | 1.63 | | 2.03 | | 2.92 | -1.07 | | | 0.04 | down | | |
| MSTRG.22408 | lamtor3 | 6.37 | 3.27 | | 6.10 | | | 11.38 | | 9.72 | | 9.68 | -1.07 | | | 0.04 | down | | |
| MSTRG.12447 | sbf1 | 2.37 | 2.76 | | 1.51 | | | 4.16 | | 7.05 | | 5.78 | -1.15 | | | 0.04 | down | | |
| MSTRG.20700 | APBB3 | 0.90 | 0.12 | | 0 | | | 1.19 | | 1.10 | | 1.79 | -1.13 | | | 0.04 | down | | |
| MSTRG.1807 | tgif1 | 0.97 | 0.85 | | 0.88 | | | 5.98 | | 2.48 | | 4.40 | -1.25 | | | 0.04 | down | | |
| MSTRG.14705 | pgam1a | 4.99 | 6.28 | | 5.16 | | | 2.80 | | 2.56 | | 1.07 | 1.25 | | | 0.04 | up | | |
| MSTRG.8214 | fn1b | 459.27 | 471.58 | | 409.28 | | | 61.06 | | 155.61 | | 141.50 | 1.69 | | | 0.04 | up | | |
| MSTRG.20654 | slc35c2 | 11.16 | 17.97 | | 6.84 | | | 2.55 | | 2.17 | | 3.83 | 1.85 | | | 0.04 | up | | |
| MSTRG.5175 | ARL8B | 1.22 | 1.95 | | 1.97 | | | 16.97 | | 10.38 | | 5.52 | -1.68 | | | 0.04 | down | | |
| MSTRG.5889 | elovl6 | 24.68 | 22.48 | | 45.60 | | | 1.33 | | 6.48 | | 1.30 | 2.85 | | | 0.04 | up | | |
| MSTRG.6363 | ano5a | 2.20 | 2.04 | | 3.10 | | | 10.97 | | 8.85 | | 5.53 | -1.32 | | | 0.04 | down | | |
| MSTRG.19015 | ENSONIG00000011868 | 7.06 | 4.04 | | 5.96 | | | 19.03 | | 11.27 | | 20.53 | -1.50 | | | 0.04 | down | | |
| MSTRG.7395 | rerea | 16.44 | 17.06 | | 23.26 | | | 38.49 | | 34.95 | | 52.51 | -1.08 | | | 0.04 | down | | |
| MSTRG.15484 | ENSONIG00000006971;ENSONIG00000006973 | 2.89 | 1.97 | | 5.26 | | | 0.23 | | 0.33 | | 0.55 | 1.48 | | | 0.04 | up | | |
| MSTRG.8500 | wu:fb72h05 | 3.90 | 3.78 | | 4.51 | | | 55.99 | | 27.63 | | 15.12 | -2.26 | | | 0.04 | down | | |
| MSTRG.18720 | fnbp1a | 8.31 | 5.53 | | 7.92 | | | 2.93 | | 2.82 | | 1.53 | 1.18 | | | 0.04 | up | | |
| MSTRG.3791 | GPR137B (1 of many) | 0.31 | 0.12 | | 0.77 | | | 4.29 | | 1.92 | | 3.82 | -1.51 | | | 0.04 | down | | |
| MSTRG.13991 | gramd1a | 0.83 | 0.85 | | 2.03 | | | 7.97 | | 3.98 | | 6.23 | -1.46 | | | 0.04 | down | | |
| MSTRG.1538 | slc52a3 | 6.16 | 7.60 | | 6.68 | | | 0.88 | | 2.72 | | 2.11 | 1.34 | | | 0.04 | up | | |
| MSTRG.1412 | TRPM4 | 1.29 | 3.95 | | 1.48 | | | 1.48 | | 0.20 | | 1.17 | 1.23 | | | 0.04 | up | | |
| MSTRG.3274 | fam46ab | 0.51 | 0.32 | | 0.77 | | | 3.57 | | 1.90 | | 2.06 | -1.07 | | | 0.04 | down | | |
| MSTRG.18128 | atp6v1ba | 10.24 | 8.56 | | 11.98 | | | 34.80 | | 21.98 | | 21.36 | -1.14 | | | 0.04 | down | | |
| MSTRG.5202 | id1 | 9.50 | 8.91 | | 9.31 | | | 16.22 | | 34.86 | | 54.82 | -2.00 | | | 0.04 | down | | |
| MSTRG.23012 | ENSONIG00000014659 | 22.21 | 33.22 | | 17.67 | | | 9.16 | | 10.19 | | 11.78 | 1.22 | | | 0.04 | up | | |
| MSTRG.312 | ABCC11 | 75.26 | 263.64 | | 121.30 | | | 1.29 | | 4.36 | | 16.13 | 4.63 | | | 0.04 | up | | |
| MSTRG.6347 | grb10a | 0 | 0.13 | | 0.04 | | | 2.63 | | 1.71 | | 0.87 | -1.12 | | | 0.04 | down | | |
| MSTRG.15798 | si:dkey-186o21.1 | 30.37 | 80.69 | | 24.10 | | | 4.66 | | 4.58 | | 11.18 | 2.78 | | | 0.04 | up | | |
| MSTRG.23090 | COL14A1 (1 of many) | 34.97 | 21.90 | | 17.84 | | | 1.36 | | 5.45 | | 0.61 | 2.68 | | | 0.04 | up | | |
| MSTRG.13259 | isoc2 | 29.16 | 28.05 | | 32.46 | | | 11.34 | | 17.56 | | 9.69 | 1.10 | | | 0.04 | up | | |
| MSTRG.11132 | ptprfb | 1.17 | 2.73 | | 1.54 | | | 14.42 | | 9.51 | | 5.15 | -1.44 | | | 0.04 | down | | |
| MSTRG.4853 | rras2 | 1.46 | 1.25 | | 2.62 | | | 9.52 | | 6.26 | | 5.11 | -1.39 | | | 0.04 | down | | |
| MSTRG.14968 | ucp1 | 97.16 | 44.31 | | 34.47 | | | 178.70 | | 161.90 | | 130.85 | -1.85 | | | 0.04 | down | | |
| MSTRG.8586 | gadd45ga | 13.18 | 16.16 | | 25.26 | | | 183.17 | | 65.19 | | 227.76 | -2.58 | | | 0.04 | down | | |
| MSTRG.23383 | ENSONIG00000000300 | 1.12 | 0.86 | | 2.91 | | | 4.36 | | 7.24 | | 7.25 | -1.70 | | | 0.04 | down | | |
| MSTRG.11041 | si:ch211-193l2.10 | 1.61 | 1.67 | | 1.02 | | | 6.74 | | 3.37 | | 5.22 | -1.14 | | | 0.04 | down | | |
| MSTRG.16962 | cyp26a1 | 18.65 | 11.76 | | 19.65 | | | 36.07 | | 28.19 | | 42.78 | -1.20 | | | 0.04 | down | | |
| MSTRG.20576 | slc25a36b (1 of many) | 27.22 | 20.38 | | 45.05 | | | 153.36 | | 126.98 | | 82.22 | -1.95 | | | 0.04 | down | | |
| MSTRG.19831 | ENSONIG00000017172 | 6.18 | 1.60 | | 7.71 | | | 0 | | 0.02 | | 0 | 2.02 | | | 0.04 | up | | |
| MSTRG.5167 | zgc:113054 | 11.05 | 10.13 | | 15.34 | | | 1.33 | | 4.61 | | 2.32 | 1.61 | | | 0.04 | up | | |
| MSTRG.1579 | apex2 | 0 | 1.12 | | 0.96 | | | 0 | | 0 | | 0 | 1.03 | | | 0.04 | up | | |
| MSTRG.8840 | laptm4a | 22.76 | 10.97 | | 26.37 | | | 67.75 | | 48.88 | | 54.20 | -1.71 | | | 0.04 | down | | |
| MSTRG.602 | AQP4 | 3.07 | 3.35 | | 2.15 | | | 0.26 | | 1.12 | | 0.59 | 1.10 | | | 0.04 | up | | |
| MSTRG.13414 | ZNF800 | 0.39 | 0.37 | | 0.56 | | | 4.13 | | 1.79 | | 2.05 | -1.09 | | | 0.04 | down | | |
| MSTRG.2470 | ENSONIG00000002439 | 0 | 0 | | 0 | | | 3.06 | | 1.12 | | 1.16 | -1.18 | | | 0.04 | down | | |
| MSTRG.19169 | ENSONIG00000012437 | 0.21 | 4.92 | | 0.55 | | | 26.32 | | 7.55 | | 18.70 | -1.90 | | | 0.04 | down | | |
| MSTRG.11104 | vapal | 9.46 | 7.62 | | 18.29 | | | 32.64 | | 36.74 | | 32.56 | -1.62 | | | 0.04 | down | | |
| MSTRG.1407 | ENSONIG00000020073 | 1.77 | 1.56 | | 3.18 | | | 47.28 | | 12.38 | | 15.88 | -2.43 | | | 0.04 | down | | |
| MSTRG.13089 | ENSONIG00000018405 | 1.68 | 3.18 | | 1.11 | | | 0.23 | | 0.31 | | 0.42 | 1.27 | | | 0.04 | up | | |
| MSTRG.5628 | mgst1.1 | 84.93 | 152.03 | | 152.61 | | | 29.74 | | 51.64 | | 19.61 | 2.15 | | | 0.04 | up | | |
| MSTRG.19765 | prss16 | 9.54 | 11.48 | | 13.79 | | | 8.48 | | 4.20 | | 6.96 | 1.02 | | | 0.04 | up | | |
| MSTRG.5571 | s1pr5a | 0 | 0 | | 0 | | | 9.27 | | 1.89 | | 4.21 | -2.04 | | | 0.04 | down | | |
| MSTRG.15349 | dnase2b | 18.60 | 7.28 | | 8.00 | | | 76.60 | | 40.80 | | 37.00 | -2.30 | | | 0.04 | down | | |
| MSTRG.7577 | SYN3 | 1.67 | 1.21 | | 1.26 | | | 29.86 | | 8.05 | | 10.11 | -2.29 | | | 0.04 | down | | |
| MSTRG.2397 | si:zfos-1404b8.2 | 0.03 | 4.37 | | 2.54 | | | 0 | | 0 | | 0.16 | 2.09 | | | 0.04 | up | | |
| MSTRG.15289 | ENSONIG00000019763 | 0.40 | 0.75 | | 0.43 | | | 2.98 | | 2.58 | | 6.46 | -1.59 | | | 0.04 | down | | |
| MSTRG.3634 | zdhhc16a | 2.57 | 3.51 | | 1.29 | | | 0.41 | | 0.49 | | 0.13 | 1.41 | | | 0.04 | up | | |
| MSTRG.16833 | zgc:162171 | 1.33 | 1.68 | | 1.33 | | | 9.97 | | 7.15 | | 3.87 | -1.42 | | | 0.04 | down | | |
| MSTRG.12524 | opcml | 0 | 2.65 | | 2.24 | | | 0 | | 0 | | 0 | 1.78 | | | 0.04 | up | | |
| MSTRG.11078 | fam73a | 0.69 | 0.67 | | 0.54 | | | 2.34 | | 6.12 | | 2.26 | -1.58 | | | 0.04 | down | | |
| MSTRG.25509 | ENSONIG00000006136 | 8.42 | 18.23 | | 8.66 | | | 2.43 | | 4.45 | | 2.88 | 1.70 | | | 0.04 | up | | |
| MSTRG.15724 | ildr2 | 1.51 | 1.22 | | 1.11 | | | 5.90 | | 2.93 | | 5.68 | -1.23 | | | 0.04 | down | | |
| MSTRG.20897 | PXDN | 0.37 | 0.56 | | 0.58 | | | 3.56 | | 1.50 | | 3.32 | -1.07 | | | 0.04 | down | | |
| MSTRG.7320 | PRELID3B | 0.97 | 2.20 | | 2.60 | | | 22.71 | | 14.34 | | 6.87 | -1.87 | | | 0.04 | down | | |
| MSTRG.8724 | MAPRE3 (1 of many) | 5.72 | 6.84 | | 7.17 | | | 36.25 | | 17.54 | | 15.81 | -1.25 | | | 0.04 | down | | |
| MSTRG.5899 | ENSONIG00000001217 | 1.08 | 2.86 | | 1.12 | | | 1.84 | | 0 | | 0 | 1.49 | | | 0.04 | up | | |
| MSTRG.9986 | ENSONIG00000020987 | 9.32 | 12.28 | | 9.99 | | | 2.41 | | 3.91 | | 5.17 | 1.24 | | | 0.04 | up | | |
| MSTRG.2087 | gabarapl2 | 0.17 | 0.42 | | 0.69 | | | 1.32 | | 2.63 | | 2.74 | -1.23 | | | 0.04 | down | | |
| MSTRG.17368 | EPB41 (1 of many) | 11.94 | 19.19 | | 8.53 | | | 7.34 | | 4.91 | | 5.82 | 1.24 | | | 0.04 | up | | |
| MSTRG.9922 | pttg1ipb | 0.56 | 0.92 | | 0.73 | | | 8.37 | | 2.72 | | 3.73 | -1.27 | | | 0.04 | down | | |
| MSTRG.11209 | si:ch73-247j11.2 | 4.96 | 3.28 | | 2.08 | | | 0.86 | | 0.33 | | 0.52 | 1.40 | | | 0.04 | up | | |
| MSTRG.23362 | ENSONIG00000011530 | 4.42 | 5.67 | | 7.70 | | | 1.23 | | 2.35 | | 2.10 | 1.23 | | | 0.04 | up | | |
| MSTRG.22261 | ENSONIG00000021213;ENSONIG00000020594 | 5.27 | 16.05 | | 10.77 | | | 0.52 | | 1.42 | | 2.67 | 2.42 | | | 0.04 | up | | |
| MSTRG.18555 | ENSONIG00000002698 | 1.50 | 3.10 | | 1.62 | | | 0.23 | | 0.31 | | 0.83 | 1.21 | | | 0.04 | up | | |
| MSTRG.1212 | aco2 | 7.19 | 4.96 | | 3.65 | | | 13.91 | | 13.12 | | 9.75 | -1.22 | | | 0.04 | down | | |
| MSTRG.1701 | ENSONIG00000017271 | 4.08 | 5.92 | | 8.56 | | | 2.02 | | 2.64 | | 2.03 | 1.22 | | | 0.04 | up | | |
| MSTRG.9282 | ugt5d1 (1 of many) | 81.88 | 48.47 | | 69.36 | | | 7.89 | | 12.08 | | 25.21 | 1.76 | | | 0.04 | up | | |
| MSTRG.2779 | si:ch211-22d5.2 | 4.11 | 3.37 | | 7.22 | | | 14.67 | | 38.11 | | 14.60 | -2.17 | | | 0.05 | down | | |
| MSTRG.15692 | sept9a | 3.07 | 48.70 | | 3.61 | | | 1.33 | | 0.21 | | 1.41 | 3.58 | | | 0.05 | up | | |
| MSTRG.10294 | rem1 | 0.82 | 0.77 | | 0.98 | | | 8.68 | | 3.46 | | 3.65 | -1.40 | | | 0.05 | down | | |
| MSTRG.22354 | zgc:55781 | 7.99 | 6.56 | | 8.43 | | | 20.06 | | 18.47 | | 12.30 | -1.03 | | | 0.05 | down | | |
| MSTRG.12997 | prdm1a | 0.32 | 0.04 | | 0.19 | | | 2.02 | | 1.76 | | 0.83 | -1.11 | | | 0.05 | down | | |
| MSTRG.808 | mtf2 | 1.37 | 1.70 | | 1.95 | | | 0 | | 0.62 | | 0 | 1.12 | | | 0.05 | up | | |
| MSTRG.20204 | cx28.9 | 3.06 | 26.50 | | 33.19 | | | 315.63 | | 207.88 | | 160.63 | -2.75 | | | 0.05 | down | | |
| MSTRG.5390 | ptbp1a | 0.38 | 2.41 | | 0.56 | | | 5.59 | | 8.27 | | 6.43 | -1.69 | | | 0.05 | down | | |
| MSTRG.23738 | ENSONIG00000020391 | 4.68 | 22.90 | | 8.02 | | | 8.09 | | 3.11 | | 7.53 | 1.48 | | | 0.05 | up | | |
| MSTRG.20883 | sugct | 4.17 | 10.81 | | 3.64 | | | 0.35 | | 1.09 | | 1.08 | 2.05 | | | 0.05 | up | | |
| MSTRG.15479 | ENSONIG00000011846 | 0.34 | 0.87 | | 0.74 | | | 8.41 | | 2.38 | | 3.94 | -1.25 | | | 0.05 | down | | |
| MSTRG.17795 | dgkza | 1.56 | 4.05 | | 2.34 | | | 0.66 | | 0.95 | | 1.12 | 1.12 | | | 0.05 | up | | |
| MSTRG.6299 | ST3GAL1 (1 of many) | 1.18 | 0 | | 0 | | | 1.20 | | 1.43 | | 3.65 | -1.63 | | | 0.05 | down | | |
| MSTRG.12383 | lrba | 5.87 | 1.72 | | 5.54 | | | 14.41 | | 16.36 | | 8.89 | -1.86 | | | 0.05 | down | | |
| MSTRG.2932 | BRSK2 (1 of many) | 0.80 | 2.18 | | 0.50 | | | 0.23 | | 0 | | 0.16 | 1.16 | | | 0.05 | up | | |
| MSTRG.4789 | nedd4a | 3.20 | 1.95 | | 2.92 | | | 12.49 | | 6.78 | | 7.18 | -1.34 | | | 0.05 | down | | |
| MSTRG.73 | tmem220 | 23.93 | 23.36 | | 27.26 | | | 3.60 | | 9.63 | | 8.67 | 1.44 | | | 0.05 | up | | |
| MSTRG.11597 | ece2b | 0.58 | 0.13 | | 0.31 | | | 5.96 | | 4.16 | | 1.88 | -1.81 | | | 0.05 | down | | |
| MSTRG.25143 | ENSONIG00000008142 | 1.02 | 1.03 | | 2.09 | | | 0.05 | | 0.12 | | 0.29 | 1.00 | | | 0.05 | up | | |
| MSTRG.7426 | slc16a4 | 0.31 | 3.41 | | 0.85 | | | 0.17 | | 0.14 | | 0.16 | 1.46 | | | 0.05 | up | | |
| MSTRG.9218 | tmem30ab | 6.50 | 5.94 | | 5.15 | | | 28.27 | | 15.87 | | 13.43 | -1.32 | | | 0.05 | down | | |
| MSTRG.17189 | prkcz | 387.87 | 182.15 | | 142.06 | | | 740.23 | | 600.19 | | 548.64 | -1.80 | | | 0.05 | down | | |
| MSTRG.4793 | cgnl1 | 2.65 | 6.14 | | 3.26 | | | 1.73 | | 1.74 | | 2.24 | 1.00 | | | 0.05 | up | | |
| MSTRG.7239 | zgc:153018 | 0.22 | 0.25 | | 0.25 | | | 9.77 | | 3.36 | | 2.66 | -1.81 | | | 0.05 | down | | |
| MSTRG.15862 | dctd (1 of many) | 0 | 2.25 | | 0.87 | | | 0.07 | | 0 | | 0.18 | 1.28 | | | 0.05 | up | | |
| MSTRG.7764 | fbxw5 | 4.40 | 5.03 | | 4.13 | | | 1.66 | | 2.31 | | 0.84 | 1.15 | | | 0.05 | up | | |
| MSTRG.13035 | ppargc1a | 1.00 | 0.38 | | 0.99 | | | 3.09 | | 4.85 | | 1.82 | -1.41 | | | 0.05 | down | | |
| MSTRG.12294 | fbp1b | 393.73 | 388.15 | | 226.92 | | | 739.56 | | 721.90 | | 717.17 | -1.17 | | | 0.05 | down | | |
| MSTRG.5903 | ctso | 4.44 | 8.15 | | 7.36 | | | 2.32 | | 2.89 | | 3.68 | 1.09 | | | 0.05 | up | | |
| MSTRG.10846 | acvr1bb | 2.09 | 1.18 | | 1.63 | | | 9.38 | | 4.46 | | 5.43 | -1.40 | | | 0.05 | down | | |
| MSTRG.26946 | ENSONIG00000009177 | 25.41 | 73.43 | | 23.60 | | | 21.29 | | 2.44 | | 12.63 | 2.80 | | | 0.05 | up | | |
| MSTRG.5085 | bcat2;hsd17b14 | 39.91 | 90.16 | | 38.77 | | | 38.74 | | 28.57 | | 30.55 | 1.10 | | | 0.05 | up | | |
| MSTRG.6466 | ADAMTS20 | 0.03 | 3.82 | | 1.80 | | | 0.05 | | 0.12 | | 0.11 | 1.80 | | | 0.05 | up | | |
| MSTRG.16169 | zgc:56409 | 2.03 | 1.74 | | 2.95 | | | 3.88 | | 5.03 | | 7.15 | -1.10 | | | 0.05 | down | | |
| MSTRG.16373 | dpys | 18.00 | 10.57 | | 27.51 | | | 0.93 | | 4.76 | | 2.26 | 1.96 | | | 0.05 | up | | |
| MSTRG.20520 | crtc3 | 0 | 1.47 | | 0.94 | | | 6.68 | | 9.83 | | 4.31 | -1.81 | | | 0.05 | down | | |
| MSTRG.12012 | inppl1a | 0.13 | 0 | | 0 | | | 1.87 | | 1.43 | | 0.66 | -1.07 | | | 0.05 | down | | |
| MSTRG.2706 | MBD2 | 3.35 | 2.67 | | 2.92 | | | 13.13 | | 7.00 | | 7.16 | -1.17 | | | 0.05 | down | | |
| MSTRG.4005 | BHLHE40 (1 of many) | 6.33 | 7.86 | | 3.12 | | | 18.43 | | 16.75 | | 18.39 | -1.47 | | | 0.05 | down | | |
| MSTRG.7732 | f9a | 30.55 | 47.97 | | 46.41 | | | 2.46 | | 8.44 | | 9.69 | 2.40 | | | 0.05 | up | | |
| MSTRG.725 | map2k2b | 3.26 | 3.55 | | 3.50 | | | 11.34 | | 10.63 | | 6.09 | -1.08 | | | 0.05 | down | | |
| MSTRG.13299 | rbp5 (1 of many) | 91.45 | 133.99 | | 121.54 | | | 27.95 | | 47.74 | | 52.44 | 1.42 | | | 0.05 | up | | |
| MSTRG.16632 | ENSONIG00000009574 | 5.26 | 9.29 | | 9.24 | | | 1.84 | | 3.49 | | 2.84 | 1.33 | | | 0.05 | up | | |
| MSTRG.16629 | gria4b | 0.16 | 2.74 | | 0.57 | | | 0.84 | | 0.28 | | 0.33 | 1.00 | | | 0.05 | up | | |
| MSTRG.5895 | egf | 0.98 | 1.96 | | 2.82 | | | 0.03 | | 0.18 | | 0.48 | 1.33 | | | 0.05 | up | | |
| MSTRG.20061 | spred2a | 0.52 | 0.02 | | 0.33 | | | 1.97 | | 1.37 | | 1.01 | -1.00 | | | 0.05 | down | | |
| MSTRG.8975 | ENSONIG00000006597 | 288.49 | 174.55 | | 392.68 | | | 2262.17 | | 1896.16 | | 807.81 | -2.53 | | | 0.05 | down | | |
| MSTRG.5641 | PMM1 | 1.20 | 1.08 | | 0.27 | | | 11.20 | | 4.38 | | 15.64 | -2.39 | | | 0.05 | down | | |
| MSTRG.22855 | ENSONIG00000021280 | 0.07 | 1.70 | | 0.55 | | | 0 | | 0 | | 0.08 | 1.08 | | | 0.05 | up | | |
| MSTRG.8097 | PXN (1 of many) | 2.78 | 2.56 | | 2.65 | | | 13.08 | | 6.41 | | 6.75 | -1.15 | | | 0.05 | down | | |
| MSTRG.18847 | cnih3 | 3.72 | 4.35 | | 2.67 | | | 0.53 | | 1.21 | | 1.38 | 1.08 | | | 0.05 | up | | |
| MSTRG.655 | arl14 | 0.93 | 1.44 | | 0.95 | | | 6.84 | | 5.06 | | 2.79 | -1.19 | | | 0.05 | down | | |
| MSTRG.25667 | ENSONIG00000015143;ENSONIG00000015141 | 1.96 | 5.69 | | 2.50 | | | 1.28 | | 1.52 | | 1.11 | 1.17 | | | 0.05 | up | | |
| MSTRG.11474 | stil | 0.82 | 1.09 | | 0.55 | | | 1.76 | | 2.65 | | 3.58 | -1.06 | | | 0.05 | down | | |
| MSTRG.11189 | add2 | 1.98 | 1.74 | | 1.55 | | | 1.07 | | 0.24 | | 0.20 | 1.07 | | | 0.05 | up | | |
| MSTRG.6736 | mcf2l2 | 1.18 | 1.28 | | 1.47 | | | 7.52 | | 3.53 | | 3.65 | -1.04 | | | 0.05 | down | | |
| MSTRG.5565 | junbb | 0 | 0 | | 0.34 | | | 3.15 | | 1.29 | | 4.15 | -1.59 | | | 0.05 | down | | |
| MSTRG.8254 | afap1l1a | 0.14 | 1.50 | | 0.97 | | | 0 | | 0.16 | | 0 | 1.07 | | | 0.05 | up | | |
| MSTRG.4861 | plekha7b | 1.29 | 3.18 | | 1.55 | | | 0.23 | | 0.61 | | 0.54 | 1.17 | | | 0.05 | up | | |
| MSTRG.13617 | chrnb2b | 3.10 | 0.76 | | 1.43 | | | 9.31 | | 4.73 | | 6.84 | -1.78 | | | 0.05 | down | | |
| MSTRG.3561 | APP (1 of many) | 4.41 | 0 | | 4.38 | | | 10.22 | | 10.43 | | 10.72 | -2.62 | | | 0.05 | down | | |
| MSTRG.1534 | ENSONIG00000016742 | 0.86 | 2.85 | | 1.38 | | | 0.09 | | 0.44 | | 0.31 | 1.24 | | | 0.05 | up | | |
| MSTRG.4888 | gbgt1l2 | 3.22 | 5.25 | | 6.12 | | | 1.74 | | 1.70 | | 2.74 | 1.08 | | | 0.05 | up | | |
| MSTRG.25612 | ENSONIG00000017773 | 39.00 | 41.51 | | 27.76 | | | 11.07 | | 13.88 | | 5.17 | 1.85 | | | 0.05 | up | | |
| MSTRG.1859 | OTULIN | 2.80 | 4.61 | | 2.90 | | | 1.94 | | 0.81 | | 2.23 | 1.04 | | | 0.05 | up | | |
| MSTRG.8579 | oxct1b | 30.89 | 49.18 | | 26.59 | | | 11.15 | | 12.96 | | 17.88 | 1.37 | | | 0.05 | up | | |
| MSTRG.2110 | cyb5r2 | 314.62 | 337.09 | | 475.98 | | | 75.98 | | 169.57 | | 95.21 | 1.60 | | | 0.05 | up | | |
| MSTRG.7358 | kazna (1 of many) | 3.25 | 14.59 | | 7.05 | | | 3.59 | | 3.71 | | 3.40 | 1.40 | | | 0.05 | up | | |
| MSTRG.14264 | prlr | 0.06 | 0.06 | | 0.09 | | | 4.32 | | 2.77 | | 1.17 | -1.54 | | | 0.05 | down | | |
