# Supplementary material for: Multi-omics analysis reveals the glycolipid metabolism response mechanism in the liver of genetically improved farmed Tilapia (GIFT, Oreochromis niloticus) under hypoxia stress
Source: BMC Genomics. 2021 Feb 6;22:105. doi: 10.1186/s12864-021-07410-x (PMC7866651; doi:10.1186/s12864-021-07410-x)
Supplement: Supplementary file 10 — Additional file 10: Table S2. P values of top 20 enriched pathways under hypoxia stress. [file 12864_2021_7410_MOESM10_ESM.docx]

Table S2 *P* values of top 20 enriched pathways under hypoxia stress

| Pathway_id | Pathway_name | *P* value |
| --- | --- | --- |
| ko00980 | Metabolism of xenobiotics by cytochrome P450 | 2.33E-07 |
| ko00982 | Drug metabolism - cytochrome P450 | 2.33E-07 |
| ko00640 | Propanoate metabolism | 8.98E-07 |
| ko00280 | Valine, leucine and isoleucine degradation | 1.09E-06 |
| ko00010 | Glycolysis / Gluconeogenesis | 1.25E-06 |
| ko00380 | Tryptophan metabolism | 7.12E-06 |
| ko00051 | Fructose and mannose metabolism | 1.70E-05 |
| ko03320 | PPAR signaling pathway | 1.90E-05 |
| ko00071 | Fatty acid metabolism | 8.69E-05 |
| ko00983 | Drug metabolism - other enzymes | 3.44E-04 |
| ko00330 | Arginine and proline metabolism | 3.45E-04 |
| ko00830 | Retinol metabolism | 4.08E-04 |
| ko00770 | Pantothenate and CoA biosynthesis | 8.16E-04 |
| ko00650 | Butanoate metabolism | 1.20E-03 |
| ko00410 | beta-Alanine metabolism | 1.46E-03 |
| ko04146 | Peroxisome | 1.50E-03 |
| ko00140 | Steroid hormone biosynthesis | 1.80E-03 |
| ko04910 | Insulin signaling pathway | 1.96E-03 |
| ko00120 | Primary bile acid biosynthesis | 2.44E-03 |
| ko00670 | One carbon pool by folate | 2.44E-03 |
