# Supplementary material for: Multi-omics analysis reveals the glycolipid metabolism response mechanism in the liver of genetically improved farmed Tilapia (GIFT, Oreochromis niloticus) under hypoxia stress
Source: BMC Genomics. 2021 Feb 6;22:105. doi: 10.1186/s12864-021-07410-x (PMC7866651; doi:10.1186/s12864-021-07410-x)
Supplement: Supplementary file 11 — Additional file 11: Table S3. Sequences of primers used to amplify differentially expressed mRNAs. [file 12864_2021_7410_MOESM11_ESM.docx]

Table S3 Sequences of primers used to amplify differentially expressed mRNAs.

| Gene abbreviation | Sequence |
| --- | --- |
| PCK1 | F:5’- ATCCCCAAAACAGGCCTCAG -3’ |
|  | R:5’- ACGTACATGGTGCGACCTTT-3’ |
| INSR | F:5’- ATGGTGATGAGGAGCTGCAT -3’ |
|  | R:5’- ACGTAGAAATAGGTGGGTTCCG -3’ |
| HSPB1 | F:5’- CGCGGAAATACACGCTGC -3’ |
|  | R:5’- CGGATTTTGCAGCTTCTGGG -3’ |
| MB | F:5’- GACACCCAGTGAGCCCATAC -3’ |
|  | R:5’- GGTGACCCTTAAAGAGCCTGA -3’ |
| GAPDHS | F:5’- TACACCGAGGATGAGGTCGT -3’ |
|  | R:5’- TCCCGTTTCACTTGTCTCGG-3’ |
| ELOVL6 | F:5’- ACAGTTCAACGAGGACGAAGC -3’ |
|  | R:5’- AGCAAGGGTGAGTGACCACAG -3’ |
| ACAT2 | F:5’- CCGGAAGATGTGTCTGAGGT -3’ |
|  | R:5’- TGCAACCACAATGCTGGAGT -3’ |
| LDHA | F:5’- ACGTACTGCATTTGCCCCTT -3’ |
|  | R:5’- ACTGGATCCCAGGATGTGAC -3’ |
